# Key *FAD2*, *FAD3*, and *SAD* Genes Involved in the Fatty Acid Synthesis in Flax Identified Based on Genomic and Transcriptomic Data

**DOI:** 10.3390/ijms241914885

**Published:** 2023-10-04

**Authors:** Ekaterina M. Dvorianinova, Olga L. Zinovieva, Elena N. Pushkova, Daiana A. Zhernova, Tatiana A. Rozhmina, Liubov V. Povkhova, Roman O. Novakovskiy, Elizaveta A. Sigova, Anastasia A. Turba, Elena V. Borkhert, George S. Krasnov, Chengjiang Ruan, Alexey A. Dmitriev, Nataliya V. Melnikova

**Affiliations:** 1 Engelhardt Institute of Molecular Biology, Russian Academy of Sciences, Moscow 119991, Russia; 2 Faculty of Biology, Lomonosov Moscow State University, Moscow 119234, Russia; 3 Federal Research Center for Bast Fiber Crops, Torzhok 172002, Russia; 4 Moscow Institute of Physics and Technology, Moscow 141701, Russia; 5 Key Laboratory of Biotechnology and Bioresources Utilization, Ministry of Education, Institute of Plant Resources, Dalian Minzu University, Dalian 116600, China

**Keywords:** flax, *Linum usitatissimum* L., unsaturated fatty acids, desaturases, *FAD*, *SAD*, phylogenetic analysis, expression level

## Abstract

*FAD* (fatty acid desaturase) and *SAD* (stearoyl-ACP desaturase) genes play key roles in the synthesis of fatty acids (FA) and determination of oil composition in flax (*Linum usitatissimum* L.). We searched for *FAD* and *SAD* genes in the most widely used flax genome of the variety CDC Bethune and three available long-read assembled flax genomes—YY5, 3896, and Atlant. We identified fifteen *FAD2*, six *FAD3*, and four *SAD* genes. Of all the identified genes, 24 were present in duplicated pairs. In most cases, two genes from a pair differed by a significant number of gene-specific SNPs (single nucleotide polymorphisms) or even InDels (insertions/deletions), except for *FAD2a-1* and *FAD2a-2*, where only seven SNPs distinguished these genes. Errors were detected in the *FAD2a-1*, *FAD2a-2*, *FAD3c-1*, and *FAD3d-2* sequences in the CDC Bethune genome assembly but not in the long-read genome assemblies. Expression analysis of the available transcriptomic data for different flax organs/tissues revealed that *FAD2a-1*, *FAD2a-2*, *FAD3a*, *FAD3b*, *SAD3-1*, and *SAD3-2* were specifically expressed in embryos/seeds/capsules and could play a crucial role in the synthesis of FA in flax seeds. In contrast, *FAD2b-1*, *FAD2b-2*, *SAD2-1*, and *SAD2-2* were highly expressed in all analyzed organs/tissues and could be involved in FA synthesis in whole flax plants. *FAD2c-2*, *FAD2d-1*, *FAD3c-1*, *FAD3c-2*, *FAD3d-1*, *FAD3d-2*, *SAD3-1*, and *SAD3-2* showed differential expression under stress conditions—*Fusarium oxysporum* infection and drought. The obtained results are essential for research on molecular mechanisms of fatty acid synthesis, *FAD* and *SAD* editing, and marker-assisted and genomic selection for breeding flax varieties with a determined fatty acid composition of oil.

## 1. Introduction

Stearoyl-ACP (acyl carrier protein) desaturases (SADs) and fatty acid desaturases (FADs) regulate the biosynthesis of unsaturated fatty acids (FAs) in plants [1]. SADs introduce a double bond at the ∆9 position of hydrocarbon chains of C18-carbon saturated FAs (C18:0) and form monounsaturated FAs (C18:1) [2]. FAD2 enzymes introduce a double bond at the ∆12 position of monounsaturated FAs (C18:1) and, thus, convert them to polyunsaturated FAs (PUFAs) with two double bonds (C18:2) [3]. FAD3 enzymes create one more double bond at the ∆15 position of C18:2 FAs, yielding PUFAs with three double bonds (C18:3) [4].

Genes encoding SADs and FADs were identified and characterized in *Arabidopsis* and other plant species, including canola, olive, sunflower, sesame, corn, rice, wheat, soybean, cotton, chickpea, barrelclover, *Perilla*, *Cyperus esculentus*, peanut, chia, *Brassica*, oil tea, and poplar [3,5,6,7,8,9,10,11,12,13,14,15,16,17,18,19,20,21,22,23,24,25,26,27,28,29,30]. *SAD* and *FAD* genes in flax (*Linum usitatissimum* L.) have attracted special attention, as its seeds are among the richest plant sources of polyunsaturated linolenic acid (C18:3). Thus, they are traditionally used in food, pharmaceuticals, feed, and polymer production [31,32,33,34,35,36,37]. The following flax *SAD* and *FAD* genes were identified first: *SAD* genes (*SAD1* and *SAD2*), which convert stearic acid (STE, C18:0) into oleic acid (OLE, C18:1) [38,39,40]; *FAD2* genes (*FAD2-2* and *FAD2*), which convert OLE (C18:1) into linoleic acid (LIO, C18:2) [41,42]; and *FAD3* genes (*FAD3A*, *FAD3B*, and *FAD3C*), which convert LIO (C18:2) into α-linolenic acid (LIN, C18:3) [43,44].

The sequencing of the whole genome of the linseed variety CDC Bethune [45] allowed You et al. to identify new *SAD* and *FAD* genes. Thus, 25 genes were revealed, 24 of which were duplicated and formed 12 pairs: four *SAD* genes—*SAD2-1* (*SAD1* according to [40])/*SAD2-2* (*SAD2* according to [40]) and *SAD3-1*/*SAD3-2*; fifteen *FAD2* genes—*FAD2a-1*/*FAD2a-2* (*FAD2* according to [42]), *FAD2b-1*/*FAD2b-2* (*FAD2-2* according to [41]), *FAD2c-1*/*FAD2c-2*, *FAD2d-1*/*FAD2d-2*, *FAD2e-1*/*FAD2e-2*, *FAD2f-1*/*FAD2f-2*, *FAD2g-1*/*FAD2g-2*, and *FAD2h*; and six *FAD3* genes—*FAD3a*/*FAD3b*, *FAD3c-1*/*FAD3c-2*, and *FAD3d-1*/*FAD3d-2*. Errors were detected in the *FAD2a-1* and *FAD2a-2* gene sequences in the CDC Bethune genome assembly (https://phytozome-next.jgi.doe.gov/info/Lusitatissimum_v1_0, accessed on 1 August 2023) [46]. The inaccurate genome sequence complicated molecular genetic studies and editing of the *FAD2* genes.

For *SAD* and *FAD*, You et al. [46] examined the large-scale generation of expressed sequence tags (ESTs) [47] and assessed expression in embryo, endosperm, seed coat, seedlings, stem, leaf, flower, and boll. Due to the high similarity between the gene sequences of duplicated pairs (*FAD2a-1*/*FAD2a-2*, *FAD2b-1*/*FAD2b-2*, etc.), expression was evaluated only for gene pairs instead of individual genes, except for *FAD3a*/*FAD3b*. Among all studied *FAD2* genes, *FAD2b-1*/*FAD2b-2* had the highest number of EST hits. Fewer ESTs were identified for *FAD2a-1*/*FAD2a-2*. The smallest number of ESTs were revealed for other *FAD2* genes. *FAD2b-1*/*FAD2b-2* and *FAD2a-1*/*FAD2a-2* were predominantly expressed in mature embryos. Among the *FAD3* genes, *FAD3a*/*FAD3b* had the highest number of EST hits, predominantly in mature embryos. For the *SAD2-1/SAD2-2* and *SAD3-1/SAD3-2* genes, some EST hits were revealed in different organs/tissues and at various developmental stages of flax plants. You et al. suggested that *FAD2a-1*/*FAD2a-2*, *FAD2b-1*/*FAD2b-2*, and *FAD3a*/*FAD3b* play a crucial role in the synthesis of linseed oil FAs [46]. The same role of these genes was also demonstrated in other studies [44,48,49,50].

In the work by Thambugala et al., *SAD* and *FAD* loci were compared between the high-LIN cultivar CDC Bethune and the low-LIN line M5791 using bacterial artificial chromosome clones. This allowed researchers to gain insight into the structural organization and diversity of these regions of the flax genome [51].

In the past few years, the genome assembly of CDC Bethune has been significantly improved with different techniques, including optical mapping [52]. Moreover, several genome assemblies of other flax genotypes were obtained from the third-generation sequencing and/or Hi-C data [53,54,55,56]. Long reads allow reliable assembly of highly homologous contiguous loci and enable the evaluation of the differences between pairs of duplicated genes [57,58]. The obtained flax genomes opened up new opportunities for the identification and comparison of the *SAD*, *FAD2*, and *FAD3* genes of different flax genotypes. In addition to whole-genome sequencing, a significant number of transcriptomes have been obtained for flax plants in recent years [59,60,61,62,63,64,65,66,67,68,69,70,71,72,73,74,75]. Such studies enabled extensive analysis of *SAD*, *FAD2*, and *FAD3* expression in different organs/tissues and during various developmental stages and under various stress conditions. The obtained data are necessary to determine the role of *SAD* and *FAD* in FA synthesis and other processes in flax plants. Our work aimed at the genome-wide identification of *FAD2*, *FAD3*, and *SAD* genes in the most frequently used CDC Bethune genome and three flax genomes sequenced on third-generation platforms. We also analyzed the expression of these genes in different flax organs/tissues and under different stress conditions.

## 2. Results

### 2.1. Identification of FAD2 Genes in Flax Genome Assemblies

Using sequences of the *FAD2* genes from the study of You et al. [46], we identified the location of the *FAD2* genes in four analyzed genomes: fiber flax YY5 (Yiya No. 5; from PacBio HiFi and Hi-C data [54]); linseed line 3896 (from Nanopore data [56]); fiber flax Atlant (from Nanopore data [55]); and linseed CDC Bethune (from Illumina and optical mapping data [52]). Gene coordinates are presented in Table 1.

Fifteen *FAD2* genes were identified in each studied genome, except for that of cultivar Atlant. In this genotype, additional FAD2b-2* and FAD2c-1* genes were revealed. These copies were localized in one contig (JACHUY010001688.1). Several additional SNPs (single nucleotide polymorphisms) and short InDels (insertions/deletions) were revealed in FAD2c-1* compared to other *FAD2c* genes identified in the studied flax varieties. The Atlant genome assembly is less contiguous than two other long-read flax genome assemblies (N50 = 0.4 Mb for Atlant, N50 = 6.2 Mb for 3896, and N50 = 9.6 Mb for YY5) [54,55,56]. Therefore, the presence of two additional *FAD2* genes is unlikely to be the Atlant genotype feature but could be associated with errors in the genome assembly. To avoid inaccuracies, we excluded the FAD2b-2* and FAD2c-1* genes of Atlant from further analysis.

In the genomes of the four studied flax varieties, the *FAD2* genes were located similarly. *FAD2a-1* and *FAD2a-2* were on two different chromosomes/contigs, and the other thirteen *FAD2* genes were organized into two clusters. *FAD2b-1*, *FAD2c-1*, *FAD2d-1*, *FAD2e-1*, *FAD2f-1*, *FAD2g-1*, and *FAD2h* were located on the same chromosome/contig in a 31-32-kb region, whereas *FAD2b-2*, *FAD2c-2*, *FAD2d-2*, *FAD2e-2*, *FAD2f-2*, and *FAD2g-2* were in about a 28-kb region on another chromosome/contig. However, the analyzed flax varieties slightly differed in the distances between the clustered *FAD2* genes (Table 2).

### 2.2. Phylogenetic Analysis of FAD2 Genes of Different Flax Varieties

**Figure 1:**
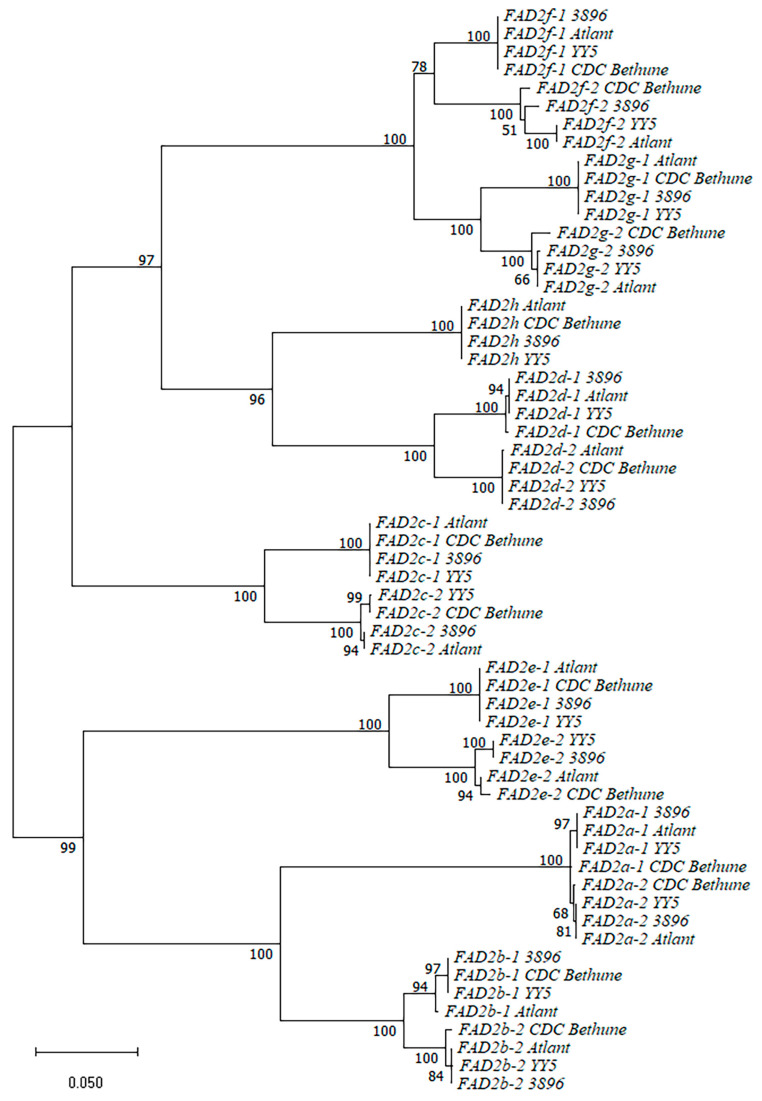
Phylogenetic analysis of *FAD2* genes of flax varieties YY5, 3896, Atlant, and CDC Bethune.

We analyzed sequences of 60 *FAD2* genes that we identified in four flax varieties (15 genes per variety) (sequences are presented in Supplementary Table S1). A dendrogram for the *FAD2* gene sequences is presented in Figure 1. The sequences of the same *FAD2* genes of different varieties clustered with each other and formed subclusters. However, two genes were exceptions: *FAD2a-1* and *FAD2a-2* of CDC Bethune. For these genes, You et al. showed the presence of mis-assemblies in the previous version of the CDC Bethune genome (https://phytozome-next.jgi.doe.gov/info/Lusitatissimum_v1_0, accessed on 1 August 2023) [46]. Probably, the current version of the CDC Bethune genome assembly (NCBI GenBank, GCA_000224295.2) can still have errors in the *FAD2a-1* and *FAD2a-2* sequences. Indeed, we found a long deletion in *FAD2a-1* and an insertion in *FAD2a-2* of CDC Bethune compared to these genes of varieties YY5, 3896, and Atlant. In the YY5, 3896, and Atlant genomes, the *FAD2a-1* gene sequences were identical; the same was true for the *FAD2a-2* gene. *FAD2a-1* differed from *FAD2a-2* by only seven SNPs in all three varieties. Thus, *FAD2a-1* and *FAD2a-2* are very conserved in flax and can be distinguished one from another by seven SNPs. Notably, one part of the *FAD2a-1* gene of CDC Bethune was similar to the *FAD2a-2* gene of YY5, 3896, and Atlant, but not *FAD2a-1* of these varieties. This also indicated an error in the *FAD2a-1* sequence of CDC Bethune.

Generally, all pairs of the duplicated *FAD2* genes differed from each other by a large number of SNPs and several InDels. The analysis of the *FAD2* sequences of the four flax varieties allowed us to separate polymorphisms that were specific to a particular *FAD2* gene from those specific to a genotype. In most pairs of the duplicated *FAD2* genes (*FAD2b-1*/*FAD2b-2*, *FAD2c-1*/*FAD2c-2*, *FAD2d-1*/*FAD2d-2*, *FAD2e-1*/*FAD2e-2*, *FAD2f-1*/*FAD2f-2*, *FAD2g-1*/*FAD2g-2*), we revealed a significant number of SNPs that were present in all studied flax varieties and allowed distinguishing between two genes from a pair. The *FAD2a-1*/*FAD2a-2* pair was an exception, where *FAD2a-1* differed from *FAD2a-2* by only seven SNPs.

### 2.3. Identification and Phylogenetic Analysis of FAD3 Genes in Genomes of Different Flax Varieties

Using the sequences of the *FAD3a*, *FAD3b*, *FAD3c-1*, *FAD3c-2*, *FAD3d-1*, and *FAD3d-2* flax genes described by You et al. [46], we identified the *FAD3* genes in the genomes of the flax varieties YY5, 3896, Atlant, and CDC Bethune. Gene coordinates are presented in Table 3.

**Figure 2:**
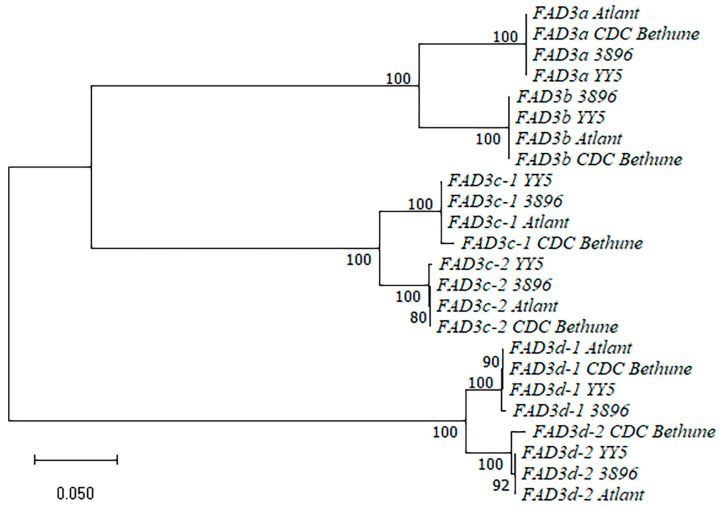
Phylogenetic analysis of *FAD3* genes of flax varieties YY5, 3896, Atlant, and CDC Bethune.

We analyzed the sequences of the *FAD3a*, *FAD3b*, *FAD3c-1*, *FAD3c-2*, *FAD3d-1*, and *FAD3d-2* genes of the four flax varieties (sequences are presented in Supplementary Table S1). A dendrogram for the *FAD3* gene sequences is presented in Figure 2. The sequences of the same *FAD3* genes of different flax varieties clustered with each other and formed subclusters. Subclusters of the duplicated genes grouped together: *FAD3a* and *FAD3b*, *FAD3c-1* and *FAD3c-2*, *FAD3d-1* and *FAD3d-2*. Pairs of the duplicated *FAD3* genes differed from each other by a significant number of SNPs and InDels. For each pair of the duplicated *FAD3* genes (*FAD3a*/*FAD3b*, *FAD3c-1*/*FAD3c-2*, and *FAD3d-1*/*FAD3d-2*), we revealed a significant number of SNPs and several short InDels that were present in all studied flax varieties and allowed distinguishing between two genes from a pair. We observed the similarity between the sequences of the same *FAD3* genes of the studied genotypes: few SNPs/InDels were revealed for *FAD3c-2*, *FAD3d-1*, *FAD3a*, and *FAD3b*. The *FAD3d-2* gene had no polymorphisms in the YY5, 3896, or Atlant genomes. However, this gene of CDC Bethune had about a 240-bp deletion compared to *FAD3d-2* of the other three varieties. The sequence before the deletion corresponded to *FAD3d-1* of YY5, 3896, and Atlant, while the sequence after the deletion corresponded to *FAD3d-2* of these varieties. We suggested that this was rather an error in the CDC Bethune genome assembly than a genotype feature. A similar picture was observed for *FAD3c-1*—one region of several dozens of nucleotides in length contained SNPs and InDels and was present only in *FAD3c-1* of CDC Bethune. In the same region of *FAD3c-1* in the three long-read flax genome assemblies, there was a slight difference in the length of homopolymers. However, this could be an assembly error. Similar to the analysis of the *FAD2* genes, the analysis of the *FAD3* sequences of the four flax varieties allowed us to distinguish polymorphisms that were specific to a particular *FAD3* gene from those specific to a genotype.

### 2.4. Identification and Phylogenetic Analysis of SAD Genes in Genomes of Different Flax Varieties

Using the sequences of the *SAD2-1*, *SAD2-2*, *SAD3-1*, and *SAD3-2* flax genes described by You et al. [46], we identified the *SAD* genes in the genomes of flax varieties YY5, 3896, Atlant, and CDC Bethune. Gene coordinates are presented in Table 4.

**Figure 3:**
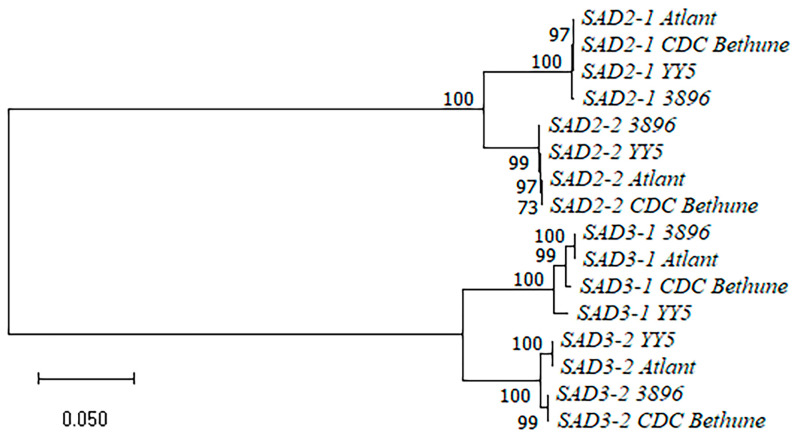
Phylogenetic analysis of *SAD* genes of flax varieties YY5, 3896, Atlant, and CDC Bethune.

We compared the *SAD2-1*, *SAD2-2*, *SAD3-1*, and *SAD3-2* gene sequences of the four flax varieties (sequences are presented in Supplementary Table S1). A dendrogram for the *SAD* gene sequences is presented in Figure 3. The sequences of the same *SAD* genes of different flax varieties clustered with each other and formed subclusters. *SAD2-1* and *SAD2-2* subclusters, as well as *SAD3-1* and *SAD3-2* subclusters, grouped together. The *SAD2-1*/*SAD2-2* pair differed from the *SAD3-1*/*SAD3-2* pair by a significant number of SNPs and InDels. For each pair of the duplicated *SAD* genes, we revealed a significant number of SNPs and several short InDels that were present in all the studied flax varieties and allowed distinguishing between two genes from a pair. The sequences of the same *SAD* genes were similar enough in the studied genotypes and mostly differed by single SNPs. However, a 20-30-nucleotide InDel and several 1-5-nucleotide InDels were identified in the *SAD3-1* gene. Two one-nucleotide InDels were revealed in the *SAD3-2* gene within the studied varieties. Thus, the analysis of the *SAD* sequences of the four flax varieties enabled distinguishing between polymorphisms that were specific to a particular *SAD* gene and those specific to a genotype.

### 2.5. Expression Analysis of FAD2 Genes

**Figure 4:**
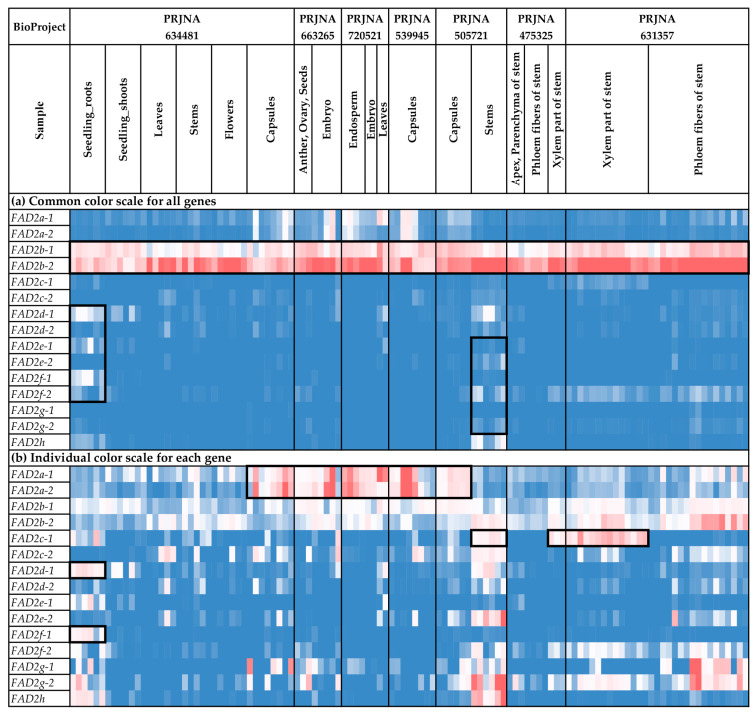
Expression of *FAD2* genes in different flax organs/tissues. (**a**) Common color scale for all genes. (**b**) Individual color scale for each gene. Blue (0.01 × Average)-White (Average)-Red (10 × Average) color scale is used. The expression data for organs/tissues and stress conditions were analyzed together (as in Supplementary Table S2), so the color scale in Figure 4 and Figure 5 is common. Noted gene expression patterns of interest are highlighted in boxes.

**Figure 5:**
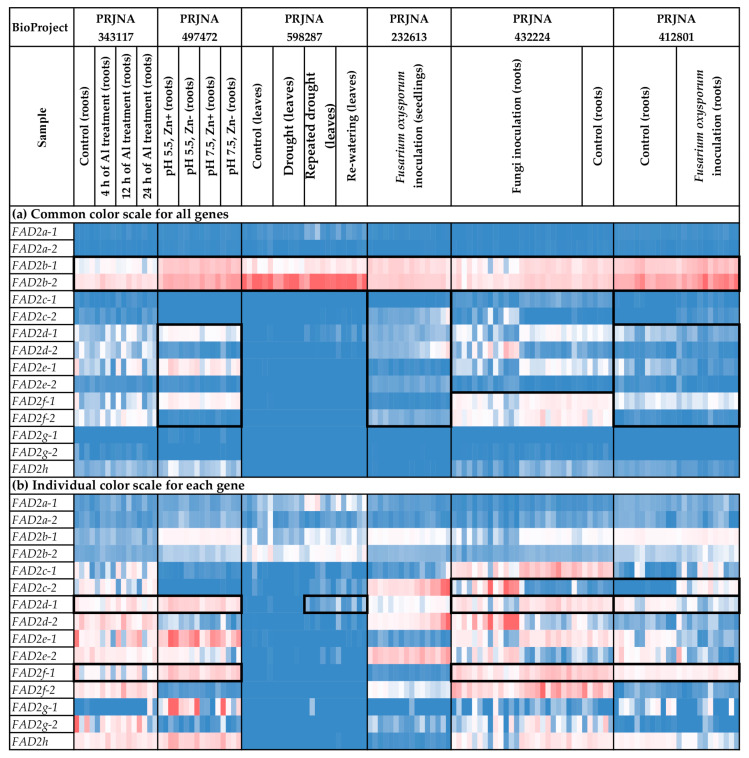
Expression of *FAD2* genes in flax plants under different stress conditions. (**a**) Common color scale for all genes. (**b**) Individual color scale for each gene. Blue (0.01 × Average)-White (Average)-Red (10 × Average) color scale is used. The expression data for organs/tissues and stress conditions were analyzed together (as in Supplementary Table S2), so the color scale in Figure 4 and Figure 5 is common. Noted gene expression patterns of interest are highlighted in boxes.

We analyzed the expression of the *FAD2* genes in different flax organs/tissues and under various biotic/abiotic stress conditions (Figure 4 and Figure 5, Supplementary Table S2). The highest expression levels were observed for *FAD2b-1* and *FAD2b-2* (Figure 4a). These genes were expressed at high levels in all the analyzed samples. For the other *FAD2* genes, expression was more organ-/tissue-specific. Notably, *FAD2a-1* and *FAD2a-2* were predominantly expressed in embryos, endosperm, seeds, and capsules, suggesting their significant role in the synthesis of flax oil FAs (Figure 4b). Increased expression of *FAD2a-1* was also observed in leaves in one of the used transcriptomic datasets (Figure 4b). Among the *FAD2* genes, *FAD2b-1* and *FAD2b-2* had the highest expression levels in embryos, endosperm, seeds, and capsules (Figure 4b). However, the expression of *FAD2b-1* and *FAD2b-2* was also high in other studied organs/tissues (Figure 4b). Therefore, these genes could play a key role in common processes in all flax organs and tissues, as well as the synthesis of flax oil FAs. Pronounced organ-/tissue-specific expression was observed for the *FAD2c-1*, *FAD2d-1*, and *FAD2f-1* genes. *FAD2c-1* had an increased expression level in the xylem part of stem, compared to phloem fibers, the apical part, and the parenchyma of stem (Figure 4b). Probably, this gene is involved in the functioning of stem xylem tissues. For *FAD2d-1* and *FAD2f-1*, high expression levels were specific to root samples (Figure 4b and Figure 5b). Therefore, these genes could have a significant role in root cells.

Along with organ-/tissue-specific expression, we discovered the effects of different stressors on *FAD2* expression in the analyzed flax samples. As such, *Fusarium oxysporum* inoculation increased *FAD2c-2* expression in roots (Figure 5b). This effect was most prominent in the data from the study of the early response to the fungus (NCBI BioProject, PRJNA412801). The other experimental data (PRJNA432224) demonstrated that the increased expression of *FAD2c-2* was more associated with *F. oxysporum* than other fungal species (Supplementary Table S2). For *FAD2d-1*, differential expression was detected in the samples from the study of drought influence on flax. The increased expression was characteristic of samples under repeated drought stress and re-watering conditions, compared to control and drought conditions (Figure 5b). However, no *FAD2* genes were activated in response to the other analyzed abiotic stresses (different soil pH, increased concentration of aluminum (Al), and zinc (Zn) deficiency) (Figure 5b).

We revealed regularities in the expression profiles for the pairs of duplicated *FAD2* genes. The profiles were quite similar between the *FAD2a-1* and *FAD2a-2* genes, as well as between *FAD2b-1* and *FAD2b-2* (Figure 5a). In contrast, the expression profiles were significantly different between the genes from a pair for the *FAD2c-1/FAD2c-2*, *FAD2d-1/FAD2d-2*, *FAD2e-1*/*FAD2e-2*, and *FAD2f-1/FAD2f-1* pairs (Figure 5a). However, the profiles of the *FAD2c-1*, *FAD2d-1*, *FAD2e-1*, and *FAD2f-1* genes located in a cluster were similar enough for a number of organs/tissues and conditions. The same effect was observed for *FAD2c-2*, *FAD2d-2*, *FAD2e-2*, and *FAD2f-2* located in another cluster (Figure 5a).

### 2.6. Expression Analysis of FAD3 Genes

**Figure 6:**
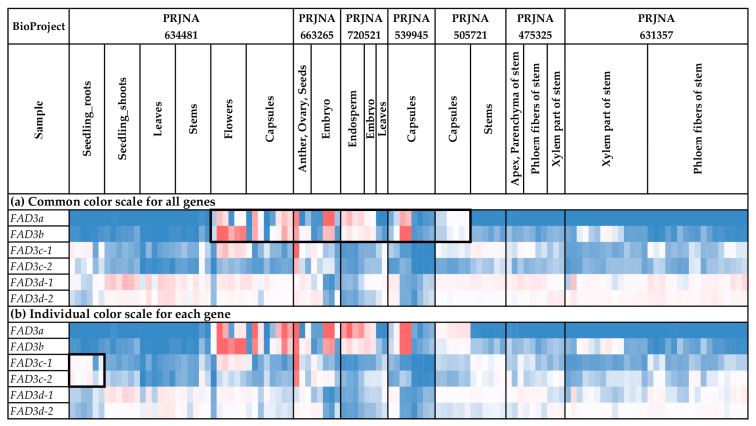
Expression of *FAD3* genes in different flax organs/tissues. (**a**) Common color scale for all genes. (**b**) Individual color scale for each gene. Blue (0.01 × Average)-White (Average)-Red (10 × Average) color scale is used. The expression data for organs/tissues and stress conditions were analyzed together (as in Supplementary Table S2), so the color scale in Figure 6 and Figure 7 is common. Noted gene expression patterns of interest are highlighted in boxes.

**Figure 7:**
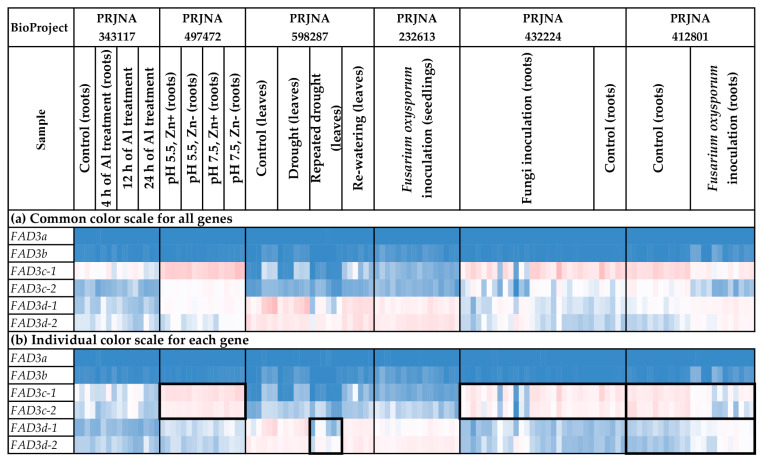
Expression of *FAD3* genes in flax plants under different stress conditions. (**a**) Common color scale for all genes. (**b**) Individual color scale for each gene. Blue (0.01 × Average)-White (Average)-Red (10 × Average) color scale is used. The expression data for organs/tissues and stress conditions were analyzed together (as in Supplementary Table S2), so the color scale in Figure 6 and Figure 7 is common. Noted gene expression patterns of interest are highlighted in boxes.

*FAD3* expression was analyzed for different flax organs/tissues and biotic/abiotic stress conditions (Figure 6 and Figure 7, Supplementary Table S2). *FAD3a* and *FAD3b* were expressed at high levels in flowers, capsules, and seeds (Figure 6a), with the most remarkable expression increase at the torpedo and cotyledon stages of embryo development (Supplementary Table S2). However, the expression was either low or zero in the majority of other tissues (Figure 6a). *FAD3c-1* and *FAD3c-2* had increased expression in most root tissues (PRJNA634481, PRJNA497472, PRJNA432224, PRJNA412801); however, they were also expressed in other organs/tissues (Figure 6b and Figure 7b). Meanwhile, decreased expression of *FAD3c-1* and *FAD3c-2* was observed for samples inoculated with *F. oxysporum* (PRJNA412801) (Figure 7b). Therefore, these genes could be involved in the response to the pathogen. In contrast to *FAD3c-1* and *FAD3c-2*, inoculation with *F. oxysporum* increased the expression of *FAD3d-1* and *FAD3d-2* (PRJNA412801) (Figure 7b). However, the expression of these genes decreased in flax samples under repeated drought conditions in the study of flax response to drought (Figure 7b).

### 2.7. Expression Analysis of SAD Genes

**Figure 8:**
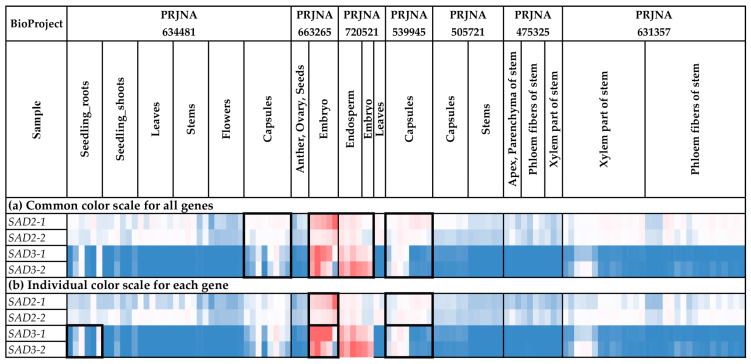
Expression of *SAD* genes in different flax organs/tissues. (**a**) Common color scale for all genes. (**b**) Individual color scale for each gene. Blue (0.01 × Average)-White (Average)-Red (10 × Average) color scale is used. The expression data for organs/tissues and stress conditions were analyzed together (as in Supplementary Table S2), so the color scale in Figure 8 and Figure 9 is common. Noted gene expression patterns of interest are highlighted in boxes.

**Figure 9:**
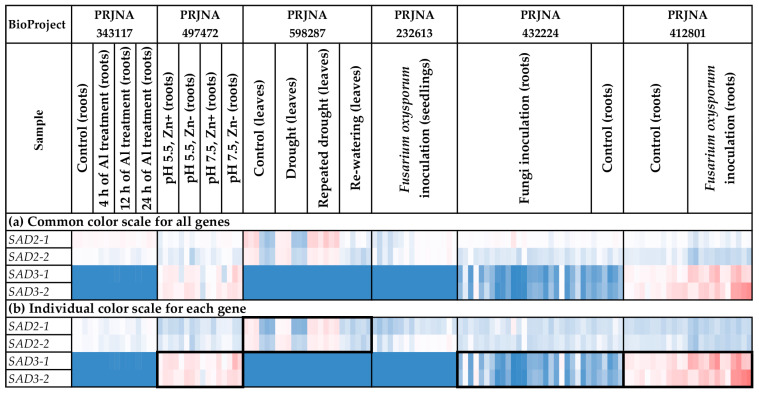
Expression of *SAD* genes in flax plants under different stress conditions. (**a**) Common color scale for all genes. (**b**) Individual color scale for each gene. Blue (0.01 × Average)-White (Average)-Red (10 × Average) color scale is used. The expression data for organs/tissues and stress conditions were analyzed together (as in Supplementary Table S2), so the color scale in Figure 8 and Figure 9 is common. Noted gene expression patterns of interest are highlighted in boxes.

We analyzed the expression of the *SAD* genes in different flax organs/tissues and under various biotic/abiotic stress conditions (Figure 8 and Figure 9, Supplementary Table S2). *SAD2-1* had expression profiles close to those of *SAD2-2*. Similarly, the *SAD3-1* expression profiles resembled those of *SAD3-2* (Figure 8 and Figure 9).

For all *SAD* genes (*SAD2-1*, *SAD2-2*, *SAD3-1*, and *SAD3-2*), increased expression was characteristic of embryos, endosperm, and capsules (Figure 8a). However, *SAD3-1* and *SAD3-2* had an increased expression level in capsules only at the early development stages (PRJNA539945) (Supplementary Table S2). For *SAD2-1* and *SAD2-2*, there were no significant differences between the various stages of capsule development. Notably, the difference between the expression levels of *SAD2* and *SAD3* was observed in the tissues of the developing flax embryo. While *SAD2-1* and *SAD2-2* had high expression levels in mature embryos, high *SAD3-1* and *SAD3-2* expression levels were characteristic of the early stages of embryo development (Supplementary Table S2). Therefore, *SAD3-1* and *SAD3-2* could be more important for the early stages of seed development. Meanwhile, *SAD2-1* and *SAD2-2* could also function at later stages of seed development.

*SAD2-1* and *SAD2-2* were expressed in most analyzed flax organs/tissues (Figure 8a). Unlike *SAD2-1* and *SAD2-2*, differences in the expression levels of *SAD3-1* and *SAD3-2* between organs/tissues were more pronounced (Figure 8a). In addition to embryos, endosperm, and capsules, *SAD3-1* and *SAD3-2* had increased expression levels in roots in most studied experiments (all samples of PRJNA497472 and PRJNA412801 and most samples of PRJNA432224 and PRJNA634481) (Figure 8b). In the majority of the other organs/tissues, *SAD3-1* and *SAD3-2* were unexpressed or expressed at very low levels (Figure 8b). Possibly, the *SAD3-1* and *SAD3-2* genes are involved in some processes in roots but not in common processes in all flax organs/tissues.

In addition, we revealed trends in the changes of *SAD* expression in flax under stress conditions. In the study of drought effects, flax genotypes under control and drought conditions significantly differed in *SAD2-1* and *SAD2-2* expression (Figure 9b, Supplementary Table S2). However, genotype differences almost vanished under repeated drought stress and re-watering conditions (Figure 9b, Supplementary Table S2). For *SAD3-1* and *SAD3-2*, increased expression was revealed in flax samples inoculated with *F. oxysporum* (Figure 9b).

## 3. Discussion

Genome assemblies of four flax genotypes allowed us to compare sequences of the *FAD2* (*FAD2a-1*/*FAD2a-2*, *FAD2b-1*/*FAD2b-2*, *FAD2c-1*/*FAD2c-2*, *FAD2d-1*/*FAD2d-2*, *FAD2e-1*/*FAD2e-2*, *FAD2f-1*/*FAD2f-2*, *FAD2g-1*/*FAD2g-2*, and *FAD2h*), *FAD3* (*FAD3a*/*FAD3b*, *FAD3c-1*/*FAD3c-2*, and *FAD3d-1*/*FAD3d-2*), and *SAD* (*SAD2-1*/*SAD2-2* and *SAD3-1*/*SAD3-2*) genes. We determined gene-specific polymorphisms, which are unrelated to a genotype but distinguish one gene from others. Our study demonstrated that even the latest CDC Bethune assembly contains errors in the *FAD2a-1* and *FAD2a-2* sequences—the same as in the previous assembly version (according to You et al.) [46]. Initially, the CDC Bethune genome was assembled from short Illumina reads [45]. Then, the assembly was improved to the chromosome level with optical mapping, BAC-clone libraries, and genetic maps [52]. However, the similarity between the *FAD2a-1* and *FAD2a-2* sequences (differing only by seven SNPs) could have led to errors in the assembly. The other three used genomes (YY5, 3896, and Atlant) were assembled with the use of long third-generation-sequencing reads [54,55,56]. Most likely, this allowed obtaining the correct *FAD2a-1* and *FAD2a-2* sequences. In the CDC Bethune genome, *FAD3c-1* and *FAD3d-2* were drastically different from the same genes in the three long-read flax assemblies. Likely, these sequences in the short-read assembly also had errors. The CDC Bethune assembly is often used as a reference in different molecular genetic studies of flax [61,63,75,76,77,78,79]. However, the errors in *FAD* sequences demonstrate that even a chromosome-level assembly can have inaccuracies in highly homologous regions. Therefore, the use of such sequences in experimental design and analysis can distort the results. Meanwhile, FADs are extensively studied because of their importance for oil crops.

*FAD2* genes play a critical role in the biosynthesis of FAs and the determination of OLE and LIO content in oilseed crops [80]. Manipulating the *FAD2* genes was used for the suppression of their expression and development of high-OLE plants, including canola, soybeans, *Camelina sativa*, rice, safflower, and peanut [81,82,83,84,85,86,87,88,89,90,91,92,93,94,95,96,97,98,99,100]. OLE is more resistant to oxidation than LIO and LIN. Therefore, a high content of OLE is preferable for premium-quality oil, and the development of oilseed crops with a high OLE content in their oil is desirable.

However, the effective application of genome editing and RNA interference (RNAi) for the suppression of gene expression requires knowledge of the number and sequences of *FAD2*. The correct choice of genome editing targets also needs information on the differences between the *FAD2* sequences and their expression levels in different tissues. In flax seeds/capsules, the genes of the OLE-LIO transformation are the most promising targets for creating flax varieties with high OLE content. Our analysis established that *FAD2a-1* and *FAD2a-2* were predominantly expressed in seeds/capsules, but *FAD2b-1* and *FAD2b-2* had higher expression in the same tissues. Moreover, *FAD2b-1* and *FAD2b-2* were expressed at high levels in all the studied flax organs/tissues. Another study showed that FAD2b had greater desaturase activity compared to that of FAD2a [48]. Thus, creating flax varieties with high OLE content can be challenging. Probably, all four *FAD* genes (*FAD2a-1*/*FAD2a-2* and *FAD2b-1*/*FAD2b-2*) should be inactivated. Meanwhile, the expression of *FAD2b-1* and *FAD2b-2* in all the studied flax organs/tissues could point out their vital role in the synthesis of FAs not only in plant seeds, where Fas serve as energy reserves, but also in all other organs/tissues, taking part in plant metabolism. Therefore, *FAD2b-1* and *FAD2b-2* inactivation could lead to a reduced capacity for flax survival. For example, manipulating the *FAD2* genes could result in poor agronomic characteristics of the obtained high-OLE plants [1]. However, RNAi-mediated silencing of the *FAD2* genes in flax (the experiment was conducted to suppress *FAD2a* and *FAD2b* expression regardless of the gene duplicates) allowed obtaining high-OLE plants with a normal phenotype [33]. In light of such studies, further development of high-OLE flax varieties is promising.

You et al. [46] analyzed the expression of the duplicated *FAD* and *SAD* genes only in pairs (except for *FAD3a* and *FAD3b*, which were treated individually) and in a small number of tissues. We demonstrated that genes from each pair differed by a significant number of SNPs and InDels (except for the *FAD2a-1*/*FAD2a-2* pair, where only seven SNPs distinguished these genes). Due to these differences, we could analyze the expression of individual genes. Moreover, sequencing a great number of transcriptomes of different organs/tissues of flax plants (including those under biotic/abiotic stress conditions) enabled the analysis of *FAD* and *SAD* expression for a representative set of samples [59,60,61,62,63,64,65,66,67,68,69,70].

In this study, we revealed seed-/capsule-specific expression of *FAD2a-1*, *FAD2a-2*, *FAD3a*, *FAD3b*, *SAD3-1*, and *SAD3-2*. Therefore, these genes could substantially contribute to the determination of the FA composition of flax oil. Since oil composition determines the further application of flax seeds, it is one of the most important characteristics of linseed. *FAD2b-1*, *FAD2b-2*, *SAD2-1*, and *SAD2-2* had high expression in embryos/seeds/capsules, as well as other tissues. Hence, these genes could be responsible for the synthesis of FAs in seeds and other flax organs/tissues. Organ-/tissue-specific expression was observed for *FAD2c-1* (high expression in stem xylem), and *FAD2d-1*, *FAD2f-1*, *FAD3c-1*, and *FAD3c-2* (high expression in roots). *FAD2* plays an important role in the synthesis of PUFAs in non-photosynthetic tissues, including roots [101,102]. In flax roots, the *FAD2d-1* and *FAD2f-1* genes seemed to be especially important for FA synthesis. Thus, our study significantly improved the understanding of which *FAD* and *SAD* genes play key roles in fatty acid synthesis in various organs and tissues of flax.

We demonstrated that four *SAD* genes had the highest expression in embryos, endosperm, and capsules. *SAD2-1* and *SAD2-2* were also expressed in the majority of the analyzed organs/tissues but at a lower level than in capsules. The differences in *SAD3-1* and *SAD3-2* expression between various flax organs/tissues were more distinct, with extremely low/zero expression in certain samples. In most articles on flax, the authors analyzed the *SAD2-1* and *SAD2-2* genes [51,79,103,104] (according to the classification of You et al. [46]; called *SAD1* and *SAD2* in the earlier study [40]). However, *SAD3-1* and *SAD3-2* remained out of scope in their research. Nevertheless, poorly studied *SAD3* could significantly contribute to the synthesis of oil FAs because of the high expression of these genes in flax seeds. Thus, the *SAD3* genes deserve to be studied in detail.

*FAD* and *SAD* are known to be involved in the response to stressors [80,105,106,107]. We discovered the differential expression of *FAD* and *SAD* in flax plants in response to biotic and abiotic stress conditions. *FAD2c-2*, *FAD2d-1*, *FAD3c-1*, *FAD3c-2*, *FAD3d-1*, *FAD3d-2*, *SAD3-1*, and *SAD3-2* changed expression levels on inoculation with the *Fusarium* species, while the *FAD2d-1* and *FAD3d-1* expression changed in response to drought. Although the difference between expression in susceptible and resistant flax genotypes was insignificant, these genes could be targeted in research on the stress response of flax plants.

We observed similar expression profiles for duplicated genes from a pair in the *FAD3* and *SAD* families (*FAD3a*/*FAD3b*, *FAD3c-1*/*FAD3c-2*, *FAD3d-1*/*FAD3d-2*, *SAD2-1*/*SAD2-2*, and *SAD3-1*/*SAD3-2*). The same was revealed for *FAD2a-1*/*FAD2a-2* and *FAD2b-1*/*FAD2b-2*. Likely, the genes from a pair co-express and have analogous functions in flax. Previous research showed co-expression and additive effects in LIN synthesis for *FAD3a*/*FAD3b* [43,44,46]. Meanwhile, expression profiles were markedly different in the *FAD2c-1*/*FAD2c-2*, *FAD2d-1*/*FAD2d-2*, *FAD2e-1*/*FAD2e-2*, *FAD2f-1*/*FAD2f-2*, and *FAD2g-1*/*FAD2g-2* pairs. Probably, duplicated genes from these pairs have different functions in flax plants. However, expression profiles were similar for the clustered *FAD2* genes. *FAD2c-1*, *FAD2d-1*, *FAD2e-1*, and *FAD2f-1* formed the first cluster, and *FAD2c-2*, *FAD2d-2*, *FAD2e-2*, and *FAD2f-2* formed the second one. Coregulation of clustered plant genes was reviewed by Tohge and Fernie [108]. In flax, *FAD2* from the same cluster could have common regulatory mechanisms determining their expression profiles.

Thus, the role of certain *FAD2*, *FAD3*, and *SAD* genes in the development of key flax plant characteristics could be elucidated from the analysis of their expression in various organs/tissues, at different developmental stages, and under different growth conditions. For instance, such research could establish the contribution of *FAD2*, *FAD3*, and *SAD* to the FA composition of flax oil. The information collected in the present study is the basis for developing effective flax genome editing procedures, as well as marker-assisted and genomic selection. These technologies are essential for the creation of flax varieties with a determined oil composition and require a solid theoretical background. In addition, recent research into different plant species aimed at *FAD* and *SAD* identification [10,12,13,14,16,109,110,111]. Based on the analysis of gene sequences of several genotypes and further expression evaluation in a representative set of transcriptomic data, our approach can be effective in a broad range of studies on the *FAD* and *SAD* genes.

## 4. Materials and Methods

### 4.1. Phylogenetic Analysis

*FAD2*, *FAD3*, and *SAD* sequences (accessions: Lus10012007 + Lus10012008, Lus10029283 + Lus10029284, Lus10021051, Lus10004175, Lus10021050, Lus10004176, Lus10021049, Lus10004177, Lus10021048, Lus10004178, Lus10021047, Lus10004180, Lus10021046, Lus10004181, Lus10021045, Lus10038321, Lus10036184, Lus10040660, Lus10018245, Lus10027809, Lus10005039, Lus10027486, Lus10039241, Lus10018926, Lus10028627) were downloaded from the phytozome database (https://phytozome-next.jgi.doe.gov/, accessed on 1 August 2023) and blasted (default parameters) against four *L. usitatissimum* genomes: YY5 (https://zenodo.org/record/4872894#.Y0_723ZBxaQ, accessed on 1 August 2023), Atlant (NCBI GenBank, GCA_014858635.1), line 3896 (GCA_030674075.2), and CDC Bethune (GCA_000224295.2). The selected hits of the chosen identity (>90.0%) and length (>400 bp) were extracted into separate fasta files using a custom script employing bedtools (the bedtools getfasta tool with default parameters) [112]. As the genes of the same family shared a high degree of similarity, duplicated sequences were filtered out manually. Exon–intron junctions in *FAD2*, *FAD3*, and *SAD* genes were determined based on transcript sequences from the phytozome database listed above.

For the extracted sequences, phylogenetic analysis was carried out in MEGA X [113]. Multiple alignment of the extracted sequences was performed using the MUSCLE algorithm with default parameters. Then, phylogenetic trees were constructed using the maximum likelihood method (default parameters, 1000 bootstrap replicates).

### 4.2. Expression Analysis

Raw fastq reads were downloaded from NCBI SRA using SRA Toolkit 3.0.0 (NCBI BioProject, PRJNA475325, PRJNA631357, PRJNA720521, PRJNA663265, PRJNA634481, PRJNA505721, PRJNA539945, PRJNA598287, PRJNA232613, PRJNA497472, PRJNA432224, PRJNA412801, PRJNA343117). Data were obtained for the following tissues: seedling roots, seedling shoots, leaves, capsules, seeds, embryos, endosperm, stems, the xylem and phloem parts of a stem, the apical part and the parenchyma of a stem. In addition, data for flax tissues under diverse stresses were analyzed, including Al treatment, Zn deficiency, drought, and fungi inoculation. The complete list of BioProjects and samples examined is presented in Supplementary Table S2. Sequencing runs were prefetched (prefetch SRRXXXXXX). The fasterq-dump tool was used to extract fastq files (fasterq-dump SRRXXXXXX). The downloaded reads were checked for integrity using cleanFastq (https://github.com/davidvi/cleanFastq, accessed on 1 August 2023). Paired reads were merged into single files.

Expression analysis was conducted using PPline [114]. Fastq reads were aligned to the YY5 v2.0 genome, and CPM (counts per million) values were calculated for 100-bp intervals covering the *FAD2*, *FAD3*, and *SAD* genes. The following specific options were set: ‘-crop 75′ (trim reads by 75 bases from the end), ‘--coverage-profiles--report-mode--for-intervals tmm_cpm raw_counts’ (output raw counts and calculate CPM values, use TMM normalization for the samples), ‘--coverage-profiles--split-regions--for-intervals--max-chunk-length 100’ (perform analysis for ~100-bp intervals), ‘--star-outFilterMultimapNmax 2’ (or ‘--star-outFilterMultimapNmax 1’), ‘--star-outFilterMismatchNmax 0’, ‘--star-outFilterScoreMinOverLread 0.8’, ‘--star-outFilterMatchNminOverLread 0.8’, ‘--coverage-profiles--count-duplicated-reads--for-intervals yes’, ‘--create-coverage-profiles-for-bed-intervals yes’, ‘--create-coverage-profiles-for-bed-intervals yes’. The *FAD2a-1* and *FAD2a-2* sequences were very similar (only 7 SNPs distinguish them), so the results of expression analysis for these genes could be biased in some samples. Further analysis and heatmap construction were performed in Microsoft Excel. The gene expression level was calculated as an average CPM for 100-bp intervals covering a gene. Blue (0.01 × Average)-White (Average)-Red (10 × Average) color scale was used, where Average—average expression level of all studied genes (common color scale, Figure 4a, Figure 5a, Figure 6a, Figure 7a, Figure 8a and Figure 9a) or individual genes (individual color scale, Figure 4b, Figure 5b, Figure 6b, Figure 7b, Figure 8b and Figure 9b) in all analyzed samples.

## Figures and Tables

**Table 1:** Coordinates of *FAD2* genes in genomes of flax varieties YY5, 3896, Atlant, and CDC Bethune.

Gene	Chromosome/ Contig (Gene Orientation)	Start	End	Chromosome/ Contig (Gene Orientation)	Start	End
	**YY5**			**3896**		
*FAD2a-1*	1 (+)	5,021,855	5,022,991	tig00001017 (−)	272,765	273,901
*FAD2a-2*	7 (−)	1,996,580	1,997,716	tig00001158 (−)	1,528,587	1,529,723
*FAD2b-1*	14 (+)	5,313,949	5,315,097	tig00001080 (−)	5,062,638	5,061,490
*FAD2b-2*	12 (+)	17,531,094	17,532,242	tig00001150 (−)	3,193,326	3,194,474
*FAD2c-1*	14 (+)	5,303,922	5,305,064	tig00001080 (−)	5,070,909	5,072,051
*FAD2c-2*	12 (+)	17,523,668	17,524,786	tig00001150 (−)	3,200,716	3,201,834
*FAD2d-1*	14 (−)	5,300,901	5,302,025	tig00001080 (+)	5,073,948	5,075,072
*FAD2d-2*	12 (−)	17,520,741	17,521,865	tig00001150 (+)	3,203,685	3,204,809
*FAD2e-1*	14 (+)	5,295,123	5,295,638	tig00001080 (−)	5,080,334	5,080,849
*FAD2e-2*	12 (+)	17,514,741	17,516,583	tig00001150 (−)	3,208,965	3,210,807
*FAD2f-1*	14 (+)	5,289,454	5,290,599	tig00001080 (−)	5,085,380	5,086,525
*FAD2f-2*	12 (+)	17,507,420	17,508,565	tig00001150 (−)	3,216,527	3,217,668
*FAD2g-1*	14 (+)	5,286,568	5,287,692	tig00001080 (−)	5,088,286	5,089,410
*FAD2g-2*	12 (+)	17,504,525	17,505,670	tig00001150 (−)	3,219,754	3,220,899
*FAD2h*	14 (+)	5,282,536	5,283,645	tig00001080 (−)	5,092,333	5,093,442
	**Atlant**			**CDC Bethune**		
*FAD2a-1*	JACHUY010000564.1 (−)	75,232	76,368	Lu1 (+)	5,295,671	5,296,667
*FAD2a-2*	JACHUY010000609.1 (+)	130,868	132,004	Lu15 (−)	2,178,694	2,179,867
*FAD2b-1*	JACHUY010000090.1 (−)	265,579	266,727	Lu8 (+)	5,480,883	5,482,031
*FAD2b-2*	JACHUY010000099.1 (+)	607,247	608,395	Lu6 (+)	15,636,287	15,637,435
*FAD2c-1*	JACHUY010000090.1 (−)	275,608	276,750	Lu8 (+)	5,471,361	5,472,503
*FAD2c-2*	JACHUY010000099.1 (+)	599,887	601,005	Lu6 (+)	15,628,973	15,630,091
*FAD2d-1*	JACHUY010000090.1 (+)	278,647	279,771	Lu8 (−)	5,468,309	5,469,463
*FAD2d-2*	JACHUY010000099.1 (−)	596,912	598,036	Lu6 (−)	15,626,046	15,627,170
*FAD2e-1*	JACHUY010000090.1 (−)	285,034	285,549	Lu8 (+)	5,462,609	5,463,124
*FAD2e-2*	JACHUY010000099.1 (+)	590,896	592,741	Lu6 (+)	15,620,008	15,621,871
*FAD2f-1*	JACHUY010000090.1 (−)	290,078	291,223	Lu8 (+)	5,457,127	5,458,272
*FAD2f-2*	JACHUY010000099.1 (+)	583,320	584,465	Lu6 (+)	15,611,968	15,613,113
*FAD2g-1*	JACHUY010000090.1 (−)	292,985	294,109	Lu8 (+)	5,454,415	5,455,539
*FAD2g-2*	JACHUY010000099.1 (+)	580,426	581,571	Lu6 (+)	15,609,199	15,610,344
*FAD2h*	JACHUY010000090.1 (−)	297,032	298,141	Lu8 (+)	5,450,383	5,451,492
FAD2b-2*	JACHUY010001688.1 (−)	21,994	23,144			
FAD2c-1*	JACHUY010001688.1 (−)	32,029	33,175			

Note: * Additional *FAD2b-2* and *FAD2c-1* were revealed for cultivar Atlant, which could be a result of errors in the Atlant genome assembly. (+)—forward orientation, (−)—reverse orientation.

**Table 2:** Clusters of *FAD2* genes in genomes of flax varieties YY5, 3896, Atlant, and CDC Bethune: (1) from *FAD2b-1* to *FAD2h* and (2) from *FAD2b-2* to *FAD2g-2*.

Cluster	Gene	YY5		3896		Atlant		CDC Bethune	
		Start	End	Start	End	Start	End	Start	End
1	*FAD2b-1*	1	1149	1	1149	1	1149	1	1149
	*FAD2c-1*	10,034	11,176	9420	10,562	10,030	11,172	9529	10,671
	*FAD2d-1*	13,073	14,197	12,459	13,583	13,069	14,193	12,569	13,723
	*FAD2e-1*	19,460	19,975	18,845	19,360	19,456	19,971	18,908	19,423
	*FAD2f-1*	24,499	25,644	23,891	25,036	24,500	25,645	23,760	24,905
	*FAD2g-1*	27,406	28,530	26,797	27,921	27,407	28,531	26,493	27,617
	*FAD2h*	31,453	32,562	30,844	31,953	31,454	32,563	30,540	31,649
2	*FAD2b-2*	1	1149	1	1149	1	1149	1	1149
	*FAD2c-2*	7457	8575	7391	8509	7391	8509	7345	8463
	*FAD2d-2*	10,378	11,502	10,360	11,484	10,360	11,484	10,266	11,390
	*FAD2e-2*	15,660	17,502	15,640	17,482	15,655	17,500	15,565	17,428
	*FAD2f-2*	23,678	24,823	23,202	24,343	23,931	25,076	24,323	25,468
	*FAD2g-2*	26,573	27,718	26,429	27,574	26,825	27,970	27,092	28,237

**Table 3:** Coordinates of *FAD3* genes in genomes of flax varieties YY5, 3896, Atlant, and CDC Bethune.

Gene	Chromosome/ Contig (Gene Orientation)	Start	End	Chromosome/ Contig (Gene Orientation)	Start	End
	**YY5**			**3896**		
*FAD3a*	13 (−)	19,562,809	19,566,016	tig00001077 (−)	4,099,093	4,102,300
*FAD3b*	4 (+)	1,112,215	1,115,203	tig00001161 (+)	1,111,942	1,114,930
*FAD3c-1*	9 (+)	6,171,137	6,173,194	tig00001132 (+)	6,016,888	6,018,941
*FAD3c-2*	4 (−)	4,763,983	4,766,021	tig00001161 (−)	4,738,958	4,740,996
*FAD3d-1*	1 (−)	4,338,899	4,341,004	tig00001017 (+)	958,035	960,140
*FAD3d-2*	13 (−)	8,289,964	8,292,051	tig00001016 (+)	5,664,077	5,666,164
	**Atlant**			**CDC Bethune**		
*FAD3a*	JACHUY010000211.1 (−)	243,193	246,400	Lu7 (−)	16,089,395	16,092,602
*FAD3b*	JACHUY010000300.1 (−)	181,559	184,547	Lu12 (+)	1,035,256	1,038,244
*FAD3c-1*	JACHUY010000050.1 (−)	42,666	44,714	Lu3 (+)	6,170,950	6,173,029
*FAD3c-2*	JACHUY010000317.1 (−)	123,347	125,385	Lu12 (−)	4,934,348	4,936,386
*FAD3d-1*	JACHUY010000337.1 (−)	185,049	187,154	Lu1 (−)	4,658,469	4,660,574
*FAD3d-2*	JACHUY010000034.1 (+)	397,509	399,596	Lu1 (−)	8,589,844	8,591,694

Note: (+)—forward orientation, (−)—reverse orientation.

**Table 4:** Coordinates of *SAD* genes in genomes of flax varieties YY5, 3896, Atlant, and CDC Bethune.

Gene	Chromosome/ Contig (Gene Orientation)	Start	End	Chromosome/ Contig (Gene Orientation)	Start	End
	**YY5**			**3896**		
*SAD2-1*	2 (+)	2,202,321	2,204,835	tig00001202 (+)	2,180,069	2,182,583
*SAD2-2*	3 (−)	21,506,947	21,509,465	tig00000059 (−)	5,662,980	5,665,498
*SAD3-1*	1 (+)	30,544,869	30,546,943	tig00001229 (+)	1,656,155	1,658,243
*SAD3-2*	6 (−)	1,060,842	1,062,735	tig00000142 (+)	241,674	243,567
	**Atlant**			**CDC Bethune**		
*SAD2-1*	JACHUY010000024.1 (−)	322,689	325,203	Lu10 (+)	2,257,483	2,259,997
*SAD2-2*	JACHUY010000302.1 (+)	70,480	72,998	Lu11 (−)	17,364,817	17,367,335
*SAD3-1*	JACHUY010000005.1 (−)	624,071	626,159	Lu1 (+)	28,379,153	28,381,248
*SAD3-2*	JACHUY010000319.1 (+)	241,463	243,356	Lu14 (+)	1,106,499	1,108,392

Note: (+)—forward orientation, (−)—reverse orientation.

## Data Availability

Publicly available datasets were analyzed in this study. Accession numbers can be found in the Section 4.

## Supplementary Materials

The following supporting information can be downloaded at: https://www.mdpi.com/article/10.3390/ijms241914885/s1.
