# Supplementary material for: Key FAD2, FAD3, and SAD Genes Involved in the Fatty Acid Synthesis in Flax Identified Based on Genomic and Transcriptomic Data
Source: Int J Mol Sci. 2023 Oct 4;24(19):14885. doi: 10.3390/ijms241914885 (PMC10573214; doi:10.3390/ijms241914885)
Supplement: Supplementary file 1 [file ijms-24-14885-s001.zip › Supplementary Table S1.pdf]

Supplementary Table S1. Sequences of *FAD2* (*FAD2a-1*/*FAD2a-2*, *FAD2b-1*/*FAD2b-2*, *FAD2c-1*/*FAD2c-2*, *FAD2d-1*/*FAD2d-2*, *FAD2e-1*/*FAD2e-2*, *FAD2f-1*/*FAD2f-2*, *FAD2g-1*/*FAD2g-2*, and *FAD2h*), *FAD3* (*FAD3a*/*FAD3b*, *FAD3c-1*/*FAD3c-2*, and *FAD3d-1*/*FAD3d-2*), and *SAD* (*SAD2-1*/*SAD2-2* and *SAD3-1*/*SAD3-2*) genes of flax varieties YY5, 3896, Atlant, and CDC Bethune.

**Gene**	**Sequence**
	**YY5**
FAD2a-1	ATGGGTGCCGGTGGCAGAATGTCAGTGCCTCCATCATCCAAACCTATGAAGAGGT<br>CTCCTTACTCAAAGCCACCATTCACGCTCGGTGAGCTCAAGAAGGCCATCCCTCC<br>ACACTGTTTCAAACGCTCAATCCCCCGATCGTTCGCCTACGTGGCGTACGACCTCA<br>CCATTGCAGCAATCTTCTACTACATCGCCACCACTTACTTCCACCTCCTCCCTAGC<br>CCTCTCAACTACCTCGCCTGGCCGGTCTACTGGGCCTGCCAGGGCTGCATCCTCAC<br>TGGAGTATGGGTGTTGGCTCACGAATGCGGTCACCATGCCTTCAGCGACTACCAG<br>TGGCTCGACGACATGGTTGGCTTCGTCCTCCATTCGTCCCTCCTTGTTCCTTACTTC<br>TCCTGGAAGCACAGCCACCGCCGCCACCATTCCAACACGGGATCGCTTGATCGTG<br>ATGAGGTGTTTGTCCCCAAGCAGAAGGCCGAAATCGGGTGGTACTCCAAGTACCT<br>TAACAACCCACCTGGCCGTGTGATCACATTGGCCGTCACATTAACGCTCGGTTGG<br>CCTCTGTACTTGGCATTCAACGTCTCCGGGAGACCATATGACCGGTTCGCATGCCA<br>TTTTGACCCTCACGGTCCGATTTACAATGATCGCGAGCGTATGGAGATATACCTAT<br>CCGACGCAGGGATATTCACCGTGTGCTACATCCTATACAGACTCGTCCTCACGAA<br>AGGACTCGTTTGGGTCGTGTCCATATACGGAGTCCCACTATTGATAGTGAATGGAT<br>TCCTAGTCCTCATCACTTTCTTGCAGCACACGCATCCTTCTCTTCCGCACTACAAGT<br>CCTCCGAATGGGACTGGATGCGAGGCGCCCTCTCGACCGTGGATCGAGACTACGG<br>GTTACTCAACACCGTGTTCCACAACATCACCGATACACATGTCGCGCACCATCTC<br>TTCTCCACGATGCCTCATTACCACGCGATGGAGGCTACCAAGGCGATCAAGCCGG<br>TTCTCGGGGAGTATTACCAGTTCGATGGGACTCCCTTTGTGAAGGCCATGTGGAG<br>GGAGGCAAAGGAGTGCATCTATGTCGAGCCGGATGAAGGCGACCCCAGCCAAGG<br>CGTGTTCTGGTACAACAACAAGCTGTGA
FAD2a-2	ATGGGTGCCGGTGGCAGAATGTCAGTGCCTCCATCATCCAAACCTATGAAGAGGT<br>CTCCTTACTCAAAGCCACCATTCACGCTCGGTGAGCTCAAGAAGGCCATTCCTCC<br>ACACTGTTTCAAACGTTCAATCCCCCGATCGTTCGCCTACGTGGCGTACGACCTCA<br>CCATTGCAGCAATCTTCTACTACATCGCCACCACTTACTTCCACCTCCTCCCTAGC<br>CCTCTCAACTACCTCGCCTGGCCGGTCTACTGGGCCTGCCAGGGCTGCATCCTCAC<br>TGGAGTATGGGTGTTGGCTCACGAATGCGGTCACCATGCCTTCAGCGACTACCAG<br>TGGCTCGACGACATGGTTGGCTTCGTCCTCCATTCGTCCCTCCTTGTTCCTTACTTC<br>TCCTGGAAGCACAGCCACCGCCGCCACCATTCCAACACGGGGTCGCTTGATCGTG<br>ATGAGGTGTTTGTCCCCAAGCAGAAGGCCGAAATCGGGTGGTACTCCAAGTACCT<br>TAACAACCCACCTGGCCGTGTGATCACATTGGCCGTCACATTAACGCTCGGTTGG<br>CCTCTGTACTTGGCATTCAACGTCTCCGGGAGACCATATGACCGGTTCGCATGCCA<br>TTTCGACCCTCACGGTCCGATTTACAATGATCGCGAGCGTATGGAGATATACCTAT<br>CCGACGCAGGGATATTCACCGTGTGCTACATCCTATACAGACTCGTCCTCACGAA<br>AGGACTCGTTTGGGTCGTGTCCATTTACGGAGTCCCACTATTGATAGTGAATGGAT<br>TCCTAGTCCTCATCACTTTCTTGCAGCACACGCATCCTTCTCTTCCGCACTACAAGT<br>CCTCCGAATGGGACTGGCTGCGAGGCGCCCTCTCGACCGTGGATCGAGACTACGG<br>GTTACTCAACACCGTGTTCCACAACATCACCGACACACATGTCGCGCACCATCTC<br>TTCTCCACGATGCCTCATTACCACGCGATGGAGGCTACCAAGGCGATCAAGCCGG<br>TTCTCGGGGAGTATTACCAGTTCGATGGGACTCCCTTTGTGAAGGCCATGTGGAG<br>GGAGGCAAAGGAGTGCATCTATGTCGAGCCGGATGAAGGCGACCCCAGCCAAGG<br>CGTGTTCTGGTACAACAACAAGCTGTGA
FAD2b-1	ATGGGTGCTGGCGGAAGAATGGCCGTGCCTCCATCGAACAAGGCGGACTCCGAA<br>ACCTTTAAGCGGTCTCCTTACTCAAAACCTCCCTTCACTCTTGGTGAGATCAAGAA<br>AGCCGTCCCTCCACACTGCTTCAAAAGGTCCATCCCCCGCTCGTTCTCCTACGTGG<br>CTTATGACCTCACCATAGCCGCCATCTTCTACTACATCGCCACCACTTACATCCAC

	CTCCTCCCCAATCCTCTCTCCTACGTGGCGTGGCCGATCTACTGGGCCTGCCAAGG<br>CTGCGTCCTCACTGGTGTCTGGGTCCTAGCCCACGAATGCGGTCACCATGCCTTCA<br>GCGACTACCAATGGCTCGACGACTTGGTCGGCTTTGTCCTCCACTCATGCCTCATG<br>GTACCCTACTTCTCGTGGAAGCACAGCCACCGTCGCCACCACTCCAATACTGGGT<br>CCCTCGAACGAGACGAGGTTTTTGTCCCCAAGCAGAAATCAGCCATTGGCTGGCA<br>CTCAAAGTACCTCAACAACCCACCTGGCCGTGTGCTCACACTTGCAGTCACTCTC<br>ACTCTCGGCTGGCCTTTGTACTTGGCATTCAACGTCTCTGGAAGGCCGTACGACCG<br>GTTCGCCTGCCATTACGATCCTAAATCCCCCATCTACAACGACCGCGAGCGAACG<br>GAGATATTCTTCTCCGATGCTGGCATCCTTGCTGTGAGCTTTGCGCTCTACAAGCTT<br>GCTGTCGCCAAGGGACTGGCTTGGGTGGTTTGTGTCTACGGAGTTCCACTCCTTGT<br>AGTGAATGGATTCCTTGTCTTGATCACTTTCTTGCAGCACACCCACCCATCATTGC<br>CGCACTACAAATCCTCCGAATGGGACTGGCTGAGAGGTGCTCTGGCGACCATGGA<br>CAGAGACTACGGGTTTCTGAACACGGTGTTCCATAACATCACGGATACCCACGTG<br>GCGCACCACCTGTTCTCGACGATGCCTCATTACCATGCAATGGAAGCTACAAAGG<br>CGATCAAGCCGGTATTGGGAGAGTACTACCAATTCGACGGACTCCATTCATCAA<br>GGCGATGTGGAGGGAGGCTAAGGAGTGTGTTTATGTCGAGCCCGACGAAGGTGA<br>CCAGAACAAAGGCGTGTTCTGGTACAACAACAAGCTGTGA
FAD2b-2	ATGGGTGCTGGTGGAAGAATGGCTGTGCCTCCATCGAACAAGGCGGACTCGGAA<br>ACCTTTAAGCGGTCTCCATACTCAAAACCTCCATTCACACTTGGTGAGATCAAGA<br>AAGCCATCCCTCCACATTGCTTCAAAAGGTCCATCCCCCGCTCATTCTCCTATGTG<br>GCTTATGACCTCACCATTGCCGCCATCTTCTACTACATCGCCACCACTTACATCCA<br>CCTCCTCCCCAATCCTCTCTCCTACGTTGCGTGGCCTATCTACTGGGCCTGCCAAG<br>GCTGCGTCCTCACCGGTGTCTGGGTCCTAGCCCACGAATGCGGTCACCACGCCTTC<br>AGCGACTACCAATGGCTTGATGACTTGGTCGGCTTTGTCCTTCACTCATGCCTCAT<br>GGTCCCCTACTTCTCGTGGAAGCACAGCCACCGTCGCCACCACTCCAACACCGGG<br>TCCCTTGAACGAGATGAGGTTTTTGTCCCCAAGCAGAAATCGGCCATCGGCTGGC<br>ACTCAAAGTACCTCAACAATCCACCTGGCCGTGTGCTCACACTTGCAGTCACTCT<br>CACTCTCGGCTGGCCTTTATACTTGGCATTCAACGTTTCCGGAAGGCCGTACGACC<br>GGTTCGCCTGCCATTACGATCCTAAATCCCCTATCTACAACGACCGCGAGCGAAC<br>TGAGATCTTCCTCTCCGATGCCGGCATCCTTGCTGTGAGCTTTGCAATCTACAAGC<br>TTGCTGTCGCCAAGGGACTGGCTTGGGTGGTTTGTGTCTATGGAGTTCCACTCCTT<br>GTAGTGAATGGATTTCTTGTCTTAATCACTTTCTTGCAGCACACCCACCCATCATT<br>GCCGCACTACAAATCCTCCGAATGGGACTGGCTGAGAGGTGCTCTGGCGACCATG<br>GACAGAGACTACGGGTTTCTGAACACGGTGTTCCATAACATCACGGATACCCACG<br>TGGCACATCACCTGTTCTCGACGATGCCTCATTACCATGCGATGGAAGCTACCAA<br>GGCGATCAAGCCGGTACTGGGAGAGTACTACCAGTTCGATGGACTCCATTCATC<br>AAGGCGATGTGGAGGGAGGCTAAGGAGTGTGTCTATGTTGAGCCTGACGAAGGT<br>GACCAGAACAAAGGCGTGTTCTGGTACAACAACAAGCTGTGA
FAD2c-1	ATGTTCTCATCCTCTGCAACTAAAATGCCGGCGCCGCCTTCATCATCCAACACGA<br>CCATGAAGCGATCGCCTCACTCAAAGCCGCCGTTCACCGTCTCCGACGTCAAGAA<br>AGCCATTCCACCACACTGCTTCCAAAGGTCCCTCCTCCGATCATTCTCCTACCTAA<br>CCTACGACCTCACCATCATCACAATCCTCTACCAAGTCGCCACCACTTACTTCCAC<br>CTCCTCCCGACACCTCTCTCATCCTACGTGGCATGGCCGGCCTACTGGGCCGGCCA<br>AGGCTGCTTCTTCGTCGCTGTATGGATGGTGGCCCACGAGTGTGGCCATCACGCCT<br>TCAGCGACCAGCACTGGCTGGAAGACTCGGTCGGATTCATCCTCCACTCAGCCCT<br>CCTTTCACCTTACTTCTCATGGAAGCACAGCCACCGTCGCCACCATGCCAACACG<br>AGCTCCCTCGAACGTGACGAGGTGTTCGTCCCTAAACCCAAGTCCAAGCTGAGCT<br>GGCACTTCAAGTTCTTCAACAACCCGCCTGGCCGCGTCCTGCAGTTGGCATTTGCT<br>CTGCTTCTTGGATGGCCTCTCTATTTGGCGATCAACATCGCCGGAAGGCCGTACGA<br>GAAATTCGCTAGCCACTTCGATCCGAGAAGCCCTATCTACAACGATCGAGAGCGG<br>ATCGAGATATTCGCATCCGATGTTGGCGTGCTCTGCATGTGGTTCGCGCTGTATAA<br>ACTTGCTCTAGTGAACGGAGTTGGTTGGGTTGTTTGTGTGTACGGGATCCCGCTGT<br>TGGTGATGAATGGATGGGTCGTTACGATCACTTACTTGCATCACACGCATATAGC

	GTTGCCGCGGTATGATTCGTCCGAATGGGATTGGTTGAGGGGTGCATTGGCGACT<br>GTGGATAGGGACTATGGTGTTTTGAACAAGGTGTTTCATAACATCACGGATACTC<br>ATGTGGCGCACCATCTGTTTTCGGCGATGCCTCATTACCATGCGGCGGAAGCGAC<br>CGAGGCGATCAAGCCCGTTTTGGGCGAGTATTATCGGTGTGACCGGACTCCTATT<br>ATCAAGGCGTTATGGAGGGAGTTTAAGCACTGTATTTATGTCGAGTCAGATGAGG<br>ATAAAGGCGTGTTTTGGTTCAATGACAAGTTGTAA
FAD2c-2	ATGCCGCCGCCGCCTTCATCATCCAACACAACCATGAAGCGATCGCCACACTCAA<br>AGCCGCCGTTCACCCTCTCTGACATCAAGAAAGCCATACCACCGCACTGCTTCCA<br>AAGGTCCCTCCTCCGATCATTTGCCTACTTAGCCTACGACCTCACCGCCGTCACAA<br>TCCTCTACCACATCGCCACCACGTACTTCCACCTCCTCCCGACACCTCTCTCATCC<br>TACGTGGCTTGGTCGGCCTACTGGGCCAGCCAAGGCTGCTTCTTCGTCGCTGTATG<br>GATGGTGGCCCACGAGTGCGGTCACCACGCCTTTAGCGACCAACACTGGCTGGA<br>AGACTCGGTAGGGTTCATCCTCCACTCGGCCCTCCTTTCGCCTTACTTCTCGTGGA<br>AGCACAGCCACCGCCGCCACCACGCCAACACGAGTTCCCTCGAGCGTGACGAGG<br>TGTTCGTCCCTAAGCTGAAATCCAAGCTGAGCTGGCACTTCAAGTTCTTCAATAAC<br>CCGCCTGGCCGCGTCCTGCAGCTGGCCTTTGCTCTGCTTCTAGGATGGCCTCTCTA<br>CCTTGCGGTCAACATCGCAGGAAGACCGTACGAGAAATTCGCTAGCCACTTCGAT<br>CCGCGAAGCCCTATCTATAACGATCGGGAGCGAATTGAGATATTTGTATCTGATG<br>TTGGCGTGCTTGCTATGTGGTTCGCTCTGTATAAACTTGCCCTGACGAACGGAGTT<br>GGTTGGGTTGTTTGTGTGTACGGGGTCCCTCTGTTGGTGATGAATGGATGGATCGT<br>TACGATCACTTACTTGCATCACACGCATGTCGCATTGCCGCGGTATGATTCCTCGG<br>AGTGGGATTGGTTGAGGGGTGCGCTCGCGACTGTAGATAGGGATTATGGGGTTTT<br>GAACAAGGTGTTTCACAACATTACAGATACTCATGTGGCGCACCATCTGTTTTCG<br>ACGATGCCTCATTACCATGCGGCGGAAGCAACCGAAGCGATCAAGCCCGTTTTGG<br>GTGAGTATTATCGGTCTGACCGGACTCCTATCGCCAAGGCATTATGGAGGGAGAC<br>TAAGCACTGTGTCTATGTCGAGGTTGATGAAGATAAAGGCGTGTTGTGGTTCAAT<br>GATAAGATATGA
FAD2d-1	ATGGTATCCAATACCACCATAAAGCGAACACCGACCTCAAAACCGCCGTTCACC<br>CTCTCCGACGTCAAGAAAGCAATACCACCGCACTGCTTCCAAAGATCCCTCCTCA<br>AATCCTTCACCTACCTAGCCTACGATCTCACCGTCATCACAATCCTCTACCACATC<br>GCCACGTCATACTTCCACCTCCTCCCCAACCCTCTCTCCTACGTGGCGTGGCCGCT<br>CTACTGGGCCGCCCAGGGGTCACACTTCATCGCCGTCTGGGTCATCGCCCACGAG<br>TGCGGCCACCACGCCTTCAGCGACTACCAGTGGCTAGACGACGCCGTTGGGTTCG<br>TCCTCCACTCCCTCCTCTTAGCTCCTTACTTCTCCTGGAAGCACAGCCACCGCCGT<br>CACCACGCCAACGCCGCCTCCATCGAGCGCGACGAGAATTACATCCCGAAAAAG<br>AAGGACGAAGTCAACTGGCATTTTAAATACCTCGACAACCCTCCCGGACACGTGT<br>TCTATATATTCTTCACTCTCACCCTCGGATGGCCGCTCTACTTGCTGGTCAACATCT<br>CCGGCAGGAAATACGACGACGGATTCGCCAGCCATTTGTATCCTTTCTCCCCTATC<br>TACAACGACCGCGAGAGATTCGGGATAGTCCTCTCCGTCGCCGGGATGCTCGCCA<br>CGTGGTTCGGTCTTTATAAACTCGCGATGGTCAACGGGTTCGGTTGGGTTGTTTGC<br>GTGTACGGGGTGCCTCTCATTCTCCAGAATGCGATGTTGATTACCATCACTTACTT<br>GCACCACACGCATCTCAACTTGCCCCACTACGACTCGTCGGAGTGGGACTGGATG<br>AGGGGTGCGCTCGCGACTGTGGATAGGGATTACGGAATTTTGAATAAGGTGATGC<br>ATAATATTACGGATACCCACGTGGCGCACCATCTGTTCTCGATGATTCCTCATTAT<br>CATGCGATGGAGGCTACAAATGCGATAAAGCCGGTTCTTGGGGAGTACTACCAA<br>GTTGATACTACGCCGTTTTTGAAGGCGTTGTGGAGAGAGACTAAGGATTGTGTTTA<br>TGTTGAGGCGGATGATGAGGGATCTGATCGGGAGAAGAAAGGAGGGGTGTTTTG<br>GTTCAAAACCAAGCTGTAA
FAD2d-2	ATGGTATCCAATACCACCCTAAAGCGAACACCGACCACAAAACCGCCGTTCACC<br>CTCTCCGACCTCAAGAAAGCAATACCACCACACTGCTTCCAAAGATCCCTCCTCA<br>AATCCTTCTCCTACCTAGCCTACGACCTCACCGTCATCACAATCCTCTACCACATC<br>GCCACGTCATACTTCCACCTCCTTCCCAAACCTCTCTCCTACGTGGCCTGGGTGGC<br>CTATTGGGCCGCTCAGGGGTCCCACTTCATCGCCGTGTGGGTCATCGCCCACGAG

	TGCGGCCACCACGCCTTCAGCGACTACCAGTGGCTAGACGACGCCGTCGGCTTCC<br>TTCTCCACTCCCTCCTCTTAGCTCCTTACTTCTCCTGGAAGCACAGCCACCGCCGC<br>CACCACGCCAACGCCGCCTCCATCGAACGTGACGAGAATTACATCCCGAGAAAG<br>AAGGACGAAGTCAACTGGCATTTTAAGTACCTTGATAACCCTCCCGGACACGTGT<br>TCTATATATTCTTCACTCTCACCCTCGGATGGCCGCTCTACTTGCTGGTCAACATCT<br>CCGGGAGGAAATACGACGACGGATTCGCCAGCCATTTGTATCCTTTCTCCCCGAT<br>CTACAACGATCGGGAGAGGTTCGGGGTGTTCCTCTCCGTCGCGGGGATGCTCGCC<br>ACGTGGTTCGGACTTTATAAGCTCGCAATGGTCAACGGTCTCGGCTGGGTTGTTTG<br>CGTGTACGGGGTCCCTCTGATTCTCCAGAATGCGATGTTGATTACCATCACTTACT<br>TGCACCACACGCATCTCAACCTGCCCCACTACGACTCGTCGGAGTGGGACTGGAT<br>GCGGGGTGCGCTCGCGACTGTGGATAGGGACTACGGGATTTTGAACAAGGTAAT<br>GCATAATATTACGGATACTCACGTGGCACACCATCTGTTTTCGATGATTCCTCATT<br>ACCATGCGATGGAGGCTACGAATGCGATCAGGCCGGTTCTGGGGGAGTACTACC<br>AGGCCGATACGACGCCGTTTATTAAGGCGTTGTGGAGGGAGACTAAGGACTGTGT<br>TTATGTTGAGGCGGATGATGAGGGCGGTGATCAGGAGAAGAAAGGAGGGGTGTT<br>CTGGTTCAAAACCAAACTGTGA
FAD2e-1	ATGTCCCCTATTTACAGCGACCGCGAGCGAGCCGAGGTATTCGCCTCAGATGTTG<br>GCTTGCTCGCTGTCTGCTTCGCGTTGTACAAACTTATTATGGTCAAGGGAATGGCG<br>TGGGTTTTTTGCGTCTATGGGGCTCCGGTCATGGTGGTGAATGGATTCTTCATTACC<br>ATCACTTACTTGCATCACACTCATCTCGCGGTCCCGCGATACGATTCGTCTGAATG<br>GGATTGGTTGAGGGGAGCCCTGGCAACCATGGACAGGGACTTTGGCCTTTTGAAC<br>AAGGTGTTCCATAATGTTACAGATACTCACGTGACGCACCATCTGATTTCGACGA<br>TCCCTCATTATCATGCCATGGAAGCCAACAACGCAATTAGGCCCGTGTTGGGGGA<br>CTACTACCATATCGACAGGACGCCGGTGGTTAAGGCGTTGTGGAGGGAGGCTAA<br>GGAGTGTGTTTACATCGAGGCCGATGATGGTGAAAAGAACAAAGGCGTGTTCTG<br>GTTCAATACCAAGCTCTAA
FAD2e-2 (exon-intron junctions are not given)	ATGCACTTTCCGATAGCTAACCTTTGTCTGATGCTAAAAACAGGCCATACATGGC<br>GCCACAAAGCACTGCTCTAGTAAGTACACATGATTGGGATTACACTACACTTGAG<br>TAATTAAATCAGTCCATATTTTACCTGGATCCAAAGTTGAAAACTAACTCGAAAA<br>CAAATTTGTGCAAAATTGAGCATCAAATGAGACAATATTAAACTATTTCGAGTTTT<br>GTTGGCGCTGTTCTCGAGTTGTATTAGCCTCTCCGAAAACATGTATAAAAAGCACT<br>CGACCTATTCATATTATACAACATCAAAATCCCACCAATCAATCAAAAAACAGA<br>GAGACACATCTAAAAGCCCCCCCCCCCCCCCCCCTTACAAAAATGGTGGAGC<br>GACGTTCATCCAACAAGGCGGCGGCGGAAACCGAAACCGCCGTCGTCAAAC<br>GGTTCCCGTCCTCAAAGCCGCCGTTCACACTCGCCGACCTCAAGAAGGCCATTCC<br>GCCGCACTGTTTCAAAAGATCCATCCCACGCTCCTTCTCCTACTTGGTTTTCGACCT<br>CATCGTCGCCGCCGTCTTCTACCACATCGCCGCCACTTATTTCCCCCTCATCCCCA<br>AACCTCTCTCCTACGTGGCGTGGCCGGCCTACTGGTTCGTCCAGGGCAGCGTCCTG<br>ACCGGTGTGTGGGTCATCGGCCACGAGTGCGGCCACCACGCATTTAGCGAGCACC<br>AGTGGCTCGACGACCTGGTCGGGTTCATCCTCCACTCGGCCCTCCTCACCCCTTAC<br>TTCTCGTGGAAGATCAGCCACCGCCGCCACCACGCCAACACCTGCTCCCTCGAGC<br>GCGACGAGGTATATACTGTTGGGAACCGGACAAGAAGCAATATTCAGGGTTTATC<br>GATCTGGCTGAGTATACATGGACAAACTATCCATCAACATCCAAGATAAAAACA<br>GAGCTATAACTTTCAACTTCCGTGTGCAAAAATAGGGAGATGAATAAAATTTCAG<br>ATAATTTAAACAAATAAATTATCTCCAAAATGAATAATATTGGTCTCAAGTCCAA<br>TCCAACCTGGAGAACTGGATCTTTTTACAAAGACGTTTTTGCATTAGTCTTTTTTTC<br>TTTCTCCGATGTGATATTTGAGACTTAATTATTCACATTTTTCAACAGGTGTACATT<br>CCGAGGAAGAAGTCTCAGTTGCGGTGGTGGTACTCGAGCTACCTGAACAACCCGC<br>CCGGCAGACTTTTGGCGCTGGCTTACACCATCCTACTCGGCTGGCCTTCCTACTTG<br>ACTTTCAACCTATCCGGAAGGGAATACAACGGTTTGGCCTGTCATTTCTACCCGAT<br>GTCCCCTATTTACAGCGACCGGGAGCGGGCCGAGGTATTCGCCTCCGATGTTGGC<br>TTGCTTGCTGTGTGCTTCGCGCTGTACAAACTTATCCTCGTCAAGGGAATGGCGTG<br>GGTCTTTTGCGTCTACGGGGCTCCGGTCATGGTGGTGAACGGATTCTTCATAACCA

	TCACGTACTTGCACCACACTCATCTTGCGGTCCCGCGGTACAATTCCTCGGAATGG<br>GATTGGTTGAGGGGAGCCCTAGCAACGATGGATAGAGACTTTGGGTTATTGAACA<br>GGGTGTTCCATAACGTTACAGATACTCATGTGACGCATCATCTGATCTCGACCATC<br>CCTCACTACCATGCCATGGAAGCCAACAACGCGATTAGGCCCGTGTTGGGGGACT<br>ACTACCACATCGACAGGACGCCTGTTGTTAAGGCGTTGTGGAGGGAGGCTAAGC<br>AGTGTGTTTACATCGAGGCGGACGAAGGTGAAAATAACAACGGCGTGTTCTGGTT<br>CAATACCAAGCTCTAA
FAD2f-1	ATGGTGTCCTCCGGCAAAACCATGAGCAACAAAACCACCACGAAGCGGCCGCCG<br>GTGTCAAAACCGCCGTTCACTCTGGCCGACATCAAGAGAGCCGTACCGCCACACT<br>GCTTCAAAAGATCCCTCGTCAAATCATTCGCCTACCTAGCCTACGACCTCACCGTC<br>ATCACAATCCTCTACCACATCGCCAACACATACTTCCACCTCCTCCCCAAACCTCT<br>CTCCTACGTGGCGTGGCCCGTCTATTGGGCTGCCCAATGCTGCTTTTTCGTCGCTTT<br>ATGGATGGTGGGCCACGACTGCGGCCATCATTCATTCAGCGACTATCAGTGGGTG<br>GACGACACCGTTGGATTCGTCGTCCACTCGTTCCTCCTCGCCCCTTACTTCTCGTGG<br>AAGCATAGCCACCGCCGCCACCACGCCAACTCCGGATCTCTAGAACGCGACGAG<br>TCGTTCGTCCCGAAAACCAAAGACAATATCACCTGGCACTTCAAGTACCTCGACC<br>ACCTACCGGGGCGGATCTTCTACGTGGTCTTCACTCTAACCCTCGGATGGCCGCTC<br>TACTTGATGTTCAACATCACCGGAAGGCCGTACAAGGACGGATTCGCCAGTCATT<br>TCTACCCCATGTCGCCTATCTACGAGGATCACGAGCGGTTCGGAATATTCCTCTCT<br>GATGTTGGCATGCTCGCTATGTGGTTCACACTCTACAAGCTTTCCGTGGCGTATGG<br>AGTCGGTTGGGTTCTTTGTGTGTATTTTATTCCGCTGGTTTTACAAAATGCGTTGTTT<br>GTCACGATCACTTACTTACACCACACGCATCTGAACTTGCCTCACTATGACTCTTC<br>GGGGTGGGACTGGATGAGGGGGTCGTTGACGACTGTGGATAGGGACTATGGGTTT<br>TTGAACAAGGTGTTGCATAACGTGACAGATACTCACGTGGCGCACCATCTGTTCA<br>CCCATATGCCTCATTACCACCAATCGGAAGCGACCAAGGCGTTCATTCCGGTTTTG<br>GGGGAATACTACCAGGTTGATCCGACTCCGTTCTACAAGGCGTTGTGGAGGGAGA<br>TGAAGCACTGTGTTTATATCGAGCAAGATGAGGATGCGGATTCTGATAATAACAA<br>AAAAGGCGTGTACTGGTACAAAACCAAGCTCTAA
FAD2f-2	ATGGTATCCTCCGACAAAACCACGAACAACAAAACCACCACGAAGCGGCCGCCG<br>CTGTCAAAACCGCCGTTCACCCTCGCCGACATCAAGAGAGCCGTACCGCCGCACT<br>GCTTCAAAAGATCTCTAGTCAAATCATTCGCCTACCTAGCCTACGACCTCACCGTC<br>ATCACCTTCCTCTACCACATCGCCAACACATACTTCCATCTCCTCCTCAAACCTCT<br>CTCCTACGTGGCGTGGCCCCTCTACTGGGCCGCTCAATGTTGCTTCTTCGTCGCTTT<br>ATGGATGGTGGGTCATGATTGTGGCCACCATTTGTTCAGCGACTATCAGTGGATTG<br>ACGACACTGTCGGTTTCATCATCCACTCGTTCCTCCTTGCTCCTTATTTCTCCTGGA<br>AGCACAGCCACCGCCGCCATCATGCCAACTCCGGATCTCTAGAACGCAACGAGT<br>CATTCGTCCCGAAAACCAAAGACAATATCGCCTGGCACTTCAAGTACCTCGACCA<br>CCTACCGGGGCGGATCTTCTACGTCGTTTTCACTCTGACCCTCGGGTGGCCACTAT<br>ACTTGATGTTCCACATCACTGGAAGGCCGTACAAGGATGGATTCGCCAGCCATTT<br>CTACCCCATGTCGCCGATCTATGAGGATCACGAGCGGTTCGGGATATTCCTCTCCG<br>ACGTTGGAATGCTCGCCATGTGGTTCACGCTCTACAAGCTCTCAATGGCGTATGG<br>AGTCGGTTGGGTTCTTTGTGTGTATTTTATTCCGTTGGTTTTACAAAATGCTTTGTTT<br>GTTACGATTACTTACTTGCACCACACGCATTTGAACTTACCTCACTATGACTCTTC<br>GGGGTGGGACTGGATGAGGGGGTCGTTGACGACTGTGGATAGAGACTATTCGTTT<br>TTGAACAAGGTGTTGCATAACGTGACAGATACTCACGTGGCGCACCATTTGTTCA<br>CACATATGCCTCATTACCATCAATCGGAAGCGACCAAGGCGTTCATTCCGGTTTTG<br>GGGGAGTACTACCAGGCTGATCCGACGCCGTTCTACAAGGCGTTGTGGAGGGAG<br>ATAAAGCATTGTGTTTATGTTGAACAAGATGAGGATGCGGATTCCGATCAGAACA<br>AAAGAGGAGTGTACTGGTACAAAACCAAGCTGTGA
FAD2g-1	ATGAACAACAGAACCACCACGAAGCTGCTGCCGCTGTCAAAACCGCCGTTCACT<br>CTGGCCGACATCAAGAGAGCCGTACCGCCACACTGTTTCAAAAGATCCCTCGTCA<br>AATCATTCGCCTACCTAGCCTACGACATCACCGTCATCACAATCCTCTACCACATC<br>GCCAACACATACTTCTACCTCCTCCCCAAACCTCTCTCCTACGTGGCGTGGCCCGT

	CTATTGGGCCGCCCAAAGCTGCTTCTTCGTCGCTGTATGGATGGTGGGTCACGATT<br>GCGGCCACCATTCATTCAGCGACTACCAGTGGGTGGACGACACTGTCGGATTCGT<br>CGTCCATTCGTTCCTCCTCACCCCTTACTTCTCGTGGAAGCACACCCACCGCAGCC<br>ACCACGCCAACAACGGATCTCTAGAACGCGACGAATCTTTCGTCCCCAAGACCA<br>AAGACGAAGTCAGGTGGCACTTCAAGTACCTCGACCACCTACCGGGGCGAATCTT<br>CTACGTGTTCTTTACTCTAACCCTAGGATGGCCTCTCTACTTGATGTTCAACATCAC<br>CGGAAGGCCGTACAAGGATGGATTCGCTAGCCATTTCTACCCCATGTCGCCTATG<br>TACGAGGACCATGAGCGGTTTGGAGTAGTCCTGTCCGACATGGGAATGCTCGCTA<br>TGTGGTTTACGCTCTACAAGCTCTCGGTGGCGTTCGGAGTCACTTGGGTTCTCTGT<br>GTGTACTTTATTCCACTAGTTTTACAAAACGCGTTGTTTGTTACGATCACTTACTTG<br>CACCACACGCATCCAAACGTGCCTCGTTATGACTCTTCGGGGTGGGGTTGGATGA<br>GGGGGTCGTTGGTGACCGTGGATAGAGACTATGGGTTTTTGAATAAAGTGTTCCA<br>TAACGTGACGGATACTCACGTGGCGCACCATCTGTTCACGCATATGCCTCATTACC<br>ACCAATTGGAAGCTACCAAGGCGTTCATCCCGATTTTGGGAGAGTACTACCAGGC<br>TGATCCAACACCGTTCTATAAAGCATTGTGGAGAGAAATGAAGCACTGTGTTTAT<br>GTCGAACAAGACAAAGATGCAAATGTCGATCAGAACAAAAGAGGTGTGTATTGG<br>TACAAAACAAAGTCGTGA
FAD2g-2	ATGGTATCCTCCGACAAAACCATGAACAACAAAACCACCACGAAGCTGCTGCCG<br>CTGTCAAAACCGCCGTTCACTCTCGCCGACATCAAGAGAGCCGTACCGCCACACT<br>GCTTCAAAAGATCCCTCGTCAAATCATTCGCCTACCTAGCCTACGACATCACAGT<br>CATCACAATCCTCTACCACATCGCCAACACATACTTCCACCTCCTCCCCAAACCTC<br>TCTCCTACGTGGTGTGGCCCCTCTACTGGGCTGCCCAATGTTGCTTCTTCGTCGCGG<br>TATGGATGGTGGCCCATGACTGTGGCCACCATTCATTCAGCGACTATCAATGGGT<br>CGACGACACCGTCGGTTTCATCATCCACTCGTTCCTCCTCGCCCCTTACTTCTCATG<br>GAAGCACACCCACCGCAGCCACCACGCCAACAATGGATCTCTAGAACGCGACGA<br>GTCTTTCGTCCCCAAGACCAAAGACGAAATCAGGTGGCACTTCAAGTACCTCGAC<br>CACCTACCGGGGAGAATCTTTTACGTGTTCTTCACTCTAACCCTAGGATGGCCTCT<br>CTATTTGATGTTCAACATCACCGGAAGGCCGTACAAGGACGGATTCGCCAGCCAT<br>TTCTACCCCATGTCGCCTATGTACGAGGACCACGAGCGGTTCGGAGTAGTCCTCTC<br>TGACATGGGAATGCTCGCCATGTGGTTCACGCTCTACAAGCTCTCAATGGCGTAC<br>GGAGTCAGTTGGGTTCTCTGTGTGTACTTTATTCCACTGGTTTTACAAAATGCGTTG<br>TTTGTCACGATCACTTACTTGCACCACACGCATCCGAACTTGCCTCGTTATGACTC<br>TTCGGGGTGGGGTTGGATGAGGGGGTCGTTGGTAACTGTGGATAGAGACTACGGG<br>TTTTTGAACAAAGTGTTGCATAACGTGACAGATACTCACGTGGCGCACCATCTGTT<br>CACGCATATGCCTCATTACCACCAATTGGAAGCTACCAAGGCGTTCATTCCGGTTT<br>TGGGGGAGTACTACCAAGCTGATGCAACGCCGTTCTACAAGGCATTGTGGAGGG<br>AGATGAAACACTGTGTTTATGTTGAGCAAGATGAGGATGCAGATTCCGACCAAA<br>ACAAAAGAGGTGTGTACTGGTACAAAACCAAGCCGTGA
FAD2h	ATGGTATCCAACAAAACCATAAACCGGCCGCCGTCCTCAAAACCGCCGTTCATCC<br>TCTCCGACATCAAGAAAGCAATCCCACCACACTGCTTCCGCAAATCCCTCCTCCG<br>ATCATTCTCCTACGTAGCCTACGACCTCGCCGTCATCGCAATCCTCTACCACATCG<br>CCACGTCATACTTCCACCTCCTCCCCAAACCTCTCTCCTACGTGGCCTGGCCGGCA<br>TACTGGGCCGCCCAGGGATCCCACTTCATCGCCGTCTGGGTCCTGGCCCACGAGT<br>GCGGCCACCACGCCTTCAGCGACTACCAGTGGCTAGACGACGTCGTCGGATTCGT<br>CCTCCACTCCGCTCTCCTCTCGCCGTACTTCTCGTGGAAACACAGCCACCGCCGCC<br>ACCACTCCAACTCGGCATCTCTAGAACGCGACGAGCTGTACATCCCGAAGAAGA<br>AATCGGAGATTAGCTGGCATTACAAGTACCTCGACAACCCGCCGGGTCATCTTTT<br>CTACTTGGTGTTCACTCTCACTCTCGGATGGCCTCTCTACGTGATGTTCAACGTCTC<br>CGGCAGGGAATACGACGACGGATTCGCTAGCCATCTGTATCCTTTCTCCCCGATCT<br>ACAACGAACGGGAGAGATTCGGGATACTTTTGTCGGACGCTGGGATGCTTGCCAC<br>TTGGTTTGGTCTGTACAAGCTGTCCATGGTCAACGGACTGAGTTGGGTTGTTTGTG<br>TGTACGGGGTCCCGCTATTGGTGATGAATGGATTGCTTGTGACGATCACTTACTTG<br>CATCACACCCATCTTTCGCTCCCGCATTATGACTCGTCGGAGTGGGAGTGGATGA

	GAGGTGCGCTAGCGACTGTGGATAGAGACTACGGGTTCCCGCTGAATAAGGTGAT<br>GCATCATATTACGGATACTCACGTGGTGCACCATTTGTTCTCGATGATTCCTCATT<br>ACCATGCGACGGAGGCTACAAATGCGATTAGGCCGATTTGGGTGAATATTATCA<br>AGTTGATCCGACTCCGTTTGTCAAGGCGTTGTGGAGGGAGATGACGCACTGTGTTT<br>ACGTTGAGGCCGATGAGAAGAAACGAGGTGTGTTCTGGTATAAAACCAAGCTAT<br>GA
FAD3a	ATGAGCCCTCCAAACTCAATGAGTCCCGCCACCAACGGCAGCACCAATGGTGTG<br>GCTATCAATGGGGCGAAGAAGCTACTCGATTTCGACCCGAGTGCTGCTCCCCCTT<br>TCAAGATTGCAGACATCCGTGCTGCAATCCCGCCGCATTGTTGGGTGAAGAACCC<br>CTGGAGGTCACTCAGCTACGTCCTGAGAGACCTCCTGGTCATCCTCAGCTTCGCCG<br>TTGCGGCGACAAAGCTGGACAGCTGGACTGTCTGGCCTCTCTACTGGATTGCTCA<br>AGGAACCATGTTCTGGGCAGTCTTTGTTCTTGGACATGATTGGTAATTTCACATGA<br>TCTTTCTGGTAATGTGGGTTTTCTTTTCTTATTGAAAAAGATTAAAACTTTTTATCTG<br>GGCTGTTGCATGCAGTGGCCATGGGAGCTTCTCAGACAGTTGGTTGTTGAACAAC<br>GTGATGGGACATATACTCCATTCCTCAATCCTCGTACCTTACCATGGATGGTATTG<br>TAACTATTGTTCGATATTCGATTATGATTACTGTTCTTTCAGATGAAGAATCTGTAC<br>CCTAATTGTTTTTTGTTACCAGGAGAATTAGCCACAAGACCCATCACCAGAATCA<br>CGGCAATGTGGAGAAAGATGAATCCTGGGTTCCAGTAAGTTGACATGCAGTTTGC<br>TCTAAAATGCAGAGTCCTCTGTTTTTTGTGTGTTCTTGTGCTTTAATGACGATGATA<br>ATGAAATTGAAATTTGTAATAGCTGCCGGAGAAGGTGTACAAGAGCTTGGATACC<br>GGCACCAAGTTCATGAGGTTCACCATCCCTCTCCCAATGTTTGCGTATCCTATCTA<br>CTTGGTAAGTAAACAGACTGACTCCAAAGTAGGAACTAATGACAATTTTGGACCC<br>GACCTGGTTTGGTTGACTCGGGTCGATATGTTTCGGGTGGGTAATTACCCGATCTG<br>GCGATGGGTGTGCGGCGGACATTGTCTTGCTCGTGGTCCACCCCGCTCCCAACCC<br>GCCCCATTCTTGACGAAAAAGATTTCGGAATATGTATCAACAGAAAAATCTAGTT<br>TTTATGTTACTAGTTTTCTGTATTTCCATGTTTTTTCCTCAATTCTAGCCGGAATTTG<br>AATTCAAACTGAAATCGGGTAATTCCGTCCATAACAAAACGGAATTGGGCAGCC<br>GTAATTAGTTGAAACTAGACCTCAATTTTGGCGGAATTGGACCCGGCCATTTTTTA<br>CGTTTGCAAAACGGAAAACGTTTTTCTTTTGTAAAGCGCAAAATGAAAAACGTAT<br>CTAGTGGAATTATTGGACCCATCTAGAATGGGTCCAATTCCACCCCAATTTCGGCT<br>CCAATTCATGCCCGGAAAACACTACTGTCATGCATTTTAATCTTGTATGGTTTTAC<br>CCCAATGGATGCAGCGATGGATCCGGACGATTTTTAAAATATTATCGGGTTAAAT<br>TTAAAAATATCTTAAAACTATAAGAAAAAAATAACCAATTTTAAAGAATAAAAG<br>AACTGGACACATATGACGGGTGTCGTGGATGGATGTACTTGTCCCGCTCTATTAA<br>AGGCTGATAATATACAGGTCAACGGTGAATGAAGGTTAGATGCGCTATTGGATTT<br>GAATCCGATATGAAATGATAATTTTGGACACGATCTGTTTTGGGTGGGTAATATTT<br>GATCTAGGGATGGCTCGTGCTCCAAACCGCACCAAAACCGCCTAATTCTCGACCA<br>AAAAGATTTTATGAATACATATCAACAGAAAAATCTAGTTTTCATGTTACTAGTTT<br>TATGTACAACAATATTAGGTGTCGTTTTCCCAGCCTTTTTCTTCAATTCCGGCCGGA<br>ATTCGCATTCAAACCGGAATTGGATGGAATCGGTATACCTCGTCACGGATGCATT<br>GTCAATTCCTAGTTAGTTTCATGGTTTTGAAACCAATCAATCTATTCTATATGGTTT<br>TGATTAACAGTGGAGGAGAAGTCCGGGGAAGAAAGGGTCGCATTTCAACCCATA<br>CAGTGACCTGTTCGCACCGAACGAGAGGACATCGGTCATGATTTCGACATTGTGC<br>TGGACAGCCATGGCCTTACTCCTCTGCTACTCATCGTTCATCTACGGCTTCCTTCCG<br>GTCTTCAAAATCTACGGCGTCCCTTATCTAATATTCGTGGCGTGGCTCGACATGGT<br>GACCTACCTTCACCACCACGGGTACGAGCAGAAGCTGCCGTGGTACAGAGGCAA<br>AGAGTGGAGCTACCTACGTGGAGGGCTGACGACCGTCGATCGAGATTACGGGGT<br>CATCAACAACATCCACCATGACATTGGCACCCATGTTATTCACCATCTCTTCCCTC<br>AAATGCCACACTATCACCTAGTCGAAGCGGTAAGGAGGTCTTGATTATTAACTTA<br>ATGTTTTTGTTGTTATAATTTGAGTCCGATTCTGGAGTCAGGGGATTTCCTTCTTGG<br>ATCCGATCCAGGATCAAGCTGGTCCCTTGAATTTCTATATGATCTTATATTAATTA<br>AGGATAATGTGGTCATATGTTTTTAAATATTTTTGTTTACCATCATTTTCGATCACC<br>GGAAAATGTCCTGAGCAGTTTTCCGGTCACTTTAACCTCCATTGACAAATTTTTC

	ACCCACATGATCACCCTAGCCGGGTTTACGTTTATTGAAAATTTTTATTTTTTTGAA<br>TTTTTTTTCGATGACCAACTGTACAACTTTGTATTGAAAGTTGTATGGATCATACA<br>AATGTGTATGTACAAAAGTATATTCTAAGTACTATACTAAGCATTACTTAGTATTA<br>CGTTTCTACAAACCTATAGAGAAATGCATACAATTTTGTATAGAACTTAGTATAC<br>ACGTAGCTGTGAAATGTCAATTTCCCTCCGTATTTTCAGAGACAAGACATGATTTT<br>TAGACTGGCAGATTTTTTTTATCGGATAGATTTCTCCAACTTCAGATTCGGACTGG<br>ATTATTAACTATATTATTCATCAACTCTGACGTTTGATGTTGCATGTGACAGACTC<br>AGGCAGCGAAGCACGTGCTGGGGAAGTACTACAGAGAACCGAAGAAATCAGGG<br>CCTTTCCCATTCCACTTGTTTGGGTACTTGGTGAGGAGCCTGGGCGAGGATCACTA<br>CGTTAGCGATACAGGCGACGTCGTTTTCTATCAATCTGACCCACATATTCCCAAGT<br>TCCCTACCAGTGCCACCACCAAGTCCAAATCTAGCTGA
FAD3b	ATGAGCCCTCCAAACTCAATGAGTCCCACCACCAACGGCAATGGTGTGGCTATGA<br>ATGGGGCGAAGAAGCAGCTCGATTTCGACCCGAGTGCTGCCCCCCCTTTCAAGAT<br>TGCAGACATCCGTGCTGCAATTCCGCCGCATTGCTGGGTGAAGAACCCCTGGAGG<br>TCGCTCAGCTACGTCCTGAGAGACCTCCTTGTCATCCTCAGCTTCGCCGTTGCGGC<br>GGCAAAGCTGGACAGCTGGACTTTCTGGCCTCTTTACTGGGTTGCTCAAGGAACC<br>ATGTTCTGGGCAGTCTTTGTTCTTGGACATGATTGGTAAACTAATTTCACATTTTCT<br>TTCTGGTAATGTGGGTTTTATTGAAAAAGATTAAAACTTTTTATCTGGGTTGTTGCA<br>TGCAGTGGCCATGGGAGCTTCTCAGACATCTGGTTGTTGAACAATGTGATGGGAC<br>ATATACTCCATTCCTCAATCCTCGTACCTTACCATGGATGGTATTGTAACTATTGTT<br>CAATATTAGATTATTGCTAGTTCTTCAGCTGAAGAATCCAAACCCTAATTTTCTTTT<br>TCTGAATATTGACCAGGAGAATTAGCCACAAGACCCATCACCAGAATCACGGCA<br>ATGTGGAGAAAGATGAATCCTGGGTTCCTGTAAGTTGACATGCAGTTTGCTGTAA<br>AAATGCAGAGTGCTCTGTTTTTTTGTGTTCTTGTGCTTTAATGGTGATAATAATGAA<br>ATTGTTGAAATGTAACAGCTACCGGAGAAAGTGTACAAGAGCTTGGATACCAGC<br>ACTAAGTTCATGAGGTTCACCATTCCTCTCCCAATGTTTGCTTATCCTATCTACTTG<br>GTAAGTAAAGAGACTGATAAGACTCCAAAGTAGGAATTAATGACAATTTTGGAC<br>CCGAGCTTCCGACTCGGGTCGATTTATTTCGGGTGGGTACTTACCCGATGCGGCGG<br>ACATTGTTTTGCTCGTGGTCCACCTCGCTCCCAACCCGCCCCATTCTTGACGAAAA<br>AGATTACGGAATATGTATCAACAGAAATATCTAGTTTTTATGTTACTAGTTTTCTGT<br>ATTTCCGTGTTTTTCCCCTCAATTCCGTCAGAAATTTGAATTCAAACTGACATTGGG<br>TAATTCTGTCCATAAGAGAACGGAATTGGGAAGCCGTAATTAGTTGGAATTAGAC<br>CTCGATTTCGGCGGAATTGGACCCGGCCATTTTTCGCGTCCGGAAAAGCGTTTCCA<br>GTGGAGTTAGACGCCCATCTAGAATGGGTCCAATTCCACCCCAATTTCGGGGCTA<br>CCTATTTTTAGTGTACATGCATTTTAGATCTTTTACGGTCTTACCCCGATGGATATT<br>CTTGTCGCGCTCTATTAAAAACTGACAATATACAGGTTGATTGTGAATGAAGATT<br>AGGTGCTGCACGCTCTTCGTGTTCCAACCCGCACCCAAACCGCCCCATTCTCGAC<br>CGAAAAGATTTTATGAATACATATCTACAGAAAAATCTAGTTGTCATGTCACTAG<br>TTTAATGTACAACAGTATAGGTGTCGTTTTCCGCGTCTTTTTCTTCAATTCCGGCTG<br>GAATTCGCATTCAAACAGGAATTGGATGGAATTGGTCCCGGATGCATAGTCATTT<br>CCCAGGCAGTTTCATGGTTTTATAACCAATCAATCTAATCTTATGCTTTTGATAAA<br>CAGTGGACGAGAAGTCCGGGGAAGAAAGGGTCGCATTTCAACCCATACAGCGAC<br>CTATTCGCACCAAACGAGAGGGCAGCGGTCTTGATTTCAACATTGTGCTGGACAG<br>CCATGGCCTTACTCCTCTGCTACTCATCGTTCATATACGGCTTCGCTCCGGTCCTCA<br>AAATCTACGGCGTACCTTATCTGATATTCGTGGCATGGCTCGACATGGTGACCTAC<br>CTTCATCACCACGGGTACGAGCAGAAGCTGCCGTGGTACAGAGGCAAAGAATGG<br>AGCTACCTACGTGGAGGGCTGACGACCGTTGATCGAGATTACGGGGTCATCAACA<br>ACATCCACCATGACATTGGCACCCATGTCATTCACCATCTCTTCCCTCAAATGCCA<br>CACTATCACCTTGTGGAAGCGGTAAACAATTTGATTATTAATTTACTGTTTTTGTTG<br>TTATAATTTGAGTCGGGAGATTTCCTTCCTAAATCCGATCCCTGGTCAATCTTGGC<br>CCTTGAATCTTCATATAATCTAAAAATCTAGATTAATCAGGAACAATATGATCAT<br>GTTGTTTAAACTAATTTTGTTGGACCATAACCTACCGCCAACTGATGGACCACCGT<br>CTCTGGTTACCGGACCCATCATTTCCGGTTACCAAGAAGTTTCTCGATCAGTTTTC

	CGGTTACTTTGACCTGCGTTGAGGAAAATTCTTTCACCCACGTAAACACTGTCGTC<br>AACTTTACGTTTCTGGAAAGTTTTTCCGATGATTGGCCGTACAATTTTGTACGAAG<br>AGTTGTACGGATCATATAAATGTGTATAAGTTTCTAGAAATCCGTACTGAAATAT<br>ATACATATTTGACTTTTGTATAAAGTGTAATACTAAATACTATACTAAGTGCTGTA<br>CTCAGTATGATACTTAGTACACACATTTGTATGACTATGAAATGTCAATTTTGCCC<br>TTATATTCTCAGCCGTTAGATCTAAGACACAGTTTTTATACGGCTGAAATTTGTGG<br>GGGCTTTGTAGATCGGATCCATAAGTCATTTCTTGGCTCAAGATTCGGACTCGATT<br>ATTAACTATATTATTCATCAACTCTGACGTTTGATGTTGCATGTGACAGACTCAGG<br>CAGCGAAGCACGTGCTGGGGAAGTACTACAGAGAGCCGAAGAAATCAGGGCCTT<br>TCCCATTCCACTTGTTTGGGTACTTGGTAAGGAGCCTGGGCGAGGATCACTACGTT<br>AGCGACACAGGCGACGTCGTTTTCTATCAGTCTGACCCACATATTCCCAAGTTCCG<br>TACCAGCAGTGCCACCACCAAGTCCAAATCCAGCTGA
FAD3c-1	ATGAGCCCTCCAAACTCCATGGGAATGGAAGCTGCTCATCCTACCGGCAACGGC<br>AATGGCGTCGCAGTCATGAACGGCGCCTCCGCTAACAAACCCGATTTCGATCCCA<br>GCGAGGCTCCTCCCTTCAAGATTGCCGACATCCGAGCCGCCATCCCCCCGCATTG<br>CTGGGTGAAGAATCCATGGAGGTCTCTTAGCTACGTCCTTAGGGACGCCGTCGTC<br>ATTCTCGCATTCGCAGCTGCCGCCCTCAAGCTCGACCTCTGGGCTGTTTGGCCGCT<br>CTACTGGATCGCTCAGGGCACCATGTTTTGGGCTGTCTTCGTTCTCGGCCACGATT<br>GGTAAGAGGAGGAGGTTTTTTTTAAAATTTCAATCCGATGCTGTTTTGTTATATTA<br>TTGGTTGATATGATATATAAGATTAACCCTAGAGAAAAGTGAAAAGCATCCGCCA<br>TTGTTGAATGAATTGGAATTGGATGGTGCAGCGGCCATGGGAGTTTCTCGGATAG<br>CTGGTGGCTGAACAATGTGGTGGGGCATATTCTGCATTCTGCAATCCTTGTGCCTT<br>ACCATGGATGGTAAGAAAAGAATATGAACAGATCCTTTATCCTTACAACTGTAAA<br>ATGTAGGTCATTCAGCTGAACTAAAGTCACATTCTGTGTTGTTTAATTGATTGAAC<br>AGGAGAATTAGCCACAAAACACACCACCAGAATCATGGCAATGTTGAGAAAGAT<br>GAATCATGGGTTCCGGTGAGTTTTATTTCATTTTTTCTCTCGTCAATTGGCATCAGT<br>TTGTTCTGTTTCAATGCTTGATATGAATTGGGTGATATAATGCAGCTGCCGGAGAA<br>AGTATACAAGACCCTGGACACAAGCACCAAATTCATGAGGTTCACTATCCCTCTC<br>CCAATGTTTGCTTATCCTATCTACTTGGTAAGAACCACCCCACATTCTTACATCCTT<br>GCATGTTGTGTATTGGTTTCTGGTTGGATTTGGATTCAGATTTGGGGAATTGATTGT<br>GTGCCTTTGAAATGAATGAATGGTTACAGTGGACGAGGAGCCCAGGGAAGAAAG<br>GGTCCCATTTCAACCCCTACAGTGACCTGTTTGCCCCACAAGAAAGGAAATCAGT<br>CTTAATCTCTACCATCTCTTGGATTTCCATGGTCCTAATCCTCCTCTACGCCTCCTT<br>CCTTTTTGGTTTCCTCACTGTCTTCAAAGTCTATACCGTCCCTTACCTGGTAAAACT<br>ACTATCTCCAAATTCAACAATCTGTAAGCTTCATTTCATTTGCTTTGGCTCCTGAGT<br>TCCCTGTCAAGTTTCTCCTAGCAGTGTAACTTGTTATTTGATAACTTTGTGCCATCT<br>CACTATTGGCTGCATTTTACTATAATCAAACTGCTACATACGTTATTGCAACTTGTT<br>AATCCTTGGTACGAGGAATCCAATGAATAGGATGGTGATTAGCACAAACAAGGC<br>TGAAACTTTGAGCAATTTTGCAGATATTTGTGGCGTGGCTGGACATGGTGACATAC<br>CTGCACCACCACGGGCACGAAGAGAAGCTGCCGTGGTACAGAGGTCAAGAGTGG<br>AGCTACCTACGTGGAGGGCTGACAACCGTAGATAGAGATTACGGATTATCAAC<br>AACATCCACCACGACATTGGCACTCACGTGATTCACCATCTGTTCCCTCAAATCCC<br>TCACTACCATCTCGTAGAAGCGGTACAGTAGTAGTAAACAAAACCCCATATTATT<br>ATTACTTACTGCAGTGATTTCTGACTCTATATTATTAAATATCACTTCTGGGTTTTT<br>GGCCGCGGGGGGGGGGGGGGGGGGGGGTTGTTTGGATCTTTCGAACTTATAAT<br>AATATGATATTATTATTAAAATGGTGGCAGACAAAGGCAGCAAAGTCGGTGCTTG<br>GGAAGTACTACAGAGAGCCAAAGAAATCAGGACCATTCCCATTCCACTTGTTCGA<br>CAACTTAGTGAGAAGCCTTGGCGAAGATCACTATGTTAGTGATGCAGGGGATGTC<br>GTGTTCTATCAGTCTGACCCAGAAATCTTCAAGTTTTCCAAGTCCAAGTCAGCCTA<br>A
FAD3c-2	ATGGGAATGGAAGCTGCTCATCCTACCGGCAACGGCAATGGCGTCGCCGTCATGA<br>ATGGCTCCGCCGCTAACAAACCCGATTTCGATCCCAGCGAGGCTCCTCCGTTCAA<br>GATTGCCGACATCCGAGCCGCCATCCCTCCGCATTGCTGGGTGAAGAATCCATGG

	AGGTCACTCAGTTACGTCCTTAGGGACGCCGTTGTCATTCTCGCATTCGCCGCTGC<br>CGCACTCAAGCTCGACCTCTGGGCTGTTTGGCCGCTCTACTGGATCGCTCAGGGC<br>ACCATGTTTTGGGCTGTCTTCGTTCTCGGCCACGATTGGTAAGAGGAGGAGTTGTT<br>TTTTTTAAATATTTATCCGATGCTGTTTTGTTATATTATTGGTTGATATGTAAGATTA<br>ACCCTAGAAAAAAGTGGAAAACGTCCGCCATTGTTGAATTGAATTGGAATTGGAT<br>GGTGCAGCGGCCATGGGAGTTTCTCGGATAGCTGGTTGCTGAACAATGTGGTGGG<br>GCATATCCTGCATTCTGCAATCCTTGTACCTTACCATGGATGGTAAGAAAAGAAT<br>ATGAACAGATCCTTTATCCTTGAAACGGTAAAATTTAGGTTATTCAGCTGAAACA<br>AAAGTGACATCCTGTGTTGTTCTTTATTGATTGAACAGGAGAATTAGTCACAAAA<br>CTCATCACCAGAATCATGGCAATGTTGAGAAAGATGAATCATGGGTTCCGGTGGG<br>TTTTATTTCTCTTCCTTACTTGTCAATTTGCCATCATTTGTCTGTTTCACTGATTGAC<br>ATGAACTGGGTGGGAAAATGCAGCTGCCAGAGAAAGTGTACAAGACTCTGGACA<br>CAAGCACAAAATTCATGAGGTTCACTATCCCTCTCCCAATGTTTGCTTATCCTATC<br>TACTTGGTAAGAACCACCCTACATTCTCACATCCTTGCATGTTGTGTATTGGTTTCT<br>GGTTGGATTTGGATTCAGATTTGGGAAATTGATTGTGTGCATATGAAATGAATGA<br>ATGGTTACAGTGGACAAGAAGCCCAGGGAAGAAAGGGTCCCATTTCAATCCCTA<br>CAGTGACCTGTTTGCCCCACAGGAAAGGAAATCAGTCTTAATCTCTACCATCTCTT<br>GGATTTCCATGGTCCTAATCCTCCTCTACGCCTCCTTCCTATTTGGCTTCCTCACAG<br>TCTTCAAAGTCTACACCGTCCCTTACCTGGTAAAAACTCCACCCTTGGTTTACTAG<br>CTTCAAATTCAACAATCTATCAGCTTCATTTCATTTGCTTTGGCTCCTAAGTTCCCT<br>CTTAAGTTTCTCGTAGCAGAGTAACTTGTCTATTTGTTAACTTTGTGCCATCTCCCT<br>ATTGGGTGCATTTCACTATAATCAAACTGCTACATGCATACTGGAATGTCCATATT<br>GTAATTTGCTATAATCCTTGGTACGAGAATCCAATGAATAGGATGGTGATTAGAA<br>CAAGAAAGACTGAAACTTTGAGCAAATTTTGCAGATATTTGTGGCGTGGCTGGAC<br>ATGGTGACATACCTGCACCACCACGGGCACGAAGAGAAGCTGCCGTGGTACAGA<br>GGCAAAGAGTGGAGCTACCTACGTGGAGGGCTGACAACCGTAGATAGAGATTAC<br>GGGATTATCAACAACATCCACCACGACATTGGCACTCACGTGATTCACCACCTCT<br>TCCCTCAAATCCCTCACTACCATCTCGTAGAAGCGGTACTCTAGTAAACAAAACC<br>CATATTATTATTACTTACTGCAGTGATTTCTGACTATGTTATTATTAAATATCAGTT<br>CTGGGTTTTGGCCTTTGGGGGGGTTGGTTTGGATCTTTGGCACTTATAATATTATTA<br>AAATGGTGGCAGACAAAGGCAGCAAAGTCGGTGCTTGGGAAGTACTACAGAGA<br>GCCAAAGAAATCAGGGCCATTCCCATTCCACTTGTTCGACAACTTAGTGAGAAGC<br>CTTGGCGAAGATCACTATGTTAGTGATGCAGGGGATGTCGTGTTCTATCAGTCTGA<br>CCCAGACATCTTCAAGTTTTCCAAGTCCAAGTCAGCCTAA
FAD3d-1	ATGGCGAGCTGGGTTCTATCAGAATGCGGCATAAAGCCACTCCCTTCCGCCTTCC<br>CTAAGCCCAGAACCGGAGCGCTTTCCCGAAATACCCTCCCCAAGCTCAGGTATTT<br>ACCCCCGAGGAGCAATCTCTCCGCCGCTGCCGACGGCGGCGCCTCAGATCTAAGC<br>CTTAACCTACCTAAATTATCCTCCGTAGAGAAGAAAAAGCCGCTTTTGGGACAGG<br>TGAGAGTTACTGCTCCGTTCAAGGTCGCTCCGGTGAAGGAGGAAGACGGAGAAG<br>GAGAGTCCAATTTCGACCCAGGAGCGCCGCCGCCGTTCAATTTGGCCGACATTCG<br>AGCTGCTATACCCAAGCATTGCTGGGTTAAAGATCCATGGAGGTCAATGGCTTAC<br>GTGGCGAGGGACGTGGCCGTTGTTCTCGGATTGGCAGCTGCCGCAGCTTATCTGA<br>ACAATTGGATCGTTTGGCCGCTGTATTGGGCGGCTCAGGGGACCATGTTCTGGGCT<br>CTCTTTGTTCTTGGCCATGACTGGTGAGTAAAGTTGTTAACTTTCTCTTTGGTCTTTT<br>ATGCTAATCTTTGTAATAATGGAAAATTGAAGAAAAATTCTCATGTTTGGCAGTG<br>GACATGGGAGCTTTTCAAGCAATCACAAGCTGAACAGTGTAGTTGGACACATTCT<br>CCATTCTTCAATTCTTGTGCCTTACCATGGATGGTAACTAAAGCTCTCTGCTTTCCT<br>CTGCCCATCTGTCATTTTGTTCTTCAATTTTGAAGCAATTTGAAACAAATTTTGTAT<br>CAATGTCTTTACAGGAGAATTAGCCATAGGACGCATCATCAGAACCATGGACATG<br>TTGAGAACGATGAGTCATGGCATCCTGTGAGTCTTTGTTTCTAAATCTTATCTATA<br>GGATTGATTCAGATGCTGATGTACTAATTTCTGTGACTTTTTCTGTTTTGTTTCTGTT<br>CTTGAAGTTGTCTGAGAAGATATTCAGGAGCTTGGATGCTACAACTCGAAGTATG<br>AGGTTCACATTGCCTTTCCCCATGCTTGCTTATCCTTTCTATCTGGTGAGTTTGTATC

	TACAAGAAAGTTCCTTTACAAAATGTGGATTTAGTTAACAGAAGCTAAAAGGGTT<br>CTTTCTTCTTGTTCCGTAATGCAGTGGAACCGGAGTCCTGGAAAAAAGGGGTCTC<br>ATTTCGACCCGAGTAGTGATTTGTTTGTGGCGAGTGAGAAGAAAGATGTGATCAC<br>TTCCACTGTTTGTTGGACAGCTATGGCTGCTTTGCTCGCAGGCTTGTCCTTTGTAAT<br>GGGTCCAATTCAGATGATCAAGCTATACGGAATTCCATATTGGGTATAGACAACA<br>TCTTTTGCCTTATCTTGCAAACTTGTTGTTTCAACTCTCAACCAACCACTCTGCCTC<br>TTGGATGTTTTTGTAGGGATTTGTTATGTGGTTGGATTTTGTAACTTACTTGCATCA<br>CCATGGTCACGACGATAAGCTTCCTTGGTACCGCGGCAAGGTAATCCATGTTCTTT<br>CCTCCTGTGTCCATCTATCTGGTCTTTCATGTTGAATCACTATATCCTAAAATCTTT<br>TCAATGTATAATTATTGATTCAAACCAAATGAAAAGTCAATTGTTTTGAGATGTCT<br>TTCTAGACTTTCAGTGAGATTTTGTTGAACTTATGCTTGGAACTTGGAACAGGAAT<br>GGAGTTACTTGAGAGGAGGGTTAACCACCATTGATAGAGACTATGGATGGATCA<br>ACAACATCCACCACGATATTGGAACCCATGTTATTCACCATCTCTTCCCACAAATC<br>CCTCACTACCACTTGATTGAAGCAGTAAGCAATAACTTTCCTTTTTCCCTTTAGTTC<br>CATATGCAATCTCTTCAATGGGGACACTGAGAAAAAGAAACAAACTTTGACCTG<br>AAAACAAATATCATGATGTTTCAGACGGAAGCTGCAAAGCCAGTGCTTGGGAAG<br>TACTACAAGGAACCAGCAAAATCAAAGCCGCTCCCTTTCCACCTAATCGGAGACT<br>TGATAAAGAGCTTGAAGAGAGACCATTATGTTAGTGACACCGGAGATGTCGTCTA<br>CTATCAAACTGACCCTGAACTCCAAAGACCCTCATCATCATCATGA
FAD3d-2	ATGGCGAGCTGGGTTCTATCAGAATGCGGCATAAAGCCACTCCCTTCCGCCTTCC<br>CCAAGCCCAGAACCGGAGCGCTTTCCCGGAATACCCTCCCCAAGCTCAGGTATTT<br>ACCCCCCAGGAGCAATCTCTCCGCCGCTGCCGACGGTGGCGCCTCAGATCTAAGC<br>CTTAACCTACCTAAATTATCCTCCGTAGAGAAGAAAAGGCCGCTTTTGGGGCAGG<br>TGAGAGTTACTGCTCCGTTCAAGGTCGCTCCGGTGAAGGAGGAAGACGGAGGAG<br>GAGAGTCCAATTTCGACCCAGGAGCGCCGCCGCCGTTCAATTTGGCCGACATTCG<br>AGCTGCTATACCTAAGCATTGCTGGGTTAAGGATCCATGGAGGTCAATGGCCTAT<br>GTGGCGAGAGACGTGGCTGTTGTTCTCGGATTGGCAGCTGCCGCAGCTTTTCTGAA<br>CAATTGGATTGTTTGGCCGCTGTATTGGGCGGCTCAGGGGACTATGTTCTGGGCTC<br>TCTTTGTTCTTGGCCATGACTGGTGAGTGAAAATTCTCAATTTTCGCTACTGTGATT<br>ACTAATCTGATGTAAAAAGTGGGTGGAAAGTTCTCATTTTTCCTTTTGCAGTGGAC<br>ATGGGAGCTTTTCAAGCAATCACAAGCTGAACAGTGTAGTTGGACACATTCTGCA<br>TTCTTCAATTCTTGTGCCTTACCATGGATGGTAAAAAGCTCTCTGCTTTCCTCTGCT<br>CATCCGCCATTTTGTTCTTCAAGTTTGAAGCAATTGAAAAAATAATTGTATCAATG<br>TCTTTACAGGAGAATTAGCCATAGGACGCATCATCAGAACCACGGCCACGTTGAG<br>AACGATGAGTCATGGCATCCTGTGAGTCTTTGTATCTAAATCTTAGCCATAGGGTT<br>GATTCAGATGCCGATGTACTAATTTCTGTGACTTTGTTTTGTTTCTGTTCTTGAAGTT<br>GTCTGAGAAGATATTCAGGAGCTTGGATGCTACAACTCGAAGTATGAGGTTCACA<br>TTGCCTTTCCCCATGCTTGCTTATCCTTTCTATCTGGTGAGTTTGTAGCTGCAAGTA<br>AGTTTCTTTACAAAATATGGATTTAGTTAACGAAGCTAAAAGGGTTATTTCTCCTA<br>ATGCAGTGGAACAGGAGTCCTGGGAAAAAGGGGTCTCATTTCGACCCGAGTAGC<br>GATTTGTTTGTGGCGAGTGAGAAGAAAGATGTGATCACTTCCACTGTTTGTTGGAC<br>AGCCATGGCTGCTTTGCTCGCAGGCTTGTCCTTTGTAATGGGTCCAATTCAGATGA<br>TCAAGCTCTACGGAATTCCATATTGGGTATAGACAACATCTTTTGCCTTATCTTAC<br>AGACTTGTTGTTTCAACTCTCAACCAACCACTCTGCCTCTTTGATGTTTTTGTAGGG<br>ATTTGTTATGTGGTTGGATTTCGTAACTTACTTGCATCACCATGGTCACGACGATA<br>AGCTTCCTTGGTACCGCGGCAAGGTAATCAATGTTCTTTGCTCCTGCGTCTATCTAT<br>CTAGTCTTTCATGTTTAATCACTATATCCTAAAATCTTTTCAATGTATAATTCTTGA<br>TTCAAACCCGATGAAAAGTCAATTGCTTTGAGATGTCTTTCTAGGCTTTCAGTGAC<br>ATTTTGTTGAACTTGTGCTTGGAACTTGGAACAGGAATGGAATTACTTGAGAGGA<br>GGGCTAACCACCATTGATAGAGACTATGGATGGATCAACAACATCCACCACGAT<br>ATTGGAACCCATGTCATTCACCATCTCTTCCCACAAATCCCTCACTACCACTTGAT<br>TGAAGCAGTAAGCACTAACGTCCCATTTTCCCTTTAGTTCCATACGCAATCTCTTC<br>AATGGGGAAACTGAGAAAAAGAAACGAACTTTGACCTGAAAACAAATATCATG

	ATATTTCAGACGGAAGCTGCAAAGCCAGTGCTCGGGAAGTACTACAAGGAACCG<br>GCGAAATCAAAGCCGCTCCCTTTCCACCTAATCGGAGACTTGATAAAGAGCTTGA<br>AGAGAGACCATTATGTTAGTGACACCGGAGATGTCGTGTACTATCAAACCGACCC<br>CGAACTCCAAAGACCCTCATCATCATCATGA
SAD2-1	ATGGCTCTCAAGCTCAACCCAGTCACCACCTTCCCTTCAACACGCTCCCTCAACA<br>ACTTCTCCTCCAGATCTCCTCGCACCTTTCTCATGGCTGCTTCCACTTTCAATTCCA<br>CCTCCACCAAGTAAGCATCTCCTCCTCCTCGGAATCTCCGCCGATTTCTTTTAAGC<br>GATTGATCGTAGATAAATTTGTCGGTTGCTTACCGTTCATCAAAATCTGCACGGTT<br>CGTTTCTTCTTCTGCGCCTAGATTGCATTATGTCATTGTTCGTTTTCCGATTTGACTG<br>ACCGACATAAATCAATTCCTTTGTGTTTCACGATTCTGGGTTTTGCGCTGTAATTGA<br>TTGTCAGTGTTTGCACAGGTTTCCCCTTCTCCTCCTCCGTCCATCAAATGCATGTTA<br>TTACCATTTCAATTTCAGTTTCCTTCTCTGAAATATCCGTCTCTGGGAAAATAAGTC<br>TCTGTATCTACTATCCTATCAGCTTGTTTAGGAGAGGTTCGATATTCGTTTACATAA<br>ACCAATTGGCTTACAGTCCTTGAACGTTCTAAATGTTGGTCGCGGTGATAATAGGT<br>TCTCAAAAGAGGTTTGTCTATGTTGTTTGGCAAAATCTTGTTTCTGTGAATCATGTT<br>TAAGGTCCTTGGAAGAATGACTAATGAGCTATGACATGATTACGACGTAGTAGTT<br>ATTGAACTGCTGATAATTCAATATAGGGGTAACTTTGTTGATTGTTTGGTCACAGG<br>GAGGCTGAGAAGCTAAAGAAGTCACATGGACCACCAAAAGAGGTGCATATGCA<br>AGTGACCCATTCCATGCCCCCACAGAAGCTGGAGATATTTAAGTCTCTGGAAGGT<br>TGGGCTGAGGATGTTCTATTACCGCACCTGAAGCCAGTTGAGAAATGCTGGCAGC<br>CACAGGATTTCCTGCCCGAACCTGAGTCGGATGGGTTCGAGGAGCAAGTGAAGG<br>AGCTCAGGGCAAGGGCCAAAGAACTGCCCGATGACTATTTTGTTGTGCTGGTTGG<br>GGATATGATCACCGAAGAAGCTCTGCCGACTTACCAGACAATGCTCAACACCCTT<br>GACGGGGTGAGGGACGAGACTGGAGCCAGCCTTACGCCGTGGGCAATCTGGACA<br>AGGGCGTGGACCGCTGAAGAGAATAGGCACGGTGACCTTCTCAACAAGTATCTA<br>TACCTCTCTGGAAGGGTGGACATGAGGCAAATTGAAAAGACCATTCAGTATCTCA<br>TCGGCTCTGGAATGGTATGTAATCACATACTTCATCCTTTTCTATTAATCTTTGCGT<br>GAACAAAATTCACTACACTGGTAGCAGCTGAAACTTTAGATGATTTTTTTACTGC<br>CTAGCTTCTATGAAACAAAACCACGTAAGTCAAATAGGGTTGACAATGAGTTCAA<br>GTGGCAAAATTTTTCTTATATACCAACTTCGAACCACTTTATATGACATACCAACT<br>CCTAGTTCGGTTAAAATTCCTCCGTCGAAGATATAATACTTGGATTGGTTAAATGA<br>ATTGTGAAAGGATACACGTGATGTGGTCTGGAATTAATTTGTTTGAATGATCAGTT<br>GGGTTCGGGGCGACAACTGTGAACTGGAACCACCCTAAGTAAATTTTCTTTCTGTC<br>CTACAAATTTGAGGTTCTCCTTGATCACCTTAGTCCATCTTAGGTTTGCCCGTTAGT<br>AAGATCTGCATTTAGCAGTTTGTCCTGGTATTTGATATCACTAGTATCTTTGTTTGA<br>TTCCCTAGCATCTCTGAAACCATCGGACAAGTAGGTGGTTTAGGACAAATTTGGTT<br>CATTGCGGCATTTTTTGTTTGTATCGCCGTATCATCTGGAAGAAGCAGACAGTTTT<br>GCAAAGTGGCATCAAGCTCAAGAAAGCAACGGCTAGAAGAAGTTCTACATCTGA<br>TGCTTTCCTTTTGTTTCTTTGTGTGCTTTTTGGACTTTGTTCTTTTTTCCTGTAGGATC<br>CAAAAACAGAAAACAACCCCTACCTCGGTTTCATCTACACCTCATTCCAAGAGAG<br>GGCAACGTTCATCTCCCACGGAAACACAGCCAGACTCGCCAAGGACCATGGGGA<br>CATGAAGCTGGCGCAGATCTGCGGGATCATCGCAGCAGACGAGAAACGGCACGA<br>AACCGCATACACCAAGATCGTCGAGAAGCTCTTCGAGATCGACCCTGACGGTAC<br>AGTGCTGGCACTGGCGGACATGATGAGGAAGAAGATATCGATGCCCGCCCACTT<br>GATGTACGATGGAGAAGACGACAACCTCTTCGACAATTACTCGTCAGTCGCTCAA<br>CGCATCGGGGTGTATACTGCCAAGGATTATGCCGATATCCTGGAGTTCCTGGTGG<br>GGAGGTGGAAAGTGGATGCTTTTACGGGGCTTTCCGGGGAAGGGAACAAAGCTC<br>AGGATTTTGTCTGCGGGCTTCCTGCGAGGATTCGAAAGTTGGAGGAGAGGGCTGC<br>GGGGAGGGCAAAGCAAACGTCGAAATCTGTCCCGTTCAGCTGGATCTTCAGCAG<br>AGAATTGGTACTCTAA
SAD2-2	ATGGCTCTCAAGCTCAACCCAGTCACCACCTTCCCTTCGACCCGCTCCCTCAACA<br>ACTTCTCCTCCAGATCTCCTCGCACCTTTCTCATGGCTGCTTCCACTTTCAATTCCA<br>CTTCCACCAAGTAAGTTCCCGTCACCATCTCCTCTTCCTCGGAATCTCCGCCGTTTC

	ATTTAAGCGATTGATCGTAGAAAATCTGTCGGTTGCTTAGCGTTCATTCAAATCTG<br>CGCGGTTCGTTTCTTTTTCTTTCTTCAGACTGCCTCGTCTGCATTATGTTATTGTTCG<br>TTTCCGATTTGACTAACCTACATAATCAATTCCTTTGTGTTTCACGAGTCTGGATTT<br>TGCGCTGTAATTGATTGTCAGCGTTTGCACAGGTTTCCATTTCTCCACCTCCGTCCA<br>TCAAATGCATGTTATTACCTACCAATTTCAGCGTCTTTCTCTGGAAATTTCTGTCTC<br>TGTATCTACTATCCTATTAGCTTGTTTGAGAGAGGTTCAATATTGGTTTGCATGAAC<br>CAAGTGGCTTACAATCCTTCAACGTTCTAAATGTTGGTCGCAGTAACAATAGGTTC<br>TCAAAAGAGGTTTTTCTATGTTGTTTGGCAAAATCTTGTTTCTGTGAATCATGTTAA<br>GGTCCTGGGAAGAATGATTAATGAGCTATGACATGATTAAGGCGTAGTAGTTATT<br>GAACTGCTGATAATTCAATATAGGGGTAACTTTGTTGGTTGTTTGGTGACAGGGAG<br>GCTGAGAAGCTAAAGAAGTCACATGGACCACCAAAAGAGGTGCATATGCAAGTG<br>ACCCATTCCATGCCCCCACAGAAGCTGGAGATCTTTAAGTCCCTTGAAGGTTGGG<br>CAGAGGACGTTCTGTTGCCGCACCTGAAGCCGGTTGAGAAATGCTGGCAGCCACA<br>AGATTTCCTGCCCGAACCCGAGTCGGATGGGTTCGAGGAGCAAGTGAAGGAGCT<br>CAGGGCAAGGGCTAAAGAACTCCCCGATGACTATTTTGTTGTGCTGGTTGGGGAT<br>ATGATCACCGAAGAAGCTCTACCGACTTACCAGACAATGCTCAACACCCTTGACG<br>GGGTGAGGGACGAGACTGGAGCCAGCCTTACGCCGTGGGCAATCTGGACAAGGG<br>CGTGGACCGCTGAAGAGAATAGGCACGGTGACCTTCTCAACAAGTATCTTTACCT<br>CTCTGGAAGGGTGGACATGAGGCAAATTGAAAAGACCATTCAGTATCTCATCGGC<br>TCTGGAATGGTATGTACTCACATCCTATCTGCTCCTTTATCCTTTTCCATTAATCTTT<br>GATTGAACAAAATTCAATAAACTGGTAGCTGAAACTTTAGATGATTTGTTATAAC<br>TGCCTAGCTTCTATGAGAAAACCACTGAAGTCAAATAGGTTTGACAATGGGTTTA<br>AATGGAAAAAGTTTCATATACCATCTTCCATCTATTTTACATGACATACCAACTTC<br>TACTTCGGAGAAAATTCGCCGTGGATAATCATATTATTGAAGATATAGTACTTAGT<br>AGATTGGTTAGATGAACTGTTAAACAATACATGTGATGTCGTGTGCAATTAATTTG<br>TGTAAATGATTAGCTGGGTTCGGGACGACAAATGTGAACTGGAACCCTAGTAAAC<br>TATGAATTGAGGTTGTCCTTCATCACTTTATTCTGTCCTGGGTTTGTTTGCCTGTTTG<br>CAAGATCTGCATGTAGCAGTTTGTCCTGGTATTTGCTACCAGTGGTATCTTTGTTTG<br>ATTCCCTAGCATCTCTGAAAACATCGGACCAAGTATCTGGTTAGGACAAATTTGG<br>TTCATTGCGGCATTTTTTGTTTGTATCGCTGTATCGTCTGGAAGAAGCAGACAGTTT<br>TGCAAAGTGGCATCAAGCTCAAGAAAGCAACGGCTAGAAGAAGTTCTACATCTG<br>ATGCGTTCCTTTTGTTTCTTTGTGTGCTTTTTGGACTTTGTTCTTTTTGCCTGTAGGAT<br>CCAAAAACAGAAAACAACCCCTACCTCGGTTTCATCTACACCTCATTCCAAGAGA<br>GGGCAACGTTCATCTCCCACGGAAATACGGCCAGACTCGCCAAGGACCACGGGG<br>ACATGAAGCTGGCGCAGATCTGCGGGATCATCGCAGCAGACGAGAAGCGGCACG<br>AAACAGCATACACCAAGATCGTCGAGAAGCTCTTCGAGATCGACCCTGACGGTA<br>CAGTGTTGGCTCTGGCGGACATGATGAGGAAGAAGATATCGATGCCCGCACACTT<br>GATGTACGATGGAGAAGACGACAACCTCTTCGACAATTACTCGTCGGTCGCTCAA<br>CGCATCGGGGTGTATACTGCCAAGGATTATGCTGATATCCTGGAGTTCCTGGTGG<br>GGAGGTGGAAAGTGGATGCTTTTACGGGACTTTCCGGGGAAGGGAACAAAGCTC<br>AGGAGTTTGTCTGTGGGCTTCCAGCGAGGATTCGAAAATTGGAGGAGAGGGCTGC<br>GGGGAGGGCAAAGCAAACGTCGAAATCTGTCCCATTCAGCTGGATCTTCAGCAG<br>AGAATTGGTACTCTAA
SAD3-1	ATGCAAGCCACCCACCACGTCAGCGCCCACCGTCCAGCATGGATGCCACGTGGT<br>GGCCGAACCACCTCCACCACCATCATGCACCTCTGCCCTCCACGCGCCGCCATCA<br>AAACTGCTCCGCCGCCTCCCCCTCCGCTCACCCTGAAACACCAACACAAAACCCA<br>CACAATGCCGGCGGAGAAAATCGAGCTTTCAAGTCCCTCGAGGATTGGGCCTCC<br>GAGAATGTCCTCCCACTTCTCAAGCCCGTCAATAAATGCTGGCAGCCTCAGGACT<br>TCCTCCCCGACCCGTCCCTCTCCGTCGCTGATTTCTCCGACGAGGTAATGAACAAT<br>TGCGTCTCAATACCTTGTATAAGCTACACTTTTACAGGGATTTTGAGCTGGTATTTG<br>ACAAATTCATCAGTTAGGTTTTTTGTCATTACGGATTAGTCATTGTAGTTTTGTCAT<br>TTGTTCATATAGCTACTCATTTTTCAGTTCATCAAATTCTCATTTTCTATAGTAAGA<br>TCATTTGGAAGGGTTTTGGAGAGTAATTTCTTGGTCCCACATGATTTGATCGAAAT

	ATTATTTTAGAATACAAAAATAAATTGGGATTTGAAATCACCTCGATAGACTAGG TTCCCCAAAAGGTGATTTCAAATTCATTTTATGGACTTGAGATTTGCAAAATCTCA AGTGATTTGAATAGTAAATGATCAAATTATCTTGTTGAGTTTAAGTGAACGAGAT AACTTAAGGGGTGCATTAGTCATTTAAAAAATCAAATTTTTTTTAAATTTTCAAA ACTATTTTGTGATTGACCGTTAAACCAGTTCGGTTTGATACTGATTTTGAGTTTTGA CTGACAGGTGAGGTTGTTGAGGGAAAGGACGGCGGAGCTACCGGACGAGTACTT CGTCGTCTTGGTGGGTGACATGATAACGGAGGATGCGTTACCGACGTACGAGACC ATGATCAACACACTTGACGGCGTTAGGGACGAGACGGGCGCCAGTCAAAGCCCA TGGGCCTTGTGGACCCGGGCCTGGACCGCCGAGGAGAATCGCCACGGCGATCTG CTTCGGACCTTTCTGTACCTATCGGGTCGGGTCGACATGAAGATGGTAGAAAGGA CTGTTCAGTACCTCATTGGATCTGGCATGGTAAGTGTAGAATAGCCCTTTCGGTAT TCCGAACCGGTTTGATCCGTCAGTGGGATCGTTCGTTCGATTAATCGTTCGATTAA TCGGGTGATGAGTATGCCAAATATTTATCACTTGATTTGATTTGATAGTACATTTTA TAAGCTAGTGTTAGCTAATTATATTGTTTGAGAATGTCACGAATTCGAAGGAGAA AAAAACCAAAATGCCACTTGGATAATTTATTTTACATGATATAGTCATTGAACCTT CAATCTTTTTACAAATCGGATCCAAATTTGTGTACGCAGTGGTCACTCAACTGTCA TTTTAGAATAGGAATAGCACTTTAACAAGTGATTTACTCGGGTCGGGTAACATTA CAGGACCCGGGCACAGAGAACAACCCGTACTTGGGGTTCGTGTACACGTCATTCC AGGAGCGAGCCACATTCATATCTCACGGCAACACGGCTCGGCTAGCCAAGGAAG GAGGGGATCCGGTACTCGCACGGATATGTGGCACCATAGCATCGGACGAAAAGC GACACGAGAACGCCTACACGCGCATCGTGGAGAAGCTCCTAGAGGTGGACCCGA ACGGTGCGGTTTGTGCGGTGGCCGACATGATGCATAAGAAGATCACAATGCCGG CGCACCTGATGTACGACGGGGAGGACCCGAAGCTGTTTGAGCACTTCTCATCGGT GGCTCAGCGGCTCGGGGTGTACACCGCACAGGACTACGCAGACATACTCGAATT CTTGATCGGACGGTGGGGACTGGACAAGGTGGAGCAAGGGCTGGACGGTGAAGG GAGGCGGGCGCAGGATTTCGTGTGTGGGCTGCCGCAGAGGATCATGCGGCTCCA GGAGCGAGCCGATGAGAAGGCTAAGAAGATGAAGAAGCCGGAAGCCGTCAAGT TTAGCTGGATTTTCAAACGGCAAGTCGTTTTGTGA
SAD3-2	ATGCACCTCTGCCCTCCACGCGCCGCCATCAAAACTGCTCCGCCGCCGCCTCCTC CGCTCACCCTGAAACACCAACACAAAACCCACACAATGCCGGCGGAGAAAATCG AGCTTTTCAAATCCCTCGAGGATTGGGCCTCCGTCAATGTCCTCCCACTTCTCAAG CCCGTCGACAAATGCTGGCAGCCTCAGGACTTCCTCCCCGACCCGTCCCTCTCCGT CGCTGATTTCTCCGACCAGGTAATGAACCATTGTGTTCCAATAACTCGTATTGTAA TGTTACTCGTATTTTGAGCTAATATTTGATAAATTCGTCACTTAACTTTTTTGTCTTG ACGGATTAGTCATTGTAGTTTTATTATTTGTTCGATTTTCAGTTAAATAATTCTCATT TTTTATAGTAAGATCATTTGGAAGGAATTTGGAGAGTAATTTCATGGTCCTGGGTT GATTTGATCGAAATATCGCGTTGGAACACGAAAATAAAGGTGGATTTGAGTAGA AAATAACCAAATTGACTTGTTGAAATTGTATTGTTGAGTGAACAGGGGGTTTATTA GTCATTTACAAAATTAAAATGTTTCTTTTAATGTCCAAAACTCAACTAAACACTGA TTTTGAGATTGGCCGTTAAACCAGTTCGGTTTGATAAATACTGATTTTGAGTTTTGA CTGACAGGTGAGGTTGTTGAGGGAAAGGACGGCGGAGCTACCGGACGAGTACTT CGTCGTCTTGGTGGGTGACATGATAACGGAGGATGCGTTACCGACGTACGAGACC ATGATCAACACCCTTGACGGCGTTAGGGACGAGACGGGCGCCAGTCAAAGCCCA TGGGCCCTATGGACCCGAGCCTGGACCGCCGAGGAGAATCGCCACGGGGATTTG CTCCGGACCTTTCTGTACCTCTCGGGTCGGGTCGACATGAGGATGGTTGAAAAGA CTGTTCAGTACCTCATTGGATCTGGCATGGTAAAGTGCAGAATAGTCCTTTTTGTA TTCCGAACTGGTTTGATCGGTTGTTTGGTTCGTTCGTTTGTTAATTGGGTGATGAGT ATGCGAAATTGTCACTAAATTATCACTTCATAGTACATTTATAAGCTAGTGTTTAG TAACAATAGTTTAAGAATGTTACAAATTTGAACCGGGGAAAAACCAAAATGCCA CTTGGATAATTTATTTTGCATGATATAGTCATTGAACCTTTAATCTTCTTACAAATC GGATCCTAATTTGTGTACACATTGGTCACTCAACTGTCATTTTAGAATAGGAATAA CATTTTAACAAGTGATTACTCGGGTCGGGTAACAATTGCAGGACCCGGGCACGGA GAACAACCCGTACTTGGGGTTCGTATACACGTCATTCCAGGAACGAGCCACATTC

	ATATCGCACGGCAACACGGCTCGGCTAGCCAAGGAGGGAGGGGATCCGGTACTT<br>GCACGAATATGCGGCACCATAGCATCGGATGAGAAGCGACACGAGAACGCCTAC<br>ACGCGCATCGTGGAGAAGCTCCTCGAGGTGGACCCGAACGGTGCGGTTTGTGCCG<br>TGGCCGACATGATGCGTAAAAAGATCACAATGCCGGCGCACCTGATGTACGACG<br>GGGAGGACCCAAAGCTGTTCGAGCACTTCTCGGCGGTGGCGCAGCGGCTCGGGG<br>TGTACACGGCGCAGGACTACGCGGACATACTGGATTTCCTGATCGGACGCTGGGG<br>GCTGGACAAGGTGGAACAAGGGCTGGACGGTGAAGGGAGGCGGGCGCAGGATT<br>TCGTGTGTGGGCTGCCGCAGAGGATCATCCGACTCCAGGAGCGAGCCGATGAGA<br>AGGCTAAGAAGATGAAGAAGCCGGAAGCCGTCAAATTTAGCTGGATCTTCAAAC<br>GGCAAGTCGTCTTGTGA
**3896**	
FAD2a-1	ATGGGTGCCGGTGGCAGAATGTCAGTGCCTCCATCATCCAAACCTATGAAGAGGT<br>CTCCTTACTCAAAGCCACCATTCACGCTCGGTGAGCTCAAGAAGGCCATCCCTCC<br>ACACTGTTTCAAACGCTCAATCCCCCGATCGTTCGCCTACGTGGCGTACGACCTCA<br>CCATTGCAGCAATCTTCTACTACATCGCCACCACTTACTTCCACCTCCTCCCTAGC<br>CCTCTCAACTACCTCGCCTGGCCGGTCTACTGGGCCTGCCAGGGCTGCATCCTCAC<br>TGGAGTATGGGTGTTGGCTCACGAATGCGGTCACCATGCCTTCAGCGACTACCAG<br>TGGCTCGACGACATGGTTGGCTTCGTCCTCCATTCGTCCCTCCTTGTTCCTTACTTC<br>TCCTGGAAGCACAGCCACCGCCGCCACCATTCCAACACGGGATCGCTTGATCGTG<br>ATGAGGTGTTTGTCCCCAAGCAGAAGGCCGAAATCGGGTGGTACTCCAAGTACCT<br>TAACAACCCACCTGGCCGTGTGATCACATTGGCCGTCACATTAACGCTCGGTTGG<br>CCTCTGTACTTGGCATTCAACGTCTCCGGGAGACCATATGACCGGTTCGCATGCCA<br>TTTTGACCCTCACGGTCCGATTTACAATGATCGCGAGCGTATGGAGATATACCTAT<br>CCGACGCAGGGATATTCACCGTGTGCTACATCCTATACAGACTCGTCCTCACGAA<br>AGGACTCGTTTGGGTCGTGTCCATATACGGAGTCCCACTATTGATAGTGAATGGAT<br>TCCTAGTCCTCATCACTTTCTTGCAGCACACGCATCCTTCTCTTCCGCACTACAAGT<br>CCTCCGAATGGGACTGGATGCGAGGCGCCCTCTCGACCGTGGATCGAGACTACGG<br>GTTACTCAACACCGTGTTCCACAACATCACCGATACACATGTCGCGCACCATCTC<br>TTCTCCACGATGCCTCATTACCACGCGATGGAGGCTACCAAGGCGATCAAGCCGG<br>TTCTCGGGGAGTATTACCAGTTCGATGGGACTCCCTTTGTGAAGGCCATGTGGAG<br>GGAGGCAAAGGAGTGCATCTATGTCGAGCCGGATGAAGGCGACCCCAGCCAAGG<br>CGTGTTCTGGTACAACAACAAGCTGTGA
FAD2a-2	ATGGGTGCCGGTGGCAGAATGTCAGTGCCTCCATCATCCAAACCTATGAAGAGGT<br>CTCCTTACTCAAAGCCACCATTCACGCTCGGTGAGCTCAAGAAGGCCATTCCTCC<br>ACACTGTTTCAAACGTTCAATCCCCCGATCGTTCGCCTACGTGGCGTACGACCTCA<br>CCATTGCAGCAATCTTCTACTACATCGCCACCACTTACTTCCACCTCCTCCCTAGC<br>CCTCTCAACTACCTCGCCTGGCCGGTCTACTGGGCCTGCCAGGGCTGCATCCTCAC<br>TGGAGTATGGGTGTTGGCTCACGAATGCGGTCACCATGCCTTCAGCGACTACCAG<br>TGGCTCGACGACATGGTTGGCTTCGTCCTCCATTCGTCCCTCCTTGTTCCTTACTTC<br>TCCTGGAAGCACAGCCACCGCCGCCACCATTCCAACACGGGGTCGCTTGATCGTG<br>ATGAGGTGTTTGTCCCCAAGCAGAAGGCCGAAATCGGGTGGTACTCCAAGTACCT<br>TAACAACCCACCTGGCCGTGTGATCACATTGGCCGTCACATTAACGCTCGGTTGG<br>CCTCTGTACTTGGCATTCAACGTCTCCGGGAGACCATATGACCGGTTCGCATGCCA<br>TTTCGACCCTCACGGTCCGATTTACAATGATCGCGAGCGTATGGAGATATACCTAT<br>CCGACGCAGGGATATTCACCGTGTGCTACATCCTATACAGACTCGTCCTCACGAA<br>AGGACTCGTTTGGGTCGTGTCCATTTACGGAGTCCCACTATTGATAGTGAATGGAT<br>TCCTAGTCCTCATCACTTTCTTGCAGCACACGCATCCTTCTCTTCCGCACTACAAGT<br>CCTCCGAATGGGACTGGCTGCGAGGCGCCCTCTCGACCGTGGATCGAGACTACGG<br>GTTACTCAACACCGTGTTCCACAACATCACCGACACACATGTCGCGCACCATCTC<br>TTCTCCACGATGCCTCATTACCACGCGATGGAGGCTACCAAGGCGATCAAGCCGG<br>TTCTCGGGGAGTATTACCAGTTCGATGGGACTCCCTTTGTGAAGGCCATGTGGAG<br>GGAGGCAAAGGAGTGCATCTATGTCGAGCCGGATGAAGGCGACCCCAGCCAAGG<br>CGTGTTCTGGTACAACAACAAGCTGTGA

FAD2b-1	ATGGGTGCTGGCGGAAGAATGGCCGTGCCTCCATCGAACAAGGCGGACTCCGAA<br>ACCTTTAAGCGGTCTCCTTACTCAAAACCTCCCTTCACTCTTGGTGAGATCAAGAA<br>AGCCGTCCCTCCACACTGCTTCAAAAGGTCCATCCCCCGCTCGTTCTCCTACGTGG<br>CTTATGACCTCACCATAGCCGCCATCTTCTACTACATCGCCACCACTTACATCCAC<br>CTCCTCCCCAATCCTCTCTCCTACGTGGCGTGGCCGATCTACTGGGCCTGCCAAGG<br>CTGCGTCCTCACTGGTGTCTGGGTCCTAGCCCACGAATGCGGTCACCATGCCTTCA<br>GCGACTACCAATGGCTCGACGACTTGGTCGGCTTTGTCCTCCACTCATGCCTCATG<br>GTACCCTACTTCTCGTGGAAGCACAGCCACCGTCGCCACCACTCCAATACTGGGT<br>CCCTCGAACGAGACGAGGTTTTTGTCCCCAAGCAGAAATCAGCCATTGGCTGGCA<br>CTCAAAGTACCTCAACAACCCACCTGGCCGTGTGCTCACACTTGCAGTCACTCTC<br>ACTCTCGGCTGGCCTTTGTACTTGGCATTCAACGTCTCTGGAAGGCCGTACGACCG<br>GTTCGCCTGCCATTACGATCCTAAATCCCCCATCTACAACGACCGCGAGCGAACG<br>GAGATATTCTTCTCCGATGCTGGCATCCTTGCTGTGAGCTTTGCGCTCTACAAGCTT<br>GCTGTCGCCAAGGGACTGGCTTGGGTGGTTTGTGTCTACGGAGTTCCACTCCTTGT<br>AGTGAATGGATTCCTTGTCTTGATCACTTTCTTGCAGCACACCCACCCATCATTGC<br>CGCACTACAAATCCTCCGAATGGGACTGGCTGAGAGGTGCTCTGGCGACCATGGA<br>CAGAGACTACGGGTTTCTGAACACGGTGTTCCATAACATCACGGATACCCACGTG<br>GCGCACCACCTGTTCTCGACGATGCCTCATTACCATGCAATGGAAGCTACAAAGG<br>CGATCAAGCCGGTATTGGGAGAGTACTACCAATTCGACGGGACTCCATTCATCAA<br>GGCGATGTGGAGGGAGGCTAAGGAGTGTGTTTATGTCGAGCCCGACGAAGGTGA<br>CCAGAACAAAGGCGTGTTCTGGTACAACAACAAGCTGTGA
FAD2b-2	ATGGGTGCTGGTGGAAGAATGGCTGTGCCTCCATCGAACAAGGCGGACTCGGAA<br>ACCTTTAAGCGGTCTCCATACTCAAAACCTCCATTCACACTTGGTGAGATCAAGA<br>AAGCCATCCCTCCACATTGCTTCAAAAGGTCCATCCCCCGCTCATTCTCCTATGTG<br>GCTTATGACCTCACCATTGCCGCCATCTTCTACTACATCGCCACCACTTACATCCA<br>CCTCCTCCCCAATCCTCTCTCCTACGTTGCGTGGCCTATCTACTGGGCCTGCCAAG<br>GCTGCGTCCTCACCGGTGTCTGGGTCCTAGCCCACGAATGCGGTCACCACGCCTTC<br>AGCGACTACCAATGGCTTGATGACTTGGTCGGCTTTGTCCTTCACTCATGCCTCAT<br>GGTCCCCTACTTCTCGTGGAAGCACAGCCACCGTCGCCACCACTCCAACACCGGG<br>TCCCTTGAACGAGATGAGGTTTTCGTCCCCAAGCAGAAATCGGCCATCGGCTGGC<br>ACTCAAAGTACCTCAACAATCCACCTGGCCGTGTGCTCACACTTGCAGTCACTCT<br>CACTCTCGGCTGGCCTTTATACTTGGCATTCAACGTTTCCGGAAGGCCGTACGACC<br>GGTTCGCCTGCCATTACGATCCTAAATCCCCTATCTACAACGACCGCGAGCGAAC<br>TGAGATCTTCCTCTCCGATGCCGGCATCCTTGCTGTGAGCTTTGCAATCTACAAGC<br>TTGCTGTCGCCAAGGGACTGGCTTGGGTGGTTTGTGTCTATGGAGTTCCACTCCTT<br>GTAGTGAATGGATTTCTTGTCTTAATCACTTTCTTGCAGCACACCCACCCATCATT<br>GCCGCACTACAAATCCTCCGAATGGGACTGGCTGAGAGGTGCTCTGGCGACCATG<br>GACAGAGACTACGGGTTTCTGAACACGGTGTTCCATAACATCACGGATACCCACG<br>TGGCACATCACCTGTTCTCGACGATGCCTCATTACCATGCGATGGAAGCTACCAA<br>GGCGATCAAGCCGGTACTGGGAGAGTACTACCAGTTCGATGGGACTCCATTCATC<br>AAGGCGATGTGGAGGGAGGCTAAGGAGTGTGTCTATGTTGAGCCTGACGAAGGT<br>GACCAGAACAAAGGCGTGTTCTGGTACAACAACAAGCTGTGA
FAD2c-1	ATGTTCTCATCCTCTGCAACTAAAATGCCGGCGCCGCCTTCATCATCCAACACGA<br>CCATGAAGCGATCGCCTCACTCAAAGCCGCCGTTCACCGTCTCCGACGTCAAGAA<br>AGCCATTCCACCACACTGCTTCCAAAGGTCCCTCCTCCGATCATTCTCCTACCTAA<br>CCTACGACCTCACCATCATCACAATCCTCTACCAAGTCGCCACCACTTACTTCCAC<br>CTCCTCCCGACACCTCTCTCATCCTACGTGGCATGGCCGGCCTACTGGGCCGGCCA<br>AGGCTGCTTCTTCGTCGCTGTATGGATGGTGGCCCACGAGTGTGGCCATCACGCCT<br>TCAGCGACCAGCACTGGCTGGAAGACTCGGTCGGATTCATCCTCCACTCAGCCCT<br>CCTTTCACCTTACTTCTCATGGAAGCACAGCCACCGTCGCCACCATGCCAACACG<br>AGCTCCCTCGAACGTGACGAGGTGTTCGTCCCTAAACCCAAGTCCAAGCTGAGCT<br>GGCACTTCAAGTTCTTCAACAACCCGCCTGGCCGCGTCCTGCAGTTGGCATTTGCT<br>CTGCTTCTTGGATGGCCTCTCTATTTGGCGATCAACATCGCCGGAAGGCCGTACGA

	GAAATTCGCTAGCCACTTCGATCCGAGAAGCCCTATCTACAACGATCGAGAGCGG<br>ATCGAGATATTCGCATCCGATGTTGGCGTGCTCTGCATGTGGTTCGCGCTGTATAA<br>ACTTGCTCTAGTGAACGGAGTTGGTTGGGTTGTTTGTGTGTACGGGATCCCGCTGT<br>TGGTGATGAATGGATGGGTCGTTACGATCACTTACTTGCATCACACGCATATAGC<br>GTTGCCGCGGTATGATTCGTCCGAATGGGATTGGTTGAGGGGTGCATTGGCGACT<br>GTGGATAGGGACTATGGTGTTTTGAACAAGGTGTTTCATAACATCACGGATACTC<br>ATGTGGCGCACCATCTGTTTTCGGCGATGCCTCATTACCATGCGGCGGAAGCGAC<br>CGAGGCGATCAAGCCCGTTTTGGGCGAGTATTATCGGTGTGACCGGACTCCTATT<br>ATCAAGGCGTTATGGAGGGAGTTTAAGCACTGTATTTATGTCGAGTCAGATGAGG<br>ATAAAGGCGTGTTTTGGTTCAATGACAAGTTGTAA
FAD2c-2	ATGCCGCCGCCGCCTTCATCATCCAACACAACCATGAAGCGATCGCCACACTCAA<br>AGCCGCCGTTCACCCTCTCTGACATCAAGAAAGCCATACCACCGCACTGCTTCCA<br>AAGGTCCCTCCTCCGATCATTTGCCTACTTAGCCTACGACCTCACCGCCGTCACAA<br>TCCTCTACCACATCGCCACCACGTACTTCCACCTCCTCCCGACACCTCTCTCATCC<br>TACGTGGCTTGGCCGGCCTACTGGGCCAGCCAAGGCTGCTTCTTCGTCGCTGTATG<br>GATGGTGGCCCACGAGTGCGGTCACCACGCCTTTAGCGACCAACACTGGCTGGA<br>AGACTCGGTAGGGTTCATCCTCCACTCGGCCCTCCTTTCGCCTTACTTCTCGTGGA<br>AGCACAGCCACCGCCGCCACCACGCCAACACTAGCTCCCTCGAGCGTGACGAGG<br>TGTTCGTCCCTAAGCTGAAATCCAAGCTGAGCTGGCACTTCAAGTTCTTCAATAAC<br>CCGCCTGGCCGCGTCCTGCAGCTGGCCTTTGCTCTGCTTCTAGGATGGCCTCTCTA<br>CCTTGCGGTCAACATCGCAGGAAGACCGTACGAGAAATTCGCTAGCCACTTCGAT<br>CCGCGAAGCCCTATCTATAACGATCGGGAGCGAAATGAGATATTTGTATCTGATG<br>TTGGCGTGCTTGCTATGTGGTTCGCTCTGTATAAACTTGCCCTGGCGAACGGAGTT<br>GGTTGGGTTGTTTGTGTGTACGGGGTCCCTCTGTTGGTGATGAATGGATGGATCGT<br>TACGATCACTTACTTGCATCACACGCATGTCGCATTGCCGCGGTATGATTCCTCGG<br>AGTGGGATTGGTTGAGGGGTGCGCTCGCGACTGTAGATAGGGATTATGGGGTTTT<br>GAACAAGGTGTTTCACAACATTACAGATACTCATGTGGCGCACCATCTGTTTTCG<br>ACGATGCCTCATTACCATGCGGCGGAAGCAACCGAAGCGATCAAGCCCGTTTTGG<br>GTGAGTATTATCGGTCTGACCGGACTCCTATCGTCAAGGCATTATGGAGGGAGAC<br>TAAGCACTGTGTTTATGTCGAGGTTGATGAAAAAAAAGGCGTGTTGTGGTTCAAT<br>GATAAGATGTGA
FAD2d-1	ATGGTATCCAATACCACCATAAAGCGAACACCGACCTCAAAACCGCCGTTCACC<br>CTCTCCGACGTCAAGAAAGCAATACCACCGCACTGCTTCCAAAGATCCCTCCTCA<br>AATCCTTCACCTACCTAGCCTACGATCTCACCGTCATCACAATCCTCTACCACATC<br>GCCACGTCATACTTCCACCTCCTCCCCAACCCTCTCTCCTACGTGGCGTGGCCGCT<br>CTACTGGGCCGCCCAGGGGTCACACTTCATCGCCGTCTGGGTCATCGCCCACGAG<br>TGCGGCCACCACGCCTTCAGCGACTACCAGTGGCTAGACGACGCCGTTGGGTTCG<br>TCCTCCACTCCCTCCTCTTAGCTCCTTACTTCTCCTGGAAGCACAGCCACCGCCGT<br>CACCACGCCAACGCCGCCTCCATCGAGCGCGACGAGAATTACATCCCGAAAAAG<br>AAGGACGAAGTCAACTGGCATTTTAAATACCTCGACAACCCTCCCGGACACGTGT<br>TCTATATATTCTTCACTCTCACCCTCGGATGGCCGCTCTACTTGCTGGTCAACATCT<br>CCGGCAGGAAATACGACGACGGATTCGCCAGCCATTTGTATCCTTTCTCCCCTATC<br>TACAACGACCGCGAGAGATTCGGGATAGTCCTCTCCGTCGCCGGGATGCTCGCCA<br>CGTGGTTCGGTCTTTATAAACTCGCGATGGTCAACGGGTTCGGTTGGGTTGTTTGC<br>GTGTACGGGGTGCCTCTCATTCTCCAGAATGCGATGTTGATTACCATCACTTACTT<br>GCACCACACGCATCTCAACTTGCCCCACTACGACTCGTCGGAGTGGGACTGGATG<br>AGGGGTGCGCTCGCGACTGTGGATAGGGATTACGGAATTTTGAATAAGGTGATGC<br>ATAATATTACGGATACCCACGTGGCGCACCATCTGTTCTCGATGATTCCTCATTAT<br>CATGCGATGGAGGCTACAAATGCGATAAAGCCGGTTCTTGGGGAGTACTACCAA<br>GTTGATACTACGCCGTTTTTGAAGGCGTTGTGGAGAGAGACTAAGGATTGTGTTTA<br>TGTTGAGGCGGATGATGAGGGATCTGATCGGGAGAAGAAAGGAGGGGTGTTTTG<br>GTTCAAAACCAAGCTGTAA

FAD2d-2	ATGGTATCCAATACCACCCTAAAGCGAACACCGACCACAAAACCGCCGTTCACC<br>CTCTCCGACCTCAAGAAAGCAATACCACCACACTGCTTCCAAAGATCCCTCCTCA<br>AATCCTTCTCCTACCTAGCCTACGACCTCACCGTCATCACAATCCTCTACCACATC<br>GCCACGTCATACTTCCACCTCCTTCCCAAACCTCTCTCCTACGTGGCCTGGGTGGC<br>CTATTGGGCCGCTCAGGGGTCCCACTTCATCGCCGTGTGGGTCATCGCCCACGAG<br>TGCGGCCACCACGCCTTCAGCGACTACCAGTGGCTAGACGACGCCGTCGGCTTCC<br>TTCTCCACTCCCTCCTCTTAGCTCCTTACTTCTCCTGGAAGCACAGCCACCGCCGC<br>CACCACGCCAACGCCGCCTCCATCGAACGTGACGAGAATTACATCCCGAGAAAG<br>AAGGACGAAGTCAACTGGCATTTTAAGTACCTTGATAACCCTCCCGGACACGTGT<br>TCTATATATTCTTCACTCTCACCCTCGGATGGCCGCTCTACTTGCTGGTCAACATCT<br>CCGGGAGGAAATACGACGACGGATTCGCCAGCCATTTGTATCCTTTCTCCCCGAT<br>CTACAACGATCGGGAGAGGTTCGGGGTGTTCCTCTCCGTCGCGGGGATGCTCGCC<br>ACGTGGTTCGGACTTTATAAGCTCGCAATGGTCAACGGTCTCGGCTGGGTTGTTTG<br>CGTGTACGGGGTCCCTCTGATTCTCCAGAATGCGATGTTGATTACCATCACTTACT<br>TGCACCACACGCATCTCAACCTGCCCCACTACGACTCGTCGGAGTGGGACTGGAT<br>GCGGGGTGCGCTCGCGACTGTGGATAGGGACTACGGGATTTTGAACAAGGTAAT<br>GCATAATATTACGGATACTCACGTGGCACACCATCTGTTTTCGATGATTCCTCATT<br>ACCATGCGATGGAGGCTACGAATGCGATCAGGCCGGTTCTGGGGGAGTACTACC<br>AGGCCGATACGACGCCGTTTATTAAGGCGTTGTGGAGGGAGACTAAGGACTGTGT<br>TTATGTTGAGGCGGATGATGAGGGCGGTGATCAGGAGAAGAAAGGAGGGGTGTT<br>CTGGTTCAAAACCAAACTGTGA
FAD2e-1	ATGTCCCCTATTTACAGCGACCGCGAGCGAGCCGAGGTATTCGCCTCAGATGTTG<br>GCTTGCTCGCTGTCTGCTTCGCGTTGTACAAACTTATTATGGTCAAGGGAATGGCG<br>TGGGTTTTTTGCGTCTATGGGGCTCCGGTCATGGTGGTGAATGGATTCTTCATTACC<br>ATCACTTACTTGCATCACACTCATCTCGCGGTCCCGCGATACGATTCGTCTGAATG<br>GGATTGGTTGAGGGGAGCCCTGGCAACCATGGACAGGGACTTTGGCCTTTTGAAC<br>AAGGTGTTCCATAATGTTACAGATACTCACGTGACGCACCATCTGATTTCGACGA<br>TCCCTCATTATCATGCCATGGAAGCCAACAACGCAATTAGGCCCGTGTTGGGGGA<br>CTACTACCATATCGACAGGACGCCGGTGGTTAAGGCGTTGTGGAGGGAGGCTAA<br>GGAGTGTGTTTACATCGAGGCCGATGATGGTGAAAAGAACAAAGGCGTGTTCTG<br>GTTCAATACCAAGCTCTAA
FAD2e-2 (exon-intron junctions are not given)	ATGCACTTTCCGATAGCTAACCTTTGTCTGATGCTAAAAACAGGCCATACATGGC<br>GCCACAAAGCACTGCTCTAGTAAGTACACATGATTGGGATTACACTACACTTGAG<br>TAATTAAATCAGTCCATATTTTACCTGGATCCAAAGTTGAAAACTAACTCGAAAA<br>CAAATTTGTGCAAAATTGAGCATCAAATGAGACAATATTAAACTATTTCGAGTTTT<br>GTTGGCGCTGTTCTCGAGTTGTATTAGCCTCTCCGAAAACATGTATAAAAAGCACT<br>CGACCTATTCATATTATACAACATCAAAATCCCACCAATCAATCAAAAAACAGA<br>GAGACACATCTAAAAGCCCCCCCCCCCCCCCCCCCTTACAAAAATGGTGGAGC<br>GACGTTCATCCAACAAGGCGGCGGCGGCGGAAACCGAAACCGCCGTCGTCAAAC<br>GGTTCCCGTCCTCAAAGCCGCCGTTCACACTCGCCGACCTCAAGAAGGCCATTCC<br>GCCGCACTGTTTCAAAAGATCCATCCCACGCTCCTTCTCCTACTTGGTTTTCGACCT<br>CATCGTCGCCGCCGTCTTCTACCACATCGCCGCCACTTATTTCCCCCTCATCCCCA<br>AACCTCTCTCCTACGTGGCGTGGCCGGCCTACTGGTTCGTCCAGGGCAGCGTCCTG<br>ACCGGTGTGTGGGTCATCGGCCACGAGTGCGGCCACCACGCATTTAGCGAGCACC<br>AGTGGCTCGACGACCTGGTCGGGTTCATCCTCCACTCGGCCCTCCTCACCCCTTAC<br>TTCTCGTGGAAGATCAGCCACCGCCGCCACCACGCCAACACCTGCTCCCTCGAGC<br>GCGACGAGGTATATACTGTTGGGAACCGGACAAGAAGCAATATTCAGGGTTTATC<br>GATCTGGCTGAGTATACATGGACAAACTATCCATCAACATCCAAGATAAAAACA<br>GAGCTATAACTTTCAACTTCCGTGTGCAAAAATAGGGAGATGAATAAAATTTCAG<br>ATAATTTAAACAAATAAATTATCTCCAAAATGAATAATATTGGTCTCAAGTCCAA<br>TCCAACCTGGAGAACTGGATCTTTTTACAAAGACGTTTTTGCATTAGTCTTTTTTTC<br>TTTCTCCGATGTGATATTTGAGACTTAATTATTCACATTTTTCAACAGGTGTACATT<br>CCGAGGAAGAAGTCTCAGTTGCGGTGGTGGTACTCGAGCTACCTGAACAACCCGC

	CCGGCAGACTTTTGGCGCTGGCTTACACCATCCTACTCGGCTGGCCTTCCTACTTG<br>ACTTTCAACCTATCCGGAAGGGAATACAACGGTTTGGCCTGTCATTTCTACCCGAT<br>GTCCCCTATTTACAGCGACCGGGAGCGGGCCGAGGTATTCGCCTCCGATGTTGGC<br>TTGCTTGCTGTGTGCTTCGCGCTGTACAAACTTATCCTCGTCAAGGGAATGGCGTG<br>GGTCTTTTGCGTCTACGGGGCTCCGGTCATGGTGGTGAACGGATTCTTCATAACCA<br>TCACGTACTTGCACCACACTCATCTTGCGGTCCCGCGGTACAATTCCTCGGAATGG<br>GATTGGTTGAGGGGAGCCCTAGCAACGATGGATAGAGACTTTGGGTTATTGAACA<br>GGGTGTTCCATAACGTTACAGATACTCATGTGACGCATCATCTGATCTCGACCATC<br>CCTCACTACCATGCCATGGAAGCCAACAACGCGATTAGGCCCGTGTTGGGGGACT<br>ACTACCACATCGACAGGACGCCTGTTGTTAAGGCGTTGTGGAGGGAGGCTAAGC<br>AGTGTGTTTACATCGAGGCGGACGAAGGTGAAAATAACAACGGCGTGTTCTGGTT<br>CAATACCAAGCTCTAA
FAD2f-1	ATGGTGTCCTCCGGCAAAACCATGAGCAACAAAACCACCACGAAGCGGCCGCCG<br>GTGTCAAAACCGCCGTTCACTCTGGCCGACATCAAGAGAGCCGTACCGCCACACT<br>GCTTCAAAAGATCCCTCGTCAAATCATTCGCCTACCTAGCCTACGACCTCACCGTC<br>ATCACAATCCTCTACCACATCGCCAACACATACTTCCACCTCCTCCCCAAACCTCT<br>CTCCTACGTGGCGTGGCCCGTCTATTGGGCTGCCCAATGCTGCTTTTTCGTCGCTTT<br>ATGGATGGTGGGCCACGACTGCGGCCATCATTCATTCAGCGACTATCAGTGGGTG<br>GACGACACCGTTGGATTCGTCGTCCACTCGTTCCTCCTCGCCCCTTACTTCTCGTGG<br>AAGCATAGCCACCGCCGCCACCACGCCAACTCCGGATCTCTAGAACGCGACGAG<br>TCGTTCGTCCCGAAAACCAAAGACAATATCACCTGGCACTTCAAGTACCTCGACC<br>ACCTACCGGGGCGGATCTTCTACGTGGTCTTCACTCTAACCCTCGGATGGCCGCTC<br>TACTTGATGTTCAACATCACCGGAAGGCCGTACAAGGACGGATTCGCCAGTCATT<br>TCTACCCCATGTCGCCTATCTACGAGGATCACGAGCGGTTCGGAATATTCCTCTCT<br>GATGTTGGCATGCTCGCTATGTGGTTCACACTCTACAAGCTTTCCGTGGCGTATGG<br>AGTCGGTTGGGTTCTTTGTGTGTATTTTATTCCGCTGGTTTTACAAAATGCGTTGTTT<br>GTCACGATCACTTACTTACACCACACGCATCTGAACTTGCCTCACTATGACTCTTC<br>GGGGTGGGACTGGATGAGGGGGTCGTTGACGACTGTGGATAGGGACTATGGGTTT<br>TTGAACAAGGTGTTGCATAACGTGACAGATACTCACGTGGCGCACCATCTGTTCA<br>CCCATATGCCTCATTACCACCAATCGGAAGCGACCAAGGCGTTCATTCCGGTTTTG<br>GGGGAATACTACCAGGTTGATCCGACTCCGTTCTACAAGGCGTTGTGGAGGGAGA<br>TGAAGCACTGTGTTTATATCGAGCAAGATGAGGATGCGGATTCTGATAATAACAA<br>AAAAGGCGTGTACTGGTACAAAACCAAGCTCTAA
FAD2f-2	ATGGTATCCTCCGACAGAACCACGAACAACAAAACCACCACGAAGCGGCCGCCG<br>CTGTCAAAACCGCCGTTCACCCTCGCCGACATCAAGAGAGCCGTACCGCCGCACT<br>GCTTCTAGATCCCTCGTCAAATCATTCGCCTACCTAGCCTACGACCTCACCGCATC<br>ACCTTCCTCTACCACATCGCCAACACATACTTCCATCTCCTCCTCAAACCTCTCTC<br>CTACGTGGCGTGGCCCCTCTACTGGGCCGCTCAATGTTGCTTCTTCGTCGCTTTATG<br>GATGGTGGGTCACGATTGTGGCCACCATTCGTTCAGCGACTATCAATGGATTGAC<br>GACACTGTCGGTTTCATCATCCACTCGTTCCTCCTTGCTCCTTATTTCTCCTGGAAG<br>CACAGCCACCGCCGCCATCATGCCAACTCCGGATCTCTAGAACGCGACGAGTCGT<br>TCGTCCCGAAAACCAAAGACAATATCGCCTGGCACTTCAAGTACCTCGACCACCT<br>ACCGGGGCGGATCTTCTACGTCGTTTTCACTCTGACCCTCGGGTGGCCACTATACT<br>TGATGTTCAACATCACTGGAAGGCCGTACAAGGATGGATTCGCCAGCCATTTCTA<br>CCCCATGTCGCCGATCTATGAGGATCACGAGCGGTTCGGGATATTCCTCTCCGAC<br>GTTGGAATGCTCGCCATGTGGTTCACGCTCTACAAGCTCTCAGTGGCGTATGGAGT<br>CGGTTGGGTTCTTTGTGTGTATTTTATTCCGTTGGTTTTACAAAATGCTTTGTTTGTT<br>ACGATCACTTACTTGCACCACACGCATTTGAACTTACCTCACTATGATTCTTCGGG<br>GTGGGACTGGATGAGCGGGTCGCTGACGACTGTGGATAGAGACTATGGGTTTTTG<br>AACAAGGTGTTGTATAACGTGACAGATACTCATGTGGCGCACCATTTGTTCACAC<br>ATATGCCTCTTACCATCAATCGGAAGCGACCAAGGCGTTCATTCCGGTTCTGGGG<br>GAGTACTACCAGGCTGATCCGACATCGTTCTACAAGGCGTTGTGGAGGGAGATGA

	AGCATTGTGTTTATGTCGAGCAGGATGAGGATGCGGATTCCGATCAGAACAAAAG<br>AGGAGTGTACTGGTACAAAACCAAGCTGTGA
FAD2g-1	ATGAACAACAGAACCACCACGAAGCTGCTGCCGCTGTCAAAACCGCCGTTCACT<br>CTGGCCGACATCAAGAGAGCCGTACCGCCACACTGTTTCAAAAGATCCCTCGTCA<br>AATCATTCGCCTACCTAGCCTACGACATCACCGTCATCACAATCCTCTACCACATC<br>GCCAACACATACTTCTACCTCCTCCCCAAACCTCTCTCCTACGTGGCGTGGCCCGT<br>CTATTGGGCCGCCCAAAGCTGCTTCTTCGTCGCTGTATGGATGGTGGGTCACGATT<br>GCGGCCACCATTCATTCAGCGACTACCAGTGGGTGGACGACACTGTCGGATTCGT<br>CGTCCATTCGTTCCTCCTCACCCCTTACTTCTCGTGGAAGCACACCCACCGCAGCC<br>ACCACGCCAACAACGGATCTCTAGAACGCGACGAATCTTTCGTCCCCAAGACCA<br>AAGACGAAGTCAGGTGGCACTTCAAGTACCTCGACCACCTACCGGGGCGAATCTT<br>CTACGTGTTCTTTACTCTAACCCTAGGATGGCCTCTCTACTTGATGTTCAACATCAC<br>CGGAAGGCCGTACAAGGATGGATTCGCTAGCCATTTCTACCCCATGTCGCCTATG<br>TACGAGGACCATGAGCGGTTTGGAGTAGTCCTGTCCGACATGGGAATGCTCGCTA<br>TGTGGTTTACGCTCTACAAGCTCTCGGTGGCGTTCGGAGTCACTTGGGTTCTCTGT<br>GTGTACTTTATTCCACTAGTTTTACAAAACGCGTTGTTTGTTACGATCACTTACTTG<br>CACCACACGCATCCAAACGTGCCTCGTTATGACTCTTCGGGGTGGGGTTGGATGA<br>GGGGGTCGTTGGTGACCGTGGATAGAGACTATGGGTTTTTGAATAAAGTGTTCCA<br>TAACGTGACGGATACTCACGTGGCGCACCATCTGTTCACGCATATGCCTCATTACC<br>ACCAATTGGAAGCTACCAAGGCGTTCATCCCGATTTTGGGAGAGTACTACCAGGC<br>TGATCCAACACCGTTCTATAAAGCATTGTGGAGAGAAATGAAGCACTGTGTTTAT<br>GTCGAACAAGACAAAGATGCAAATGTCGATCAGAACAAAAGAGGTGTGTATTGG<br>TACAAAACAAAGTCGTGA
FAD2g-2	ATGGTATCCTCCGACAAAACCATGAACAACAAAACCACCACGAAGCTGCTGCCG<br>CTGTCAAAACCGCCGTTCACTCTCGCCGACATCAAGAGAGCCGTACCGCCACACT<br>GCTTCAAAAGATCCCTCGTCAAATCATTCGCCTACCTAGCCTACGACATCACAGT<br>CATCACAATCCTCTACCACATCGCCAACACATACTTCCACCTCCTCCCCAAACCTC<br>TCTCCTACGTGGTGTGGCCCCTCTACTGGGCTGCCCAATGTTGCTTCTTCGTCGCGG<br>TATGGATGGTGGCCCATGACTGTGGCCACCATTCATTCAGCGACTATCAATGGGT<br>CGACGACACCGTCGGTTTCATCATCCACTCGTTCCTCCTCGCCCCTTACTTCTCATG<br>GAAGCACACCCACCGCAGCCACCACGCCAACAATGGATCTCTAGAACGCGACGA<br>GTCTTTCGTCCCCAAGACCAAAGACGAAATCAGGTGGCACTTCAAGTACCTCGAC<br>CACCTACCGGGGAGAATCTTTTACGTGTTCTTCACTCTAACCCTAGGATGGCCTCT<br>CTATTTGATGTTCAACATCACCGGAAGGCCGTACAAGGACGGATTCGCCAGCCAT<br>TTCTACCCCATGTCGCCTATGTACGAGGACCACGAGCGGTTCGGAGTAGTCCTCTC<br>TGACATGGGAATGCTCGCCATGTGGTTCACGCTCTACAAGCTCTCAATGGCGTAC<br>GGAGTCAGTTGGGTTCTCTGTGTGTACTTTATTCCACTGGTTTTACAAAATGCGTTG<br>TTTGTCACGATCACTTACTTGCACCACACGCATCCGAACTTGCCTCGTTATGACTC<br>TTCGGGGTGGGGTTGGATGAGGGGGTCGTTGGTAACTGTGGATAGAGACTATGGG<br>TTTTTGAACAAAGTGTTGCATAACGTGACAGATACTCACGTGGCGCACCATCTGTT<br>CACGCATATGCCTCATTACCACCAATTGGAAGCTACCAAGGCGTTCATTCCGGTTT<br>TGGGGGAGTACTACCAAGCTGATGCAACGCCGTTCTACAAGGCATTGTGGAGGG<br>AGATGAAACACTGTGTTTATGTTGAGCAAGATGAGGATGCAGATTCCGACCAAA<br>ACAAAAGAGGTGTGTACTGGTACAAATCCAAGCCGTGA
FAD2h	ATGGTATCCAACAAAACCATAAACCGGCCGCCGTCCTCAAAACCGCCGTTCATCC<br>TCTCCGACATCAAGAAAGCAATCCCACCACACTGCTTCCGCAAATCCCTCCTCCG<br>ATCATTCTCCTACGTAGCCTACGACCTCGCCGTCATCGCAATCCTCTACCACATCG<br>CCACGTCATACTTCCACCTCCTCCCCAAACCTCTCTCCTACGTGGCCTGGCCGGCA<br>TACTGGGCCGCCCAGGGATCCCACTTCATCGCCGTCTGGGTCCTGGCCCACGAGT<br>GCGGCCACCACGCCTTCAGCGACTACCAGTGGCTAGACGACGTCGTCGGATTCGT<br>CCTCCACTCCGCTCTCCTCTCGCCGTACTTCTCGTGGAAACACAGCCACCGCCGCC<br>ACCACTCCAACTCGGCATCTCTAGAACGCGACGAGCTGTACATCCCGAAGAAGA<br>AATCGGAGATTAGCTGGCATTACAAGTACCTCGACAACCCGCCGGGTCATCTTTT

	CTACTTGGTGTTCACTCTCACTCTCGGATGGCCTCTCTACGTGATGTTCAACGTCTC<br>CGGCAGGGAATACGACGACGGATTCGCTAGCCATCTGTATCCTTTCTCCCCGATCT<br>ACAACGAACGGGAGAGATTCGGGATACTTTTGTCGGACGCTGGATGCTTGCCAC<br>TTGGTTTGGTCTGTACAAGCTGTCCATGGTCAACGGACTGAGTTGGGTTGTTTGTG<br>TGTACGGGGTCCCGCTATTGGTGATGAATGGATTGCTTGTGACGATCACTTACTTG<br>CATCACACCCATCTTTCGCTCCCGCATTATGACTCGTCGGAGTGGGAGTGGATGA<br>GAGGTGCGCTAGCGACTGTGGATAGAGACTACGGGTTCCCGCTGAATAAGGTGAT<br>GCATCATATTACGGATACTCACGTGGTGCACCATTTGTTCTCGATGATTCCTCATT<br>ACCATGCGACGGAGGCTACAAATGCGATTAGGCCGATTTGGGTGAATATTATCA<br>AGTTGATCCGACTCCGTTTGTCAAGGCGTTGTGGAGGGAGATGACGCACTGTGTTT<br>ACGTTGAGGCCGATGAGAAGAAACGAGGTGTGTTCTGGTATAAAACCAAGCTAT<br>GA
FAD3a	ATGAGCCCTCCAAACTCAATGAGTCCCGCCACCAACGGCAGCACCAATGGTGTG<br>GCTATCAATGGGGCGAAGAAGCTACTCGATTTCGACCCGAGTGCTGCTCCCCCTT<br>TCAAGATTGCAGACATCCGTGCTGCAATCCCGCCGCATTGTTGGGTGAAGAACCC<br>CTGGAGGTCACTCAGCTACGTCCTGAGAGACCTCCTGGTCATCCTCAGCTTCGCCG<br>TTGCGGCGACAAAGCTGGACAGCTGGACTGTCTGGCCTCTCTACTGGATTGCTCA<br>AGGAACCATGTTCTGGGCAGTCTTTGTTCTTGGACATGATTGGTAATTTCACATGA<br>TCTTTCTGGTAATGTGGGTTTTCTTTTCTTATTGAAAAAGATTAAAACTTTTTATCTG<br>GGCTGTTGCATGCAGTGGCCATGGGAGCTTCTCAGACAGTTGGTTGTTGAACAAC<br>GTGATGGGACATATACTCCATTCCTCAATCCTCGTACCTTACCATGGATGGTATTG<br>TAACTATTGTTCGATATTCGATTATGATTACTGTTCTTTCAGATGAAGAATCTGTAC<br>CCTAATTGTTTTTTGTTACCAGGAGAATTAGCCACAAGACCCATCACCAGAATCA<br>CGGCAATGTGGAGAAAGATGAATCCTGGGTTCCAGTAAGTTGACATGCAGTTTGC<br>TCTAAAATGCAGAGTCCTCTGTTTTTTGTGTGTTCTTGTGCTTTAATGACGATGATA<br>ATGAAATTGAAATTTGTAATAGCTGCCGGAGAAGGTGTACAAGAGCTTGGATACC<br>GGCACCAAGTTCATGAGGTTCACCATCCCTCTCCCAATGTTTGCGTATCCTATCTA<br>CTTGGTAAGTAAACAGACTGACTCCAAAGTAGGAACTAATGACAATTTTGGACCC<br>GACCTGGTTTGGTTGACTCGGGTCGATATGTTTCGGGTGGGTAATTACCCGATCTG<br>GCGATGGGTGTGCGGCGGACATTGTCTTGCTCGTGGTCCACCCCGCTCCCAACCC<br>GCCCCATTCTTGACGAAAAAGATTTCGGAATATGTATCAACAGAAAAATCTAGTT<br>TTTATGTTACTAGTTTTCTGTATTTCCATGTTTTTTCCTCAATTCTAGCCGGAATTTG<br>AATTCAAACTGAAATCGGGTAATTCCGTCCATAACAAAACGGAATTGGGCAGCC<br>GTAATTAGTTGAAACTAGACCTCAATTTTGGCGGAATTGGACCCGGCCATTTTTTA<br>CGTTTGCAAAACGGAAAACGTTTTTCTTTTGTAAAGCGCAAAATGAAAAACGTAT<br>CTAGTGGAATTATTGGACCCATCTAGAATGGGTCCAATTCCACCCCAATTTCGGCT<br>CCAATTCATGCCCGGAAAACACTACTGTCATGCATTTTAATCTTGTATGGTTTTAC<br>CCCAATGGATGCAGCGATGGATCCGGACGATTTTTAAAATATTATCGGGTTAAAT<br>TTAAAAATATCTTAAAACTATAAGAAAAAAATAACCAATTTTAAAGAATAAAAG<br>AACTGGACACATATGACGGGTGTCGTGGATGGATGTACTTGTCCCGCTCTATTAA<br>AGGCTGATAATATACAGGTCAACGGTGAATGAAGGTTAGATGCGCTATTGGATTT<br>GAATCCGATATGAAATGATAATTTTGGACACGATCTGTTTTGGGTGGGTAATATTT<br>GATCTAGGGATGGCTCGTGCTCCAAACCGCACCAAAACCGCCTAATTCTCGACCA<br>AAAAGATTTTATGAATACATATCAACAGAAAAATCTAGTTTTCATGTTACTAGTTT<br>TATGTACAACAATATTAGGTGTCGTTTTCCCAGCCTTTTTCTTCAATTCCGGCCGGA<br>ATTCGCATTCAAACCGGAATTGGATGGAATCGGTATACCTCGTCACGGATGCATT<br>GTCAATTCCTAGTTAGTTTCATGGTTTTGAAACCAATCAATCTATTCTATATGGTTT<br>TGATTAACAGTGGAGGAGAAGTCCGGGGAAGAAAGGGTCGCATTTCAACCCATA<br>CAGTGACCTGTTCGCACCGAACGAGAGGACATCGGTCATGATTTCGACATTGTGC<br>TGGACAGCCATGGCCTTACTCCTCTGCTACTCATCGTTCATCTACGGCTTCCTTCCG<br>GTCTTCAAAATCTACGGCGTCCCTTATCTAATATTCGTGGCGTGGCTCGACATGGT<br>GACCTACCTTCACCACCACGGGTACGAGCAGAAGCTGCCGTGGTACAGAGGCAA<br>AGAGTGGAGCTACCTACGTGGAGGGCTGACGACCGTCGATCGAGATTACGGGT

	CATCAACAACATCCACCATGACATTGGCACCCATGTTATTCACCATCTCTTCCCTC<br>AAATGCCACACTATCACCTAGTCGAAGCGGTAAGGAGGTCTTGATTATTAACTTA<br>ATGTTTTTGTTGTTATAATTTGAGTCCGATTCTGGAGTCAGGGGATTTCCTTCTTGG<br>ATCCGATCCAGGATCAAGCTGGTCCCTTGAATTTCTATATGATCTTATATTAATTA<br>AGGATAATGTGGTCATATGTTTTTAAATATTTTTGTTTACCATCATTTTCGATCACC<br>GGAAAATGTCCTGAGCAGTTTTCCGGTCACTTTAACCTCCATTGACAAATTTTTTC<br>ACCCACATGATCACCCTAGCCGGGTTTACGTTTATTGAAAATTTTTATTTTTTTGAA<br>TTTTTTTTCGATGACCAACTGTACAACTTTGTATTGAAAGTTGTATGGATCATACA<br>AATGTGTATGTACAAAAGTATATTCTAAGTACTATACTAAGCATTACTTAGTATTA<br>CGTTTCTACAAACCTATAGAGAAATGCATACAATTTTGTATAGAACTTAGTATAC<br>ACGTAGCTGTGAAATGTCAATTTCCCTCCGTATTTCAGAGACAAGACATGATTTT<br>TAGACTGGCAGATTTTTTTTATCGGATAGATTTCTCCAACTTCAGATTCGGACTGG<br>ATTATTAACTATATTATTCATCAACTCTGACGTTTGATGTTGCATGTGACAGACTC<br>AGGCAGCGAAGCACGTGCTGGGGAAGTACTACAGAGAACCGAAGAAATCAGGG<br>CCTTTCCCATTCCACTTGTTTGGGTACTTGGTGAGGAGCCTGGGCGAGGATCACTA<br>CGTTAGCGATACAGGCGACGTCGTTTTCTATCAATCTGACCCACATATTCCCAAGT<br>TCCCTACCAGTGCCACCACCAAGTCCAAATCTAGCTGA
FAD3b	ATGAGCCCTCCAAACTCAATGAGTCCCACCACCAACGGCAATGGTGTGGCTATGA<br>ATGGGGCGAAGAAGCAGCTCGATTTCGACCCGAGTGCTGCCCCCCCTTTCAAGAT<br>TGCAGACATCCGTGCTGCAATTCCGCCGCATTGCTGGGTGAAGAACCCCTGGAGG<br>TCGCTCAGCTACGTCCTGAGAGACCTCCTTGTCATCCTCAGCTTCGCCGTTGCGGC<br>GGCGAAGCTGGACAGCTGGACTTTCTGGCCTCTTTACTGGGTTGCTCAAGGAACC<br>ATGTTCTGGGCAGTCTTTGTTCTTGGACATGATTGGTAAACTAATTTCACATTTTCT<br>TTCTGGTAATGTGGGTTTTATTGAAAAAGATTCAAACTTTTTATCTGGGTTGTTGCA<br>TGCAGTGGCCATGGGAGCTTCTCAGACAGCTGGTTGTTGAACAATGTGATGGGAC<br>ATATACTCCATTCCTCAATCCTCGTACCTTACCATGGATGGTATTGTAACTATTGTT<br>CAATATTAGATTATTGCTAGTTCTTCAGCTGAAGAATCCAAACCCTAATTTTCTTTT<br>TCTGAATATTGACCAGGAGAATTAGCCACAAGACCCATCACCAGAATCACGGCA<br>ATGTGGAGAAAGATGAATCCTGGGTTCCTGTAAGTTGACATGCAGTTTGCTGTAA<br>AAATGCAGAGTGCTCTGTTTTTTTGTGTTCTTGTGCTTTAATGGTGATAATAATGAA<br>ATTGTTGAAATGTAACAGCTACCGGAGAAAGTGTACAAGAGCTTGGATACCAGC<br>ACTAAGTTCATGAGGTTCACCATTCCTCTCCCAATGTTTGCTTATCCTATCTACTTG<br>GTAAGTAAAGAGACTGATAAGACTCCAAAGTAGGAATTAATGACAATTTTGGAC<br>CCGAGCTTCCGACTCGGGTCGATTTATTTCGGGTGGGTACTTACCCGATGCGGCGG<br>ACATTGTTTTGCTCGTGGTCCACCTCGCTCCCAACCCGCCCCATTCTTGACGAAAA<br>AGATTACGGAATATGTATCAACAGAAATATCTAGTTTTTATGTTACTAGTTTTCTGT<br>ATTTCCGTGTTTTTCCCCTCAATTCCGTCAGAAATTTGAATTCAAACTGACATTGGG<br>TAATTCTGTCCATAAGAGAACGGAATTGGGAAGCCGTAATTAGTTGGAATTAGAC<br>CTCGATTTCGGCGGAATTGGACCCGGCCATTTTTCGCGTCCGGAAAAGCGTTTCCA<br>GTGGAGTTAGACGCCCATCTAGAATGGGTCCAATTCCACCCCAATTTCGGGGCTA<br>CCTATTTTTAGTGTACATGCATTTTAGATCTTTTACGGTCTTACCCCGATGGATATT<br>CTTGTCGCGCTCTATTAAAAACTGACAATATACAGGTTGATTGTGAATGAAGATT<br>AGGTGCTGCACGCTCTTCGTGTTCCAACCCGCACCCAAACCGCCCCATTCTCGAC<br>CGAAAAGATTTTATGAATACATATCTACAGAAAAATCTAGTTGTCATGTCACTAG<br>TTTAATGTACAACAGTATAGGTGTCGTTTTCCGCGTCTTTTTCTTCAATTCCGGCTG<br>GAATTCGCATTCAAACAGGAATTGGATGGAATTGGTCCCGGATGCATAGTCATTT<br>CCCAGGCAGTTTCATGGTTTTATAACCAATCAATCTAATCTTATGCTTTTGATAAA<br>CAGTGGACGAGAAGTCCGGGGAAGAAAGGGTCGCATTTCAACCCATACAGCGAC<br>CTATTCGCACCAAACGAGAGGGCAGCGGTCTTGATTTCAACATTGTGCTGGACAG<br>CCATGGCCTTACTCCTCTGCTACTCATCGTTCATATACGGCTTCGCTCCGGTCCTCA<br>AAATCTACGGCGTACCTTATCTGATATTCGTGGCATGGCTCGACATGGTGACCTAC<br>CTTCATCACCACGGGTACGAGCAGAAGCTGCCGTGGTACAGAGGCAAAGAATGG

	AGCTACCTACGTGGAGGGCTGACGACCGTTGATCGAGATTACGGGGTCATCAACA<br>ACATCCACCATGACATTGGCACCCATGTCATTCACCATCTCTTCCCTCAAATGCCA<br>CACTATCACCTTGTGGAAGCGGTAAACAATTTGATTATTAATTTACTGTTTTTGTTG<br>TTATAATTTGAGTCGGGAGATTTCCTTCCTAAATCCGATCCCTGGTCAATCTTGGC<br>CCTTGAATCTTCATATAATCTAAAAATCTAGATTAATCAGGAACAATATGATCAT<br>GTTGTTTAAACTAATTTTGTTGGACCATAACCTACCGCCAACTGATGGACCACCGT<br>CTCTGGTTACCGGACCCATCATTTCCGGTTACCAAGAAGTTTCTCGATCAGTTTTC<br>CGGTTACTTTGACCTGCGTTGAGGAAAATTCTTTCACCCACGTAAACACTGTCGTC<br>AACTTTACGTTTCTGGAAAGTTTTTCCGATGATTGGCCGTACAATTTTGTACGAAG<br>AGTTGTACGGATCATATAAATGTGTATAAGTTTCTAGAAATCCGTACTGAAATAT<br>ATACATATTTGACTTTTGTATAAAGTGTAATACTAAATACTATACTAAGTGCTGTA<br>CTCAGTATGATACTTAGTACACACATTTGTATGACTATGAAATGTCAATTTTGCCC<br>TTATATTCTCAGCCGTTAGATCTAAGACACAGTTTTTATACGGCTGAAATTTGTGG<br>GGGCTTTGTAGATCGGATCCATAAGTCATTTCTTGGCTCAAGATTCGGACTCGATT<br>ATTAACTATATTATTCATCAACTCTGACGTTTGATGTTGCATGTGACAGACTCAGG<br>CAGCGAAGCACGTGCTGGGGAAGTACTACAGAGAGCCGAAGAAATCAGGGCCTT<br>TCCCATTCCACTTGTTTGGGTACTTGGTAAGGAGCCTGGGCGAGGATCACTACGTT<br>AGCGACACAGGCGACGTCGTTTTCTATCAGTCTGACCCACATATTCCCAAGTTCCG<br>TACCAGCAGTGCCACCACCAAGTCCAAATCCAGCTGA
FAD3c-1	ATGAGCCCTCCAAACTCCATGGGAATGGAAGCTGCTCATCCTACCGGCAACGGC<br>AATGGCGTCGCAGTCATGAACGGCGCCTCCGCTAACAAACCCGATTTCGATCCCA<br>GCGAGGCTCCTCCCTTCAAGATTGCCGACATCCGAGCCGCCATCCCCCCGCATTG<br>CTGGGTGAAGAATCCATGGAGGTCTCTTAGCTACGTCCTTAGGGACGCCGTCGTC<br>ATTCTCGCATTCGCAGCTGCCGCCCTCAAGCTCGACCTCTGGGCTGTTTGGCCGCT<br>CTACTGGATCGCTCAGGGCACCATGTTTTGGGCTGTCTTCGTTCTCGGCCACGATT<br>GGTAAGAGGAGGAGGTTTTTTTTTAAAATTTCAATCCGATGCTGTTTTGTTATATTA<br>TTGGTTGATATGATATATAAGATTAACCCTAGAGAAAAGTGAAAAGCATCCGCCA<br>TTGTTGAATGAATTGGAATTGGATGGTGCAGCGGCCATGGGAGTTTCTCGGATAG<br>CTGGTGGCTGAACAATGTGGTGGGGCATATTCTGCATTCTGCAATCCTTGTGCCTT<br>ACCATGGATGGTAAGAAAAGAATATGAACAGATCCTTTATCCTTACAACTGTAAA<br>ATGTAGGTCATTCAGCTGAACTAAAGTCACATTCTGTGTTGTTTAATTGATTGAAC<br>AGGAGAATTAGCCACAAAACACACCACCAGAATCATGGCAATGTTGAGAAAGAT<br>GAATCATGGGTTCCGGTGAGTTTTATTTCATTTTTTCTCTCGTCAATTGGCATCAGT<br>TTGTTCTGTTTCAATGCTTGATATGAATTGGGTGATATAATGCAGCTGCCGGAGAA<br>AGTATACAAGACCCTGGACACAAGCACCAAATTCATGAGGTTCACTATCCCTCTC<br>CCAATGTTTGCTTATCCTATCTACTTGGTAAGAACCACCCCACATTCTTACATCCTT<br>GCATGTTGTGTATTGGTTTCTGGTTGGATTTGGATTCAGATTTGGGGAATTGATTGT<br>GTGCCTTTGAAATGAATGAATGGTTACAGTGGACGAGGAGCCCAGGGAAGAAAG<br>GGTCCCATTTCAACCCCTACAGTGACCTGTTTGCCCCACAAGAAAGGAAATCAGT<br>CTTAATCTCTACCATCTCTTGGATTTCCATGGTCCTAATCCTCCTCTACGCCTCCTT<br>CCTTTTTGGTTTCCTCACTGTCTTCAAAGTCTATACCGTCCCTTACCTGGTAAAACT<br>ACTATCTCCAAATTCAACAATCTGTAAGCTTCATTTCATTTGCTTTGGCTCCTGAGT<br>TCCCTGTCAAGTTTCTCCTAGCAGTGTAACTTGTTATTTGATAACTTTGTGCCATCT<br>CACTATTGGCTGCATTTTACTATAATCAAACTGCTACATACGTTATTGCAACTTGTT<br>AATCCTTGGTACGAGGAATCCAATGAATAGGATGGTGATTAGCACAAACAAGGC<br>TGAAACTTTGAGCAATTTTGCAGATATTTGTGGCGTGGCTGGACATGGTGACATAC<br>CTGCACCACCACGGGCACGAAGAGAAGCTGCCGTGGTACAGAGGTCAAGAGTGG<br>AGCTACCTACGTGGAGGGCTGACAACCGTAGATAGAGATTACGGATTATCAAC<br>AACATCCACCACGACATTGGCACTCACGTGATTCACCATCTGTTCCCTCAAATCCC<br>TCACTACCATCTCGTAGAAGCGGTACAGTAGTAGTAAACAAAACCCCATATTATT<br>ATTACTTACTGCAGTGATTTCTGACTCTATATTATTAAATATCACTTCTGGGTTTTT<br>GGCCGCGGGGGGGGGGGGGGGGGGTTGTTTGGATCTTTCGAACTTATAATAATA<br>TGATATTATTATTAAAATGGTGGCAGACAAAGGCAGCAAAGTCGGTGCTTGGGAA

	GTACTACAGAGAGCCAAAGAAATCAGGACCATTCCCATTCCACTTGTTCGACAAC<br>TTAGTGAGAAGCCTTGGCGAAGATCACTATGTTAGTGATGCAGGGGATGTCGTGT<br>TCTATCAGTCTGACCCAGAAATCTTCAAGTTTTCCAAGTCCAAGTCAGCCTAA
FAD3c-2	ATGGGAATGGAAGCTGCTCATCCTACCGGCAACGGCAATGGCGTCGCCGTCATGA<br>ATGGCTCCGCCGCTAACAAACCCGATTTCGATCCCAGCGAGGCTCCTCCGTTCAA<br>GATTGCCGACATCCGAGCCGCCATCCCTCCGCATTGCTGGGTGAAGAATCCATGG<br>AGGTCACTCAGTTACGTCCTTAGGGATGCCGTTGTCATTCTCGCATTCGCCGCTGC<br>CGCACTCAAGCTCGACCTCTGGGCTGTTTGGCCGCTCTACTGGATCGCTCAGGGC<br>ACCATGTTTTGGGCTGTCTTCGTTCTCGGCCACGATTGGTAAGAGGAGGAGTTGTT<br>TTTTTTAAATATTTATCCGATGCTGTTTTGTTATATTATTGGTTGATATGTAAGATTA<br>ACCCTAGAAAAAAGTGGAAAACGTCCGCCATTGTTGAATTGAATTGGAATTGGAT<br>GGTGCAGCGGCCATGGGAGTTTCTCGGATAGCTGGTTGCTGAACAATGTGGTGGG<br>GCATATCCTGCATTCTGCAATCCTTGTACCTTACCATGGATGGTAAGAAAAGAAT<br>ATGAACAGATCCTTTATCCTTGAAACGGTAAAATGTAGGTTATTCAGCTGAAACA<br>AAAGTGACATCCTGTGTTGTTCTTTATTGATTGAACAGGAGAATTAGTCACAAAA<br>CTCATCACCAGAATCATGGCAATGTTGAGAAAGATGAATCATGGGTTCCGGTGGG<br>TTTTATTTCTCTTCCTTACTTGTCAATTTGCCATCATTTGTCTGTTTCACTGATTGAC<br>ATGAATTGGGTGGGAAAATGCAGCTGCCAGAGAAAGTGTACAAGACTCTGGACA<br>CAAGCACAAAATTCATGAGGTTCACTATCCCTCTCCCAATGTTTGCTTATCCTATC<br>TACTTGGTAAGAACCACCCTACATTCTCACATCCTTGCATGTTGTGTATTGGTTTCT<br>GGTTGGATTTGGATTCAGATTTGGGAAATTGATTGTGTGCATATGAAATGAATGA<br>ATGGTTACAGTGGACAAGAAGCCCAGGGAAGAAAGGGTCCCATTTCAATCCCTA<br>CAGTGACCTGTTTGCCCCACAGGAAAGGAAATCAGTCTTAATCTCTACCATCTCTT<br>GGATTTCCATGGTCCTAATCCTCCTCTACGCCTCCTTCCTATTTGGCTTCCTCACAG<br>TCTTCAAAGTCTATACCGTCCCTTACCTGGTAAAAACTCCACCCTTGGTTTACTAG<br>CTTCAAATTCAACAATCTATCAGCTTCATTTCATTTGCTTTGGCTCCTAAGTTCCCT<br>CTTAAGTTTCTCGTAGCAGAGTAACTTGTCTATTTGTTAACTTTGTGCCATCTCCCT<br>ATTGGGTGCATTTCACTATAATCAAACTGCTACATGCATACTGGAATGTCCATATT<br>GTAATTTGCTATAATCCTTGGTACGAGAATCCAATGAATAGGATGGTGATTAGAA<br>CAAGAAAGACTGAAACTTTGAGCAAATTTTGCAGATATTTGTGGCGTGGCTGGAC<br>ATGGTGACATACCTGCACCACCACGGGCACGAAGAGAAGCTGCCGTGGTACAGA<br>GGCAAAGAGTGGAGCTACCTACGTGGAGGGCTGACAACCGTAGATAGAGATTAC<br>GGGATTATCAACAACATCCACCACGACATTGGCACTCACGTGATTCACCACCTCT<br>TCCCTCAAATCCCTCACTACCATCTCGTAGAAGCGGTACTGTAGTAAACAAAACC<br>CATATTATTATTACTTACTGCAGTGATTTCTGACTATGTTATTATTAAATATCAGTT<br>CTGGGTTTTGGCCTTTGGGGGGGTTGGTTTGGATCTTTGGCACTTATAATATTATTA<br>AAATGGTGGCAGACAAAGGCAGCAAAGTCGGTGCTTGGGAAGTACTACAGAGA<br>GCCAAAGAAATCAGGGCCATTCCCATTCCACTTGTTCGACAACTTAGTGAGAAGC<br>CTTGGCGAAGATCACTATGTTAGTGATGCAGGGGATGTCGTGTTCTATCAGTCTGA<br>CCCAGACATCTTCAAGTTTTCCAAGTCCAAGTCAGCCTAA
FAD3d-1	ATGGCGAGCTGGGTTCTATCAGAATGCGGCATAAAGCCTCTCCCTTCCGCCTTCCC<br>TAAGCCCAGAACCGGAGCGCTTTCCCGAAATACCCTCCCCAAGCTCAGGTATTTA<br>CCCCCGAGGAGCAATCTCTCCGCCGCTGCCGACGGCGGCGCCTCAGATCTAAGAC<br>TTAACCTACCTAAATTATCCTCCGTAGAGAAGAAAAAGCCGCTTTTGGGACAGGT<br>GAGAGTTACTGCTCCGTTCAAGGTCGCTCCGGTGAAGGAGGAAGACGGAGAAGG<br>AGAGTCCAATTTCGACCCAGGAGCGCCGCCGCCGTTCAATTTGGCCGACATTCGA<br>GCTGCTATACCCAAGCATTGCTGGGTTAAAGATCCATGGAGGTCAATGGCTTACG<br>TGGCGAGGGACGTGGCCGTTGTTCTCGGATTGGCAGCTGCCGCAGCTTATCTGAA<br>CAATTGGATCGTTTGGCCGCTGTATTGGGCGGCTCAGGGGACCATGTTCTGGGCTC<br>TCTTTGTTCTTGGCCATGACTGGTGAGTAAAGTTGTTAACTTTCTCTTTGGTCTTTTA<br>TGCTAATCTTTGTAATAATGGAAAATTGAAGAAAAATTCTCATGTTTGGCAGTGG<br>ACATGGGAGCTTTTCAAGCAATCACAAGCTGAACAGTGTAGTTGGACACATTCTC<br>CATTCTTCAATTCTTGTGCCTTACCATGGATGGTAACTAAAGCTCTCTGCTTTCCTC

	TGCCCATCTGTCATTTTGTTCTTCAATTTTGAAGCAATTTGAAACAAATTTTGTATC<br>TATGTCTTTACAGGAGAATTAGCCATAGGACGCATCATCAGAACCATGGACATGT<br>TGAGAACGATGAGTCATGGCATCCTGTGAGTCTTTGTTTCTAAATCTTATCTATAG<br>GATTGATTCAGATGCTGATGTACTAATTTCTGTGACTTTTTCTGTTTTGTTTCTGTTC<br>TTGAAGTTGTCTGAGAAGATATTCAGGAGCTTGGATGCTACAACTCGAAGTATGA<br>GGTTCACATTGCCTTTCCCCATGCTTGCTTATCCTTTCTATCTGGTGAGTTTGTATCT<br>ACAAGAAAGTTCCTTTACAAAATGTGGATTTAGTTAACAGAAGCTAAAAGGGTTC<br>TTTCTTCTTGTTCCGTAATGCAGTGGAACCGGAGTCCTGGAAAAAAGGGGTCTCAT<br>TTCGACCCGAGTAGTGATTTGTTTGTGGCGAGTGAGAAGAAAGATGTGATCACTT<br>CCACTGTTTGTTGGACAGCTATGGCTGCTTTGCTCGCAGGCTTGTCCTTTGTAATGG<br>GTCCAATTCAGATGATCAAGCTATACGGAATTCCATATTGGGTATAGACAACATC<br>TTTTGCCTTATCTTGCAAACTTGTTGTTTCAACTCTCAACCAACCACTCTGCCTCTT<br>GGATGTTTTTGTAGGGATTTGTTATGTGGTTGGATTTTGTAACTTACTTGCATCACC<br>ATGGTCACGACGATAAGCTTCCTTGGTACCGCGGCAAGGTAATCGATGTTCTTTCC<br>TCCTGTGTCTATCTATCTGGTCTTTCATGTTGAATCACTATATCCTAAAATCTTTTCA<br>ATGTATAATTATTGATTCAAACCAAATGAAAAGTCAATTGTTTTGAGATGTCTTTC<br>TAGACTTTCAGTGAGATTTTGTTGAACTTATGCTTGGAACTTGGAACAGGAATGGA<br>GTTACTTGAGAGGAGGGTTAACCACCATTGATAGAGACTATGGATGGATCAACA<br>ACATCCACCACGATATTGGAACCCATGTTATTCACCATCTCTTCCCACAAATCCCT<br>CACTACCACTTGATTGAAGCAGTAAGCAATAACTTTCCTTTTTCCCTTTAGTTCCAT<br>ATGCAATCTCTTCAATGGGGACACTGAGAAAAAGAAACAAACTTTGACCTGAAA<br>ACAAATATCATGATGTTTCAGACGGAAGCTGCAAAGCCAGTGCTTGGGAAGTACT<br>ACAAGGAACCAGCAAAATCAAAACCGTTCCCTTTCCACCTAATCGGAGACTTGAT<br>AAAGAGCTTGAAGAGAGACCATTATGTTAGTGACACCGGAGATGTCGTCTACTAT<br>CAAACTGACCCTGAACTCCAAAGACCCTCATCATCATCATGA
FAD3d-2	ATGGCGAGCTGGGTTCTATCAGAATGCGGCATAAAGCCACTCCCTTCCGCCTTCC<br>CCAAGCCCAGAACCGGAGCGCTTTCCCGGAATACCCTCCCCAAGCTCAGGTATTT<br>ACCCCCCAGGAGCAATCTCTCCGCCGCTGCCGACGGTGGCGCCTCAGATCTAAGC<br>CTTAACCTACCTAAATTATCCTCCGTAGAGAAGAAAAGGCCGCTTTTGGGGCAGG<br>TGAGAGTTACTGCTCCGTTCAAGGTCGCTCCGGTGAAGGAGGAAGACGGAGGAG<br>GAGAGTCCAATTTCGACCCAGGAGCGCCGCCGCCGTTCAATTTGGCCGACATTCG<br>AGCTGCTATACCTAAGCATTGCTGGGTTAAGGATCCATGGAGGTCAATGGCCTAT<br>GTGGCGAGAGACGTGGCTGTTGTTCTCGGATTGGCAGCTGCCGCAGCTTTTCTGAA<br>CAATTGGATTGTTTGGCCGCTGTATTGGGCGGCTCAGGGGACTATGTTCTGGGCTC<br>TCTTTGTTCTTGGCCATGACTGGTGAGTGAAAATTCTCAATTTTCGCTACTGTGATT<br>ACTAATCTGATGTAAAAAGTGGGTGGAAAGTTCTCATTTTTCCTTTTGCAGTGGAC<br>ATGGGAGCTTTTCAAGCAATCACAAGCTGAACAGTGTAGTTGGACACATTCTGCA<br>TTCTTCAATTCTTGTGCCTTACCATGGATGGTAAAAAGCTCTCTGCTTTCCTCTGCT<br>CATCCGCCATTTTGTTCTTCAAGTTTGAAGCAATTGAAAAAATAATTGTATCAATG<br>TCTTTACAGGAGAATTAGCCATAGGACGCATCATCAGAACCACGGCCACGTTGAG<br>AACGATGAGTCATGGCATCCTGTGAGTCTTTGTATCTAAATCTTAGCCATAGGGTT<br>GATTCAGATGCCGATGTACTAATTTCTGTGACTTTGTTTTGTTTCTGTTCTTGAAGTT<br>GTCTGAGAAGATATTCAGGAGCTTGGATGCTACAACTCGAAGTATGAGGTTCACA<br>TTGCCTTTCCCCATGCTTGCTTATCCTTTCTATCTGGTGAGTTTGTAGCTGCAAGTA<br>AGTTTCTTTACAAAATATGGATTTAGTTAACGAAGCTAAAAGGGTTATTTCTCCTA<br>ATGCAGTGGAACAGGAGTCCTGGGAAAAAGGGGTCTCATTTCGACCCGAGTAGC<br>GATTTGTTTGTGGCGAGTGAGAAGAAAGATGTGATCACTTCCACTGTTTGTTGGAC<br>AGCCATGGCTGCTTTGCTCGCAGGCTTGTCCTTTGTAATGGGTCCAATTCAGATGA<br>TCAAGCTCTACGGAATTCCATATTGGGTATAGACAACATCTTTTGCCTTATCTTAC<br>AGACTTGTTGTTTCAACTCTCAACCAACCACTCTGCCTCTTTGATGTTTTTGTAGGG<br>ATTTGTTATGTGGTTGGATTTCGTAACTTACTTGCATCACCATGGTCACGACGATA<br>AGCTTCCTTGGTACCGCGGCAAGGTAATCAATGTTCTTTGCTCCTGCGTCTATCTAT<br>CTAGTCTTTCATGTTTAATCACTATATCCTAAAATCTTTTCAATGTATAATTCTTGA

	TTCAAACCCGATGAAAAGTCAATTGCTTTGAGATGTCTTTCTAGGCTTTCAGTGAC<br>ATTTTGTTGAACTTGTGCTTGGAACTTGGAACAGGAATGGAATTACTTGAGAGGA<br>GGGCTAACCACCATTGATAGAGACTATGGATGGATCAACAACATCCACCACGAT<br>ATTGGAACCCATGTCATTCACCATCTCTTCCCACAAATCCCTCACTACCACTTGAT<br>TGAAGCAGTAAGCACTAACGTCCCATTTTCCCTTTAGTTCCATACGCAATCTCTTC<br>AATGGGGAAACTGAGAAAAAGAAACGAACTTTGACCTGAAAACAAATATCATG<br>ATATTTCAGACGGAAGCTGCAAAGCCAGTGCTCGGGAAGTACTACAAGGAACCG<br>GCGAAATCAAAGCCGCTCCCTTTCCACCTAATCGGAGACTTGATAAAGAGCTTGA<br>AGAGAGACCATTATGTTAGTGACACCGGAGATGTCGTGTACTATCAAACCGACCC<br>CGAACTCCAAAGACCCTCATCATCATCATGA
SAD2-1	ATGGCTCTCAAGCTCAACCCAGTCACCACCTTCCCTTCAACACGCTCCCTCAACA<br>ACTTCTCCTCCAGATCTCCTCGCACCTTTCTCATGGCTGCTTCCACTTTCAATTCCA<br>CCTCCACCAAGTAAGCACCTCCTCCTCCTCGGAATCTCCGCCGATTTCTTTTAAGC<br>GATTGATCGTAGATAAATTTGTCGGTTGCTTACCGTTCATCAAAATCTGCACGGTT<br>CGTTTCTTCTTCTGCGCCTAGATTGCATTATGTCATTGTTCGTTTTCCGATTTGACTG<br>ACCGACATAAATCAATTCCTTTGTGTTTCACGATTCTGGGTTTTGCGCTGTAATTGA<br>TTGTCAGTGTTTGCACAGGTTTCCCCTTCTCCTCCTCCGTCCATCAAATGCATGTTA<br>TTACCATTTCAATTTCAGCTTCCTTCTCTGAAATATCCGTCTCTGGGAAAATAAGTC<br>TCTGTATCTACTATCCTATCAGCTTGTTTAGGAGAGGTTCGATATTCGTTTACATAA<br>ACCAATTGGCTTACAGTCCTTGAACGTTCTAAATGTTGGTCGCGGTGATAATAGGT<br>TCTCAAAAGAGGTTTGTCTATGTTGTTTGGCAAAATCTTGTTTCTGTGAATCATGTT<br>TAAGGTCCTTGGAAGAATGACTAATGAGCTATGACATGATTACGACGTAGTAGTT<br>ATTGAACTGCTGATAATTCAATATAGGGGTAACTTTGTTGATTGTTTGGTCACAGG<br>GAGGCTGAGAAGCTAAAGAAGTCACATGGACCACCAAAAGAGGTGCATATGCA<br>AGTGACCCATTCCATGCCCCCACAGAAGCTGGAGATATTTAAGTCTCTGGAAGGT<br>TGGGCTGAGGATGTTCTATTACCGCACCTGAAGCCAGTTGAGAAATGCTGGCAGC<br>CACAGGATTTCCTGCCCGAACCTGAGTCGGATGGGTTCGAGGAGCAAGTGAAGG<br>AGCTCAGGGCAAGGGCCAAAGAACTGCCCGATGACTATTTTGTTGTGCTGGTTGG<br>GGATATGATCACCGAAGAAGCTCTGCCGACTTACCAGACAATGCTCAACACCCTT<br>GACGGGGTGAGGGACGAGACTGGAGCCAGCCTTACGCCGTGGGCAATCTGGACA<br>AGGGCGTGGACCGCTGAAGAGAATAGGCACGGTGACCTTCTCAACAAGTATCTA<br>TACCTCTCTGGAAGGGTGGACATGAGGCAAATTGAAAAGACCATTCAGTATCTCA<br>TCGGCTCTGGAATGGTATGTAATCACATACTTCATCCTTTTCTATTAATCTTTGCGT<br>GAACAAAATTCACTACACTGGTAGCAGCTGAAACTTTAGATGATTTTTTTACTGC<br>CTAGCTTCTATGAAACAAAACCACGTAAGTCAAATAGGGTTGACAATGAGTTCAA<br>GTGGCAAAATTTTTCTTATATACCAACTTCGAACCACTTTATATGACATACCAACT<br>CCTAGTTGGGCGAAAATTCCTCCGTCGAAGATATAATACTTGGATTGGTCAAATG<br>AATTGTGAAAGGATACACGTGATGTGGTCTGGAATTAATTTGTTTGAATGATCAGT<br>TGGGTTCGGGGCGACAACTGTGAACTGGAACCACCCTAAGTAAATTTTCTTTCTGT<br>CCTACAAATTTGAGGTTCTCCTTGATCACCTTAGTCCATCTTAGGTTTGCCCGTTAG<br>TAAGATCTGCATTTAGCAGTTTGTCCTGGTATTTGATATCACTAGTATCTTTGTTTG<br>ATTCCCTAGCATCTCTGAAACCATCGGACAAGTAGGTGGTTTAGGACAAATTTGG<br>TTCATTGCGGCATTTTTTGTTTGTATCGCCGTATCATCTGGAAGAAGCAGACAGTTT<br>TGCAAAGTGGCATCAAGCTCAAGAAAGCAACGGCTAGAAGAAGTTCTACATCTG<br>ATGCTTTCCTTTTGTTTCTTTGTGTGCTTTTTGGACTTTGTTCTTTTTTCCTGTAGGAT<br>CCAAAAACAGAAAACAACCCCTACCTCGGTTTCATCTACACCTCATTCCAAGAGA<br>GGGCAACGTTCATCTCCCACGGAAACACAGCCAGACTCGCCAAGGACCATGGGG<br>ACATGAAGCTGGCGCAGATCTGCGGGATCATCGCAGCAGACGAGAAACGGCACG<br>AAACCGCATACACCAAGATCGTCGAGAAGCTCTTCGAGATCGACCCTGACGGTA<br>CAGTGCTGGCACTGGCGGACATGATGAGGAAGAAGATATCGATGCCCGCCCACT<br>TGATGTACGATGGAGAAGACGACAACCTCTTCGACAATTACTCGTCAGTCGCTCA<br>ACGCATCGGGGTGTATACTGCCAAGGATTATGCCGATATCCTGGAGTTCCTGGTG<br>GGGAGGTGGAAAGTGGATGCTTTTACGGGGCTTTCCGGGGAAGGGAACAAAGCT

	CAGGATTTTGTCTGCGGGCTTCCTGCGAGGATTCGAAAGTTGGAGGAGAGGGCTG<br>CGGGGAGGGCAAAGCAAACGTCGAAATCTGTCCCGTTCAGCTGGATCTTCAGCA<br>GAGAATTGGTACTCTAA
SAD2-2	ATGGCTCTCAAGCTCAACCCAGTCACCACCTTCCCTTCGACCCGCTCCCTCAACA<br>ACTTCTCCTCCAGATCTCCTCGCACCTTTCTCATGGCTGCTTCCACTTTCAATTCCA<br>CTTCCACCAAGTAAGTTCCCGTCACCATCTCCTCTTCCTCGGAATCTCCGCCGTTTC<br>ATTTAAGCGATTGATCGTAGAAAATCTGTCGGTTGCTTAGCGTTCATTCAAATCTG<br>CGCGGTTCGTTTCTTTTTCTTTCTTCAGACTGCATCATCTGCATTATGTTATTGTTCG<br>TTTCCGATTTGACTAACCTACATAATCAATTCCTTTGTGTTTCACGAGTCTGGATTT<br>TGCGCTGTAATTGATTGTCAGCGTTTGCACAGGTTTCCATTTCTCCACCTCCGTCCA<br>TCAAATGCATGTTATTACCTACCAATTTCAGCGTCTTTCTCTGGAAATTTCTGTCTC<br>TGTATCTACTATCCTATTAGCTTGTTTGAGAGAGGTTCAATATTGGTTTGCATGAAC<br>CAAGTGGCTTACAATCCTTCAACGTTCTAAATGTTGGTCGCAGTAACAATAGGTTC<br>TCAAAAGAGGTTTTTCTATGTTGTTTGGCAAAATCTTGTTTCTGTGAATCATGTTAA<br>GGTCCTGGGAAGAATGATTAATGAGCTATGACATGATTAAGGCGTAGTAGTTATT<br>GAACTGCTGATAATTCAATATAGGGGTAACTTTGTTGGTTGTTTGGTGACAGGGAG<br>GCTGAGAAGCTAAAGAAGTCACATGGACCACCAAAAGAGGTGCATATGCAAGTG<br>ACCCATTCCATGCCCCCACAGAAGCTGGAGATCTTTAAGTCCCTTGAAGGTTGGG<br>CAGAGGACGTTCTGTTGCCGCACCTGAAGCCGGTTGAGAAATGCTGGCAGCCACA<br>AGATTTCCTGCCCGAACCCGAGTCGGATGGGTTCGAGGAGCAAGTGAAGGAGCT<br>CAGGGCAAGGGCTAAAGAACTCCCCGATGACTATTTTGTTGTGCTGGTTGGGGAT<br>ATGATCACCGAAGAAGCTCTACCGACTTACCAGACAATGCTCAACACCCTTGACG<br>GGGTGAGGGACGAGACTGGAGCCAGCCTTACGCCGTGGGCAATCTGGACAAGGG<br>CGTGGACCGCTGAAGAGAATAGGCACGGTGACCTTCTCAACAAGTATCTTTACCT<br>CTCTGGAAGGGTGGACATGAGGCAAATTGAAAAGACCATTCAGTATCTCATCGGC<br>TCTGGAATGGTATGTACTCACATCCTATCTGCTCCTTTATCCTTTTCCATTAATCTTT<br>GATTGAACAAAATTCAATAAACTGGTAGCTGAAACTTTAGATGATTTGTTATAAC<br>TGCCTAGCTTCTATGAGAAAACCACTGAAGTCAAATAGGTTTGACAATGGGTTTA<br>AATGGAAAAAGTTTCATATACCATCTTCCATCTATTTTATATGACATACCAACTTC<br>TACTTTGGAGAAAATTCGCCGTGGATAATCATATTATTGAAGATATAGTACTTAGT<br>AGATTGGTTAGATGAACTGTTAAACAATACATGTGATGTCGTGTGCAATTAATTTG<br>TGTAAATGATTAGCTGGGTTCGGGACGACAAATGTGAACTGGAACCCTAGTAAAC<br>TATGAATTGAGGTTGTCCTTCATCACTTTATTCTGTCCTGGGTTTGTTTGCCTGTTTG<br>CAAGATCTGCATGTAGCAGTTTGTCCTGGTATTTGCTACCAGTGGTATCTTTGTTTG<br>ATTCCCTAGCATCTCTGAAAACATCGGACCAAGTATCTGGTTAGGACAAATTTGG<br>TTCATTGCGGCATTTTTTGTTTGTATCGCTGTATCGTCTGGAAGAAGCAGACAGTTT<br>TGCAAAGTGGCATCAAGCTCAAGAAAGCAACGGCTAGAAGAAGTTCTACATCTG<br>ATGCGTTCCTTTTGTTTCTTTGTGTGCTTTTTGGACTTTGTTCTTTTTGCCTGTAGGAT<br>CCAAAAACAGAAAACAACCCCTACCTCGGTTTCATCTACACCTCATTCCAAGAGA<br>GGGCAACGTTCATCTCCCACGGAAATACGGCCAGACTCGCCAAGGACCACGGGG<br>ACATGAAGCTGGCGCAGATCTGCGGGATCATCGCAGCAGACGAGAAGCGGCACG<br>AAACAGCATACACCAAGATCGTCGAGAAGCTCTTCGAGATCGACCCTGACGGTA<br>CAGTGTTGGCTCTGGCGGACATGATGAGGAAGAAGATATCGATGCCCGCCCACTT<br>GATGTACGATGGAGAAGACGACAACCTCTTCGACAATTACTCGTCGGTCGCTCAA<br>CGCATCGGGGTGTATACTGCCAAGGATTATGCTGATATCCTGGAGTTCCTGGTGG<br>GGAGGTGGAAAGTGGATGCTTTTACGGGACTTTCCGGGGAAGGGAACAAAGCTC<br>AGGAGTTTGTCTGTGGGCTTCCAGCGAGGATTCGAAAATTGGAGGAGAGGGCTGC<br>GGGGAGGGCAAAGCAAACGTCGAAATCTGTCCCATTCAGCTGGATCTTCAGCAG<br>AGAATTGGTACTCTAA
SAD3-1	ATGCAAGCCCCCCACCACGTCAGCGCCCACCGTCCAGCATGGATGCCACGTGGTG<br>GCCGAACCACCTCCACCACCATCATGCACCTCTGCCCTCCACGCGCCGCCATCAA<br>AACTGCTCCGCCGCCTCCCCCTCCGCTCACCCTGAAACACCAACACAAAACCCAC<br>ACAATGCCGGCGGAGAAAATCGAGCTTTTCAAGTCCCTCGAGGATTGGGCCTCCG

	AGAATGTCCTCCCACTTCTCAAGCCCGTCAATAAATGCTGGCAGCCTCAGGACTT<br>CCTCCCCGACCCGTCCCTCTCCGTCGCTGATTTCTCCGACGAGGTAATGAACAATT<br>GCGTCTCAATACCTTATATAAGCTACACTTTTACAGGGATTTTGAGCTAATATTTG<br>ACAAATTCATCAGTTACGTTTTTTGTCATTACGGATTAGTCATTGTAGTTTTGTCAT<br>TTGTTCATATAGCTACTCATTTTTCAGTTCATCAAATTCTCATTTTCTATAGTAAGA<br>TCATTTGGAAGGGTTTTGGAGAGTAATTTCTTGGTCCCACATGATTTGATCGAAAT<br>ATCATTTTAAAATACAAAAATAAATTGGGATTTGAAATCACTTCGATAGGCTAGG<br>TTCCCCAAAAGGTGATTTCAGGCTCCCAAAAGGTGATTTCAAATTCATTTTATGGA<br>CTTGAGATTTGCAAAATCTCAAGTAATTTGAATAGTAAATGATCAAATTATCTTAT<br>TGAGTTTAAGTGAACGAGATAAATTAAGAGGTGCATTAGTCATTTAAAAAATCAA<br>TTTTTTTTTTTAATTTTCAAAACTATTTTGTGATTGACCGTTAAACCAGTTCGGTTTG<br>ATACTGATTTTGAGTTTTGACTGACAGGTGAGGTTGTTGAGGGAAAGGACGGCGG<br>AGCTACCGGACGAGTACTTCGTCGTCTTGGTGGGTGACATGATAACGGAGGATGC<br>GTTACCGACGTACGAGACCATGATCAACACACTTGACGGCGTTAGGGACGAGAC<br>GGGCGCCAGTCAAAGCCCATGGGCCTTGTGGACCCGGGCCTGGACCGCCGAGGA<br>GAATCGCCACGGCGATCTGCTTCGGACCTTTCTGTACCTATCGGGTCGGGTCGACA<br>TGAGGATGGTAGAAAGGACTGTTCAGTACCTCATTGGATCTGGCATGGTAAGTGT<br>AGAATAGCCCTTTCGGTATTCCGAACCGGTTTGATCCGTCAGTGGGATCTTTCGTT<br>CGATTAATCGGGTGATGAGTATATGCCAAACTGGCACTAATTTGTCACTTGATTTG<br>ATAGTACATTTTATAAGTTAGTGTTAGCTAGTTACATTGTTTGAGAATGTCACGAA<br>TTCGAAGGAGAAAAAAACCAAAATGCCACTTCGATAATTTATTTTACATGATATA<br>GTCATTGAACCTTCAATCTTTTTACAAATCGGATCCAAATTTGTGTACGCAGTGGT<br>CACTCAACTGTCATTTTAGAATAGGAATAGCATTTTAACAAGTGATTTACTCGGGT<br>CGGGTAACATTACAGGACCCGGGCACAGAGAACAACCCGTACTTGGGGTTCGTG<br>TACACGTCATTCCAGGAGCGAGCCACATTCATATCGCACGGCAACACGGCTCGGC<br>TAGCCAAGGAGGGAGGGGATCCGGTACTCGCACGGATATGTGGCACCATAGCAT<br>CGGACGAAAAGCGACACGAGAACGCCTACACGCGCATCGTGGAGAAGCTCCTAG<br>AGGTGGACCCGAACGGTGCGGTTTGTGCGGTGGCCGACATGATGCATAAGAAGA<br>TCACAATGCCGGCGCACCTGATGTACGACGGGGAGGACCCGAAGCTGTTCGAGC<br>ACTTCTCATCGGTGGCTCAGCGGCTGGGGGTGTACACCGCGCAGGACTACGCGGA<br>CATACTCGAGTTCTTGATCGGACGGTGGGGGCTGGACAAGGTGGAGCAAGGGCT<br>GGACGGTGAAGGGAGGCGGGCGCAGGATTTCGTGTGTGGGCTGCCACGGAGGAT<br>CATGCGGCTCCAGGAGCGAGCCGATGAGAAGGCTAAGAAGATGAAGAAGCCGG<br>AAGCAGTCAAGTTTAGCTGGATTTTCAAACGGCAAGTCGTTTTGTGA
SAD3-2	ATGCACCTCTGCCCTCCACGCGCCGCCATCAAAACTGCTCCGCCGCCGCCTCCTC<br>CGCTCGCCCTGAAACACCAACACAAAACCCACACAATGCCGGCGGAGAAAATCG<br>AGCTTTTCAAATCCCTCGAGGATTGGGCCTCCGTCAATGTCCTCCCACTTCTCAAG<br>CCCGTCGACAAATGCTGGCAGCCTCAGGACTTCCTCCCCGACCCGTCCCTCTCCGT<br>CGCTGATTTCTCCGACCAGGTAATGTACATTGTGTTCCAATAACTCGTATTGTAAT<br>GCTACTCGTATTTTGGGCTAATATTTGATAAATTCGTCACTTAACTTTTTTGTCTTG<br>ACGGATTAGTCATTGTAGTTTTATTATTTGTTCGATTTTCAGTTAAATAATTCTCATT<br>TTTTATAGTAAGATCATTTGGAAGGAATTTGGAGAGTAATTTCATGGTCCTGGGTT<br>GATTTGATCGAAATATCGCGTTGGAACACGAAAATAAAGATGGATTTGAGTAGA<br>AAATAACCAAATTGACTTGTTGAAATTGTATTGTTGAGTGAACAGGGGGTTTATTA<br>GTCATTTACAAAATTAAAATGTTTCTTTTAATGTCCAAAACTCAACAAAACACTGA<br>TTTTGAGATTGGCCGTTAAACCAGTTCGGTTTGATAAATACTGATTTTGAGTTTTGA<br>CTGACAGGTGAGGTTGTTGAGGGAAAGGACGGCGGAGCTACCGGACGAGTACTT<br>CGTCGTCTTGGTGGGTGACATGATAACGGAGGATGCGTTACCGACGTACGAGACC<br>ATGATCAACACCCTTGACGGCGTTAGGGACGAGACGGGCGCCAGTCAAAGCCCA<br>TGGGCCCTGTGGACCCGAGCCTGGACCGCCGAGGAGAATCGCCACGGGGATTTG<br>CTCCGGACCTTTCTGTACCTCTCGGGTCGGGTCGACATGAGGATGGTTGAAAAGA<br>CTGTTCAGTACCTCATTGGATCTGGCATGGTAAAGTGCAGAACAGTCCTTTTTGTA<br>TTCCGAACTGGTTTGATCGGTTGGTCGGTTCGTTCGTTTGTTAATTGGGTGATGAGT

	ATGCGAAATTGTCACTAAATTATCATTTCATAGTACATTTATAAGCTAGTGTTTAGTAACAATTGTTTAAGAATGTTACAAATTTGAACCGGGAAAAAACCAAAATGCCACTTGGATAATTTATTTGCATGATATAGTCATTGAACCTTTAATCTTCTTACAAATCGGATCCTAATTTGTGTACACATTGGTCACTCAACTGTCATTTTAGAATAGGAATAGCATTTTAACAAGTGATTTACTCGGGTCGGGTAACAATTGCAGGACCCTGGCACGGAGAACAACCCGTACTTGGGGTTCGTATACACGTCATTCCAGGAGCGAGCCACATTCATATCGCACGGCAACACGGCTCGGCTAGCCAAGGAGGGAGGGGATCCGGTACTTGCACGAATATGCGGCACCATAGCATCGGATGAGAAGCGACACGAGAACGCCTACACGCGCATCGTGGAGAAGCTCCTCGAGGTGGACCCGAACGGTGCGGTTTGTGCCGTGGCCGACATGATGCGTAAAAAGATCACAATGCCGGCGCACCTGATGTACGACGGGGAGGACCCAAAGCTGTTCGAGCACTTCTCGGCGGTGGCGCAGCGGCTCGGGGTGTACACGGCGCAGGACTACGCGGACATACTGGAGTTCTTGATCGGACGCTGGGGGCTGGACAAGGTGGAACAAGGGCTGGACGGTGAAGGGAGGCGGGCGCAGGATTTCGTGTGTGGGCTGCCGCAGAGGATCATCCGACTCCAGGAGCGAGCCGATGAGAAGGCTAAGAAGATGAAGAAGCCGGAAGCCGTCAAATTTAGCTGGATCTTCAAACGGCAAGTCGTCTTGTGA
**Atlant**	
FAD2a-1	ATGGGTGCCGGTGGCAGAATGTCAGTGCCTCCATCATCCAAACCTATGAAGAGGTCTCCTTACTCAAAGCCACCATTCACGCTCGGTGAGCTCAAGAAGGCCATCCCTCCACACTGTTTCAAACGCTCAATCCCCCGATCGTTCGCCTACGTGGCGTACGACCTCACCATTGCAGCAATCTTCTACTACATCGCCACCACTTACTTCCACCTCCTCCCTAGCCCTCTCAACTACCTCGCCTGGCCGGTCTACTGGGCCTGCCAGGGCTGCATCCTCACTGGAGTATGGGTGTTGGCTCACGAATGCGGTCACCATGCCTTCAGCGACTACCAGTGGCTCGACGACATGGTTGGCTTCGTCCTCCATTCGTCCCTCCTTGTTCCTTACTTCTCCTGGAAGCACAGCCACCGCCGCCACCATTCCAACACGGGATCGCTTGATCGTGATGAGGTGTTTGTCCCCAAGCAGAAGGCCGAAATCGGGTGGTACTCCAAGTACCTTAACAACCCACCTGGCCGTGTGATCACATTGGCCGTCACATTAACGCTCGGTTGGCCTCTGTACTTGGCATTCAACGTCTCCGGGAGACCATATGACCGGTTCGCATGCCATTTTGACCCTCACGGTCCGATTTACAATGATCGCGAGCGTATGGAGATATACCTATCCGACGCAGGGATATTCACCGTGTGCTACATCCTATACAGACTCGTCCTCACGAAAGGACTCGTTTGGGTCGTGTCCATATACGGAGTCCCACTATTGATAGTGAATGGATTCCTAGTCCTCATCACTTTCTTGCAGCACACGCATCCTTCTCTTCCGCACTACAAGTCCTCCGAATGGGACTGGATGCGAGGCGCCCTCTCGACCGTGGATCGAGACTACGGGTTACTCAACACCGTGTTCCACAACATCACCGATACACATGTCGCGCACCATCTCTTCTCCACGATGCCTCATTACCACGCGATGGAGGCTACCAAGGCGATCAAGCCGGTTCTCGGGGAGTATTACCAGTTCGATGGGACTCCCTTTGTGAAGGCCATGTGGAGGGAGGCAAAGGAGTGCATCTATGTCGAGCCGGATGAAGGCGACCCCAGCCAAGGCGTGTTCTGGTACAACAACAAGCTGTGA
FAD2a-2	ATGGGTGCCGGTGGCAGAATGTCAGTGCCTCCATCATCCAAACCTATGAAGAGGTCTCCTTACTCAAAGCCACCATTCACGCTCGGTGAGCTCAAGAAGGCCATTCCTCCACACTGTTTCAAACGTTCAATCCCCCGATCGTTCGCCTACGTGGCGTACGACCTCACCATTGCAGCAATCTTCTACTACATCGCCACCACTTACTTCCACCTCCTCCCTAGCCCTCTCAACTACCTCGCCTGGCCGGTCTACTGGGCCTGCCAGGGCTGCATCCTCACTGGAGTATGGGTGTTGGCTCACGAATGCGGTCACCATGCCTTCAGCGACTACCAGTGGCTCGACGACATGGTTGGCTTCGTCCTCCATTCGTCCCTCCTTGTTCCTTACTTCTCCTGGAAGCACAGCCACCGCCGCCACCATTCCAACACGGGGTCGCTTGATCGTGATGAGGTGTTTGTCCCCAAGCAGAAGGCCGAAATCGGGTGGTACTCCAAGTACCTTAACAACCCACCTGGCCGTGTGATCACATTGGCCGTCACATTAACGCTCGGTTGGCCTCTGTACTTGGCATTCAACGTCTCCGGGAGACCATATGACCGGTTCGCATGCCATTTCGACCCTCACGGTCCGATTTACAATGATCGCGAGCGTATGGAGATATACCTATCCGACGCAGGGATATTCACCGTGTGCTACATCCTATACAGACTCGTCCTCACGAAAGGACTCGTTTGGGTCGTGTCCATTTACGGAGTCCCACTATTGATAGTGAATGGATTCCTAGTCCTCATCACTTTCTTGCAGCACACGCATCCTTCTCTTCCGCACTACAAGT

	CCTCCGAATGGGACTGGCTGCGAGGCGCCCTCTCGACCGTGGATCGAGACTACGG<br>GTTACTCAACACCGTGTTCCACAACATCACCGACACACATGTCGCGCACCATCTC<br>TTCTCCACGATGCCTCATTACCACGCGATGGAGGCTACCAAGGCGATCAAGCCGG<br>TTCTCGGGGAGTATTACCAGTTCGATGGGACTCCCTTTGTGAAGGCCATGTGGAG<br>GGAGGCAAAGGAGTGCATCTATGTCGAGCCGGATGAAGGCGACCCCAGCCAAGG<br>CGTGTTCTGGTACAACAACAAGCTGTGA
FAD2b-1	ATGGGTGCTGGTGGAAGAATGGCCGTGCCTCCATCGAACAAGGCGGACTCCGAA<br>ACCTTTAAGCGGTCTCCTTACTCAAAACCTCCCTTCACTCTTGGTGAGATCAAGAA<br>AGCCGTCCCTCCACACTGCTTCAAAAGGTCCATCCCCCGCTCGTTCTCCTACGTGG<br>CTTATGACCTCACCATAGCCGCCATCTTCTACTACATCGCCACCACTTACATCCAC<br>CTCCTCCCCAATCCTCTCTCCTACGTGGCGTGGCCGATCTACTGGGCCTGCCAAGG<br>CTGCGTCCTCACTGGTGTCTGGGTCCTAGCCCACGAATGCGGTCACCACGCCTTCA<br>GCGACTACCAATGGCTCGATGACTTGGTCGGCTTTGTCCTCCACTCATGCCTCATG<br>GTACCCTACTTCTCGTGGAAGCACAGCCACCGTCGCCACCACTCCAATACTGGGT<br>CCCTCGAACGAGACGAGGTTTTTGTCCCCAAGCAGAAATCAGCCATTGGCTGGCA<br>CTCAAAGTACCTCAACAACCCACCTGGCCGTGTGCTCACACTTGCAGTCACTCTC<br>ACTCTCGGCTGGCCTTTGTACTTGGCATTCAACGTCTCTGGAAGGCCGTACGACCG<br>GTTCGCCTGCCATTACGATCCTAAATCCCCCATCTACAACGACCGCGAGCGAACG<br>GAGATATTCTTCTCCGATGCTGGCATCCTTGCTGTGAGCTTTGCGCTCTACAAGCTT<br>GCTGTCGCCAAGGGACTGGCTTGGGTGGTTTGTGTCTACGGAGTTCCACTCCTTGT<br>AGTGAATGGATTCCTTGTCTTGATCACTTTCTTGCAGCACACCCACCCATCATTGC<br>CGCACCACAAATCCTCCGAATGGGACTGGCTGAGAGGTGCTCTGGCGACCATGG<br>ACAGAGACTACGGGTTTCTGAACACGGTGTTCCATAACATCACGGATACCCACGT<br>GGCGCATCACCTGTTCTCGACGATGCCTCATTACCATGCAATGGAAGCTACAAAG<br>GCGATCAAGCCGGTACTGGGAGAGTACTACCACTTCGACGGGACTCCATTCATCA<br>AGGCGATGTGGAGGGAGGCTAAGGAGTGTGTCTATGTCGAGCCTGACGAAGGTG<br>ACCAGAACAAAGGCGTGTTCTGGTACAACAACAAGCTGTGA
FAD2b-2	ATGGGTGCTGGTGGAAGAATGGCTGTGCCTCCATCGAACAAGGCGGACTCGGAA<br>ACCTTTAAGCGGTCTCCATACTCAAAACCTCCATTCACACTTGGTGAGATCAAGA<br>AAGCCATCCCTCCACATTGCTTCAAAAGGTCCATCCCCCGCTCATTCTCCTATGTG<br>GCTTATGACCTCACCATTGCCGCCATCTTCTACTACATCGCCACCACTTACATCCA<br>CCTCCTCCCCAATCCTCTCTCCTACGTTGCGTGGCCTATCTACTGGGCCTGCCAAG<br>GCTGCGTCCTCACCGGTGTCTGGGTCCTAGCCCACGAATGCGGTCACCACGCCTTC<br>AGCGACTACCAATGGCTTGATGACTTGGTCGGCTTTGTCCTTCACTCATGCCTCAT<br>GGTCCCCTACTTCTCGTGGAAGCACAGCCACCGTCGCCACCACTCCAACACCGGG<br>TCCCTTGAACGAGATGAGGTTTTCGTCCCCAAGCAGAAATCGGCCATCGGCTGGC<br>ACTCAAAGTACCTCAACAATCCACCTGGCCGTGTGCTCACACTTGCAGTCACTCT<br>CACTCTCGGCTGGCCTTTATACTTGGCATTCAACGTTTCCGGAAGGCCGTACGACC<br>GGTTCGCCTGCCATTACGATCCTAAATCCCCTATCTACAACGACCGCGAGCGAAC<br>TGAGATCTTCCTCTCCGATGCCGGCATCCTTGCTGTGAGCTTTGCAATCTACAAGC<br>TTGCTGTCGCCAAGGGACTGGCTTGGGTGGTTTGTGTCTATGGAGTTCCACTCCTT<br>GTAGTGAATGGATTTCTTGTCTTAATCACTTTCTTGCAGCACACCCACCCATCATT<br>GCCGCACTACAAATCCTCCGAATGGGACTGGCTGAGAGGTGCTCTGGCGACCATG<br>GACAGAGACTACGGGTTTCTGAACACGGTGTTCCATAACATCACGGATACCCACG<br>TGGCACATCACCTGTTCTCGACGATGCCTCATTACCATGCGATGGAAGCTACCAA<br>GGCGATCAAGCCGGTACTGGGAGAGTACTACCAGTTCGATGGGACTCCATTCATC<br>AAGGCGATGTGGAGGGAGGCTAAGGAGTGTGTCTATGTTGAGCCTGACGAAGGT<br>GACCAGAACAAAGGCGTGTTCTGGTACAACAACAAGCTGTGA
FAD2c-1	ATGTTCTCATCCTCTGCAACTAAAATGCCGGCGCCGCCTTCATCATCCAACACGA<br>CCATGAAGCGATCGCCTCACTCAAAGCCGCCGTTCACCGTCTCCGACGTCAAGAA<br>AGCCATTCCACCACACTGCTTCCAAAGGTCCCTCCTCCGATCATTCTCCTACCTAA<br>CCTACGACCTCACCATCATCACAATCCTCTACCAAGTCGCCACCACTTACTTCCAC<br>CTCCTCCCGACACCTCTCTCATCCTACGTGGCATGGCCGGCCTACTGGGCCGGCCA

	AGGCTGCTTCTTCGTCGCTGTATGGATGGTGGCCCACGAGTGTGGCCATCACGCCT<br>TCAGCGACCAGCACTGGCTGGAAGACTCGGTCGGATTCATCCTCCACTCAGCCCT<br>CCTTTCACCTTACTTCTCATGGAAGCACAGCCACCGTCGCCACCATGCCAACACG<br>AGCTCCCTCGAACGTGACGAGGTGTTCGTCCCTAAACCCAAGTCCAAGCTGAGCT<br>GGCACTTCAAGTTCTTCAACAACCCGCCTGGCCGCGTCCTGCAGTTGGCATTTGCT<br>CTGCTTCTTGGATGGCCTCTCTATTTGGCGATCAACATCGCCGGAAGGCCGTACGA<br>GAAATTCGCTAGCCACTTCGATCCGAGAAGCCCTATCTACAACGATCGAGAGCGG<br>ATCGAGATATTCGCATCCGATGTTGGCGTGCTCTGCATGTGGTTCGCGCTGTATAA<br>ACTTGCTCTAGTGAACGGAGTTGGTTGGGTTGTTTGTGTGTACGGGATCCCGCTGT<br>TGGTGATGAATGGATGGGTCGTTACGATCACTTACTTGCATCACACGCATATAGC<br>GTTGCCGCGGTATGATTCGTCCGAATGGGATTGGTTGAGGGGTGCATTGGCGACT<br>GTGGATAGGGACTATGGTGTTTTGAACAAGGTGTTTCATAACATCACGGATACTC<br>ATGTGGCGCACCATCTGTTTTCGGCGATGCCTCATTACCATGCGGCGGAAGCGAC<br>CGAGGCGATCAAGCCCGTTTTGGGCGAGTATTATCGGTGTGACCGGACTCCTATT<br>ATCAAGGCGTTATGGAGGGAGTTTAAGCACTGTATTTATGTCGAGTCAGATGAGG<br>ATAAAGGCGTGTTTTGGTTCAATGACAAGTTGTAA
FAD2c-2	ATGCCGCCGCCGCCTTCATCATCCAACACAACCATGAAGCGATCGCCACACTCAA<br>AGCCGCCGTTCACCCTCTCTGACATCAAGAAAGCCATACCACCGCACTGCTTCCA<br>AAGGTCCCTCCTCCGATCATTTGCCTACTTAGCCTACGACCTCACCGCCGTCACAA<br>TCCTCTACCACATCGCCACCACGTACTTCCACCTCCTCCCGACACCTCTCTCATCC<br>TACGTGGCTTGGCCGGCCTACTGGGCCAGCCAAGGCTGCTTCTTCGTCGCTGTATG<br>GATGGTGGCCCACGAGTGCGGTCACCACGCCTTTAGCGACCAACACTGGCTGGA<br>AGACTCGGTAGGGTTCATCCTCCACTCGGCCCTCCTTTCGCCTTACTTCTCGTGGA<br>AGCACAGCCACCGCCGCCACCACGCCAACACTAGCTCCCTCGAGCGTGACGAGG<br>TGTTCGTCCCTAAGCTGAAATCCAAGCTGAGCTGGCACTTCAAGTTCTTCAATAAC<br>CCGCCTGGCCGCGTCCTGCAGCTGGCCTTTGCTCTGCTTCTAGGATGGCCTCTCTA<br>CCTTGCGGTCAACATCGCAGGAAGACCGTACGAGAAATTCGCTAGCCACTTCGAT<br>CCGCGAAGCCCTATCTATAACGATCGGGAGCGAAATGAGATATTTGTATCTGATG<br>TTGGCGTGCTTGCTATGTGGTTCGCTCTGTATAAACTTGCCCTGGCGAACGGAGTT<br>GGTTGGGTTGTTTGTGTGTACGGGGTCCCTCTGTTGGTGATGAATGGATGGATCGT<br>TACGATCACTTACTTGCATCACACGCATGTCGCATTGCCGCGGTATGATTCCTCGG<br>AGTGGGATTGGTTGAGGGGTGCGCTCGCGACTGTAGATAGGGATTATGGGGTTTT<br>GAACAAGGTGTTTCACAACATTACAGATACTCATGTGGCGCACCATCTGTTTTCG<br>ACGATGCCTCATTACCATGCGGCGGAAGCAACCGAAGCGATCAAGCCCGTTTTGG<br>GTGAGTATTATCGGTCTGACCGGACTCCTATCGTCAAGGCATTATGGAGGGAGAC<br>TAAGCACTGTGTTTATGTCGAGGTTGATGAAAAAAAAGGCGTGTTGTGGTTCAAT<br>GATAAGATGTGA
FAD2d-1	ATGGTATCCAATACCACCATAAAGCGAACACCGACCTCAAAACCGCCGTTCACC<br>CTCTCCGACGTCAAGAAAGCAATACCACCGCACTGCTTCCAAAGATCCCTCCTCA<br>AATCCTTCACCTACCTAGCCTACGATCTCACCGTCATCACAATCCTCTACCACATC<br>GCCACGTCATACTTCCACCTCCTCCCCAACCCTCTCTCCTACGTGGCGTGGCCGCT<br>CTACTGGGCCGCCCAGGGGTCACACTTCATCGCCGTCTGGGTCATCGCCCACGAG<br>TGCGGCCACCACGCCTTCAGCGACTACCAGTGGCTAGACGACGCCGTTGGGTTCG<br>TCCTCCACTCCCTCCTCTTAGCTCCTTACTTCTCCTGGAAGCACAGCCACCGCCGT<br>CACCACGCCAACGCCGCCTCCATCGAGCGCGACGAGAATTACATCCCGAAAAAG<br>AAGGACGAAGTCAACTGGCATTTTAAATACCTCGACAACCCTCCCGGACACGTGT<br>TCTATATATTCTTCACTCTCACCCTCGGATGGCCGCTCTACTTGCTGGTCAACATCT<br>CCGGCAGGAAATACGACGACGGATTCGCCAGCCATTTGTATCCTTTCTCCCCTATC<br>TACAACGACCGCGAGAGATTCGGGATAGTCCTCTCCGTCGCCGGGATGCTCGCCA<br>CGTGGTTCGGTCTTTATAAACTCGCGATGGTCAACGGGTTCGGTTGGGTTGTTTGC<br>GTGTACGGGGTGCCTCTCATTCTCCAGAATGCGATGTTGATTACCATCACTTACTT<br>GCACCACACGCATCTCAACTTGCCCCACTACGACTCGTCGGAGTGGGACTGGATG<br>AGGGGTGCGCTCGCGACTGTGGATAGGGATTACGGAATTTTGAATAAGGTGATGC

	ATAATATTACGGATACCCACGTGGCGCACCATCTGTTCTCGATGATTCCTCATTAT<br>CATGCGATGGAGGCTACAAATGCGATAAAGCCGGTTCTTGGGGAGTACTACCAA<br>GTTGATACTACGCCGTTTTGAAGGCGTTGTGGAGAGAGACTAAGGATTGTGTTTA<br>TGTTGAGGCGGATGATGAGGGATCTGATCGGGAGAAGAAAGGAGGGGTGTTTTG<br>GTTCAAAACCAAGCTGTAA
FAD2d-2	ATGGTATCCAATACCACCCTAAAGCGAACACCGACCACAAAACCGCCGTTCACC<br>CTCTCCGACCTCAAGAAAGCAATACCACCACACTGCTTCCAAAGATCCCTCCTCA<br>AATCCTTCTCCTACCTAGCCTACGACCTCACCGTCATCACAATCCTCTACCACATC<br>GCCACGTCATACTTCCACCTCCTTCCCAAACCTCTCTCCTACGTGGCCTGGGTGGC<br>CTATTGGGCCGCTCAGGGGTCCCACTTCATCGCCGTGTGGGTCATCGCCCACGAG<br>TGCGGCCACCACGCCTTCAGCGACTACCAGTGGCTAGACGACGCCGTCGGCTTCC<br>TTCTCCACTCCCTCCTCTTAGCTCCTTACTTCTCCTGGAAGCACAGCCACCGCCGC<br>CACCACGCCAACGCCGCCTCCATCGAACGTGACGAGAATTACATCCCGAGAAAG<br>AAGGACGAAGTCAACTGGCATTTTAAGTACCTTGATAACCCTCCCGGACACGTGT<br>TCTATATATTCTTCACTCTCACCCTCGGATGGCCGCTCTACTTGCTGGTCAACATCT<br>CCGGGAGGAAATACGACGACGGATTCGCCAGCCATTTGTATCCTTTCTCCCCGAT<br>CTACAACGATCGGGAGAGGTTCGGGGTGTTCCTCTCCGTCGCGGGGATGCTCGCC<br>ACGTGGTTCGGACTTTATAAGCTCGCAATGGTCAACGGTCTCGGCTGGGTTGTTTG<br>CGTGTACGGGGTCCCTCTGATTCTCCAGAATGCGATGTTGATTACCATCACTTACT<br>TGCACCACACGCATCTCAACCTGCCCCACTACGACTCGTCGGAGTGGGACTGGAT<br>GCGGGGTGCGCTCGCGACTGTGGATAGGGACTACGGGATTTTGAACAAGGTAAT<br>GCATAATATTACGGATACTCACGTGGCACACCATCTGTTTTCGATGATTCCTCATT<br>ACCATGCGATGGAGGCTACGAATACGATCAGGCCGGTTCTGGGGGAGTACTACC<br>AGGCCGATACGACGCCGTTTATTAAGGCGTTGTGGAGGGAGACTAAGGACTGTGT<br>TTATGTTGAGGCGGATGATGAGGGCGGTGATCAGGAGAAGAAAGGAGGGGTGTT<br>CTGGTTCAAAACCAAACTGTGA
FAD2e-1	ATGTCCCCTATTTACAGCGACCGCGAGCGAGCCGAGGTATTCGCCTCAGATGTTG<br>GCTTGCTCGCTGTCTGCTTCGCGTTGTACAAACTTATTATGGTCAAGGGAATGGCG<br>TGGGTTTTTTGCGTCTATGGGGCTCCGGTCATGGTGGTGAATGGATTCTTCATTACC<br>ATCACTTACTTGCATCACACTCATCTCGCGGTCCCGCGATACGATTCGTCTGAATG<br>GGATTGGTTGAGGGGAGCCCTGGCAACCATGGACAGGGACTTTGGCCTTTTGAAC<br>AAGGTGTTCCATAATGTTACAGATACTCACGTGACGCACCATCTGATTTCGACGA<br>TCCCTCATTATCATGCCATGGAAGCCAACAACGCAATTAGGCCCGTGTTGGGGGA<br>CTACTACCATATCGACAGGACGCCGGTGGTTAAGGCGTTGTGGAGGGAGGCTAA<br>GGAGTGTGTTTACATCGAGGCCGATGATGGTGAAAAGAACAAAGGCGTGTTCTG<br>GTTCAATACCAAGCTCTAA
FAD2e-2 (exon-intron junctions are not given)	ATGCACTTTCCGATAGCTAGCCTTTGTCTCATGCTAAAAACAGGCCATACATGGC<br>GCCACAAAGCACTGCTCTAGTAAGTACACATGATTGGGATTACACTACACTTCAG<br>TAATTAAATCAGTCCATATTTTACCTGGATCCAAAGTTGAAAACTAACTCAAAAA<br>CAAATTTGTGCAAAATTGAGCATCAAATGAGACAATATTAAACTATTTCGAGTTTT<br>GTTGGGCTGTTCTCGAGTTGTACTAGCCTCTCCGAAAACATGTATAAAAAGCACTC<br>GACCTATTCATATTAGACAACATCAAAATCCCACCAATCAATCAAAAAACAGAG<br>AGACACATCTAAAAGCGCCCCCCCCCCCCCCCCCTTTACAAAAATGGTGGAGCG<br>ACGTTCATCCAACAAGGCGGCGGCGGCGGAAACCGAAACCGCCGTCGTCAA<br>GCGGTTCCCGTCCTCAAAGCCGCCGTTCACACTCGCCGACCTCAAGAAGGCCATT<br>CCGCCGCACTGTTTCAAAAGATCCATCCCACGTTCCTTCTCCTACTTGGTTTTCGAC<br>CTCATCGTCGCCGCCGTCTTCTACCACATCGCCGCCACTTATTTCCCCCTCATCCCC<br>AAACCTCTCTCCTACGTGGCGTGGCCGGCCTACTGGTTCGTCCAGGGCAGCGTCCT<br>GACCGGTGTGTGGGTCATCGGCCACGAGTGCGGCCACCACGCATTTAGCGAGCAC<br>CAGTGGCTCGACGACCTGGTCGGGTTCATTCTCCACTCGGCCCTCCTCACCCCTTA<br>CTTCTCGTGGAAGATCAGCCACCGCCGCCACCACGCCAACACCTGCTCCCTCGAG<br>CGCGACGAGGTATATACTGTTGGGAACCGGACAAGAAGCAATATTCAGGGTTTAT<br>CGATCTGGCTGAGTATACATGGACAAACTATCCATCAACATCCAAGATAAAAAC

	AGAGCTATAACTTTCAACTTCCATGTGCAAAAATAGGGAGATGAATAAAATTTCA<br>GATAATTTAAACAAATAAATTATCTCCAAAATGAATAATATTGGTCTCAAGTCCA<br>ATCCAACCTGGAGAACTGGATCTTTCTACAAAGACGTTTTGCATTAGTCTTTTTTT<br>CTTTCTCCGATGTGATATTTGAGACTTAATTATTCACATTTTTCAACAGGTGTACAT<br>TCCGAGGAAGAAGTCTCAGTTGCGGTGGTGGTACTCGAGCTACCTGAACAACCCG<br>CCCGGCAGACTTTTGGCGCTGGCTTACACCATCCTCCTCGGCTGGCCTTCCTACTT<br>GACTTTCAACCTATCCGGTAGGGAATACAACGGTTTCGCCTGTCATTTCTACCCGA<br>TGTCCCCTATTTACAGCGACCGGGAGCGGGCCGAGGTATTCGCCTCCGATGTTGG<br>CTTGCTTGCTGTGTGCTTCGCGCTGTACAAACTTATCCTCGTCAAGGGAATGGCGT<br>GGGTTTTTTGCGTCTACGGGGCTCCGGTCATGGTGGTGAACGGATTCTTCATAACC<br>ATCACTTACTTGCACCACACTCATCTTGCGGTCCCGCGGTACGATTCCTCGGAATG<br>GGATTGGTTGAGGGGAGCCCTAGCAACGATGGATAGAGACTTTGGGTTATTGAAC<br>AGGGTGTTCCATAACGTTACAGATACTCATGTGACGCACCATCTGATCTCGACCA<br>TCCCTCACTACCATGCCATGGAAGCCAACAACGCGATTAGGCCCGTGTTGGGGGA<br>CTACTACCACATCGACAGGACGCCTGTCGTTAAGGCGTTGTGGAGGGAGGCTAAG<br>CAGTGTGTTTACATCGAGGCGGACGAAGGTGAAAATAACAAAGGCGTGTTCTGGT<br>TCAATACCAAGCTCTAA
FAD2f-1	ATGGTGTCCTCCGGCAAAACCATGAGCAACAAAACCACCACGAAGCGGCCGCCG<br>GTGTCAAAACCGCCGTTCACTCTGGCCGACATCAAGAGAGCCGTACCGCCACACT<br>GCTTCAAAAGATCCCTCGTCAAATCATTCGCCTACCTAGCCTACGACCTCACCGTC<br>ATCACAATCCTCTACCACATCGCCAACACATACTTCCACCTCCTCCCCAAACCTCT<br>CTCCTACGTGGCGTGGCCCGTCTATTGGGCTGCCCAATGCTGCTTTTTCGTCGCTTT<br>ATGGATGGTGGGCCACGACTGCGGCCATCATTCATTCAGCGACTATCAGTGGGTG<br>GACGACACCGTTGGATTCGTCGTCCACTCGTTCCTCCTCGCCCCTTACTTCTCGTGG<br>AAGCATAGCCACCGCCGCCACCACGCCAACTCCGGATCTCTAGAACGCGACGAG<br>TCGTTCGTCCCGAAAACCAAAGACAATATCACCTGGCACTTCAAGTACCTCGACC<br>ACCTACCGGGGCGGATCTTCTACGTGGTCTTCACTCTAACCCTCGGATGGCCGCTC<br>TACTTGATGTTCAACATCACCGGAAGGCCGTACAAGGACGGATTCGCCAGTCATT<br>TCTACCCCATGTCGCCTATCTACGAGGATCACGAGCGGTTCGGAATATTCCTCTCT<br>GATGTTGGCATGCTCGCTATGTGGTTCACACTCTACAAGCTTTCCGTGGCGTATGG<br>AGTCGGTTGGGTTCTTTGTGTGTATTTTATTCCGCTGGTTTTACAAAATGCGTTGTTT<br>GTCACGATCACTTACTTACACCACACGCATCTGAACTTGCCTCACTATGACTCTTC<br>GGGGTGGGACTGGATGAGGGGGTCGTTGACGACTGTGGATAGGGACTATGGGTTT<br>TTGAACAAGGTGTTGCATAACGTGACAGATACTCACGTGGCGCACCATCTGTTCA<br>CCCATATGCCTCATTACCACCAATCGGAAGCGACCAAGGCGTTCATTCCGGTTTTG<br>GGGGAATACTACCAGGTTGATCCGACTCCGTTCTACAAGGCGTTGTGGAGGGAGA<br>TGAAGCACTGTGTTTATATCGAGCAAGATGAGGATGCGGATTCTGATAATAACAA<br>AAAAGGCGTGTACTGGTACAAAACCAAGCTCTAA
FAD2f-2	ATGGTATCCTCCGACAAAACCACGAACAACAAAACCACCACGAAGCGGCCGCCG<br>CTGTCAAAACCGCCGTTCACCCTCGCCGACATCAAGAGAGCCGTACCGCCGCACT<br>GCTTCAAAAGATCTCTAGTCAAATCATTCGCCTACCTAGCCTACGACCTCACCGTC<br>ATCACCTTCCTCTACCACATCGCCAACACATACTTCCATCTCCTCCTCAAACCTCT<br>CTCCTACGTGGCGTGGCCCCTCTACTGGGCCGCTCAATGTTGCTTCTTCGTCGCTTT<br>ATGGATGGTGGGTCATGATTGTGGCCACCATTTGTTCAGCGACTATCAGTGGATTG<br>ACGACACTGTCGGTTTCATCATCCACTCGTTCCTCCTTGCTCCTTATTTCTCCTGGA<br>AGCACAGCCACCGCCGCCATCATGCCAACTCCGGATCTCTAGAACGCAACGAGT<br>CATTCGTCCCGAAAACCAAAGACAATATCGCCTGGCACTTCAAGTACCTCGACCA<br>CCTACCGGGGCGGATCTTCTACGTCGTTTTCACTCTGACCCTCGGGTGGCCACTAT<br>ACTTGATGTTCCACATCACTGGAAGGCCGTACAAGGATGGATTCGCCAGCCATTT<br>CTACCCCATGTCGCCGATCTATGAGGATCACGAGCGGTTCGGGATATTCCTCTCCG<br>ACGTTGGAATGCTCGCCATGTGGTTCACGCTCTACAAGCTCTCAATGGCGTATGG<br>AGTCGGTTGGGTTCTTTGTGTGTATTTTATTCCGTTGGTTTTACAAAATGCTTTGTTT<br>GTTACGATTACTTACTTGCACCACACGCATTTGAACTTACCTCACTATGACTCTTC

	GGGGTGGGACTGGATGAGGGGGTCGTTGACGACTGTGGATAGAGACTATTCGTTT<br>TTGAACAAGGTGTTGCATAACGTGACAGATACTCACGTGGCGCACCATTTGTTCA<br>CACATATGCCTCATTACCATCAATCGGAAGCGACCAAGGCGTTCATTCCGGTTTTG<br>GGGGAGTACTACCAGGCTGATCCGACGCCGTTCTACAAGGCGTTGTGGAGGGAG<br>ATAAAGCATTGTGTTTATGTTGAACAAGATGAGGATGCGGATTCCGATCAGAACA<br>AAAGAGGAGTGTACTGGTACAAAACCAAGCTGTGA
FAD2g-1	ATGAACAACAGAACCACCACGAAGCTGCTGCCGCTGTCAAAACCGCCGTTCACT<br>CTGGCCGACATCAAGAGAGCCGTACCGCCACACTGTTTCAAAAGATCCCTCGTCA<br>AATCATTCGCCTACCTAGCCTACGACATCACCGTCATCACAATCCTCTACCACATC<br>GCCAACACATACTTCTACCTCCTCCCCAAACCTCTCTCCTACGTGGCGTGGCCCGT<br>CTATTGGGCCGCCCAAAGCTGCTTCTTCGTCGCTGTATGGATGGTGGGTCACGATT<br>GCGGCCACCATTCATTCAGCGACTACCAGTGGGTGGACGACACTGTCGGATTCGT<br>CGTCCATTCGTTCCTCCTCACCCCTTACTTCTCGTGGAAGCACACCCACCGCAGCC<br>ACCACGCCAACAACGGATCTCTAGAACGCGACGAATCTTTCGTCCCCAAGACCA<br>AAGACGAAGTCAGGTGGCACTTCAAGTACCTCGACCACCTACCGGGGCGAATCTT<br>CTACGTGTTCTTTACTCTAACCCTAGGATGGCCTCTCTACTTGATGTTCAACATCAC<br>CGGAAGGCCGTACAAGGATGGATTCGCTAGCCATTTCTACCCCATGTCGCCTATG<br>TACGAGGACCATGAGCGGTTTGGAGTAGTCCTGTCCGACATGGGAATGCTCGCTA<br>TGTGGTTTACGCTCTACAAGCTCTCGGTGGCGTTCGGAGTCACTTGGGTTCTCTGT<br>GTGTACTTTATTCCACTAGTTTTACAAAACGCGTTGTTTGTTACGATCACTTACTTG<br>CACCACACGCATCCAAACGTGCCTCGTTATGACTCTTCGGGGTGGGGTTGGATGA<br>GGGGGTCGTTGGTGACCGTGGATAGAGACTATGGGTTTTTGAATAAAGTGTTCCA<br>TAACGTGACGGATACTCACGTGGCGCACCATCTGTTCACGCATATGCCTCATTACC<br>ACCAATTGGAAGCTACCAAGGCGTTCATCCCGATTTTGGGAGAGTACTACCAGGC<br>TGATCCAACACCGTTCTATAAAGCATTGTGGAGAGAAATGAAGCACTGTGTTTAT<br>GTCGAACAAGACAAAGATGCAAATGTCGATCAGAACAAAAGAGGTGTGTATTGG<br>TACAAAACAAAGTCGTGA
FAD2g-2	ATGGTATCCTCCGACAAAACCATGAACAACAAAACCACCACGAAGCTGCTGCCG<br>CTGTCAAAACCGCCGTTCACTCTCGCCGACATCAAGAGAGCCGTACCGCCACACT<br>GCTTCAAAAGATCCCTCGTCAAATCATTCGCCTACCTAGCCTACGACATCACAGT<br>CATCACAATCCTCTACCACATCGCCAACACATACTTCCACCTCCTCCCCAAACCTC<br>TCTCCTACGTGGTGTGGCCCCTCTACTGGGCTGCCCAATGTTGCTTCTTCGTCGCGG<br>TATGGATGGTGGCCCATGACTGTGGCCACCATTCATTCAGCGACTATCAATGGGT<br>CGACGACACCGTCGGTTTCATCATCCACTCGTTCCTCCTCGCCCCTTACTTCTCATG<br>GAAGCACACCCACCGCAGCCACCACGCCAACAATGGATCTCTAGAACGCGACGA<br>GTCTTTCGTCCCCAAGACCAAAGACGAAATCAGGTGGCACTTCAAGTACCTCGAC<br>CACCTACCGGGGAGAATCTTTTACGTGTTCTTCACTCTAACCCTAGGATGGCCTCT<br>CTATTTGATGTTCAACATCACCGGAAGGCCGTACAAGGACGGATTCGCCAGCCAT<br>TTCTACCCCATGTCGCCTATGTACGAGGACCACGAGCGGTTCGGAGTAGTCCTCTC<br>TGACATGGGAATGCTCGCCATGTGGTTCACGCTCTACAAGCTCTCAATGGCGTAC<br>GGAGTCAGTTGGGTTCTCTGTGTGTACTTTATTCCACTGGTTTTACAAAATGCGTTG<br>TTTGTCACGATCACTTACTTGCACCACACGCATCCGAACTTGCCTCGTTATGACTC<br>TTCGGGGTGGGGTTGGATGAGGGGGTCGTTGGTAACTGTGGATAGAGACTACGGG<br>TTTTTGAACAAAGTGTTGCATAACGTGACAGATACTCACGTGGCGCACCATCTGTT<br>CACGCATATGCCTCATTACCACCAATTGGAAGCTACCAAGGCGTTCATTCCGGTTT<br>TGGGGGAGTACTACCAAGCTGATGCAACGCCGTTCTACAAGGCATTGTGGAGGG<br>AGATGAAACACTGTGTTTATGTTGAGCAAGATGAGGATGCAGATTCCGACCAAA<br>ACAAAAGAGGTGTGTACTGGTACAAAACCAAGCCGTGA
FAD2h	ATGGTATCCAACAAAACCATAAACCGGCCGCCGTCCTCAAAACCGCCGTTCATCC<br>TCTCCGACATCAAGAAAGCAATCCCACCACACTGCTTCCGCAAATCCCTCCTCCG<br>ATCATTCTCCTACGTAGCCTACGACCTCGCCGTCATCGCAATCCTCTACCACATCG<br>CCACGTCATACTTCCACCTCCTCCCCAAACCTCTCTCCTACGTGGCCTGGCCGGCA<br>TACTGGGCCGCCCAGGGATCCCACTTCATCGCCGTCTGGGTCCTGGCCCACGAGT

	GCGGCCACCACGCCTTCAGCGACTACCAGTGGCTAGACGACGTCGTCGGATTCGT<br>CCTCCACTCCGCTCTCCTCTCGCCGTACTTCTCGTGGAAACACAGCCACCGCCGCC<br>ACCACTCCAACTCGGCATCTCTAGAACGCGACGAGCTGTACATCCCGAAGAAGA<br>AATCGGAGATTAGCTGGCATTACAAGTACCTCGACAACCCGCCGGGTCATCTTTT<br>CTACTTGGTGTTCACTCTCACTCTCGGATGGCCTCTCTACGTGATGTTCAACGTCTC<br>CGGCAGGGAATACGACGACGGATTCGCTAGCCATCTGTATCCTTTCTCCCCGATCT<br>ACAACGAACGGGAGAGATTCGGGATACTTTTGTCGGACGCTGGGATGCTTGCCAC<br>TTGGTTTGGTCTGTACAAGCTGTCCATGGTCAACGGACTGAGTTGGGTTGTTTGTG<br>TGTACGGGGTCCCGCTATTGGTGATGAATGGATTGCTTGTGACGATCACTTACTTG<br>CATCACACCCATCTTTCGCTCCCGCATTATGACTCGTCGGAGTGGGAGTGGATGA<br>GAGGTGCGCTAGCGACTGTGGATAGAGACTACGGGTTCCCGCTGAATAAGGTGAT<br>GCATCATATTACGGATACTCACGTGGTGCACCATTTGTTCTCGATGATTCCTCATT<br>ACCATGCGACGGAGGCTACAAATGCGATTAGGCCGATTTTGGGTGAATATTATCA<br>AGTTGATCCGACTCCGTTTGTCAAGGCGTTGTGGAGGGAGATGACGCACTGTGTTT<br>ACGTTGAGGCCGATGAGAAGAAACGAGGTGTGTTCTGGTATAAAACCAAGCTAT<br>GA
*FAD2b-2**	ATGGGTGCTGGCGGAAGAATGGCCGTGCCTCCATCGAACAAGGCGGACTCCGAA<br>ACCTTTAAGCGGTCTCCTTACTCAAAACCTCCCTTCACTCTTGGTGAGATCAAGAA<br>AGCCGTCCCTCCACACTGCTTCAAAAGGTCCATCCCCCGCTCGTTCTCCTACGTGG<br>CTTATGACCTCACCATAGCCGCCATCTTCTACTACATCGCCACCACTTACATCCAC<br>CTCCTCCCCCAATCCTCTCTCCTACGTGGCGTGGCCGATCTACTGGGCCTGCCAAG<br>GCTGCGTCCTCACTGGTGTCTGGGTCCTAGCCCACGAATGCGGTCACCATGCCTTC<br>AGCGACTACCAATGGCTCGACGACTTGGTCGGCTTTGTCCTCCACTCATGCCTCAT<br>GGTACCCTACTTCTCGTGGAAGCACAGCCACCGTCGCCACCACTCCAATACTGGG<br>TCCCTCGAACGAGACGAGGTTTTTGTCCCCAAGCAGAAATCAGCCATTGGCTGGC<br>ACTCAAAGTACCTCAACAACCCACCTGGCCGTGTGCTCACACTTGCAGTCACTCT<br>CACTCTCGGCTGGCCTTTGTACTTGGCATTCAACGTCTCTGGAAGGCCGTACGACC<br>GGTTCGCCTGCCATTACGATCCTAAATCCCCCATCTACAACGATCAGCGAGCGAA<br>CGGAGATATTCTTCTCCGATGCTGGCATCCTTGCTGTGAGCTTTGCGCTCTACAAG<br>CTTGCTGTCGCCAAGGGACTGGCTTGGGTGGTTTGTGTCTACGGAGTTCCACTCCT<br>TGTAGTGAATGGATTCCTTGTCTTGATCACTTTCTTGCAGCACACCCACCCATCATT<br>GCCGCACTACAAATCCTCCGAATGGGACTGGCTGAGAGGTGCTCTGGCGACCATG<br>GACAGAGACTACGGGTTTCTGAACACGGTGTTCCATAACATCACGGATACCCACG<br>TGGCGCACCACCTGTTCTCGACGATGCCTCATTACCATGCAATGGAAGCTACAAA<br>GGCGATCAAGCCGGTATTGGGAGAGTACTACCAATTCGACGGGACTCCATTCATC<br>AAGGCGATGTGGAGGGAGGCTAAGGAGTGTGTTTATGTCGAGCCCGACGAAGGT<br>GACCAGAACAAAGGCGTGTTCTGGTACAACAACAAGCTGTGA
*FAD2c-1**	ATGTTCTCATCCTCTGCCAGCTAAAAAATGCCGGCGCCGCCTTCATCATCCAACA<br>CGACCATGAAGCGATCGCCTCTCTCCAAAGCGCCGTACACCGTCTCCGACGTCAG<br>TCCAACCATTCCACCACACTGCTTCCAAAGGTCCCTCCTCCGATCATTCTCCCTAC<br>CTAACCTACGACCTCACCATCATCACAATCCTCTACCAAGTCGCCACCACTTACTT<br>CCACCTCCTCCGGCTCCCTCGCATCCTACGTGGCATGGCCGGCCTACTTCGGGCCG<br>GCCAAGGCTACTTCTTCGTCGCTGTATGGATGGTGGCCCACGGGGTGTGGCCATC<br>ACGCCTTCCAGCGACCAGCACTGGCTGGAAGACTCGTCGGATTCATCCTCCACTC<br>AGCCCCTCCTTTCACCTTACTTCTCATGGAAGCACAGCCACCGTCGCCACCATGCC<br>AACACGAGCTCCCTCGAACGTGACGAGGTGTTCGTCCCTAAACCCAAGTCCAAGC<br>TGAGCTGGCACTTCAAGTTCTTCAACAACCCGCCTGGCCGCGTCCTGCAGTTGGC<br>ATTTGCTCTGCTTCTTGGATGGCCTCTCTATTTGGCGATCAACATCGCCGGAAGGC<br>CGTACGAGAAATTCGCTAGCCACTTCGATCCGAGAAGCCCTATCTACAACGATCG<br>AGAGCGGATCGAGATATTCGCATCCGATGTTGGCGTGCTCTGCATGTGGTTCGCG<br>CTGTATAAAACTTGCTCTGGTGAACGGAGTTGGTTGGGTTGTTTGTGTGTACGGGA<br>TCCCGCTGTTGGTGATGAATGGATGGGTCGTTACGATCACTTACTTGCATCACACG<br>CATATAGCGTTGCCGCGGTATGATTCGTCCGAATGGGATTGGTTGAGGGTGCATTG

	GCACTGTGGATAGGGACTATGGTGTTTTGAACAAGGTGTTTCATAACATCACGGA<br>TACTCATGTGGCGCACCATTGTTTTCGGCGATGCCTCATTACCATGCGGCGGAAG<br>CGACCAGGCGATCAAGCCCGTTTTGGGCGAGTATTATCGGTGTGACCGGACTCCT<br>ATTATCAAGGCGTTATGGAGGGAGTTTAAGCACTGTATTTATGTCGAGTCAGATG<br>AGGATAAAGGCGTGTTTTGGTTCAATGACAAGTTGTAA
FAD3a	ATGAGCCCTCCAAACTCAATGAGTCCCGCCACCAACGGCAGCACCAATGGTGTG<br>GCTATCAATGGGGCGAAGAAGCTACTCGATTTCGACCCGAGTGCTGCTCCCCCTT<br>TCAAGATTGCAGACATCCGTGCTGCAATCCCGCCGCATTGTTGGGTGAAGAACCC<br>CTGGAGGTCACTCAGCTACGTCCTGAGAGACCTCCTGGTCATCCTCAGCTTCGCCG<br>TTGCGGCGACAAAGCTGGACAGCTGGACTGTCTGGCCTCTCTACTGGATTGCTCA<br>AGGAACCATGTTCTGGGCAGTCTTTGTTCTTGGACATGATTGGTAATTTCACATGA<br>TCTTTCTGGTAATGTGGGTTTTCTTTTCTTATTGAAAAAGATTAAAACTTTTTATCTG<br>GGCTGTTGCATGCAGTGGCCATGGGAGCTTCTCAGACAGTTGGTTGTTGAACAAC<br>GTGATGGGACATATACTCCATTCCTCAATCCTCGTACCTTACCATGGATGGTATTG<br>TAACTATTGTTCGATATTCGATTATGATTACTGTTCTTTCAGATGAAGAATCTGTAC<br>CCTAATTGTTTTTTGTTACCAGGAGAATTAGCCACAAGACCCATCACCAGAATCA<br>CGGCAATGTGGAGAAAGATGAATCCTGGGTTCCAGTAAGTTGACATGCAGTTTGC<br>TCTAAAATGCAGAGTCCTCTGTTTTTGTGTGTTCTTGTGCTTTAATGACGATGATA<br>ATGAAATTGAAATTTGTAATAGCTGCCGGAGAAGGTGTACAAGAGCTTGGATACC<br>GGCACCAAGTTCATGAGGTTCACCATCCCTCTCCCAATGTTTGCGTATCCTATCTA<br>CTTGGTAAGTAAACAGACTGACTCCAAAGTAGGAACTAATGACAATTTTGGACCC<br>GACCTGGTTTGGTTGACTCGGGTCGATATGTTTCGGGTGGGTAATTACCCGATCTG<br>GCGATGGGTGTGCGGCGGACATTGTCTTGCTCGTGGTCCACCCCGCTCCCAACCC<br>GCCCCATTCTTGACGAAAAAGATTTCGGAATATGTATCAACAGAAAAATCTAGTT<br>TTTATGTTACTAGTTTTCTGTATTTCCATGTTTTTTCCTCAATTCTAGCCGGAATTTG<br>AATTCAAACTGAAATCGGGTAATTCCGTCCATAACAAAACGGAATTGGGCAGCC<br>GTAATTAGTTGAAACTAGACCTCAATTTTGGCGGAATTGGACCCGGCCATTTTTTA<br>CGTTTGCAAAACGGAAAACGTTTTTCTTTTGTAAAGCGCAAAATGAAAAACGTAT<br>CTAGTGGAATTATTGGACCCATCTAGAATGGGTCCAATTCCACCCCAATTTCGGCT<br>CCAATTCATGCCCGGAAAACACTACTGTCATGCATTTTAATCTTGTATGGTTTTAC<br>CCCAATGGATGCAGCGATGGATCCGGACGATTTTTAAAATATTATCGGGTTAAAT<br>TTAAAAATATCTTAAAACTATAAGAAAAAAATAACCAATTTTAAAGAATAAAAG<br>AACTGGACACATATGACGGGTGTCGTGGATGGATGTACTTGTCCCGCTCTATTAA<br>AGGCTGATAATATACAGGTCAACGGTGAATGAAGGTTAGATGCGCTATTGGATTT<br>GAATCCGATATGAAATGATAATTTTGGACACGATCTGTTTTGGGTGGGTAATATTT<br>GATCTAGGGATGGCTCGTGCTCCAAACCGCACCAAAACCGCCTAATTCTCGACCA<br>AAAAGATTTTATGAATACATATCAACAGAAAAATCTAGTTTTCATGTTACTAGTTT<br>TATGTACAACAATATTAGGTGTCGTTTTCCCAGCCTTTTTCTTCAATTCCGGCCGGA<br>ATTCGCATTCAAACCGGAATTGGATGGAATCGGTATACCTCGTCACGGATGCATT<br>GTCAATTCCTAGTTAGTTTCATGGTTTTGAAACCAATCAATCTATTCTATATGGTTT<br>TGATTAACAGTGGAGGAGAAGTCCGGGGAAGAAAGGGTCGCATTTCAACCCATA<br>CAGTGACCTGTTCGCACCGAACGAGAGGACATCGGTCATGATTTCGACATTGTGC<br>TGGACAGCCATGGCCTTACTCCTCTGCTACTCATCGTTCATCTACGGCTTCCTTCCG<br>GTCTTCAAAATCTACGGCGTCCCTTATCTAATATTCGTGGCGTGGCTCGACATGGT<br>GACCTACCTTCACCACCACGGGTACGAGCAGAAGCTGCCGTGGTACAGAGGCAA<br>AGAGTGGAGCTACCTACGTGGAGGGCTGACGACCGTCGATCGAGATTACGGGGT<br>CATCAACAACATCCACCATGACATTGGCACCCATGTTATTCACCATCTCTTCCCTC<br>AAATGCCACACTATCACCTAGTCGAAGCGGTAAGGAGGTCTTGATTATTAACTTA<br>ATGTTTTTGTTGTTATAATTTGAGTCCGATTCTGGAGTCAGGGGATTTCCTTCTTGG<br>ATCCGATCCAGGATCAAGCTGGTCCCTTGAATTTCTATATGATCTTATATTAATTA<br>AGGATAATGTGGTCATATGTTTTTAAATATTTTTGTTTACCATCATTTTCGATCACC<br>GGAAAATGTCCTGAGCAGTTTTCCGGTCACTTTAACCTCCATTGACAAATTTTTTC<br>ACCCACATGATCACCCTAGCCGGGTTTACGTTTATTGAAAATTTTTATTTTTTGAA

	TTTTTTTTCGATGACCAACTGTACAACTTTGTATTGAAAGTTGTATGGATCATACA<br>AATGTGTATGTACAAAAGTATATTCTAAGTACTATACTAAGCATTACTTAGTATTA<br>CGTTTCTACAAACCTATAGAGAAATGCATACAATTTTGTATAGAACTTAGTATAC<br>ACGTAGCTGTGAAATGTCAATTTCCCTCCGTATTTTCAGAGACAAGACATGATTTT<br>TAGACTGGCAGATTTTTTTTATCGGATAGATTTCTCCAACTTCAGATTCGGACTGG<br>ATTATTAACTATATTATTCATCAACTCTGACGTTTGATGTTGCATGTGACAGACTC<br>AGGCAGCGAAGCACGTGCTGGGGAAGTACTACAGAGAACCGAAGAAATCAGGG<br>CCTTTCCCATTCCACTTGTTTGGGTACTTGGTGAGGAGCCTGGGCGAGGATCACTA<br>CGTTAGCGATACAGGCGACGTCGTTTTCTATCAATCTGACCCACATATTCCCAAGT<br>TCCCTACCAGTGCCACCACCAAGTCCAAATCTAGCTGA
FAD3b	ATGAGCCCTCCAAACTCAATGAGTCCCACCACCAACGGCAATGGTGTGGCTATGA<br>ATGGGGCGAAGAAGCAGCTCGATTTCGACCCGAGTGCTGCCCCCCCTTTCAAGAT<br>TGCAGACATCCGTGCTGCAATTCCGCCGCATTGCTGGGTGAAGAACCCCTGGAGG<br>TCGCTCAGCTACGTCCTGAGAGACCTCCTTGTCATCCTCAGCTTCGCCGTTGCGGC<br>GGCAAAGCTGGACAGCTGGACTTTCTGGCCTCTTTACTGGGTTGCTCAAGGAACC<br>ATGTTCTGGGCAGTCTTTGTTCTTGGACATGATTGGTAAACTAATTTCACATTTTCT<br>TTCTGGTAATGTGGGTTTTATTGAAAAAGATTAAAACTTTTTATCTGGGTTGTTGCA<br>TGCAGTGGCCATGGGAGCTTCTCAGACATCTGGTTGTTGAACAATGTGATGGGAC<br>ATATACTCCATTCCTCAATCCTCGTACCTTACCATGGATGGTATTGTAACTATTGTT<br>CAATATTAGATTATTGCTAGTTCTTCAGCTGAAGAATCCAAACCCTAATTTTCTTTT<br>TCTGAATATTGACCAGGAGAATTAGCCACAAGACCCATCACCAGAATCACGGCA<br>ATGTGGAGAAAGATGAATCCTGGGTTCCTGTAAGTTGACATGCAGTTTGCTGTAA<br>AAATGCAGAGTGCTCTGTTTTTTTGTGTTCTTGTGCTTTAATGGTGATAATAATGAA<br>ATTGTTGAAATGTAACAGCTACCGGAGAAAGTGTACAAGAGCTTGGATACCAGC<br>ACTAAGTTCATGAGGTTCACCATTCCTCTCCCAATGTTTGCTTATCCTATCTACTTG<br>GTAAGTAAAGAGACTGATAAGACTCCAAAGTAGGAATTAATGACAATTTTGGAC<br>CCGAGCTTCCGACTCGGGTCGATTTATTTCGGGTGGGTACTTACCCGATGCGGCGG<br>ACATTGTTTTGCTCGTGGTCCACCTCGCTCCCAACCCGCCCCATTCTTGACGAAAA<br>AGATTACGGAATATGTATCAACAGAAATATCTAGTTTTTATGTTACTAGTTTTCTGT<br>ATTTCCGTGTTTTTCCCCTCAATTCCGTCAGAAATTTGAATTCAAACTGACATTGGG<br>TAATTCTGTCCATAAGAGAACGGAATTGGGAAGCCGTAATTAGTTGGAATTAGAC<br>CTCGATTTCGGCGGAATTGGACCCGGCCATTTTTCGCGTCCGGAAAAGCGTTTCCA<br>GTGGAGTTAGACGCCCATCTAGAATGGGTCCAATTCCACCCCAATTTCGGGGCTA<br>CCTATTTTTAGTGTACATGCATTTTAGATCTTTTACGGTCTTACCCCGATGGATATT<br>CTTGTCGCGCTCTATTAAAAACTGACAATATACAGGTTGATTGTGAATGAAGATT<br>AGGTGCTGCACGCTCTTCGTGTTCCAACCCGCACCCAAACCGCCCCATTCTCGAC<br>CGAAAAGATTTTATGAATACATATCTACAGAAAAATCTAGTTGTCATGTCACTAG<br>TTTAATGTACAACAGTATAGGTGTCGTTTTCCGCGTCTTTTTCTTCAATTCCGGCTG<br>GAATTCGCATTCAAACAGGAATTGGATGGAATTGGTCCCGGATGCATAGTCATTT<br>CCCAGGCAGTTTCATGGTTTTATAACCAATCAATCTAATCTTATGCTTTTGATAAA<br>CAGTGGACGAGAAGTCCGGGGAAGAAAGGGTCGCATTTCAACCCATACAGCGAC<br>CTATTCGCACCAAACGAGAGGGCAGCGGTCTTGATTTCAACATTGTGCTGGACAG<br>CCATGGCCTTACTCCTCTGCTACTCATCGTTCATATACGGCTTCGCTCCGGTCCTCA<br>AAATCTACGGCGTACCTTATCTGATATTCGTGGCATGGCTCGACATGGTGACCTAC<br>CTTCATCACCACGGGTACGAGCAGAAGCTGCCGTGGTACAGAGGCAAAGAATGG<br>AGCTACCTACGTGGAGGGCTGACGACCGTTGATCGAGATTACGGGGTCATCAACA<br>ACATCCACCATGACATTGGCACCCATGTCATTCACCATCTCTTCCCTCAAATGCCA<br>CACTATCACCTTGTGGAAGCGGTAAACAATTTGATTATTAATTTACTGTTTTTGTTG<br>TTATAATTTGAGTCGGGAGATTTCCTTCCTAAATCCGATCCCTGGTCAATCTTGGC<br>CCTTGAATCTTCATATAATCTAAAAATCTAGATTAATCAGGAACAATATGATCAT<br>GTTGTTTAAACTAATTTTGTTGGACCATAACCTACCGCCAACTGATGGACCACCGT<br>CTCTGGTTACCGGACCCATCATTTCCGGTTACCAAGAAGTTTCTCGATCAGTTTTC<br>CGGTTACTTTGACCTGCGTTGAGGAAAATTCTTTCACCCACGTAAACACTGTCGTC

	AACTTTACGTTTCTGGAAAGTTTTTCCGATGATTGGCCGTACAATTTTGTACGAAG<br>AGTTGTACGGATCATATAAATGTGTATAAGTTTCTAGAAATCCGTACTGAAATAT<br>ATACATATTTGACTTTTGTATAAAGTGTAATACTAAATACTATACTAAGTGCTGTA<br>CTCAGTATGATACTTAGTACACACATTTGTATGACTATGAAATGTCAATTTTGCCC<br>TTATATTCTCAGCCGTTAGATCTAAGACACAGTTTTTATACGGCTGAAATTTGTGG<br>GGGCTTTGTAGATCGGATCCATAAGTCATTTCTTGGCTCAAGATTCGGACTCGATT<br>ATTAACTATATTATTCATCAACTCTGACGTTTGATGTTGCATGTGACAGACTCAGG<br>CAGCGAAGCACGTGCTGGGGAAGTACTACAGAGAGCCGAAGAAATCAGGGCCTT<br>TCCCATTCCACTTGTTTGGGTACTTGGTAAGGAGCCTGGGCGAGGATCACTACGTT<br>AGCGACACAGGCGACGTCGTTTTCTATCAGTCTGACCCACATATTCCCAAGTTCCG<br>TACCAGCAGTGCCACCACCAAGTCCAAATCCAGCTGA
FAD3c-1	ATGAGCCCTCCAAACTCCATGGGAATGGAAGCTGCTCATCCTACCGGCAACGGC<br>AATGGCGTCGCAGTCATGAACGGCGCCTCCGCTAACAAACCCGATTTCGATCCCA<br>GCGAGGCTCCTCCCTTCAAGATTGCCGACATCCGAGCCGCCATCCCCCCGCATTG<br>CTGGGTGAAGAATCCATGGAGGTCTCTTAGCTACGTCCTTAGGGACGCCGTCGTC<br>ATTCTCGCATTCGCAGCTGCCGCCCTCAAGCTCGACCTCTGGGCTGTTTGGCCGCT<br>CTACTGGATCGCTCAGGGCACCATGTTTTGGGCTGTCTTCGTTCTCGGCCACGATT<br>GGTAAGAGGAGGAGGTTTTTTTTTAAAATTTCAATCCGATGCTGTTTTGTTATATTA<br>TTGGTTGATATGATATATAAGATTAACCCTAGAGAAAAGTGAAAAGCATCCGCCA<br>TTGTTGAATGAATTGGAATTGGATGGTGCAGCGGCCATGGGAGTTTCTCGGATAG<br>CTGGTGGCTGAACAATGTGGTGGGGCATATTCTGCATTCTGCAATCCTTGTGCCTT<br>ACCATGGATGGTAAGAAAAGAATATGAACAGATCCTTTATCCTTACAACTGTAAA<br>ATGTAGGTCATTCAGCTGAACTAAAGTCACATTCTGTGTTGTTTAATTGATTGAAC<br>AGGAGAATTAGCCACAAAACACACCACCAGAATCATGGCAATGTTGAGAAAGAT<br>GAATCATGGGTTCCGGTGAGTTTTATTTCATTTTTTCTCTCGTCAATTGGCATCAGT<br>TTGTTCTGTTTCAATGCTTGATATGAATTGGGTGATATAATGCAGCTGCCGGAGAA<br>AGTATACAAGACCCTGGACACAAGCACCAAATTCATGAGGTTCACTATCCCTCTC<br>CCAATGTTTGCTTATCCTATCTACTTGGTAAGAACCACCCCACATTCTTACATCCTT<br>GCATGTTGTGTATTGGTTTCTGGTTGGATTTGGATTCAGATTTGGGGAATTGATTGT<br>GTGCCTTTGAAATGAATGAATGGTTACAGTGGACGAGGAGCCCAGGGAAGAAAG<br>GGTCCCATTTCAACCCCTACAGTGACCTGTTTGCCCCACAAGAAAGGAAATCAGT<br>CTTAATCTCTACCATCTCTTGGATTTCCATGGTCCTAATCCTCCTCTACGCCTCCTT<br>CCTTTTTGGTTTCCTCACTGTCTTCAAAGTCTATACCGTCCCTTACCTGGTAAAACT<br>ACTATCTCCAAATTCAACAATCTGTAAGCTTCATTTCATTTGCTTTGGCTCCTGAGT<br>TCCCTGTCAAGTTTCTCCTAGCAGTGTAACTTGTTATTTGATAACTTTGTGCCATCT<br>CACTATTGGCTGCATTTTACTATAATCAAACTGCTACATACGTTATTGCAACTTGTT<br>AATCCTTGGTACGAGGAATCCAATGAATAGGATGGTGATTAGCACAAACAAGGC<br>TGAAACTTTGAGCAATTTTGCAGATATTTGTGGCGTGGCTGGACATGGTGACATAC<br>CTGCACCACCACGGGCACGAAGAGAAGCTGCCGTGGTACAGAGGTCAAGAGTGG<br>AGCTACCTACGTGGAGGGCTGACAACCGTAGATAGAGATTACGGGATTATCAAC<br>AACATCCACCACGACATTGGCACTCACGTGATTCACCATCTGTTCCCTCAAATCCC<br>TCACTACCATCTCGTAGAAGCGGTACAGTAGTAGTAAACAAAACCCCATATTATT<br>ATTACTTACTGCAGTGATTTCTGACTCTATATTATTAAATATCACTTCTGGGTTTTT<br>GGCCGCGGGGGGGGGGGGGTTGTTTGGATCTTTCGAACTTATAATAATATGATA<br>TTATTATTAAAATGGTGGCAGACAAAGGCAGCAAAGTCGGTGCTTGGGAAGTACT<br>ACAGAGAGCCAAAGAAATCAGGACCATTCCCATTCCACTTGTTCGACAACTTAGT<br>GAGAAGCCTTGGCGAAGATCACTATGTTAGTGATGCAGGGGATGTCGTGTTCTAT<br>CAGTCTGACCCAGAAATCTTCAAGTTTTCCAAGTCCAAGTCAGCCTAA
FAD3c-2	ATGGGAATGGAAGCTGCTCATCCTACCGGCAACGGCAATGGCGTCGCCGTCATGA<br>ATGGCTCCGCCGCTAACAAACCCGATTTCGATCCCAGCGAGGCTCCTCCGTTCAA<br>GATTGCCGACATCCGAGCCGCCATCCCTCCGCATTGCTGGGTGAAGAATCCATGG<br>AGGTCACTCAGTTACGTCCTTAGGGATGCCGTTGTCATTCTCGCATTCGCCGCTGC<br>CGCACTCAAGCTCGACCTCTGGGCTGTTTGGCCGCTCTACTGGATCGCTCAGGGC

	ACCATGTTTTGGGCTGTCTTCGTTCTCGGCCACGATTGGTAAGAGGAGGAGTTGTT<br>TTTTTTAAATATTTATCCGATGCTGTTTTGTTATATTATTGGTTGATATGTAAGATTA<br>ACCCTAGAAAAAAGTGGAAAACGTCCGCCATTGTTGAATTGAATTGGAATTGGAT<br>GGTGCAGCGGCCATGGGAGTTTCTCGGATAGCTGGTTGCTGAACAATGTGGTGGG<br>GCATATCCTGCATTCTGCAATCCTTGTACCTTACCATGGATGGTAAGAAAAGAAT<br>ATGAACAGATCCTTTATCCTTGAAACGGTAAAATGTAGGTTATTCAGCTGAAACA<br>AAAGTGACATCCTGTGTTGTTCTTTATTGATTGAACAGGAGAATTAGTCACAAAA<br>CTCATCACCAGAATCATGGCAATGTTGAGAAAGATGAATCATGGGTTCCGGTGGG<br>TTTTATTTCTCTTCCTTACTTGTCAATTTGCCATCATTTGTCTGTTTCACTGATTGAC<br>ATGAATTGGGTGGGAAAATGCAGCTGCCAGAGAAAGTGTACAAGACTCTGGACA<br>CAAGCACAAAATTCATGAGGTTCACTATCCCTCTCCCAATGTTTGCTTATCCTATC<br>TACTTGGTAAGAACCACCCTACATTCTCACATCCTTGCATGTTGTGTATTGGTTTCT<br>GGTTGGATTTGGATTCAGATTTGGGAAATTGATTGTGTGCATATGAAATGAATGA<br>ATGGTTACAGTGGACAAGAAGCCCAGGGAAGAAAGGGTCCCATTTCAATCCCTA<br>CAGTGACCTGTTTGCCCCACAGGAAAGGAAATCAGTCTTAATCTCTACCATCTCTT<br>GGATTTCCATGGTCCTAATCCTCCTCTACGCCTCCTTCCTATTTGGCTTCCTCACAG<br>TCTTCAAAGTCTATACCGTCCCTTACCTGGTAAAAACTCCACCCTTGGTTTACTAG<br>CTTCAAATTCAACAATCTATCAGCTTCATTTCATTTGCTTTGGCTCCTAAGTTCCCT<br>CTTAAGTTTCTCGTAGCAGAGTAACTTGTCTATTTGTTAACTTTGTGCCATCTCCCT<br>ATTGGGTGCATTTCACTATAATCAAACTGCTACATGCATACTGGAATGTCCATATT<br>GTAATTTGCTATAATCCTTGGTACGAGAATCCAATGAATAGGATGGTGATTAGAA<br>CAAGAAAGACTGAAACTTTGAGCAAATTTTGCAGATATTTGTGGCGTGGCTGGAC<br>ATGGTGACATACCTGCACCACCACGGGCACGAAGAGAAGCTGCCGTGGTACAGA<br>GGCAAAGAGTGGAGCTACCTACGTGGAGGGCTGACAACCGTAGATAGAGATTAC<br>GGGATTATCAACAACATCCACCACGACATTGGCACTCACGTGATTCACCACCTCT<br>TCCCTCAAATCCCTCACTACCATCTCGTAGAAGCGGTACTGTAGTAAACAAAACC<br>CATATTATTATTACTTACTGCAGTGATTTCTGACTATGTTATTATTAAATATCAGTT<br>CTGGGTTTTGGCCTTTGGGGGGGTTGGTTTGGATCTTTGGCACTTATAATATTATTA<br>AAATGGTGGCAGACAAAGGCAGCAAAGTCGGTGCTTGGGAAGTACTACAGAGA<br>GCCAAAGAAATCAGGGCCATTCCCATTCCACTTGTTCGACAACTTAGTGAGAAGC<br>CTTGGCGAAGATCACTATGTTAGTGATGCAGGGGATGTCGTGTTCTATCAGTCTGA<br>CCCAGACATCTTCAAGTTTTCCAAGTCCAAGTCAGCCTAA
FAD3d-1	ATGGCGAGCTGGGTTCTATCAGAATGCGGCATAAAGCCACTCCCTTCCGCCTTCC<br>CTAAGCCCAGAACCGGAGCGCTTTCCCGAAATACCCTCCCCAAGCTCAGGTATTT<br>ACCCCCGAGGAGCAATCTCTCCGCCGCTGCCGACGGCGGCGCCTCAGATCTAAGC<br>CTTAACCTACCTAAATTATCCTCCGTAGAGAAGAAAAAGCCGCTTTTGGGACAGG<br>TGAGAGTTACTGCTCCGTTCAAGGTCGCTCCGGTGAAGGAGGAAGACGGAGAAG<br>GAGAGTCCAATTTCGACCCAGGAGCGCCGCCGCCGTTCAATTTGGCCGACATTCG<br>AGCTGCTATACCCAAGCATTGCTGGGTTAAAGATCCATGGAGGTCAATGGCTTAC<br>GTGGCGAGGGACGTGGCCGTTGTTCTCGGATTGGCAGCTGCCGCAGCTTATCTGA<br>ACAATTGGATCGTTTGGCCGCTGTATTGGGCGGCTCAGGGGACCATGTTCTGGGCT<br>CTCTTTGTTCTTGGCCATGACTGGTGAGTAAAGTTGTTAACTTTCTCTTTGGTCTTTT<br>ATGCTAATCTTTGTAATAATGGAAAATTGAAGAAAAATTCTCATGTTTGGCAGTG<br>GACATGGGAGCTTTTCAAGCAATCACAAGCTGAACAGTGTAGTTGGACACATTCT<br>CCATTCTTCAATTCTTGTGCCTTACCATGGATGGTAACTAAAGCTCTCTGCTTTCCT<br>CTGCCCATCTGTCATTTTGTTCTTCAATTTTGAAGCAATTTGAAACAAATTTTGTAT<br>CAATGTCTTTACAGGAGAATTAGCCATAGGACGCATCATCAGAACCATGGACATG<br>TTGAGAACGATGAGTCATGGCATCCTGTGAGTCTTTGTTTCTAAATCTTATCTATA<br>GGATTGATTCAGATGCTGATGTACTAATTTCTGTGACTTTTTCTGTTTTGTTTCTGTT<br>CTTGAAGTTGTCTGAGAAGATATTCAGGAGCTTGGATGCTACAACTCGAAGTATG<br>AGGTTCACATTGCCTTTCCCCATGCTTGCTTATCCTTTCTATCTGGTGAGTTTGTATC<br>TACAAGAAAGTTCCTTTACAAAATGTGGATTTAGTTAACAGAAGCTAAAAGGGTT<br>CTTTCTTCTTGTTCCGTAATGCAGTGGAACCGGAGTCCTGGAAAAAAGGGGTCTC

	ATTTCGACCCGAGTAGTGATTTGTTTGTGGCGAGTGAGAAGAAAGATGTGATCAC<br>TTCCACTGTTTGTTGGACAGCTATGGCTGCTTTGCTCGCAGGCTTGTCCTTTGTAAT<br>GGGTCCAATTCAGATGATCAAGCTATACGGAATTCCATATTGGGTATAGACAACA<br>TCTTTTGCCTTATCTTGCAAACTTGTTGTTTCAACTCTCAACCAACCACTCTGCCTC<br>TTGGATGTTTTTGTAGGGATTTGTTATGTGGTTGGATTTTGTAACTTACTTGCATCA<br>CCATGGTCACGACGATAAGCTTCCTTGGTACCGCGGCAAGGTAATCCATGTTCTTT<br>CCTCCTGTGTCCATCTATCTGGTCTTTCATGTTGAATCACTATATCCTAAAATCTTT<br>TCAATGTATAATTATTGATTCAAACCAAATGAAAAGTCAATTGTTTTGAGATGTCT<br>TTCTAGACTTTCAGTGAGATTTTGTTGAACTTATGCTTGGAACTTGGAACAGGAAT<br>GGAGTTACTTGAGAGGAGGGTTAACCACCATTGATAGAGACTATGGATGGATCA<br>ACAACATCCACCACGATATTGGAACCCATGTTATTCACCATCTCTTCCCACAAATC<br>CCTCACTACCACTTGATTGAAGCAGTAAGCAATAACTTTCCTTTTTCCCTTTAGTTC<br>CATATGCAATCTCTTCAATGGGGACACTGAGAAAAAGAAACAAACTTTGACCTG<br>AAAACAAATATCATGATGTTTCAGACGGAAGCTGCAAAGCCAGTGCTTGGGAAG<br>TACTACAAGGAACCAGCAAAATCAAAGCCGCTCCCTTTCCACCTAATCGGAGACT<br>TGATAAAGAGCTTGAAGAGAGACCATTATGTTAGTGACACCGGAGATGTCGTCTA<br>CTATCAAACTGACCCTGAACTCCAAAGACCCTCATCATCATCATGA
FAD3d-2	ATGGCGAGCTGGGTTCTATCAGAATGCGGCATAAAGCCACTCCCTTCCGCCTTCC<br>CCAAGCCCAGAACCGGAGCGCTTTCCCGGAATACCCTCCCCAAGCTCAGGTATTT<br>ACCCCCCAGGAGCAATCTCTCCGCCGCTGCCGACGGTGGCGCCTCAGATCTAAGC<br>CTTAACCTACCTAAATTATCCTCCGTAGAGAAGAAAAGGCCGCTTTTGGGGCAGG<br>TGAGAGTTACTGCTCCGTTCAAGGTCGCTCCGGTGAAGGAGGAAGACGGAGGAG<br>GAGAGTCCAATTTCGACCCAGGAGCGCCGCCGCCGTTCAATTTGGCCGACATTCG<br>AGCTGCTATACCTAAGCATTGCTGGGTTAAGGATCCATGGAGGTCAATGGCCTAT<br>GTGGCGAGAGACGTGGCTGTTGTTCTCGGATTGGCAGCTGCCGCAGCTTTTCTGAA<br>CAATTGGATTGTTTGGCCGCTGTATTGGGCGGCTCAGGGGACTATGTTCTGGGCTC<br>TCTTTGTTCTTGGCCATGACTGGTGAGTGAAAATTCTCAATTTTCGCTACTGTGATT<br>ACTAATCTGATGTAAAAAGTGGGTGGAAAGTTCTCATTTTTCCTTTTGCAGTGGAC<br>ATGGGAGCTTTTCAAGCAATCACAAGCTGAACAGTGTAGTTGGACACATTCTGCA<br>TTCTTCAATTCTTGTGCCTTACCATGGATGGTAAAAAGCTCTCTGCTTTCCTCTGCT<br>CATCCGCCATTTTGTTCTTCAAGTTTGAAGCAATTGAAAAAATAATTGTATCAATG<br>TCTTTACAGGAGAATTAGCCATAGGACGCATCATCAGAACCACGGCCACGTTGAG<br>AACGATGAGTCATGGCATCCTGTGAGTCTTTGTATCTAAATCTTAGCCATAGGGTT<br>GATTCAGATGCCGATGTACTAATTTCTGTGACTTTGTTTTGTTTCTGTTCTTGAAGTT<br>GTCTGAGAAGATATTCAGGAGCTTGGATGCTACAACTCGAAGTATGAGGTTCACA<br>TTGCCTTTCCCCATGCTTGCTTATCCTTTCTATCTGGTGAGTTTGTAGCTGCAAGTA<br>AGTTTCTTTACAAAATATGGATTTAGTTAACGAAGCTAAAAGGGTTATTTCTCCTA<br>ATGCAGTGGAACAGGAGTCCTGGGAAAAAGGGGTCTCATTTCGACCCGAGTAGC<br>GATTTGTTTGTGGCGAGTGAGAAGAAAGATGTGATCACTTCCACTGTTTGTTGGAC<br>AGCCATGGCTGCTTTGCTCGCAGGCTTGTCCTTTGTAATGGGTCCAATTCAGATGA<br>TCAAGCTCTACGGAATTCCATATTGGGTATAGACAACATCTTTTGCCTTATCTTAC<br>AGACTTGTTGTTTCAACTCTCAACCAACCACTCTGCCTCTTTGATGTTTTTGTAGGG<br>ATTTGTTATGTGGTTGGATTTCGTAACTTACTTGCATCACCATGGTCACGACGATA<br>AGCTTCCTTGGTACCGCGGCAAGGTAATCAATGTTCTTTGCTCCTGCGTCTATCTAT<br>CTAGTCTTTCATGTTTAATCACTATATCCTAAAATCTTTTCAATGTATAATTCTTGA<br>TTCAAACCCGATGAAAAGTCAATTGCTTTGAGATGTCTTTCTAGGCTTTCAGTGAC<br>ATTTTGTTGAACTTGTGCTTGGAACTTGGAACAGGAATGGAATTACTTGAGAGGA<br>GGGCTAACCACCATTGATAGAGACTATGGATGGATCAACAACATCCACCACGAT<br>ATTGGAACCCATGTCATTCACCATCTCTTCCCACAAATCCCTCACTACCACTTGAT<br>TGAAGCAGTAAGCACTAACGTCCCATTTTCCCTTTAGTTCCATACGCAATCTCTTC<br>AATGGGGAAACTGAGAAAAAGAAACGAACTTTGACCTGAAAACAAATATCATG<br>ATATTTCAGACGGAAGCTGCAAAGCCAGTGCTCGGGAAGTACTACAAGGAACCG<br>GCGAAATCAAAGCCGCTCCCTTTCCACCTAATCGGAGACTTGATAAAGAGCTTGA

	AGAGAGACCATTATGTTAGTGACACCGGAGATGTCGTGTACTATCAAACCGACCC<br>CGAACTCCAAAGACCCTCATCATCATCATGA
SAD2-1	ATGGCTCTCAAGCTCAACCCAGTCACCACCTTCCCTTCAACACGCTCCCTCAACA<br>ACTTCTCCTCCAGATCTCCTCGCACCTTTCTCATGGCTGCTTCCACTTTCAATTCCA<br>CCTCCACCAAGTAAGCATCTCCTCCTCCTCGGAATCTCCGCCGATTTCTTTTAAGC<br>GATTGATCGTAGATAAATTTGTCGGTTGCTTACCGTTCATCAAAATCTGCACGGTT<br>CGTTTCTTCTTCTGCGCCTAGATTGCATTATGTCATTGTTCGTTTTCCGATTTGACTG<br>ACCGACATAAATCAATTCCTTTGTGTTTCACGATTCTGGGTTTTGCGCTGTAATTGA<br>TTGTCAGTGTTTGCACAGGTTTCCCCTTCTCCTCCTCCGTCCATCAAATGCATGTTA<br>TTACCATTTCAATTTCAGTTTCCTTCTCTGAAATATCCGTCTCTGGGAAAATAAGTC<br>TCTGTATCTACTATCCTATCAGCTTGTTTAGGAGAGGTTCGATATTCGTTTACATAA<br>ACCAATTGGCTTACAGTCCTTGAACGTTCTAAATGTTGGTCGCGGTGATAATAGGT<br>TCTCAAAAGAGGTTTGTCTATGTTGTTTGGCAAAATCTTGTTTCTGTGAATCATGTT<br>TAAGGTCCTTGGAAGAATGACTAATGAGCTATGACATGATTACGACGTAGTAGTT<br>ATTGAACTGCTGATAATTCAATATAGGGGTAACTTTGTTGATTGTTTGGTCACAGG<br>GAGGCTGAGAAGCTAAAGAAGTCACATGGACCACCAAAAGAGGTGCATATGCA<br>AGTGACCCATTCCATGCCCCCACAGAAGCTGGAGATATTTAAGTCTCTGGAAGGT<br>TGGGCTGAGGATGTTCTATTACCGCACCTGAAGCCAGTTGAGAAATGCTGGCAGC<br>CACAGGATTTCCTGCCCGAACCTGAGTCGGATGGGTTCGAGGAGCAAGTGAAGG<br>AGCTCAGGGCAAGGGCCAAAGAACTGCCCGATGACTATTTTGTTGTGCTGGTTGG<br>GGATATGATCACCGAAGAAGCTCTGCCGACTTACCAGACAATGCTCAACACCCTT<br>GACGGGGTGAGGGACGAGACTGGAGCCAGCCTTACGCCGTGGGCAATCTGGACA<br>AGGGCGTGGACCGCTGAAGAGAATAGGCACGGTGACCTTCTCAACAAGTATCTA<br>TACCTCTCTGGAAGGGTGGACATGAGGCAAATTGAAAAGACCATTCAGTATCTCA<br>TCGGCTCTGGAATGGTATGTAATCACATACTTCATCCTTTTCTATTAATCTTTGCGT<br>GAACAAAATTCACTACACTGGTAGCAGCTGAAACTTTAGATGATTTTTTTACTGC<br>CTAGCTTCTATGAAACAAAACCACGTAAGTCAAATAGGGTTGACAATGAGTTCAA<br>GTGGCAAAATTTTTCTTATATACCAACTTCGAACCACTTTATATGACATACCAACT<br>CCTAGTTCGGTTAAAATTCCTCCGTCGAAGATATAATACTTGGATTGGTTAAATGA<br>ATTGTGAAAGGATACACGTGATGTGGTCTGGAATTAATTTGTTTGAATGATCAGTT<br>GGGTTCGGGGCGACAACTGTGAACTGGAACCACCCTAAGTAAATTTTCTTTCTGTC<br>CTACAAATTTGAGGTTCTCCTTGATCACCTTAGTCCATCTTAGGTTTGCCCGTTAGT<br>AAGATCTGCATTTAGCAGTTTGTCCTGGTATTTGATATCACTAGTATCTTTGTTTGA<br>TTCCCTAGCATCTCTGAAACCATCGGACAAGTAGGTGGTTTAGGACAAATTTGGTT<br>CATTGCGGCATTTTTTGTTTGTATCGCCGTATCATCTGGAAGAAGCAGACAGTTTT<br>GCAAAGTGGCATCAAGCTCAAGAAAGCAACGGCTAGAAGAAGTTCTACATCTGA<br>TGCTTTCCTTTTGTTTCTTTGTGTGCTTTTTGGACTTTGTTCTTTTTTCCTGTAGGATC<br>CAAAAACAGAAAACAACCCCTACCTCGGTTTCATCTACACCTCATTCCAAGAGAG<br>GGCAACGTTCATCTCCCACGGAAACACAGCCAGACTCGCCAAGGACCATGGGGA<br>CATGAAGCTGGCGCAGATCTGCGGGATCATCGCAGCAGACGAGAAACGGCACGA<br>AACCGCATACACCAAGATCGTCGAGAAGCTCTTCGAGATCGACCCTGACGGTAC<br>AGTGCTGGCACTGGCGGACATGATGAGGAAGAAGATATCGATGCCCGCCCACTT<br>GATGTACGATGGAGAAGACGACAACCTCTTCGACAATTACTCGTCAGTCGCTCAA<br>CGCATCGGGGTGTATACTGCCAAGGATTATGCCGATATCCTGGAGTTCCTGGTGG<br>GGAGGTGGAAAGTGGATGCTTTTACGGGGCTTTCCGGGGAAGGGAACAAAGCTC<br>AGGATTTTGTCTGCGGGCTTCCTGCGAGGATTCGAAAGTTGGAGGAGAGGGCTGC<br>GGGGAGGGCAAAGCAAACGTCGAAATCTGTCCCGTTCAGCTGGATCTTCAGCAG<br>AGAATTGGTACTCTAA
SAD2-2	ATGGCTCTCAAGCTCAACCCAGTCACCACCTTCCCTTCGACCCGCTCCCTCAACA<br>ACTTCTCCTCCAGATCTCCTCGCACCTTTCTCATGGCTGCTTCCACTTTCAATTCCA<br>CTTCCACCAAGTAAGTTCCCGTCACCATCTCCTCTTCCTCGGAATCTCCGCCGTTTC<br>ATTTAAGCGATTGATCGTAGAAAATCTGTCGGTTGCTTAGCGTTCATTCAAATCTG<br>CGCGGTTCGTTTCTTTTTCTTTCTTCAGACTGCCTCGTCTGCATTATGTTATTGTTCG

	TTTCCGATTTGACTAACCTACATAATCAATTCCTTTGTGTTTCACGAGTCTGGATTT<br>TGCGCTGTAATTGATTGTCAGCGTTTGCACAGGTTTCCATTTCTCCACCTCCGTCCA<br>TCAAATGCATGTTATTACCTACCAATTTCAGCGTCTTTCTCTGGAAATTTCTGTCTC<br>TGTATCTACTATCCTATTAGCTTGTTTGAGAGAGGTTCAATATTGGTTTGCATGAAC<br>CAAGTGGCTTACAATCCTTCAACGTTCTAAATGTTGGTCGCAGTAACAATAGGTTC<br>TCAAAAGAGGTTTTTCTATGTTGTTTGGCAAAATCTTGTTTCTGTGAATCATGTTAA<br>GGTCCTGGGAAGAATGATTAATGAGCTATGACATGATTAAGGCGTAGTAGTTATT<br>GAACTGCTGATAATTCAATATAGGGGTAACTTTGTTGGTTGTTTGGTGACAGGGAG<br>GCTGAGAAGCTAAAGAAGTCACATGGACCACCAAAAGAGGTGCATATGCAAGTG<br>ACCCATTCCATGCCCCCACAGAAGCTGGAGATCTTTAAGTCCCTTGAAGGTTGGG<br>CAGAGGACGTTCTGTTGCCGCACCTGAAGCCGGTTGAGAAATGCTGGCAGCCACA<br>AGATTTCCTGCCCGAACCCGAGTCGGATGGGTTCGAGGAGCAAGTGAAGGAGCT<br>CAGGGCAAGGGCTAAAGAACTCCCCGATGACTATTTTGTTGTGCTGGTTGGGGAT<br>ATGATCACCGAAGAAGCTCTACCGACTTACCAGACAATGCTCAACACCCTTGACG<br>GGGTGAGGGACGAGACTGGAGCCAGCCTTACGCCGTGGGCAATCTGGACAAGGG<br>CGTGGACCGCTGAAGAGAATAGGCACGGTGACCTTCTCAACAAGTATCTTTACCT<br>CTCTGGAAGGGTGGACATGAGGCAAATTGAAAAGACCATTCAGTATCTCATCGGC<br>TCTGGAATGGTATGTACTCACATCCTATCTGCTCCTTTATCCTTTTCCATTAATCTTT<br>GATTGAACAAAATTCAATAAACTGGTAGCTGAAACTTTAGATGATTTGTTATAAC<br>TGCCTAGCTTCTATGAGAAAACCACTGAAGTCAAATAGGTTTGACAATGGGTTTA<br>AATGGAAAAAGTTTCATATACCATCTTCCATCTATTTTACATGACATACCAACTTC<br>TACTTCGGAGAAAATTCGCCGTGGATAATCATATTATTGAAGATATAGTACTTAGT<br>AGATTGGTTAGATGAACTGTTAAACAATACATGTGATGTCGTGTGCAATTAATTTG<br>TGTAAATGATTAGCTGGGTTCGGGACGACAAATGTGAACTGGAACCCTAGTAAAC<br>TATGAATTGAGGTTGTCCTTCATCACTTTATTCTGTCCTGGGTTTGTTTGCCTGTTTG<br>CAAGATCTGCATGTAGCAGTTTGTCCTGGTATTTGCTACCAGTGGTATCTTTGTTTG<br>ATTCCCTAGCATCTCTGAAAACATCGGACCAAGTATCTGGTTAGGACAAATTTGG<br>TTCATTGCGGCATTTTTTGTTTGTATCGCTGTATCGTCTGGAAGAAGCAGACAGTTT<br>TGCAAAGTGGCATCAAGCTCAAGAAAGCAACGGCTAGAAGAAGTTCTACATCTG<br>ATGCGTTCCTTTTGTTTCTTTGTGTGCTTTTTGGACTTTGTTCTTTTTGCCTGTAGGAT<br>CCAAAAACAGAAAACAACCCCTACCTCGGTTTCATCTACACCTCATTCCAAGAGA<br>GGGCAACGTTCATCTCCCACGGAAATACGGCCAGACTCGCCAAGGACCACGGGG<br>ACATGAAGCTGGCGCAGATCTGCGGGATCATCGCAGCAGACGAGAAGCGGCACG<br>AAACAGCATACACCAAGATCGTCGAGAAGCTCTTCGAGATCGACCCTGACAGTA<br>CAGTGTTGGCTCTGGCGGACATGATGAGGAAGAAGATATCGATGCCCGCACACTT<br>GATGTACGATGGAGAAGACGACAACCTCTTCGACAATTACTCGTCGGTCGCTCAA<br>CGCATCGGGGTGTATACTGCCAAGGATTATGCTGATATCCTGGAGTTCCTGGTGG<br>GGAGGTGGAAAGTGGATGCTTTTACGGGACTTTCCGGGGAAGGGAACAAAGCTC<br>AGGAGTTTGTCTGTGGGCTTCCAGCGAGGATTCGAAAATTGGAGGAGAGGGCTGC<br>GGGGAGGGCAAAGCAAACGTCGAAATCTGTCCCATTCAGCTGGATCTTCAGCAG<br>AGAATTGGTACTCTAA
SAD3-1	ATGCAAGCCCCCCACCACGTCAGCGCCCACCGTCCAGCATGGATGCCACGTGGTG<br>GCCGAACCACCTCCACCACCATCATGCACCTCTGCCCTCCACGCGCCGCCATCAA<br>AACTGCTCCGCCGCCTCCCCCTCCGCTCACCCTGAAACACCAACACAAAACCCAC<br>ACAATGCCGGCGGAGAAAATCGAGCTTTTCAAGTCCCTCGAGGATTGGGCCTCCG<br>AGAATGTCCTCCCACTTCTCAAGCCCGTCAATAAATGCTGGCAGCCTCAGGACTT<br>CCTCCCCGACCCGTCCCTCTCCGTCGCTGATTTCTCCGACGAGGTAATGAACAATT<br>GCGTCTCAATACCTTATATAAGCTACACTTTTACAGGGATTTTGAGCTAATATTTG<br>ACAAATTCATCAGTTACGTTTTTTGTCATTACGGATTAGTCATTGTAGTTTTGTCAT<br>TTGTTCATATAGCTACTCATTTTTCAGTTCATCAAATTCTCATTTTCTATAGTAAGA<br>TCATTTGGAAGGGTTTTGGAGAGTAATTTCTTGGTCCCACATGATTTGATCGAAAT<br>ATCATTTTAAAATACAAAAATAAATTGGGATTTGAAATCACTTCGATAGGCTAGG<br>TTCCCCAAAAGGTGATTTCAGGCTCCCAAAAGGTGATTTCAAATTCATTTTATGGA

	CTTGAGATTTGCAAAATCTCAAGTAATTTGAATAGTAAATGATCAAATTATCTTAT<br>TGAGTTTAAGTGAACGAGATAAATTAAGAGGTGCATTAGTCATTTAAAAAATCAA<br>TTTTTTTTTTTAATTTTCAAAACTATTTTGTGATTGACCGTTAAACCAGTTCGGTTTG<br>ATACTGATTTTGAGTTTTGACTGACAGGTGAGGTTGTTGAGGGAAAGGACGGCGG<br>AGCTACCGGACGAGTACTTCGTCGTCTTGGTGGGTGACATGATAACGGAGGATGC<br>GTTACCGACGTACGAGACCATGATCAACACACTTGACGGCGTTAGGGACGAGAC<br>GGGCGCCAGTCAAAGCCCATGGGCCTTGTGGACCCGGGCCTGGACCGCCGAGGA<br>GAATCGCCACGGCGATCTGCTTCGGACCTTTCTGTACCTATCGGGTCGGGTCGACA<br>TGAGGATGGTAGAAAGGACTGTTCAGTACCTCATTGGATCTGGCATGGTAAGTGT<br>AGAATAGCCCTTTCGGTATTCCGAACCGGTTTGATCCGTCAGTGGGATCTTTCGTT<br>CGATTAATCGGGTGATGAGTATATGCCAAACTGGCACTAATTTGTCACTTGATTTG<br>ATAGTACATTTTATAAGTTAGTGTTAGCTAGTTACATTGTTTGAGAATGTCACGAA<br>TTCGAAGGAGAAAAAAACCAAAATGCCACTTCGATAATTTATTTTACATGATATA<br>GTCATTGAACCTTCAATCTTTTTACAAATCGGATCCAAATTTGTGTACGCAGTGGT<br>CACTCAACTGTCATTTTAGAATAGGAATAGCATTTTAACAAGTGATTTACTCGGGT<br>CGGGTAACATTACAGGACCCGGGCACAGAGAACAACCCGTACTTGGGGTTCGTG<br>TACACGTCATTCCAGGAGCGAGCCACATTCATATCGCACGGCAACACGGCTCGGC<br>TAGCCAAGGAGGGAGGGGATCCGGTACTCGCACGGATATGTGGCACCATAGCAT<br>CGGACGAAAAGCGACACGAGAACGCCTACACGCGCATCGTGGAGAAGCTCCTAG<br>AGGTGGACCCGAACGGTGCGGTTTGTGCGGTGGCCGACATGATGCATAAGAAGA<br>TCACAATGCCGGCGCACCTGATGTACGACGGGGAGGACCCGAAGCTGTTCGAGC<br>ACTTCTCATCGGTGGCTCAGCGGCTGGGGGTGTACACCGCGCAGGACTACGCGGA<br>CATACTCGAGTTCTTGATCGGACGGTGGGGGCTGGACAAGGTGGAGCAAGGGCT<br>GGACGGTGAAGGGAGGCGGGCGCAGGATTTCGTGTGTGGGCTGCCACGGAGGAT<br>CATGCGGCTCCAGGAGCGAGCCGATGAGAAGGCTAAGAAGATGAAGAAGCCGG<br>AAGCAGTCAAGTTTAGCTGGATTTTCAAACGGCAAGTCGTTTTGTGA
SAD3-2	ATGCACCTCTGCCCTCCACGCGCCGCCATCAAAACTGCTCCGCCGCCGCCTCCTC<br>CGCTCACCCTGAAACACCAACACAAAACCCACACAATGCCGGCGGAGAAAATCG<br>AGCTTTTCAAATCCCTCGAGGATTGGGCCTCCGTCAATGTCCTCCCACTTCTCAAG<br>CCCGTCGACAAATGCTGGCAGCCTCAGGACTTCCTCCCCGACCCGTCCCTCTCCGT<br>CGCTGATTTCTCCGACCAGGTAATGAACCATTGTGTTCCAATAACTCGTATTGTAA<br>TGTTACTCGTATTTTGAGCTAATATTTGATAAATTCGTCACTTAACTTTTTTGTCTTG<br>ACGGATTAGTCATTGTAGTTTTATTATTTGTTCGATTTTCAGTTAAATAATTCTCATT<br>TTTTATAGTAAGATCATTTGGAAGGAATTTGGAGAGTAATTTCATGGTCCTGGGTT<br>GATTTGATCGAAATATCGCGTTGGAACACGAAAATAAAGGTGGATTTGAGTAGA<br>AAATAACCAAATTGACTTGTTGAAATTGTATTGTTGAGTGAACAGGGGGTTTATTA<br>GTCATTTACAAAATTAAAATGTTTCTTTTAATGTCCAAAACTCAACTAAACACTGA<br>TTTTGAGATTGGCCGTTAAACCAGTTCGGTTTGATAAATACTGATTTTGAGTTTTGA<br>CTGACAGGTGAGGTTGTTGAGGGAAAGGACGGCGGAGCTACCGGACGAGTACTT<br>CGTCGTCTTGGTGGGTGACATGATAACGGAGGATGCGTTACCGACGTACGAGACC<br>ATGATCAACACCCTTGACGGCGTTAGGGACGAGACGGGCGCCAGTCAAAGCCCA<br>TGGGCCCTATGGACCCGAGCCTGGACCGCCGAGGAGAATCGCCACGGGGATTTG<br>CTCCGGACCTTTCTGTACCTCTCGGGTCGGGTCGACATGAGGATGGTTGAAAAGA<br>CTGTTCAGTACCTCATTGGATCTGGCATGGTAAAGTGCAGAATAGTCCTTTTTGTA<br>TTCCGAACTGGTTTGATCGGTTGTTTGGTTCGTTCGTTTGTTAATTGGGTGATGAGT<br>ATGCGAAATTGTCACTAAATTATCACTTCATAGTACATTTATAAGCTAGTGTTTAG<br>TAACAATAGTTTAAGAATGTTACAAATTTGAACCGGGGAAAAACCAAAATGCCA<br>CTTGGATAATTTATTTTGCATGATATAGTCATTGAACCTTTAATCTTCTTACAAATC<br>GGATCCTAATTTGTGTACACATTGGTCACTCAACTGTCATTTTAGAATAGGAATAA<br>CATTTTAACAAGTGATTACTCGGGTCGGGTAACAATTGCAGGACCCGGGCACGGA<br>GAACAACCCGTACTTGGGGTTCGTATACACGTCATTCCAGGAACGAGCCACATTC<br>ATATCGCACGGCAACACGGCTCGGCTAGCCAAGGAGGGAGGGGATCCGGTACTT<br>GCACGAATATGCGGCACCATAGCATCGGATGAGAAGCGACACGAGAACGCCTAC

	ACGCGCATCGTGGAGAAGCTCCTCGAGGTGGACCCGAACGGTGCGGTTTGTGCCGTGGCCGACATGATGCGTAAAAGATCACAATGCCGGCGCACCTGATGTACGACGGGGAGGACCCAAAGCTGTTCGAGCACTTCTCGGCGGTGGCGCAGCGGCTCGGGGTGTACACGGCGCAGGACTACGCGGACATACTGGATTTCCTGATCGGACGCTGGGGGCTGGACAAGGTGGAACAAGGGCTGGACGGTGAAGGGAGGCGGGCGCAGGATTTCGTGTGTGGGCTGCCGCAGAGGATCATCCGACTCCAGGAGCGAGCCGATGAGAAGGCTAAGAAGATGAAGAAGCCGGAAGCCGTCAAATTTAGCTGGATCTTCAAACGGCAAGTCGTCTTGTGA
**CDC Bethune**	
FAD2a-1 (wrong sequence)	ATGGGTGCCGGTGGCAGAATGTCAGTGCCTCCATCATCCAAACCTATGAAGAGGTCTCCTTACTCAAAGCCACCATTCACGCTCGGTGAGCTCAAGAAGGCCATCCCTCCACACTGTTTCAAACGCTCAATCCCCCGATCGTTCGCCTACGTGGCGTACGACCTCACCATTGCAGCAATCTTCTACTACATCGCCACCACTTACTTCCACCTCCTCCCTAGCCCTCTCAACTACCTCGCCTGGCCGGTCTACTGGGCCTGCCAGGGCTGCATCCTCACTGGAGTATGGGTGTTGGCTCACGAATGCGGTCACCATGCCTTCAGCGACTACCAGTGGCTCGACGACATGGTTGGCTTCGTCCTCCATTCGTCCCTCCTTGTTCCTTACTTCTCCTGGAAGCACAGCCACCGCCGCCACCATTCAAACACGGGGTCGCTTCTCCGGGAGACCATATGACCGGTTCGCATGCCATTTCGACCCTCACGGTCCGATTTACAATGATCGCGAGCGTATGGAGATATACCTATCCGACGCAGGGATATTCACCGTGTGCTACATCCTATACAGACTCGTCCTCACGAAAGGACTCGTTTGGGTCGTGTCCATTTACGGAGTCCCACTATTGATAGTGAATGGATTCCTAGTCCTCATCACTTTCTTGCAGCACACGCATCCTTCTCTTCCGCACTACAAGTCCTCCGAATGGGACTGGCTGCGAGGCGCCCTCTCGACCGTGGATCGAGACTACGGGTTACTCAACACCGTGTTCCACAACATCACCGATACACATGTCGCGCACCATCTCTTCTCCACGATGCCTCATTACCACGCGATGGAGGCTACCAAGGCGATCAAGCCGGTTCTCGGGGAGTATTACCAGTTCGATGGGACTCCCTTTGTGAAGGCCATGTGGAGGGAGGCAAAGGAGTGCATCTATGTCGAGCCGGATGAAGGCGACCCCAGCCAAGGCGTGTTCTGGTACAACAACAAGCTGTGA
FAD2a-2 (wrong sequence)	ATGGGTGCCGGTGGCAGAATGTCAGTGCCTCCATCATCCAAACCTATGAAGAGGTCTCCTTACTCAAAGCCACCATTCACGCTCGGTGAGCTCAAGAAGGCCATTCCTCCACACTGTTTCAAACGTTCAATCCCCCGATCGTTCGCCTACGTGGCGTACGACCTCACCATTGCAGCAATCTTCTACTACATCGCCACCACTTACTTCCACCTCCTCCCTAGCCCTCTCAACTACCTCGCCTGGCCGGTCTACTGGGCCTGCCAGGGCTGCATCCTCACTGGAGTATGGGTGTTGGCTCACGAATGCGGTCACCATGCCTTCAGCGACTACCAGTGGCTCGACGACATGGTTGGCTTCGTCCTCCATTCGTCCCTCCTTGTTCCTTACTTCTCCTGGAAGCACAGCCACCGCCGCCACCATTCAAACACGGGGTCGCTTCAGCCACCGCCGCCACCATTCCAACACGGGATCGCTTGATCGTGATGAGGTGTTTGTCCCCAAGCAGAAGGCCGAAATCGGGTGGTACTCCAAGTACCTTAACAACCCACCTGGCCGTGTGATCACATTGGCCGTCACATTAACGCTCGGTTGGCCTCTGTACTTGGCATTCAACGTCTCCGGGAGACCATATGACCGGTTCGCATGCCATTTCGACCCTCACGGTCCGATTTACAATGATCGCGAGCGTATGGAGATATACCTATCCGACGCAGGGATATTCACCGTGTGCTACATCCTATACAGACTCGTCCTCACGAAAGGACTCGTTTGGGTCGTGTCCATTTACGGAGTCCCACTATTGATAGTGAATGGATTCCTAGTCCTCATCACTTTCTTGCAGCACACGCATCCTTCTCTTCCGCACTACAAGTCCTCCGAATGGGACTGGCTGCGAGGCGCCCTCTCGACCGTGGATCGAGACTACGGGTTACTCAACACCGTGTTCCACAACATCACCGATACACATGTCGCGCACCATCTCTTCTCCACGATGCCTCATTACCACGCGATGGAGGCTACCAAGGCGATCAAGCCGGTTCTCGGGGAGTATTACCAGTTCGATGGGACTCCCTTTGTGAAGGCCATGTGGAGGGAGGCAAAGGAGTGCATCTATGTCGAGCCGGATGAAGGCGACCCCAGCCAAGGCGTGTTCTGGTACAACAACAAGCTGTGA
FAD2b-1	ATGGGTGCTGGCGGAAGAATGGCCGTGCCTCCATCGAACAAGGCGGACTCCGAAACCTTTAAGCGGTCTCCTTACTCAAAACCTCCCTTCACTCTTGGTGAGATCAAGAAAGCCGTCCCTCCACACTGCTTCAAAAGGTCCATCCCCCGCTCGTTCTCCTACGTGG

	CTTATGACCTCACCATAGCCGCCATCTTCTACTACATCGCCACCACTTACATCCAC<br>CTCCTCCCCAATCCTCTCTCCTACGTGGCGTGGCCGATCTACTGGGCCTGCCAAGG<br>CTGCGTCCTCACTGGTGTCTGGGTCCTAGCCCACGAATGCGGTCACCATGCCTTCA<br>GCGACTACCAATGGCTCGACGACTTGGTCGGCTTTGTCCTCCACTCATGCCTCATG<br>GTACCCTACTTCTCGTGGAAGCACAGCCACCGTCGCCACCACTCCAATACTGGGT<br>CCCTCGAACGAGACGAGGTTTTTGTCCCCAAGCAGAAATCAGCCATTGGCTGGCA<br>CTCAAAGTACCTCAACAACCCACCTGGCCGTGTGCTCACACTTGCAGTCACTCTC<br>ACTCTCGGCTGGCCTTTGTACTTGGCATTCAACGTCTCTGGAAGGCCGTACGACCG<br>GTTCGCCTGCCATTACGATCCTAAATCCCCCATCTACAACGACCGCGAGCGAACG<br>GAGATATTCTTCTCCGATGCTGGCATCCTTGCTGTGAGCTTTGCGCTCTACAAGCTT<br>GCTGTCGCCAAGGGACTGGCTTGGGTGGTTTGTGTCTACGGAGTTCCACTCCTTGT<br>AGTGAATGGATTCCTTGTCTTGATCACTTTCTTGCAGCACACCCACCCATCATTGC<br>CGCACTACAAATCCTCCGAATGGGACTGGCTGAGAGGTGCTCTGGCGACCATGGA<br>CAGAGACTACGGGTTTCTGAACACGGTGTTCCATAACATCACGGATACCCACGTG<br>GCGCACCACCTGTTCTCGACGATGCCTCATTACCATGCAATGGAAGCTACAAAGG<br>CGATCAAGCCGGTATTGGGAGAGTACTACCAATTCGACGGACTCCATTCATCAA<br>GGCGATGTGGAGGGAGGCTAAGGAGTGTGTTTATGTCGAGCCCGACGAAGGTGA<br>CCAGAACAAAGGCGTGTTCTGGTACAACAACAAGCTGTGA
FAD2b-2	ATGGGTGCTGGTGGAAGAATGGCTGTGCCTCCATCGAACAAGGCGGACTCGGAA<br>ACCTTTAAGCGGTCTCCATACTCAAAACCTCCATTCACACTTGGTGAGATCAAGA<br>AAGCCATCCCTCCACATTGCTTCAAAAGGTCCATCCCCCGCTCATTCTCCTATGTG<br>GCTTATGACCTCACCATTGCCGCCATCTTCTACTACATCGCCACCACTTACATCCA<br>CCTCCTCCCCAATCCTCTCTCCTACGTTGCGTGGCCGATCTACTGGGCCTGCCAAG<br>GCTGCGTCCTCACCGGTGTCTGGGTCCTAGCCCACGAATGCGGTCACCACGCCTTC<br>AGCGACTACCAATGGCTTGATGACTTGGTCGGCTTTGTCCTTCACTCATGCCTCAT<br>GGTCCCCTACTTCTCGTGGAAGCACAGCCACCGTCGCCACCACTCCAACACCGGG<br>TCCCTTGAACGAGATGAGGTTTTCGTCCCCAAGCAGAAATCGGCCATCGGCTGGC<br>ACTCAAAGTACCTCAACAATCCACCTGGCCGTGTGCTCACACTTGCAGTCACTCT<br>CACTCTCGGCTGGCCTTTATACTTGGCATTCAACGTTTCCGGAAGGCCGTACGACC<br>GGTTCGCCTGCCATTACGATCCTAAATCCCCTATCTATAACGACCGCGAGCGAAC<br>TGAGATCTTCCTCTCCGATGCCGGCATCCTTGCTGTGAGCTTTGCACTCTACAAGC<br>TTGCTGTCGCCAAGGGACTGGCTTGGGTGGTTTGTGTCTATGGAGTTCCACTCCTT<br>GTAGTGAATGGATTTCTTGTCTTAATCACTTTCTTGCAGCACACCCACCCATCATT<br>GCCGCACTACAAATCCTCCGAATGGGACTGGCTGAGAGGTGCTCTGGCGACCATG<br>GACAGAGACTACGGGTTTCTGAACACGGTGTTCCATAACATCACAGATACCCATG<br>TGGCGCATCACCTGTTCTCGACGATGCCTCATTACCATGCGATGGAAGCTACCAA<br>GGCGATCAAGCCGGTATTGGGAGAGTACTACCAGTTCGATGGACTCCATTCATC<br>AAGGCGATGTGGAGGGAGGCTAAGGAGTGTGTCTATGTTGAGCCTGACGAAGGT<br>GACCAGAACAAAGGCGTGTTCTGGTACAACAACAAGCTGTGA
FAD2c-1	ATGTTCTCATCCTCTGCAACTAAAATGCCGGCGCCGCCTTCATCATCCAACACGA<br>CCATGAAGCGATCGCCTCACTCAAAGCCGCCGTTCACCGTCTCCGACGTCAAGAA<br>AGCCATTCCACCACACTGCTTCCAAAGGTCCCTCCTCCGATCATTCTCCTACCTAA<br>CCTACGACCTCACCATCATCACAATCCTCTACCAAGTCGCCACCACTTACTTCCAC<br>CTCCTCCCGACACCTCTCTCATCCTACGTGGCATGGCCGGCCTACTGGGCCGGCCA<br>AGGCTGCTTCTTCGTCGCTGTATGGATGGTGGCCCACGAGTGTGGCCATCACGCCT<br>TCAGCGACCAGCACTGGCTGGAAGACTCGGTCGGATTCATCCTCCACTCAGCCCT<br>CCTTTCACCTTACTTCTCATGGAAGCACAGCCACCGTCGCCACCATGCCAACACG<br>AGCTCCCTCGAACGTGACGAGGTGTTCGTCCCTAAACCCAAGTCCAAGCTGAGCT<br>GGCACTTCAAGTTCTTCAACAACCCGCCTGGCCGCGTCCTGCAGTTGGCATTTGCT<br>CTGCTTCTTGGATGGCCTCTCTATTTGGCGATCAACATCGCCGGAAGGCCGTACGA<br>GAAATTCGCTAGCCACTTCGATCCGAGAAGCCCTATCTACAACGATCGAGAGCGG<br>ATCGAGATATTCGCATCCGATGTTGGCGTGCTCTGCATGTGGTTCGCGCTGTATAA<br>ACTTGCTCTAGTGAACGGAGTTGGTTGGGTTGTTTGTGTGTACGGATCCCGCTGT

	TGGTGATGAATGGATGGGTCGTTACGATCACTTACTTGCATCACACGCATATAGC<br>GTTGCCGCGGTATGATTCGTCCGAATGGGATTGGTTGAGGGGTGCATTGGCGACT<br>GTGGATAGGGACTATGGTGTTTTGAACAAGGTGTTTCATAACATCACGGATACTC<br>ATGTGGCGCACCATCTGTTTTCGGCGATGCCTCATTACCATGCGGCGGAAGCGAC<br>CGAGGCGATCAAGCCCGTTTTGGGCGAGTATTATCGGTGTGACCGGACTCCTATT<br>ATCAAGGCGTTATGGAGGGAGTTTAAGCACTGTATTTATGTCGAGTCAGATGAGG<br>ATAAAGGCGTGTTTTGGTTCAATGACAAGTTGTAA
FAD2c-2	ATGCCGCCGCCGCCTTCATCATCCAACACAACCATGAAGCGATCGCCACACTCAA<br>AGCCGCCGTTCACCCTCTCTGACATCAAGAAAGCCATACCACCGCACTGCTTCCA<br>AAGGTCCCTCCTCCGATCATTTGCCTACTTAGCCTACGACCTCACCGCCGTCACAA<br>TCCTCTACCACATCGCCACCACGTACTTCCACCTCCTCCCGACACCTCTCTCATCC<br>TACGTGGCTTGGCCGGCCTACTGGGCCAGCCAAGGCTGCTTCTTCGTCGCTGTATG<br>GATGGTGGCCCACGAGTGCGGTCACCACGCCTTTAGCGACCAACACTGGCTGGA<br>AGACTCGGTAGGGTTCATCCTCCACTCGGCCCTCCTTTCGCCTTACTTCTCGTGGA<br>AGCACAGCCACCGCCGCCACCACGCCAACACGAGTTCCCTCGAGCGTGACGAGG<br>TGTTCGTCCCTAAGCTGAAATCCAAGCTGAGCTGGCACTTCAAGTTCTTCAATAAC<br>CCGCCTGGCCGCGTCCTGCAGCTGGCCTTTGCTCTGCTTCTAGGATGGCCTCTCTA<br>CCTTGCGGTCAACATCGCAGGAAGACCGTACGAGAAATTCGCTAGCCACTTCGAT<br>CCGCGAAGCCCTATCTATAACGATCGGGAGCGAATTGAGATATTTGTATCTGATG<br>TTGGCGTGCTTGCTATGTGGTTCGCTCTGTATAAACTTGCCCTGACGAACGGAGTT<br>GGTTGGGTTGTTTGTGTGTACGGGGTCCCTCTGTTGGTGATGAATGGATGGATCGT<br>TACGATCACTTACTTGCATCACACGCATGTCGCATTGCCGCGGTATGATTCCTCGG<br>AGTGGGATTGGTTGAGGGGTGCGCTCGCGACTGTAGATAGGGATTATGGGGTTTT<br>GAACAAGGTGTTTCACAACATTACAGATACTCATGTGGCGCACCATCTGTTTTCG<br>ACGATGCCTCATTACCATGCGGCGGAAGCAACCGAAGCGATCAAGCCCGTTTTGG<br>GTGAGTATTATCGGTCTGACCGGACTCCTATCGCCAAGGCATTATGGAGGGAGAC<br>TAAGCACTGTGTCTATGTCGAGGTTGATGAAGATAAAGGCGTGTTGTGGTTCAAT<br>GATAAGATATGA
FAD2d-1	ATGGTATCCAATACCACCATAAAGCGAACACCGACCTCAAAACCGCCGTTCACC<br>CTCTCCGACGTCAAGAAAGCAATACCACCGCACTGCTTCCAAAGATCCCTCCTCA<br>AATCCTTCACCTACCTAGCCTACGATCTCACCGTCATCACAATCCTCTACCACATC<br>GCCACGTCATACTTCCACCTCCTCCCCAACCCTCTCTCCTACGTGGCGTGGCCGCT<br>CTACTGGGCCGCCCAGGGGTCACACTTCATCGCTTGGGCCGCTCAGGGGTCCCAC<br>TTCATCGCCGTGTGGGTCATCGCCCACGAGTGCGGCCAACACGCCTTCAGCGACT<br>ACCAGTGGCTAGACGACGCCGTTGGGTTCGTCCTCCACTCCCTCCTCTTAGCTCCT<br>TACTTCTCCTGGAAGCACAGCCACCGCCGTCACCACGCCAACGCCGCCTCCATCG<br>AGCGCGACGAGAATTACATCCCGAAAAAGAAGGACGAAGTCAACTGGCATTTTA<br>AATACCTCGACAACCCTCCCGGACACGTGTTCTATATATTCTTCACTCTCACCCTC<br>GGATGGCCGCTCTACTTGCTGGTCAACATCTCCGGCAGGAAATACGACGACGGAT<br>TCGCCAGCCATTTGTATCCTTTCTCCCCTATCTACAACGACCGCGAGAGATTCGGG<br>ATAGTCCTCTCCGTCGCCGGGATGCTCGCCACGTGGTTCGGTCTTTATAAACTCGC<br>GATGGTCAACGGGTTCGGTTGGGTTGTTTGCGTGTACGGGGTGCCTCTCATTCTCC<br>AGAATGCGATGTTGATTACCATCACTTACTTGCACCACACGCATCTCAACTTGCCC<br>CACTACGACTCGTCGGAGTGGGACTGGATGAGGGGTGCACTCGCGACTGTGGATA<br>GGGATTACGGAATTTTGAATAAGGTGATGCATAATATTACGGATACCCACGTGGC<br>GCACCATCTGTTCTCGATGATTCCTCATTATCATGCGATGGAGGCTACAAATGCGA<br>TAAAGCCGGTTCTTGGGGAGTACTACCAAGTTGATACTACGCCGTTTTTGAAGGC<br>GTTGTGGAGAGAGACTAAGGATTGTGTTTATGTTGAGGCGGATGATGAGGGATCT<br>GATCGGGAGAAGAAAGGAGGGGTGTTTTGGTTCAAAACCAAGCTGTAA
FAD2d-2	ATGGTATCCAATACCACCCTAAAGCGAACACCGACCACAAAACCGCCGTTCACC<br>CTCTCCGACCTCAAGAAAGCAATACCACCACACTGCTTCCAAAGATCCCTCCTCA<br>AATCCTTCTCCTACCTAGCCTACGACCTCACCGTCATCACAATCCTCTACCACATC<br>GCCACGTCATACTTCCACCTCCTTCCCAAACCTCTCTCCTACGTGGCCTGGGTGGC

	CTATTGGGCCGCTCAGGGGTCCCACTTCATCGCCGTGTGGGTCATCGCCCACGAG TGCGGCCACCACGCCTTCAGCGACTACCAGTGGCTAGACGACGCCGTCGGCTTCC TTCTCCACTCCCTCCTCTTAGCTCCTTACTTCTCCTGGAAGCACAGCCACCGCCGC CACCACGCCAACGCCGCCTCCATCGAACGTGACGAGAATTACATCCCGAGAAAG AAGGACGAAGTCAACTGGCATTTTAAGTACCTTGATAACCCTCCCGGACACGTGT TCTATATATTCTTCACTCTCACCCTCGGATGGCCGCTCTACTTGCTGGTCAACATCT CCGGGAGGAAATACGACGACGGATTCGCCAGCCATTTGTATCCTTTCTCCCCGAT CTACAACGATCGGGAGAGGTTCGGGGTGTTCCTCTCCGTCGCGGGGATGCTCGCC ACGTGGTTCGGACTTTATAAGCTCGCAATGGTCAACGGTCTCGGCTGGGTTGTTTG CGTGTACGGGGTCCCTCTGATTCTCCAGAATGCGATGTTGATTACCATCACTTACT TGCACCACACGCATCTCAACCTGCCCCACTACGACTCGTCGGAGTGGGACTGGAT GCGGGGTGCGCTCGCGACTGTGGATAGGGACTACGGGATTTTGAACAAGGTAAT GCATAATATTACGGATACTCACGTGGCACACCATCTGTTTTCGATGATTCCTCATT ACCATGCGATGGAGGCTACGAATGCGATCAGGCCGGTTCTGGGGGAGTACTACC AGGCCGATACGACGCCGTTTATTAAGGCGTTGTGGAGGGAGACTAAGGACTGTGT TTATGTTGAGGCGGATGATGAGGGCGGTGATCAGGAGAAGAAAGGAGGGGTGTT CTGGTTCAAAACCAAACTGTGA
FAD2e-1	ATGTCCCCTATTTACAGCGACCGCGAGCGAGCCGAGGTATTCGCCTCAGATGTTG GCTTGCTCGCTGTCTGCTTCGCGTTGTACAAACTTATTATGGTCAAGGGAATGGCG TGGGTTTTTTGCGTCTATGGGGCTCCGGTCATGGTGGTGAATGGATTCTTCATTACC ATCACTTACTTGCATCACACTCATCTCGCGGTCCCGCGATACGATTCGTCTGAATG GGATTGGTTGAGGGGAGCCCTGGCAACCATGGACAGGGACTTTGGCCTTTTGAAC AAGGTGTTCCATAATGTTACAGATACTCACGTGACGCACCATCTGATTTCGACGA TCCCTCATTATCATGCCATGGAAGCCAACAACGCAATTAGGCCCGTGTTGGGGGA CTACTACCATATCGACAGGACGCCGGTGGTTAAGGCGTTGTGGAGGGAGGCTAA GGAGTGTGTTTACATCGAGGCCGATGATGGTGAAAAGAACAAAGGCGTGTTCTG GTTCAATACCAAGCTCTAA
FAD2e-2 (exon-intron junctions are not given)	ATGCACTTTCCGATAGCTAGCCTTTGTCTCATGCTAAAAACAGGCCATACATGGC GCCACAAAGCACTGCTCTAGTAAGTACACATGATTGGGATTACACTACACTTCAG TAATTAAATCAGTCCATATTTTACCTGGATCCAAAGTTGAAAACTAACTCAAAAA CAAATTTGTGCAAAATTGAGCATCAAATGAGACAATATTAAACTATTTCGAGTTTT GTTGGGCTGTTCTCGAGTTGTACTAGCCTCTCCGAAAACATGTATAAAAAGCACTC GACCTATTCATATTAGACAACATCAAAATCCCACCAATCAATCAAAAAACAGAG AGACACATCTAAAAGCGCCCCCCCCCCCCACCNNNNNNNNNNNNNNNNNN NNNNNNNNNNNNNNNNNNNNNNNNNNNNNNNNNNNNNNNNNNNNNNNN NNNNNNNACCCAGTCCCGCCGGCGCCAAGCGGTTCCCGTCCTCAAAGCCGCCGT TCACACTCGCCGACCTCAAGAAGGCCATTCCGCCGCACTGTTTCAAAAGATCCAT CCCACGTTCCTTCTCCTACTTGGTTTTCGACCTCATCGTCGCCGCCGTCTTCTACCA CATCGCCGCCACTTATTTCCCCCTCATCCCCAAACCTCTCTCCTACGTGGCGTGGC CGGCCTACTGGTTCGTCCAGGGCAGCGTCCTGACCGGTGTGTGGGTCATCGGCCA CGAGTGCGGCCACCACGCATTTAGCGAGCACCAGTGGCTCGACGACCTGGTCGG GTTCATTCTCCACTCGGCCCTCCTCACCCCTTACTTCTCGTGGAAGATCAGCCACC GCCGCCACCACGCCAACACCTGCTCCCTCGAGCGCGACGAGGTATATACTGTTGG GAACCGGACAAGAAGCAATATTCAGGGTTTATCGATCTGGCTGAGTATACATGGA CAAACTATCCATCAACATCCAAGATAAAAACAGAGCTATAACTTTCAACTTCCAT GTGCAAAAATAGGGAGATGAATAAAATTTCAGATAATTTAAACAAATAAATTAT CTCCAAAATGAATAATATTGGTCTCAAGTCCAATCCAACCTGGAGAACTGGATCT TTCTACAAAGACGTTTTTGCATTAGTCTTTTTTTCTTTCTCCGATGTGATATTTGAGA CTTAATTATTCACATTTTCAACAGGTGTACATTCCGAGGAAGAAGTCTCAGTTGC GGTGGTGGTACTCGAGCTACCTGAACAACCCGCCCGGCAGACTTTTGGCGCTGGC TTACACCATCCTCCTCGGCTGGCCTTCCTACTTGACTTTCAACCTATCCGGTAGGG AATACAACGGTTTCGCCTGTCATTTCTACCCGATGTCCCCTATTTACAGCGACCGG GAGCGGGCCGAGGTATTCGCCTCCGATGTTGGCTTGCTTGTTGTGTGCTTCGCGCT

	GTACAAACTTATCCTCGTCAAGGGAATGGCGTGGGTTTTTTGCGTCTACGGGGCTC CGGTCATGGTGGTGAACGGATTCTTCATAACCATCACTTACTTGCACCACACTCAT CTTGCGGTCCCGCGGTACGATTCCTCGGAATGGGATTGGTTGAGGGGAGCCCTAG CAACGATGGATAGAGACTTTGGGTTATTGAACAGGGTGTTCCATAACGTTACAGA TACTCATGTGACGCACCATCTGATCTCGACCATCCCTCACTACCATGCCATGGAA GCCAACAACGCGATTAGGCCCGTGTTGGGGGACTACTACCACATCGACAGGACG CCTGTCGTTAAGGCGTTGTGGAGGGAGGCTAAGCAGTGTGTTTACATCGAGGCGG ACGAAGGTGAAAATAACAAAGGCGTGTTCTGGTTCAATACCAAGCTCTAA
FAD2f-1	ATGGTGTCCTCCGGCAAAACCATGAGCAACAAAACCACCACGAAGCGGCCGCCG GTGTCAAAACCGCCGTTCACTCTGGCCGACATCAAGAGAGCCGTACCGCCACACT GCTTCAAAAGATCCCTCGTCAAATCATTCGCCTACCTAGCCTACGACCTCACCGTC ATCACAATCCTCTACCACATCGCCAACACATACTTCCACCTCCTCCCCAAACCTCT CTCCTACGTGGCGTGGCCCGTCTATTGGGCTGCCCAATGCTGCTTTTTCGTCGCTTT ATGGATGGTGGGCCACGACTGCGGCCATCATTCATTCAGCGACTATCAGTGGGTG GACGACACCGTTGGATTCGTCGTCCACTCGTTCCTCCTCGCCCCTTACTTCTCGTGG AAGCATAGCCACCGCCGCCACCACGCCAACTCCGGATCTCTAGAACGCGACGAG TCGTTCGTCCCGAAAACCAAAGACAATATCACCTGGCACTTCAAGTACCTCGACC ACCTACCGGGGCGGATCTTCTACGTGGTCTTCACTCTAACCCTCGGATGGCCGCTC TACTTGATGTTCAACATCACCGGAAGGCCGTACAAGGACGGATTCGCCAGTCATT TCTACCCCATGTCGCCTATCTACGAGGATCACGAGCGGTTCGGAATATTCCTCTCT GATGTTGGCATGCTCGCTATGTGGTTCACACTCTACAAGCTTTCCGTGGCGTATGG AGTCGGTTGGGTTCTTTGTGTGTATTTTATTCCGCTGGTTTTACAAAATGCGTTGTTT GTCACGATCACTTACTTACACCACACGCATCTGAACTTGCCTCACTATGACTCTTC GGGGTGGGACTGGATGAGGGGGTCGTTGACGACTGTGGATAGGGACTATGGGTTT TTGAACAAGGTGTTGCATAACGTGACAGATACTCACGTGGCGCACCATCTGTTCA CCCATATGCCTCATTACCACCAATCGGAAGCGACCAAGGCGTTCATTCCGGTTTTG GGGGAATACTACCAGGTTGATCCGACTCCGTTCTACAAGGCGTTGTGGAGGGAGA TGAAGCACTGTGTTTATATCGAGCAAGATGAGGATGCGGATTCTGATAATAACAA AAAAGGCGTGTACTGGTACAAAACCAAGCTCTAA
FAD2f-2	ATGGTATCCTCCGACAGAACCACGAACAACAAAACCACCACGAAGCGGCCGCCG CTGTCAAAACCGCCGTTCACCCTCGCCGACATCAAGAGAGCCGTACCGCCGCACT GCTTCAAAAGATCCCTCGTCAAATCATTCGCCTACCTAGCCTACGACCTCACCGTC ATCACCTTCCTCTACCACATCGCCAACACATACTTCCACCTCCTCCTCAAGCCTCT CTCCTACGTGGCGTGGCCCCTCTACTGGGCCGCTCAATGTTGCTTCTTCGTCGCTTT ATGGATGGTGGGTCACGATTGTGGCCACCATTCGTTCAGCGACTATCAGTGGATT GACGACACTGTCGGTTTTATCATCCACTCGTTCCTCCTTGCTCCTTATTTCTCCTGG AAGCACAGCCACCGCCGCCATCATGCCAACTCCGGATCTCTAGAACGCGACGAG TCGTTCGTCCCGAAAACCAAAGACAATATCGTCTGGCACTTCAAGTACCTCGACC ACCTACCGGGGCGGATCTTCTACGTGGTTTTCACTCTGACCCTCGGGTGGCCACTA TACTTGATGTTCAACATCACTGGAAGGCCGTACAAGGATGGATTCGCCAGCCATT TCTACCCCATGTCGCCGATCTATGAGGATCACGAGCGGTTCGGGATATTCCTCTCC GATGTTGGAATGCTCGCCATGTGGTTCACGCTCTACAAGCTCTCAGTGGCATATGG AGTCGGTTGGGTTCTTTGTGTGTATTTTATTCCGTTGGTTTTACAAAATGCTTTGTTT GTTACGATCACTTACTTGCACCACACGCATTTGAACTTACCTCACTATGACTCTTC GGGGTGGGACTGGATGAGGGGGTCGCTGACGACTGTGGATAGAGACTATGGGTTT TTGAACAAGGTGTTGCATAACGTGACAGATACTCACGTGGCGCACCATTTGTTCA CACATATGCCTCATTACCATCAATCGGAAGCGACCAAGGCGTTCATTCCGGTTCT GGGGGAGTACTACCAGGCTGATCCGACACCGTTCTACAAGGCGTTGTGGAGGGA GATGAAGCATTGTGTTTATGTCGAGCAAGATGAGGATGCGGATTCCGATCAGAAC AAAAGAGGAGTGTACTGGTACAAAACCAAGTTGTGA
FAD2g-1	ATGAACAACAGAACCACCACGAAGCTGCTGCCGCTGTCAAAACCGCCGTTCACT CTGGCCGACATCAAGAGAGCCGTACCGCCACACTGTTTCAAAAGATCCCTCGTCA AATCATTCGCCTACCTAGCCTACGACATCACCGTCATCACAATCCTCTACCACATC

	GCCAACACATACTTCTACCTCCTCCCCAAACCTCTCTCCTACGTGGCGTGGCCCGT<br>CTATTGGGCCGCCCAAAGCTGCTTCTTCGTCGCTGTATGGATGGTGGGTCACGATT<br>GCGGCCACCATTCATTCAGCGACTACCAGTGGGTGGACGACACTGTCGGATTCGT<br>CGTCCATTCGTTCCTCCTCACCCCTTACTTCTCGTGGAAGCACACCCACCGCAGCC<br>ACCACGCCAACAACGGATCTCTAGAACGCGACGAATCTTTCGTCCCCAAGACCA<br>AAGACGAAGTCAGGTGGCACTTCAAGTACCTCGACCACCTACCGGGGCGAATCTT<br>CTACGTGTTCTTTACTCTAACCCTAGGATGGCCTCTCTACTTGATGTTCAACATCAC<br>CGGAAGGCCGTACAAGGATGGATTCGCTAGCCATTTCTACCCCATGTCGCCTATG<br>TACGAGGACCATGAGCGGTTTGGAGTAGTCCTGTCCGACATGGGAATGCTCGCTA<br>TGTGGTTTACGCTCTACAAGCTCTCGGTGGCGTTCGGAGTCACTTGGGTTCTCTGT<br>GTGTACTTTATTCCACTAGTTTTACAAAACGCGTTGTTTGTTACGATCACTTACTTG<br>CACCACACGCATCCAAACGTGCCTCGTTATGACTCTTCGGGGTGGGGTTGGATGA<br>GGGGGTCGTTGGTGACCGTGGATAGAGACTATGGGTTTTTGAATAAAGTGTTCCA<br>TAACGTGACGGATACTCACGTGGCGCACCATCTGTTCACGCATATGCCTCATTACC<br>ACCAATTGGAAGCTACCAAGGCGTTCATCCCGATTTTGGGAGAGTACTACCAGGC<br>TGATCCAACACCGTTCTATAAAGCATTGTGGAGAGAAATGAAGCACTGTGTTTAT<br>GTCGAACAAGACAAAGATGCAAATGTCGATCAGAACAAAAGAGGTGTGTATTGG<br>TACAAAACAAAGTCGTGA
FAD2g-2	ATGGTATCCTCCGACAAAACCATGAACAAAAAAACCACCACGAAGCTGCTGCCG<br>CTGTCAAAACCGCCGTTCACTCTCGCCGACATCAAGAGAGCCGTACCGCCACACT<br>GCTTCAAAAGATCCCTCGTCAAATCATTCGCCTACCTAGCCTACGACATCACAGT<br>CATCACAATCCTCTACCACATCGCCAACACATACTTCCACCTCCTCCCCAAACCTC<br>TCTCCTACGTGGTGTGGCCCCTCTACTGGGCTGCCCAATGTTGCTTCTTCGTCGCGG<br>TATGGATGGTGGCCCATGATTGTGGCCACAATTCATTCAGCGACTATCAATGGGT<br>CGACGACACCGTCGGTTTCATCATCCACTCGTTCCTCCTCGCCCCTTACTTCTCATG<br>GAAGCACACCCACCGCAGCCACCACGCCAACAATGGATCTCTAGAACGCGACGA<br>GTCTTTCGTCCCCAAGACCAAAGACGAAATTAGATGGCACTTCAAGTATCTCGAC<br>CACCTACCGGGGAGAATCTTTTACGTGTTCTTCACTCTAACCCTAGGATGGCCTCT<br>CTATTTGATGTTCAACATCACCGGAAGGCCGTACAAGGACGGATTCGCCAGCCAT<br>TTCTACCCCATGTCGCCTATGTACGAGGACCACGAGCGGTTCGGAGTAATCCTCTC<br>TGACATGGGAATGCTCGCCATGTGGTTCACCCTCTACAAGCTCTCAATGGCGTATG<br>GAGTCAGTTGGGTTCTCTGTGTGTACTTTATTCCACTGGTTTTACAAAATGCGTTGT<br>TTGTCACGATCACTTACTTGCACCACACGCATCCGAACTTGCCTCGTTATGACTCT<br>TCGGGGTGGGGTTGGATGAGGGGGTCGTTGGTAACAGTGGATAGAGACTACGGG<br>TTTTTGAACAAAGTGTTGCATAACGTGACAGATACTCACGTGGCGCACCATCTATT<br>CACGCATATGCCTCATTACCACCAATTGGAAGCTACCAAGGCGTTCATTCCGGTTT<br>TGGGGGAGTACTACCAAGCTGATGCAACACCGTTCTACAAGGCATTGTGGAGGG<br>AGATGAAACACTGTGTTTATGTCGAGCAAGATGAGGATGCAGATTCCGACCAAA<br>ACAAAAGAGGTGTGTACTGGTACAAAACCAAGCTGTGA
FAD2h	ATGGTATCCAACAAAACCATAAACCGGCCGCCGTCCTCAAAACCGCCGTTCATCC<br>TCTCCGACATCAAGAAAGCAATCCCACCACACTGCTTCCGCAAATCCCTCCTCCG<br>ATCATTCTCCTACGTAGCCTACGACCTCGCCGTCATCGCAATCCTCTACCACATCG<br>CCACGTCATACTTCCACCTCCTCCCCAAACCTCTCTCCTACGTGGCCTGGCCGGCA<br>TACTGGGCCGCCCAGGGATCCCACTTCATCGCCGTCTGGGTCCTGGCCCACGAGT<br>GCGGCCACCACGCCTTCAGCGACTACCAGTGGCTAGACGACGTCGTCGGATTCGT<br>CCTCCACTCCGCTCTCCTCTCGCCGTACTTCTCGTGGAAACACAGCCACCGCCGCC<br>ACCACTCCAACTCGGCATCTCTAGAACGCGACGAGCTGTACATCCCGAAGAAGA<br>AATCGGAGATTAGCTGGCATTACAAGTACCTCGACAACCCGCCGGGTCATCTTTT<br>CTACTTGGTGTTCACTCTCACTCTCGGATGGCCTCTCTACGTGATGTTCAACGTCTC<br>CGGCAGGGAATACGACGACGGATTCGCTAGCCATCTGTATCCTTTCTCCCCGATCT<br>ACAACGAACGGGAGAGATTCGGGATACTTTTGTCGGACGCTGGGATGCTTGCCAC<br>TTGGTTTGGTCTGTACAAGCTGTCCATGGTCAACGGACTGAGTTGGGTTGTTTGTG<br>TGTACGGGGTCCCGCTATTGGTGATGAATGGATTGCTTGTGACGATCACTTACTTG

	CATCACACCCATCTTTCGCTCCCGCATTATGACTCGTCGGAGTGGGAGTGGATGA<br>GAGGTGCGCTAGCGACTGTGGATAGAGACTACGGGTTCCCGCTGAATAAGGTGAT<br>GCATCATATTACGGATACTCACGTGGTGCACCATTTGTTCTCGATGATTCCTCATT<br>ACCATGCGACGGAGGCTACAAATGCGATTAGGCCGATTTTGGGTGAATATTATCA<br>AGTTGATCCGACTCCGTTTGTCAAGGCGTTGTGGAGGGAGATGACGCACTGTGTTT<br>ACGTTGAGGCCGATGAGAAGAAACGAGGTGTGTTCTGGTATAAAACCAAGCTAT<br>GA
FAD3a	ATGAGCCCTCCAAACTCAATGAGTCCCGCCACCAACGGCAGCACCAATGGTGTG<br>GCTATCAATGGGGCGAAGAAGCTACTCGATTTCGACCCGAGTGCTGCTCCCCCTT<br>TCAAGATTGCAGACATCCGTGCTGCAATCCCGCCGCATTGTTGGGTGAAGAACCC<br>CTGGAGGTCACTCAGCTACGTCCTGAGAGACCTCCTGGTCATCCTCAGCTTCGCCG<br>TTGCGGCGACAAAGCTGGACAGCTGGACTGTCTGGCCTCTCTACTGGATTGCTCA<br>AGGAACCATGTTCTGGGCAGTCTTTGTTCTTGGACATGATTGGTAATTTCACATGA<br>TCTTTCTGGTAATGTGGGTTTTCTTTTCTTATTGAAAAAGATTAAAACTTTTTATCTG<br>GGCTGTTGCATGCAGTGGCCATGGGAGCTTCTCAGACAGTTGGTTGTTGAACAAC<br>GTGATGGGACATATACTCCATTCCTCAATCCTCGTACCTTACCATGGATGGTATTG<br>TAACTATTGTTCGATATTCGATTATGATTACTGTTCTTTCAGATGAAGAATCTGTAC<br>CCTAATTGTTTTTTGTTACCAGGAGAATTAGCCACAAGACCCATCACCAGAATCA<br>CGGCAATGTGGAGAAAGATGAATCCTGGGTTCCAGTAAGTTGACATGCAGTTTGC<br>TCTAAAATGCAGAGTCCTCTGTTTTTTGTGTGTTCTTGTGCTTTAATGACGATGATA<br>ATGAAATTGAAATTTGTAATAGCTGCCGGAGAAGGTGTACAAGAGCTTGGATACC<br>GGCACCAAGTTCATGAGGTTCACCATCCCTCTCCCAATGTTTGCGTATCCTATCTA<br>CTTGGTAAGTAAACAGACTGACTCCAAAGTAGGAACTAATGACAATTTTGGACCC<br>GACCTGGTTTGGTTGACTCGGGTCGATATGTTTCGGGTGGGTAATTACCCGATCTG<br>GCGATGGGTGTGCGGCGGACATTGTCTTGCTCGTGGTCCACCCCGCTCCCAACCC<br>GCCCCATTCTTGACGAAAAAGATTTCGGAATATGTATCAACAGAAAAATCTAGTT<br>TTTATGTTACTAGTTTTCTGTATTTCCATGTTTTTTCCTCAATTCTAGCCGGAATTTG<br>AATTCAAACTGAAATCGGGTAATTCCGTCCATAACAAAACGGAATTGGGCAGCC<br>GTAATTAGTTGAAACTAGACCTCAATTTTGGCGGAATTGGACCCGGCCATTTTTTA<br>CGTTTGCAAAACGGAAAACGTTTTTCTTTTGTAAAGCGCAAAATGAAAAACGTAT<br>CTAGTGGAATTATTGGACCCATCTAGAATGGGTCCAATTCCACCCCAATTTCGGCT<br>CCAATTCATGCCCGGAAAACACTACTGTCATGCATTTTAATCTTGTATGGTTTTAC<br>CCCAATGGATGCAGCGATGGATCCGGACGATTTTTAAAATATTATCGGGTTAAAT<br>TTAAAAATATCTTAAAACTATAAGAAAAAAATAACCAATTTTAAAGAATAAAAG<br>AACTGGACACATATGACGGGTGTCGTGGATGGATGTACTTGTCCCGCTCTATTAA<br>AGGCTGATAATATACAGGTCAACGGTGAATGAAGGTTAGATGCGCTATTGGATTT<br>GAATCCGATATGAAATGATAATTTTGGACACGATCTGTTTTGGGTGGGTAATATTT<br>GATCTAGGGATGGCTCGTGCTCCAAACCGCACCAAAACCGCCTAATTCTCGACCA<br>AAAAGATTTTATGAATACATATCAACAGAAAAATCTAGTTTTCATGTTACTAGTTT<br>TATGTACAACAATATTAGGTGTCGTTTTCCCAGCCTTTTTCTTCAATTCCGGCCGGA<br>ATTCGCATTCAAACCGGAATTGGATGGAATCGGTATACCTCGTCACGGATGCATT<br>GTCAATTCCTAGTTAGTTTCATGGTTTTGAAACCAATCAATCTATTCTATATGGTTT<br>TGATTAACAGTGGAGGAGAAGTCCGGGGAAGAAAGGGTCGCATTTCAACCCATA<br>CAGTGACCTGTTCGCACCGAACGAGAGGACATCGGTCATGATTTCGACATTGTGC<br>TGGACAGCCATGGCCTTACTCCTCTGCTACTCATCGTTCATCTACGGCTTCCTTCCG<br>GTCTTCAAAATCTACGGCGTCCCTTATCTAATATTCGTGGCGTGGCTCGACATGGT<br>GACCTACCTTCACCACCACGGGTACGAGCAGAAGCTGCCGTGGTACAGAGGCAA<br>AGAGTGGAGCTACCTACGTGGAGGGCTGACGACCGTCGATCGAGATTACGGGGT<br>CATCAACAACATCCACCATGACATTGGCACCCATGTTATTCACCATCTCTTCCCTC<br>AAATGCCACACTATCACCTAGTCGAAGCGGTAAGGAGGTCTTGATTATTAACTTA<br>ATGTTTTTGTTGTTATAATTTGAGTCCGATTCTGGAGTCAGGGGATTTCCTTCTTGG<br>ATCCGATCCAGGATCAAGCTGGTCCCTTGAATTTCTATATGATCTTATATTAATTA<br>AGGATAATGTGGTCATATGTTTTTAAATATTTTTGTTTACCATCATTTTCGATCACC

	GGAAAATGTCCTGAGCAGTTTTCCGGTCACTTTAACCTCCATTGACAAATTTTTC<br>ACCCACATGATCACCCTAGCCGGGTTTACGTTTATTGAAAATTTTATTTTTTTGAA<br>TTTTTTTTCGATGACCAACTGTACAACTTTGTATTGAAAGTTGTATGGATCATACA<br>AATGTGTATGTACAAAAGTATATTCTAAGTACTATACTAAGCATTACTTAGTATTA<br>CGTTTCTACAAACCTATAGAGAAATGCATACAATTTTGTATAGAACTTAGTATAC<br>ACGTAGCTGTGAAATGTCAATTTCCCTCCGTATTTTCAGAGACAAGACATGATTTT<br>TAGACTGGCAGATTTTTTTATCGGATAGATTTCTCCAACTTCAGATTCGGACTGG<br>ATTATTAACTATATTATTCATCAACTCTGACGTTTGATGTTGCATGTGACAGACTC<br>AGGCAGCGAAGCACGTGCTGGGGAAGTACTACAGAGAACCGAAGAAATCAGGG<br>CCTTTCCCATTCCACTTGTTTGGGTACTTGGTGAGGAGCCTGGGCGAGGATCACTA<br>CGTTAGCGATACAGGCGACGTCGTTTTCTATCAATCTGACCCACATATTCCCAAGT<br>TCCCTACCAGTGCCACCACCAAGTCCAAATCTAGCTGA
FAD3b	ATGAGCCCTCCAAACTCAATGAGTCCCACCACCAACGGCAATGGTGTGGCTATGA<br>ATGGGGCGAAGAAGCAGCTCGATTTCGACCCGAGTGCTGCCCCCCCTTTCAAGAT<br>TGCAGACATCCGTGCTGCAATTCCGCCGCATTGCTGGGTGAAGAACCCCTGGAGG<br>TCGCTCAGCTACGTCCTGAGAGACCTCCTTGTCATCCTCAGCTTCGCCGTTGCGGC<br>GGCAAAGCTGGACAGCTGGACTTTCTGGCCTCTTTACTGGGTTGCTCAAGGAACC<br>ATGTTCTGGGCAGTCTTTGTTCTTGGACATGATTGGTAAACTAATTTCACATTTTCT<br>TTCTGGTAATGTGGGTTTTATTGAAAAAGATTAAAACTTTTTATCTGGGTTGTTGCA<br>TGCAGTGGCCATGGGAGCTTCTCAGACATCTGGTTGTTGAACAATGTGATGGGAC<br>ATATACTCCATTCCTCAATCCTCGTACCTTACCATGGATGGTATTGTAACTATTGTT<br>CAATATTAGATTATTGCTAGTTCTTCAGCTGAAGAATCCAAACCCTAATTTTCTTTT<br>TCTGAATATTGACCAGGAGAATTAGCCACAAGACCCATCACCAGAATCACGGCA<br>ATGTGGAGAAAGATGAATCCTGGGTTCCTGTAAGTTGACATGCAGTTTGCTGTAA<br>AAATGCAGAGTGCTCTGTTTTTTTGTGTTCTTGTGCTTTAATGGTGATAATAATGAA<br>ATTGTTGAAATGTAACAGCTACCGGAGAAAGTGTACAAGAGCTTGGATACCAGC<br>ACTAAGTTCATGAGGTTCACCATTCCTCTCCCAATGTTTGCTTATCCTATCTACTTG<br>GTAAGTAAAGAGACTGATAAGACTCCAAAGTAGGAATTAATGACAATTTTGGAC<br>CCGAGCTTCCGACTCGGGTCGATTTATTTCGGGTGGGTACTTACCCGATGCGGCGG<br>ACATTGTTTTGCTCGTGGTCCACCTCGCTCCCAACCCGCCCCATTCTTGACGAAAA<br>AGATTACGGAATATGTATCAACAGAAATATCTAGTTTTTATGTTACTAGTTTTCTGT<br>ATTTCCGTGTTTTTCCCCTCAATTCCGTCAGAAATTTGAATTCAAACTGACATTGGG<br>TAATTCTGTCCATAAGAGAACGGAATTGGGAAGCCGTAATTAGTTGGAATTAGAC<br>CTCGATTTCGGCGGAATTGGACCCGGCCATTTTTCGCGTCCGGAAAAGCGTTTCCA<br>GTGGAGTTAGACGCCCATCTAGAATGGGTCCAATTCCACCCCAATTTCGGGGCTA<br>CCTATTTTTAGTGTACATGCATTTTAGATCTTTTACGGTCTTACCCCGATGGATATT<br>CTTGTCGCGCTCTATTAAAAACTGACAATATACAGGTTGATTGTGAATGAAGATT<br>AGGTGCTGCACGCTCTTCGTGTTCCAACCCGCACCCAAACCGCCCCATTCTCGAC<br>CGAAAAGATTTTATGAATACATATCTACAGAAAAATCTAGTTGTCATGTCACTAG<br>TTTAATGTACAACAGTATAGGTGTCGTTTTCCGCGTCTTTTTCTTCAATTCCGGCTG<br>GAATTCGCATTCAAACAGGAATTGGATGGAATTGGTCCCGGATGCATAGTCATTT<br>CCCAGGCAGTTTCATGGTTTTATAACCAATCAATCTAATCTTATGCTTTTGATAAA<br>CAGTGGACGAGAAGTCCGGGGAAGAAAGGGTCGCATTTCAACCCATACAGCGAC<br>CTATTCGCACCAAACGAGAGGGCAGCGGTCTTGATTTCAACATTGTGCTGGACAG<br>CCATGGCCTTACTCCTCTGCTACTCATCGTTCATATACGGCTTCGCTCCGGTCCTCA<br>AAATCTACGGCGTACCTTATCTGATATTCGTGGCATGGCTCGACATGGTGACCTAC<br>CTTCATCACCACGGGTACGAGCAGAAGCTGCCGTGGTACAGAGGCAAAGAATGG<br>AGCTACCTACGTGGAGGGCTGACGACCGTTGATCGAGATTACGGGGTCATCAACA<br>ACATCCACCATGACATTGGCACCCATGTCATTCACCATCTCTTCCCTCAAATGCCA<br>CACTATCACCTTGTGGAAGCGGTAAACAATTTGATTATTAATTTACTGTTTTTGTTG<br>TTATAATTTGAGTCGGGAGATTTCCTTCCTAAATCCGATCCCTGGTCAATCTTGGC<br>CCTTGAATCTTCATATAATCTAAAAATCTAGATTAATCAGGAACAATATGATCAT<br>GTTGTTTAAACTAATTTTGTTGGACCATAACCTACCGCCAACTGATGGACCACCGT

	CTCTGGTTACCGGACCCATCATTTCCGGTTACCAAGAAGTTTCTCGATCAGTTTTC<br>CGGTTACTTTGACCTGCGTTGAGGAAAATTCTTTCACCCACGTAAACACTGTCGTC<br>AACTTTACGTTTCTGGAAAGTTTTTCCGATGATTGGCCGTACAATTTTGTACGAAG<br>AGTTGTACGGATCATATAAATGTGTATAAGTTTCTAGAAATCCGTACTGAAATAT<br>ATACATATTTGACTTTTGTATAAAGTGTAATACTAAATACTATACTAAGTGCTGTA<br>CTCAGTATGATACTTAGTACACACATTTGTATGACTATGAAATGTCAATTTTGCCC<br>TTATATTCTCAGCCGTTAGATCTAAGACACAGTTTTTATACGGCTGAAATTTGTGG<br>GGGCTTTGTAGATCGGATCCATAAGTCATTTCTTGGCTCAAGATTCGGACTCGATT<br>ATTAACTATATTATTCATCAACTCTGACGTTTGATGTTGCATGTGACAGACTCAGG<br>CAGCGAAGCACGTGCTGGGGAAGTACTACAGAGAGCCGAAGAAATCAGGGCCTT<br>TCCCATTCCACTTGTTTGGGTACTTGGTAAGGAGCCTGGGCGAGGATCACTACGTT<br>AGCGACACAGGCGACGTCGTTTCTATCAGTCTGACCCACATATTCCCAAGTTCCG<br>TACCAGCAGTGCCACCACCAAGTCCAAATCCAGCTGA
FAD3c-1 (wrong sequence, but CDS is not affected)	ATGAGCCCTCCAAACTCCATGGGAATGGAAGCTGCTCATCCTACCGGCAACGGC<br>AATGGCGTCGCAGTCATGAACGGCGCCTCCGCTAACAAACCCGATTTCGATCCCA<br>GCGAGGCTCCTCCCTTCAAGATTGCCGACATCCGAGCCGCCATCCCCCCGCATTG<br>CTGGGTGAAGAATCCATGGAGGTCTCTTAGCTACGTCCTTAGGGACGCCGTCGTC<br>ATTCTCGCATTCGCAGCTGCCGCCCTCAAGCTCGACCTCTGGGCTGTTTGGCCGCT<br>CTACTGGATCGCTCAGGGCACCATGTTTTGGGCTGTCTTCGTTCTCGGCCACGATT<br>GGTAAGAGGAGGAGGTTTTTTTTTAAAATTTCAATCCGATGCTGTTTTGTTATATTA<br>TTGGTTGATATGATATATAAGATTAACCCTAGAGAAAAGTGAAAAGCATCCGCCA<br>TTGTTGAATGAATTGGAATTGGATGGTGCAGCGGCCATGGGAGTTTCTCGGATAG<br>CTGGTGGCTGAACAATGTGGTGGGGCATATTCTGCATTCTGCAATCCTTGTGCCTT<br>ACCATGGATGGTAAGAAAAGAATATGAACAGATCCTTTATCCTTACAACTGTAAA<br>ATGTAGGTCATTCAGCTGAACTAAAGTCACATTCTGTGTTGTTTAATTGATTGAAC<br>AGGAGAATTAGCCACAAAACACACCACCAGAATCATGGCAATGTTGAGAAAGAT<br>GAATCATGGGTTCCGGTGAGTTTTATTTCATTTTTTCTCTCGTCAATTGGCATCAGT<br>TTGTTCTGTTTCAATGCTTGATATGAATTGGGTGATATAATGCAGCTGCCGGAGAA<br>AGTATACAAGACCCTGGACACAAGCACCAAATTCATGAGGTTCACTATCCCTCTC<br>CCAATGTTTGCTTATCCTATCTACTTGGTAAGAACCACCCCACATTCTTACATCCTT<br>GCATGTTGTGTATTGGTTTCTGGTTGGATTTGGATTCAGATTTGGGGAATTGATTGT<br>GTGCCTTTGAAATGAATGAATGGTTACAGTGGACGAGGAGCCCAGGGAAGAAAG<br>GGTCCCATTTCAACCCCTACAGTGACCTGTTTGCCCCACAAGAAAGGAAATCAGT<br>CTTAATCTCTACCATCTCTTGGATTTCCATGGTCCTAATCCTCCTCTACGCCTCCTT<br>CCTTTTTGGTTTCCTCACTGTCTTCAAAGTCTATACCGTCCCTTACCTGGTAAAACT<br>ACTATCTCCAAATTCAACAATCTGTAAGCTTCATTTCATTTGCTTTGGCTCCTGAGT<br>TCCCTGTCAAGTTTCTCCTAGCAGTGTAACTTGTTATTTGATAACTTTGTGCCATCT<br>CACTATTGGCTGCATTTTACTATAATCAAACTGCTACATACGTTATTGCAACTTGTT<br>AATCCTTGGTACGAGGAATCCAATGAATAGGATGGTGATTAGCACAAACAAGGC<br>TGAAACTTTGAGCAATTTTGCAGATATTTGTGGCGTGGCTGGACATGGTGACATAC<br>CTGCACCACCACGGGCACGAAGAGAAGCTGCCGTGGTACAGAGGTCAAGAGTGG<br>AGCTACCTACGTGGAGGGCTGACAACCGTAGATAGAGATTACGGATTATCAAC<br>AACATCCACCACGACATTGGCACTCACGTGATTCACCATCTGTTCCCTCAAATCCC<br>TCACTACCATCTCGTAGAAGCGGTACAGTAGTAGTAAACAAAACCCCATATTATT<br>ATTACTTACTGCAGTGATTTCTGACTCTATATTATTAAATATCACTTCTGGGTTTTT<br>GGCCGCGGGGGGGGGGGGGGGGGGTGTTTGGGTTTTTTTGTCTTTTTTTTAATTTT<br>TTTTTTTTTAATATTGTGGGTGATAGAATTATTATTAAAATGGTGGCAGACAAAG<br>GCAGCAAAGTCGGTGCTTGGGAAGTACTACAGAGAGCCAAAGAAATCAGGACC<br>ATTCCCATTCCACTTGTTCGACAACTTAGTGAGAAGCCTTGGCGAAGATCACTATG<br>TTAGTGATGCAGGGGATGTCGTGTTCTATCAGTCTGACCCAGAAATCTTCAAGTTT<br>TCCAAGTCCAAGTCAGCCTAA
FAD3c-2	ATGGGAATGGAAGCTGCTCATCCTACCGGCAACGGCAATGGCGTCGCCGTCATGA<br>ATGGCTCCGCCGCTAACAAACCCGATTTCGATCCCAGCGAGGCTCCTCCGTTCAA

	GATTGCCGACATCCGAGCCGCCATCCCTCCGCATTGCTGGGTGAAGAATCCATGG<br>AGGTCACTCAGTTACGTCCTTAGGGATGCCGTTGTCATTCTCGCATTCGCCGCTGC<br>CGCACTCAAGCTCGACCTCTGGGCTGTTTGGCCGCTCTACTGGATCGCTCAGGGC<br>ACCATGTTTTGGGCTGTCTTCGTTCTCGGCCACGATTGGTAAGAGGAGGAGTTGTT<br>TTTTTTAAATATTTATCCGATGCTGTTTTGTTATATTATTGGTTGATATGTAAGATTA<br>ACCCTAGAAAAAAGTGGAAAACGTCCGCCATTGTTGAATTGAATTGGAATTGGAT<br>GGTGCAGCGGCCATGGGAGTTTCTCGGATAGCTGGTTGCTGAACAATGTGGTGGG<br>GCATATCCTGCATTCTGCAATCCTTGTACCTTACCATGGATGGTAAGAAAAGAAT<br>ATGAACAGATCCTTTATCCTTGAAACGGTAAAATGTAGGTTATTCAGCTGAAACA<br>AAAGTGACATCCTGTGTTGTTCTTTATTGATTGAACAGGAGAATTAGTCACAAAA<br>CTCATCACCAGAATCATGGCAATGTTGAGAAAGATGAATCATGGGTTCCGGTGGG<br>TTTTATTTCTCTTCCTTACTTGTCAATTTGCCATCATTTGTCTGTTTCACTGATTGAC<br>ATGAATTGGGTGGGAAAATGCAGCTGCCAGAGAAAGTGTACAAGACTCTGGACA<br>CAAGCACAAAATTCATGAGGTTCACTATCCCTCTCCCAATGTTTGCTTATCCTATC<br>TACTTGGTAAGAACCACCCTACATTCTCACATCCTTGCATGTTGTGTATTGGTTTCT<br>GGTTGGATTTGGATTCAGATTTGGGAAATTGATTGTGTGCATATGAAATGAATGA<br>ATGGTTACAGTGGACAAGAAGCCCAGGGAAGAAAGGGTCCCATTTCAATCCCTA<br>CAGTGACCTGTTTGCCCCACAGGAAAGGAAATCAGTCTTAATCTCTACCATCTCTT<br>GGATTTCCATGGTCCTAATCCTCCTCTACGCCTCCTTCCTATTTGGCTTCCTCACAG<br>TCTTCAAAGTCTATACCGTCCCTTACCTGGTAAAAACTCCACCCTTGGTTTACTAG<br>CTTCAAATTCAACAATCTATCAGCTTCATTTCATTTGCTTTGGCTCCTAAGTTCCCT<br>CTTAAGTTTCTCGTAGCAGAGTAACTTGTCTATTTGTTAACTTTGTGCCATCTCCCT<br>ATTGGGTGCATTTCACTATAATCAAACTGCTACATGCATACTGGAATGTCCATATT<br>GTAATTTGCTATAATCCTTGGTACGAGAATCCAATGAATAGGATGGTGATTAGAA<br>CAAGAAAGACTGAAACTTTGAGCAAATTTTGCAGATATTTGTGGCGTGGCTGGAC<br>ATGGTGACATACCTGCACCACCACGGGCACGAAGAGAAGCTGCCGTGGTACAGA<br>GGCAAAGAGTGGAGCTACCTACGTGGAGGGCTGACAACCGTAGATAGAGATTAC<br>GGGATTATCAACAACATCCACCACGACATTGGCACTCACGTGATTCACCACCTCT<br>TCCCTCAAATCCCTCACTACCATCTCGTAGAAGCGGTACTGTAGTAAACAAAACC<br>CATATTATTATTACTTACTGCAGTGATTTCTGACTATGTTATTATTAAATATCAGTT<br>CTGGGTTTTGGCCTTTGGGGGGGTTGGTTTGGATCTTTGGCACTTATAATATTATTA<br>AAATGGTGGCAGACAAAGGCAGCAAAGTCGGTGCTTGGGAAGTACTACAGAGA<br>GCCAAAGAAATCAGGGCCATTCCCATTCCACTTGTTCGACAACTTAGTGAGAAGC<br>CTTGGCGAAGATCACTATGTTAGTGATGCAGGGGATGTCGTGTTCTATCAGTCTGA<br>CCCAGACATCTTCAAGTTTTCCAAGTCCAAGTCAGCCTAA
FAD3d-1	ATGGCGAGCTGGGTTCTATCAGAATGCGGCATAAAGCCACTCCCTTCCGCCTTCC<br>CTAAGCCCAGAACCGGAGCGCTTTCCCGAAATACCCTCCCCAAGCTCAGGTATTT<br>ACCCCCGAGGAGCAATCTCTCCGCCGCTGCCGACGGCGGCGCCTCAGATCTAAGC<br>CTTAACCTACCTAAATTATCCTCCGTAGAGAAGAAAAAGCCGCTTTTGGGACAGG<br>TGAGAGTTACTGCTCCGTTCAAGGTCGCTCCGGTGAAGGAGGAAGACGGAGAAG<br>GAGAGTCCAATTTCGACCCAGGAGCGCCGCCGCCGTTCAATTTGGCCGACATTCG<br>AGCTGCTATACCCAAGCATTGCTGGGTTAAAGATCCATGGAGGTCAATGGCTTAC<br>GTGGCGAGGGACGTGGCCGTTGTTCTCGGATTGGCAGCTGCCGCAGCTTATCTGA<br>ACAATTGGATCGTTTGGCCGCTGTATTGGGCGGCTCAGGGGACCATGTTCTGGGCT<br>CTCTTTGTTCTTGGCCATGACTGGTGAGTAAAGTTGTTAACTTTCTCTTTGGTCTTTT<br>ATGCTAATCTTTGTAATAATGGAAAATTGAAGAAAAATTCTCATGTTTGGCAGTG<br>GACATGGGAGCTTTTCAAGCAATCACAAGCTGAACAGTGTAGTTGGACACATTCT<br>CCATTCTTCAATTCTTGTGCCTTACCATGGATGGTAACTAAAGCTCTCTGCTTTCCT<br>CTGCCCATCTGTCATTTTGTTCTTCAATTTTGAAGCAATTTGAAACAAATTTTGTAT<br>CAATGTCTTTACAGGAGAATTAGCCATAGGACGCATCATCAGAACCATGGACATG<br>TTGAGAACGATGAGTCATGGCATCCTGTGAGTCTTTGTTTCTAAATCTTATCTATA<br>GGATTGATTCAGATGCTGATGTACTAATTTCTGTGACTTTTTCTGTTTTGTTTCTGTT<br>CTTGAAGTTGTCTGAGAAGATATTCAGGAGCTTGGATGCTACAACTCGAAGTATG

	AGGTTCACATTGCCTTTCCCCATGCTTGCTTATCCTTTCTATCTGGTGAGTTTGTATC<br>TACAAGAAAGTTCCTTTACAAAATGTGGATTTAGTTAACAGAAGCTAAAAGGGTT<br>CTTTCTTCTTGTTCCGTAATGCAGTGGAACCGGAGTCCTGGAAAAAAGGGGTCTC<br>ATTTCGACCCGAGTAGTGATTTGTTTGTGGCGAGTGAGAAGAAAGATGTGATCAC<br>TTCCACTGTTTGTTGGACAGCTATGGCTGCTTTGCTCGCAGGCTTGTCCTTTGTAAT<br>GGGTCCAATTCAGATGATCAAGCTATACGGAATTCCATATTGGGTATAGACAACA<br>TCTTTTGCCTTATCTTGCAAACTTGTTGTTTCAACTCTCAACCAACCACTCTGCCTC<br>TTGGATGTTTTTGTAGGGATTTGTTATGTGGTTGGATTTTGTAACTTACTTGCATCA<br>CCATGGTCACGACGATAAGCTTCCTTGGTACCGCGGCAAGGTAATCCATGTTCTTT<br>CCTCCTGTGTCCATCTATCTGGTCTTTCATGTTGAATCACTATATCCTAAAATCTTT<br>TCAATGTATAATTATTGATTCAAACCAAATGAAAAGTCAATTGTTTTGAGATGTCT<br>TTCTAGACTTTCAGTGAGATTTTGTTGAACTTATGCTTGGAACTTGGAACAGGAAT<br>GGAGTTACTTGAGAGGAGGGTTAACCACCATTGATAGAGACTATGGATGGATCA<br>ACAACATCCACCACGATATTGGAACCCATGTTATTCACCATCTCTTCCCACAAATC<br>CCTCACTACCACTTGATTGAAGCAGTAAGCAATAACTTTCCTTTTTCCCTTTAGTTC<br>CATATGCAATCTCTTCAATGGGGACACTGAGAAAAAGAAACAAACTTTGACCTG<br>AAAACAAATATCATGATGTTTCAGACGGAAGCTGCAAAGCCAGTGCTTGGGAAG<br>TACTACAAGGAACCAGCAAAATCAAAGCCGCTCCCTTTCCACCTAATCGGAGACT<br>TGATAAAGAGCTTGAAGAGAGACCATTATGTTAGTGACACCGGAGATGTCGTCTA<br>CTATCAAACTGACCCTGAACTCCAAAGACCCTCATCATCATCATGA
FAD3d-2 (wrong sequence)	ATGGCGAGCTGGGTTCTATCAGAATGCGGCATAAAGCCACTCCCTTCCGCCTTCC<br>CTAAGCCCAGAACCGGAGCGCTTTCCCGAAATACCCTCCCCAAGCTCAGGTATTT<br>ACCCCCGAGGAGCAATCTCTCCGCCGCTGCCGACGGCGGCGCCTCAGATCTAAGC<br>CTTGTCGTGGTATTGCAGGCTGCCGCAGCTTTTCTGAACAATTGGATTGTTTGGCC<br>GCTGTATTGGGCGGCTCAGGGGACTATGTTCTGGGCTCTCTTTGTTCTTGGCCATG<br>ACTGGTGAGTGAAAATTCTCAATTTTCGCTACTGTGATTACTAATCTGATGTAAAA<br>AGTGGGTGGAAAGTTCTCATTTTTCCTTTTGCAGTGGACATGGGAGCTTTTCAAGC<br>AATCACAAGCTGAACAGTGTAGTTGGACACATTCTGCATTCTTCAATTCTTGTGCC<br>TTACCATGGATGGTAAAAAGCTCTCTGCTTTCCTCTGCTCATCCGCCATTTTGTTCT<br>TCAAGTTTGAAGCAATTGAAAAAATAATTGTATCAATGTCTTTACAGGAGAATTA<br>GCCATAGGACGCATCATCAGAACCACGGCCACGTTGAGAACGATGAGTCATGGC<br>ATCCTGTGAGTCTTTGTATCTAAATCTTAGCCATAGGGTTGATTCAGATGCCGATG<br>TACTAATTTCTGTGACTTTGTTTTGTTTCTGTTCTTGAAGTTGTCTGAGAAGATATTC<br>AGGAGCTTGGATGCTACAACTCGAAGTATGAGGTTCACATTGCCTTTCCCCATGCT<br>TGCTTATCCTTTCTATCTGGTGAGTTTGTAGCTGCAAGTAAGTTTCTTTACAAAATA<br>TGGATTTAGTTAACGAAGCTAAAAGGGTTATTTCTCCTAATGCAGTGGAACAGGA<br>GTCCTGGGAAAAAGGGGTCTCATTTCGACCCGAGTAGCGATTTGTTTGTGGCGAG<br>TGAGAAGAAAGATGTGATCACTTCCACTGTTTGTTGGACAGCCATGGCTGCTTTGC<br>TCGCAGGCTTGTCCTTTGTAATGGGTCCAATTCAGATGATCAAGCTCTACGGAATT<br>CCATATTGGGTATAGACAACATCTTTTGCCTTATCTTACAGACTTGTTGTTTCAACT<br>CTCAACCAACCACTCTGCCTCTTTGATGTTTTTGTAGGGATTTGTTATGTGGTTGGA<br>TTTCGTAACTTACTTGCATCACCATGGTCACGACGATAAGCTTCCTTGGTACCGCG<br>GCAAGGTAATCAATGTTCTTTGCTCCTGCGTCTATCTATCTAGTCTTTCATGTTTAA<br>TCACTATATCCTAAAATCTTTTCAATGTATAATTCTTGATTCAAACCCGATGAAAA<br>GTCAATTGCTTTGAGATGTCTTTCTAGGCTTTCAGTGACATTTTGTTGAACTTGTGC<br>TTGGAACTTGGAACAGGAATGGAATTACTTGAGAGGAGGGCTAACCACCATTGA<br>TAGAGACTATGGATGGATCAACAACATCCACCACGATATTGGAACCCATGTCATT<br>CACCATCTCTTCCCACAAATCCCTCACTACCACTTGATTGAAGCAGTAAGCACTA<br>ACGTCCCATTTTCCCTTTAGTTCCATACGCAATCTCTTCAATGGGGAAACTGAGAA<br>AAAGAAACGAACTTTGACCTGAAAACAAATATCATGATATTTCAGACGGAAGCT<br>GCAAAGCCAGTGCTCGGGAAGTACTACAAGGAACCGGCGAAATCAAAGCCGCTC<br>CCTTTCCACCTAATCGGAGACTTGATAAAGAGCTTGAAGAGAGACCATTATGTTA

	GTGACACCGGAGATGTCGTGTACTATCAAACCGACCCCGAACTCCAAAGACCCTC<br>ATCATCATCATGA
SAD2-1	ATGGCTCTCAAGCTCAACCCAGTCACCACCTTCCCTTCAACACGCTCCCTCAACA<br>ACTTCTCCTCCAGATCTCCTCGCACCTTTCTCATGGCTGCTTCCACTTTCAATTCCA<br>CCTCCACCAAGTAAGCATCTCCTCCTCCTCGGAATCTCCGCCGATTTCTTTTAAGC<br>GATTGATCGTAGATAAATTTGTCGGTTGCTTACCGTTCATCAAAATCTGCACGGTT<br>CGTTTCTTCTTCTGCGCCTAGATTGCATTATGTCATTGTTCGTTTTCCGATTTGACTG<br>ACCGACATAAATCAATTCCTTTGTGTTTCACGATTCTGGGTTTTGCGCTGTAATTGA<br>TTGTCAGTGTTTGCACAGGTTTCCCCTTCTCCTCCTCCGTCCATCAAATGCATGTTA<br>TTACCATTTCAATTTCAGTTTCCTTCTCTGAAATATCCGTCTCTGGGAAAATAAGTC<br>TCTGTATCTACTATCCTATCAGCTTGTTTAGGAGAGGTTCGATATTCGTTTACATAA<br>ACCAATTGGCTTACAGTCCTTGAACGTTCTAAATGTTGGTCGCGGTGATAATAGGT<br>TCTCAAAAGAGGTTTGTCTATGTTGTTTGGCAAAATCTTGTTTCTGTGAATCATGTT<br>TAAGGTCCTTGGAAGAATGACTAATGAGCTATGACATGATTACGACGTAGTAGTT<br>ATTGAACTGCTGATAATTCAATATAGGGGTAACTTTGTTGATTGTTTGGTCACAGG<br>GAGGCTGAGAAGCTAAAGAAGTCACATGGACCACCAAAAGAGGTGCATATGCA<br>AGTGACCCATTCCATGCCCCCACAGAAGCTGGAGATATTTAAGTCTCTGGAAGGT<br>TGGGCTGAGGATGTTCTATTACCGCACCTGAAGCCAGTTGAGAAATGCTGGCAGC<br>CACAGGATTTCCTGCCCGAACCTGAGTCGGATGGGTTCGAGGAGCAAGTGAAGG<br>AGCTCAGGGCAAGGGCCAAAGAACTGCCCGATGACTATTTTGTTGTGCTGGTTGG<br>GGATATGATCACCGAAGAAGCTCTGCCGACTTACCAGACAATGCTCAACACCCTT<br>GACGGGGTGAGGGACGAGACTGGAGCCAGCCTTACGCCGTGGGCAATCTGGACA<br>AGGGCGTGGACCGCTGAAGAGAATAGGCACGGTGACCTTCTCAACAAGTATCTA<br>TACCTCTCTGGAAGGGTGGACATGAGGCAAATTGAAAAGACCATTCAGTATCTCA<br>TCGGCTCTGGAATGGTATGTAATCACATACTTCATCCTTTTCTATTAATCTTTGCGT<br>GAACAAAATTCACTACACTGGTAGCAGCTGAAACTTTAGATGATTTTTTTACTGC<br>CTAGCTTCTATGAAACAAAACCACGTAAGTCAAATAGGGTTGACAATGAGTTCAA<br>GTGGCAAAATTTTTCTTATATACCAACTTCGAACCACTTTATATGACATACCAACT<br>CCTAGTTCGGTTAAAATTCCTCCGTCGAAGATATAATACTTGGATTGGTTAAATGA<br>ATTGTGAAAGGATACACGTGATGTGGTCTGGAATTAATTTGTTTGAATGATCAGTT<br>GGGTTCGGGGCGACAACTGTGAACTGGAACCACCCTAAGTAAATTTTCTTTCTGTC<br>CTACAAATTTGAGGTTCTCCTTGATCACCTTAGTCCATCTTAGGTTTGCCCGTTAGT<br>AAGATCTGCATTTAGCAGTTTGTCCTGGTATTTGATATCACTAGTATCTTTGTTTGA<br>TTCCCTAGCATCTCTGAAACCATCGGACAAGTAGGTGGTTTAGGACAAATTTGGTT<br>CATTGCGGCATTTTTTGTTTGTATCGCCGTATCATCTGGAAGAAGCAGACAGTTTT<br>GCAAAGTGGCATCAAGCTCAAGAAAGCAACGGCTAGAAGAAGTTCTACATCTGA<br>TGCTTTCCTTTTGTTTCTTTGTGTGCTTTTTGGACTTTGTTCTTTTTTCCTGTAGGATC<br>CAAAAACAGAAAACAACCCCTACCTCGGTTTCATCTACACCTCATTCCAAGAGAG<br>GGCAACGTTCATCTCCCACGGAAACACAGCCAGACTCGCCAAGGACCATGGGGA<br>CATGAAGCTGGCGCAGATCTGCGGGATCATCGCAGCAGACGAGAAACGGCACGA<br>AACCGCATACACCAAGATCGTCGAGAAGCTCTTCGAGATCGACCCTGACGGTAC<br>AGTGCTGGCACTGGCGGACATGATGAGGAAGAAGATATCGATGCCCGCCCACTT<br>GATGTACGATGGAGAAGACGACAACCTCTTCGACAATTACTCGTCAGTCGCTCAA<br>CGCATCGGGGTGTATACTGCCAAGGATTATGCCGATATCCTGGAGTTCCTGGTGG<br>GGAGGTGGAAAGTGGATGCTTTTACGGGGCTTTCCGGGGAAGGGAACAAAGCTC<br>AGGATTTTGTCTGCGGGCTTCCTGCGAGGATTCGAAAGTTGGAGGAGAGGGCTGC<br>GGGGAGGGCAAAGCAAACGTCGAAATCTGTCCCGTTCAGCTGGATCTTCAGCAG<br>AGAATTGGTACTCTAA
SAD2-2	ATGGCTCTCAAGCTCAACCCAGTCACCACCTTCCCTTCGACCCGCTCCCTCAACA<br>ACTTCTCCTCCAGATCTCCTCGCACCTTTCTCATGGCTGCTTCCACTTTCAATTCCA<br>CTTCCACCAAGTAAGTTCCCGTCACCATCTCCTCTTCCTCGGAATCTCCGCCGTTTC<br>ATTTAAGCGATTGATCGTAGAAAATCTGTCGGTTGCTTAGCGTTCATTCAAATCTG<br>CGCGGTTCGTTTCTTTTTCTTTCTTCAGACTGCCTCGTCTGCATTATGTTATTGTTCG

	TTTCCGATTTGACTAACCTACATAATCAATTCCTTTGTGTTTCACGAGTCTGGATTT<br>TGCGCTGTAATTGATTGTCAGCGTTTGCACAGGTTTCCATTTCTCCACCTCCGTCCA<br>TCAAATGCATGTTATTACCTACCAATTTCAGCGTCTTTCTCTGGAAATTTCTGTCTC<br>TGTATCTACTATCCTATTAGCTTGTTTGAGAGAGGTTCAATATTGGTTTGCATGAAC<br>CAAGTGGCTTACAATCCTTCAACGTTCTAAATGTTGGTCGCAGTAACAATAGGTTC<br>TCAAAAGAGGTTTTTCTATGTTGTTTGGCAAAATCTTGTTTCTGTGAATCATGTTAA<br>GGTCCTGGGAAGAATGATTAATGAGCTATGACATGATTAAGGCGTAGTAGTTATT<br>GAACTGCTGATAATTCAATATAGGGGTAACTTTGTTGGTTGTTTGGTGACAGGGAG<br>GCTGAGAAGCTAAAGAAGTCACATGGACCACCAAAAGAGGTGCATATGCAAGTG<br>ACCCATTCCATGCCCCCACAGAAGCTGGAGATCTTTAAGTCCCTTGAAGGTTGGG<br>CAGAGGACGTTCTGTTGCCGCACCTGAAGCCGGTTGAGAAATGCTGGCAGCCACA<br>AGATTTCCTGCCCGAACCCGAGTCGGATGGGTTCGAGGAGCAAGTGAAGGAGCT<br>CAGGGCAAGGGCTAAAGAACTCCCCGATGACTATTTTGTTGTGCTGGTTGGGGAT<br>ATGATCACCGAAGAAGCTCTACCGACTTACCAGACAATGCTCAACACCCTTGACG<br>GGGTGAGGGACGAGACTGGAGCCAGCCTTACGCCGTGGGCAATCTGGACAAGGG<br>CGTGGACCGCTGAAGAGAATAGGCACGGTGACCTTCTCAACAAGTATCTTTACCT<br>CTCTGGAAGGGTGGACATGAGGCAAATTGAAAAGACCATTCAGTATCTCATCGGC<br>TCTGGAATGGTATGTACTCACATCCTATCTGCTCCTTTATCCTTTTCCATTAATCTTT<br>GATTGAACAAAATTCAATAAACTGGTAGCTGAAACTTTAGATGATTTGTTATAAC<br>TGCCTAGCTTCTATGAGAAAACCACTGAAGTCAAATAGGTTTGACAATGGGTTTA<br>AATGGAAAAAGTTTCATATACCATCTTCCATCTATTTTACATGACATACCAACTTC<br>TACTTCGGAGAAAATTCGCCGTGGATAATCATATTATTGAAGATATAGTACTTAGT<br>AGATTGGTTAGATGAACTGTTAAACAATACATGTGATGTCGTGTGCAATTAATTTG<br>TGTAAATGATTAGCTGGGTTCGGGACGACAAATGTGAACTGGAACCCTAGTAAAC<br>TATGAATTGAGGTTGTCCTTCATCACTTTATTCTGTCCTGGGTTTGTTTGCCTGTTTG<br>CAAGATCTGCATGTAGCAGTTTGTCCTGGTATTTGCTACCAGTGGTATCTTTGTTTG<br>ATTCCCTAGCATCTCTGAAAACATCGGACCAAGTATCTGGTTAGGACAAATTTGG<br>TTCATTGCGGCATTTTTTGTTTGTATCGCTGTATCGTCTGGAAGAAGCAGACAGTTT<br>TGCAAAGTGGCATCAAGCTCAAGAAAGCAACGGCTAGAAGAAGTTCTACATCTG<br>ATGCGTTCCTTTTGTTTCTTTGTGTGCTTTTTGGACTTTGTTCTTTTTGCCTGTAGGAT<br>CCAAAAACAGAAAACAACCCCTACCTCGGTTTCATCTACACCTCATTCCAAGAGA<br>GGGCAACGTTCATCTCCCACGGAAATACGGCCAGACTCGCCAAGGACCACGGGG<br>ACATGAAGCTGGCGCAGATCTGCGGGATCATCGCAGCAGACGAGAAGCGGCACG<br>AAACAGCATACACCAAGATCGTCGAGAAGCTCTTCGAGATCGACCCTGACAGTA<br>CAGTGTTGGCTCTGGCGGACATGATGAGGAAGAAGATATCGATGCCCGCACACTT<br>GATGTACGATGGAGAAGACGACAACCTCTTCGACAATTACTCGTCGGTCGCTCAA<br>CGCATCGGGGTGTATACTGCCAAGGATTATGCTGATATCCTGGAGTTCCTGGTGG<br>GGAGGTGGAAAGTGGATGCTTTTACGGGACTTTCCGGGGAAGGGAACAAAGCTC<br>AGGAGTTTGTCTGTGGGCTTCCAGCGAGGATTCGAAAATTGGAGGAGAGGGCTGC<br>GGGGAGGGCAAAGCAAACGTCGAAATCTGTCCCATTCAGCTGGATCTTCAGCAG<br>AGAATTGGTACTCTAA
SAD3-1	ATGCAAGCCACCCACCACGTCAGCGCCCACCGTCCAGCATGGATGCCACGTGGT<br>GGCCGAACCACCTCCACCATCATCATGCACCTCTGCCCTCCACGCGCCGCCATCA<br>AAACTGCTCCGCCGCCTCCCCCTCCGCTCACCCTGAAACACCAACACAAAACCCA<br>CACAATGCCGGCGGAGAAAATCGAGCTTTTCAAGTCCCTCGAGGATTGGGCCTCC<br>GAGAATGTCCTCCCACTTCTCAAGCCCGTCAATAAATGCTGGCAGCCTCAGGACT<br>TCCTCCCCGACCCGTCCCTCTCCGTCGCTGATTTCTCCGACGAGGTAATGAACAAT<br>TGCGTCTCAATACCTTGTATAAGCTACACTTTTACAGGGATTTTGAGCTAATATTT<br>GACAAATTCATCAGTTAGGTTTTTTGTCATTACGGATTAGTCATTGTAGTTTTGTCA<br>TTTGTTCATATAGCTACTCATTTTTCAGTTCATCAAATTCTCATTTTCTATAGTAAG<br>ATCATTTGGAAGGGTTTTGGAGAGTAATTTCTTGGTCCCAGATGATTTGATCGAAA<br>TATCATTTTAGAATACAAAAATAAATTGGGATTTGAAATCACCTCGATAGGTTAG<br>GTTTCCCAAAAGGTGATTTCAGGCTCCCAAAAGGTCCCAAAAGGTGATTTCAAAT

	TCATTTTATGGACTTGAGATTTATAAAATCTCAAGTAATTTGAATAGTAAATGATC<br>AAATTATCTTGTTGAGTTTAAGTGAACGAGATAAATTAAGAGGTGCATTAGTCATT<br>TAAAAAATCAATTTTTTTTTAATTTTCAAAACTATTTTGTGATTGACCGTTAAACC<br>AGTTCGGTTTGATACTGATTTTGAGTTTTGACTGACAGGTGAGGTTGTTGAGGGAA<br>AGGACGGCGGAGCTACCGGACGAGTACTTCGTCGTCTTGGTGGGTGACATGATAA<br>CGGAGGATGCGTTACCGACGTACGAGACCATGATCAACACACTTGACGGCGTTA<br>GGGACGAGACGGGCGCCAGTCAAAGCCCATGGGCCTTATGGACCCGGGCCTGGA<br>CCGCCGAGGAGAATCGCCACGGCGATCTGCTTCGGACCTTTCTGTACCTATCGGG<br>TCGGGTCGACATGAGGATGGTAGAAAGGACTGTTCAGTACCTCATTGGATCTGGC<br>ATGGTAAGTGTAGAATAGCCCTTTCGGTATTCCGAACCGGTTTGATCCGTCAGTGG<br>GATCTTTCGTTCGATTAATCGGGTGATGAGTATGCCAAACTGGCACTAATTTATCA<br>CTTGATTTGATAGTACATTTTATAAGCTAGTGTTAGCTAGTTACATTGTTTGAGAAT<br>GTCACGAATTCGAAGGAGAAAAAAACCAAAATGCCACTTCGATAATTTATTTTAC<br>ATGATATAGTCATTGAACCTTCAATCTTTTTACAAATCGGATCCAAATTTGTGTAC<br>GCAGTGGTCACTCAACTGTCATTTTAGAATAGGAATAGCATTTTAACAAGTGATTT<br>ACTCGGGTCGGGTAACATTACAGGACCCGGGCACAGAGAACAACCCGTACTTGG<br>GGTTCGTGTACACGTCATTCCAGGAGCGAGCCACATTCATATCGCACGGCAACAC<br>GGCTCGGCTAGCCAAGGAGGGAGGGGATCCGGTACTCGCACGGATATGTGGCAC<br>CATAGCATCGGACGAAAAGCGACACGAGAACGCCTACACGCGCATAGTGGAGA<br>AGCTCCTAGAGGTGGACCCGAACGGTGCGGTTTGTGCGGTGGCCGACATGATGCA<br>TAAGAAGATCACAATGCCGGCGCACCTGATGTACGACGGGGAGGACCCGAAGCT<br>GTTCGAGCACTTCTCATCGGTGGCTCAGCGGCTCGGGGTGTACACCGCGCAGGAC<br>TACGCGGACATACTCGAGTTCTTGATCGGACGGTGGGGGCTGGACAAGGTGGAG<br>CAAGGGCTGGACGGTGAAGGGAGGCGGGCGCAGGATTTCGTGTGTGGGCTGCCA<br>CGGAGGATCATGCGGCTCCAGGAGCGAGCCGATGAGAAGGCTAAGAAGATGAA<br>GAAGCCGGAAGCAGTCAAGTTTAGCTGGATTTTCAAACGGCAAGTCGTTTTGTGA
SAD3-2	ATGCACCTCTGCCCTCCACGCGCCGCCATCAAAACTGCTCCGCCGCCGCCTCCTC<br>CGCTCGCCCTGAAACACCAACACAAAACCCACACAATGCCGGCGGAGAAAATCG<br>AGCTTTTCAAATCCCTCGAGGATTGGGCCTCCGTCAATGTCCTCCCACTTCTCAAG<br>CCCGTCGACAAATGCTGGCAGCCTCAGGACTTCCTCCCCGACCCGTCCCTCTCCGT<br>CGCTGATTTCTCCGACCAGGTAATGTACATTGTGTTCCAATAACTCGTATTGTAAT<br>GCTACTCGTATTTTGGGCTAATATTTGATAAATTCGTCACTTAACTTTTTTGTCTTG<br>ACGGATTAGTCATTGTAGTTTTATTATTTGTTCGATTTTCAGTTAAATAATTCTCATT<br>TTTTATAGTAAGATCATTTGGAAGGAATTTGGAGAGTAATTTCATGGTCCTGGGTT<br>GATTTGATCGAAATATCGCGTTGGAACACGAAAATAAAGATGGATTTGAGTAGA<br>AAATAACCAAATTGACTTGTTGAAATTGTATTGTTGAGTGAACAGGGGGTTTATTA<br>GTCATTTACAAAATTAAAATGTTTCTTTTAATGTCCAAAACTCAACAAAACACTGA<br>TTTTGAGATTGGCCGTTAAACCAGTTCGGTTTGATAAATACTGATTTTGAGTTTTGA<br>CTGACAGGTGAGGTTGTTGAGGGAAAGGACGGCGGAGCTACCGGACGAGTACTT<br>CGTCGTCTTGGTGGGTGACATGATAACGGAGGATGCGTTACCGACGTACGAGACC<br>ATGATCAACACCCTTGACGGCGTTAGGGACGAGACGGGCGCCAGTCAAAGCCCA<br>TGGGCCCTGTGGACCCGAGCCTGGACCGCCGAGGAGAATCGCCACGGGGATTTG<br>CTCCGGACCTTTCTGTACCTCTCGGGTCGGGTCGACATGAGGATGGTTGAAAAGA<br>CTGTTCAGTACCTCATTGGATCTGGCATGGTAAAGTGCAGAACAGTCCTTTTTGTA<br>TTCCGAACTGGTTTGATCGGTTGGTCGGTTCGTTCGTTTGTTAATTGGGTGATGAGT<br>ATGCGAAATTGTCACTAAATTATCATTTCATAGTACATTTATAAGCTAGTGTTTAG<br>TAACAATTGTTTAAGAATGTTACAAATTTGAACCGGGAAAAAACCAAAATGCCA<br>CTTGGATAATTTATTTTGCATGATATAGTCATTGAACCTTTAATCTTCTTACAAATC<br>GGATCCTAATTTGTGTACACATTGGTCACTCAACTGTCATTTTAGAATAGGAATAG<br>CATTTTAACAAGTGATTTACTCGGGTCGGGTAACAATTGCAGGACCCTGGCACGG<br>AGAACAACCCGTACTTGGGGGTTCGTATACACGTCATTCCAGGAGCGAGCCACATT<br>CATATCGCACGGCACCACGGCTCGGCTAGCCAAGGAGGGAGGGGATCCGGTACT<br>TGCACGAATATGCGGCACCATAGCATCGGATGAGAAGCGACACGAGAACGCCTA

	CACGCGCATCGTGGAGAAGCTCCTCGAGGTGGACCCGAACGGTGCGGTTTGTGCC<br>GTGGCCGACATGATGCGTAAAAAGATCACAATGCCGGCGCACCTGATGTACGAC<br>GGGGAGGACCCAAAGCTGTTCGAGCACTTCTCGGCGGTGGCGCAGCGGCTCGGG<br>GTGTACACGGCGCAGGACTACGCGGACATACTGGAGTTCTTGATCGGACGCTGGG<br>GGCTGGACAAGGTGGAACAAGGGCTGGACGGTGAAGGGAGGCGGGCGCAGGAT<br>TTCGTGTGTGGGCTGCCGCAGAGGATCATCCGACTCCAGGAGCGAGCCGATGAG<br>AAGGCTAAGAAGATGAAGAAGCCGGAAGCCGTCAAATTTAGCTGGATCTTCAAA<br>CGGCAAGTCGTCTTGTGA

Note: Exon sequences are highlighted in gray. * Additional *FAD2b-2* and *FAD2c-1* were revealed for cultivar Atlant, which could be a result of errors in the Atlant genome assembly.
